# Cationic Carbene Analogues: Donor‐Free Phosphenium and Arsenium Ions

**DOI:** 10.1002/anie.202107975

**Published:** 2021-07-26

**Authors:** Marian Olaru, Stefan Mebs, Jens Beckmann

**Affiliations:** ^1^ Institut für Anorganische Chemie und Kristallographie Universität Bremen Leobener Straße 7 28359 Bremen Germany; ^2^ Institut für Experimentalphysik Freie Universität Berlin Arnimallee 14 14195 Berlin Germany

**Keywords:** arsenium ions, carbene analogues, cations, Lewis superacids, phosphenium ions

## Abstract

Carbenes and their analogues have constantly enthralled chemists with their intriguing reactivity of ambiphilic character stemming from their electronic structures. Phosphenium and arsenium ions are fiercely reactive cationic species, the stabilization of which has been so far achieved in the condensed phase by dispersing the positive charge through electromeric conjugation with at least one electron‐rich substituent (frequently amido groups). Although observed in the gas phase, the isolation of dicoordinate phosphenium and arsenium ions lacking such stabilizing ligands has eluded chemists for decades. Herein we show that by judicious choice of aromatic substituents, dicoordinate, donor‐free, Lewis‐superacidic phosphenium and arsenium ions can be kinetically stabilized. They feature singlet electronic ground states possessing a vacant p‐orbital and an electron lone pair with predominantly s‐character.

## Graphical abstract

Donor‐free, kinetically stabilized diarylphosphenium and arsenium ions were obtained using a combination of bulky rigid aryl substituents and a weakly coordinating anion.

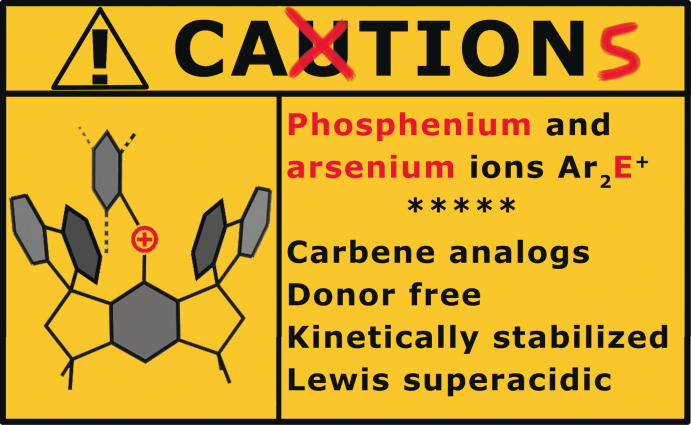

Phosphenium ions [R_2_P]^+^ (**I**) and the heavier congeners [R_2_E]^+^ (E=As, Sb, Bi), in their native form, are highly reactive, six‐valence electron species that comprise an electron lone pair as well as a vacant p‐orbital (Figure 1 A). They are isoelectronic to the neutral carbenes [R_2_C] (**II**) and to their congeners of heavier group 14 elements [R_2_E] (**II**, E=Si, Sn, Ge, Pb),^[1]^ however, due to the positive charge, they also closely parallel the strong Lewis acidity of carbenium and silylium ions [R_3_E]^+^ (**III,** E=C, Si).^[2]^ Unlike the latter, only two substituents are available to shield the pnictogen atom in [R_2_E]^+^ (**I**) and to prevent the counterion from coordination. Consequently, all low coordinate pnictogenium ions known in the literature are stabilized if at least one strongly electron‐donating substituent (Figure 1 B), usually in the form of amido or thiolato moieties, is bonded, often directly, to the highly electrophilic pnictogen. Alternative stabilizing strategies include the use of electron rich (pentamethyl)cyclopentadienyl groups or additional Lewis bases, which increase the coordination number and help dispersing the positive charge from the pnictogenium ion. This holds true for heteromethine systems in which the amino group is connected to the pnictogen atom via (extended) C=C double bonds.[^3^, ^4^, ^5^] The special case of [Fc_2_P]^+^ (Fc=ferrocenyl) reported by Cowley et al. in 1981, allegedly lacking any donor atoms, was recently reinvestigated and shown to feature stabilizing Fe⋅⋅⋅P interactions (Figure 1 C). They are strong enough to distort the ideal coordination geometry around phosphorus and moderate the Lewis acidity.^[6]^ Lighter pnictogenium ions lacking ligands and substituents capable of effective charge delocalization have been so far observed only in the gas phase.^[5a]^ Thus the isolation of such species is of paramount importance not only from a fundamental perspective but also in light of the various Lewis superacid‐enabled applications (e.g. small molecule activation).[^7^, ^8^, ^9^]

**Figure 1:**
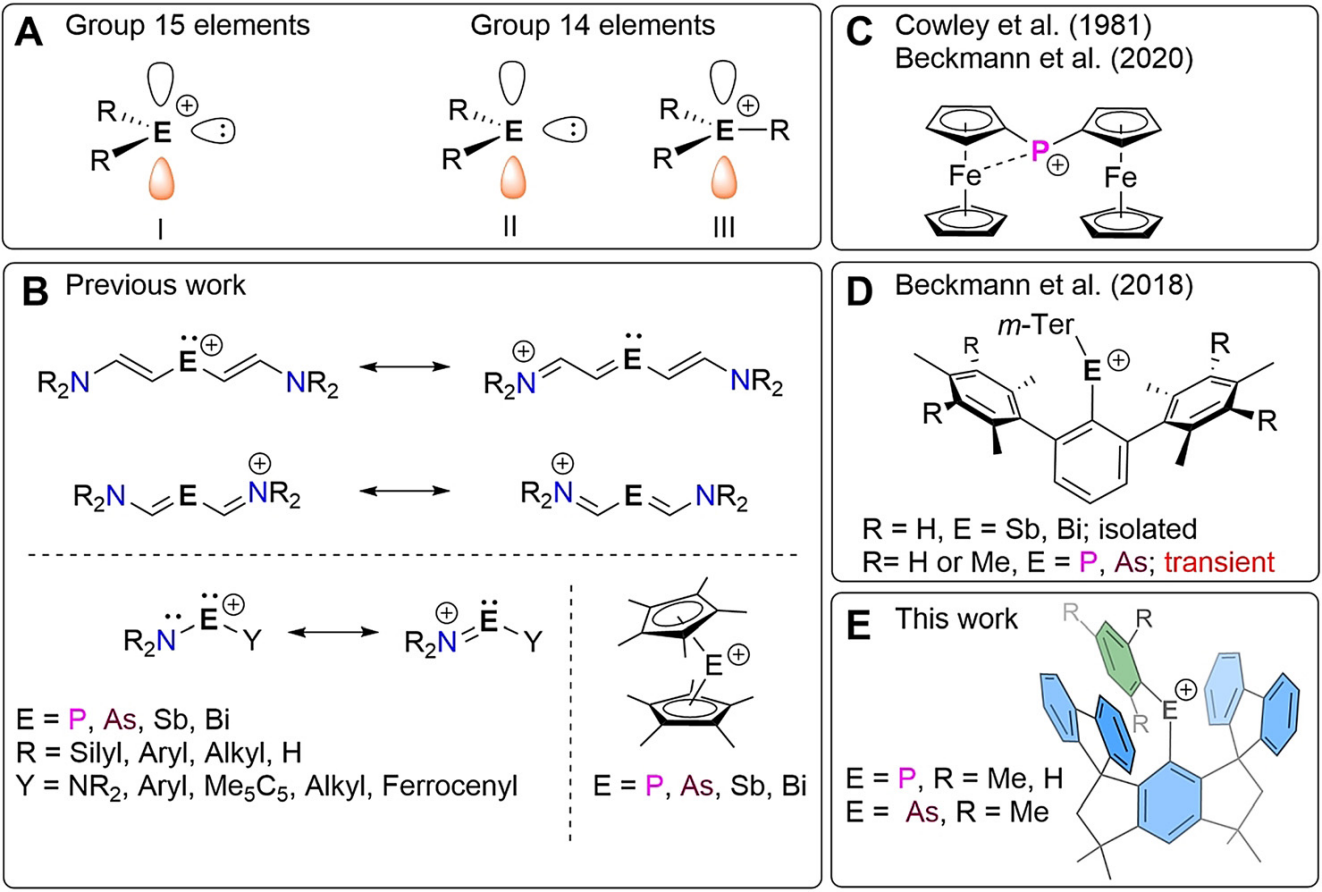
(**A**) Isoelectronic relationship between six‐valence electron species of group 14 and 15. (**B**) Well established stabilization methods of pnictogenium ions by charge delocalization.[^3^, ^4^, ^5^] (**C**) Stabilization of [Fc_2_P]^+^ by Fe⋅⋅⋅P interactions.^[6]^ (**D**) The first examples of kinetically stabilized divalent heavy pnictogenium ions [*m*‐Ter_2_E]^+^ (*m*‐Ter=2,6‐Mes_2_C_6_H_3_, E=Sb, Bi).[^10^, ^12^, ^13^] (**E**) Kinetically stabilized diarylphosphenium and ‐arsenium ions discussed in this work.

Recently, we have demonstrated that the donor free dicoordinate stibenium and bismuthenium ions can be kinetically stabilized by using two *m*‐terphenyl groups (Figure 1 D).^[10]^ Since then, our systematic efforts to apply a similar strategy for the isolation of the lighter phosphenium and arsenium congeners have failed to yield the targeted salts. In this endeavor we have noticed that the halide abstraction reagent must be carefully considered,^[11]^ and that when generated, the phosphenium and arsenium ions are shortly lived and react intramolecularly with one of the flanking aryl groups of the *m*‐terphenyl substituent.[^12^, ^13^] From these observations it became clear that the free rotation of these flanking aryl groups, which should confer steric protection around the pnictogen is of serious concern. We have thus turned our attention to the tetramethyl‐s‐hydrindacen‐4′‐yl based substituents,^[14]^ more specifically to the 9‐fluorenyl substituted one,^[15]^ which is both rigid and able to confer sufficient steric protection around the pnictogen. Herein we report the synthesis, structural and electronic characterization of the first Lewis base free phosphenium and arsenium ions [Ar^1^Ar^2^E][B(C_6_F_5_)_4_] (E=P, Ar^2^=Mes (**6**), E=As, Ar^2^=Mes (**7**); E=P, Ar^2^=Ph (**8**); Ar^1^=dispiro[fluorene‐9,3′‐(1′,1′,7′,7′‐tetramethyl‐s‐hydrindacen‐4′‐yl)‐5′,9′′‐fluorene]), lacking significant charge delocalization.

The reaction of organolithium species **1** with ECl_3_, followed by reactions of the resulting organoelement dichlorides (**2**, E=P; **3**. E=As) with MesLi (Mes=mesityl) gave diorganoelement monochlorides **4** (E=P) and **5** (E=As). The phosphenium and arsenium salts [Ar^1^MesP][B(C_6_F_5_)_4_] (**6**, 65 %) and [Ar^1^MesP][B(C_6_F_5_)_4_] (**7**, 78 %) were obtained in good yields by chloride abstraction from **4** and **5**, respectively, using K[B(C_6_F_5_)_4_] in CH_2_Cl_2_ at room temperature.

The phosphenium salt [Ar^1^PhP][B(C_6_F_5_)_4_] (**8**, 60 %) was obtained by an alternative one‐pot reaction starting from **1** and commercially available PhPCl_2_ (Figure 2). The formation of pnictogenium salts **6**–**8** was accompanied by dramatic changes of the colors of the reaction mixtures from colorless to dark blue‐green for **6**, blood‐red for **7** and dark purple for **8**. The UV/Vis spectra of these species (Supporting information) show absorption maxima at 446 and 589 (**6**), 435 and 510 (**7**), 358, 419 and 561 nm (**8**). Under inert atmosphere **6**–**8** are stable at room temperature in solution (dichloromethane, at least several days) and solid state (months). They scavenge water to yield the colorless [Ar^1^Ar^2^EH(OH)][B(C_6_F_5_)_4_] species, the phosphenium salts **6** and **8** much faster than **7**. When heated in 1,1,2,2‐tetrachloroethane, only **6** and **8** decomposed (slowly at 60–100 °C, faster above 125 °C, Supplementary Tables S1, S2) while **7** showed no decomposition after 1.5 h at 150 °C. The main decomposition pathway involves the breaking of one fluorenyl‐C_*spiro*_ bonds and the formation of a new phenyl‐phosphorus bond eventually giving a phosphonium ion with the same elemental composition as **6** or **8**. (more details in Supporting information).

**Figure 2:**
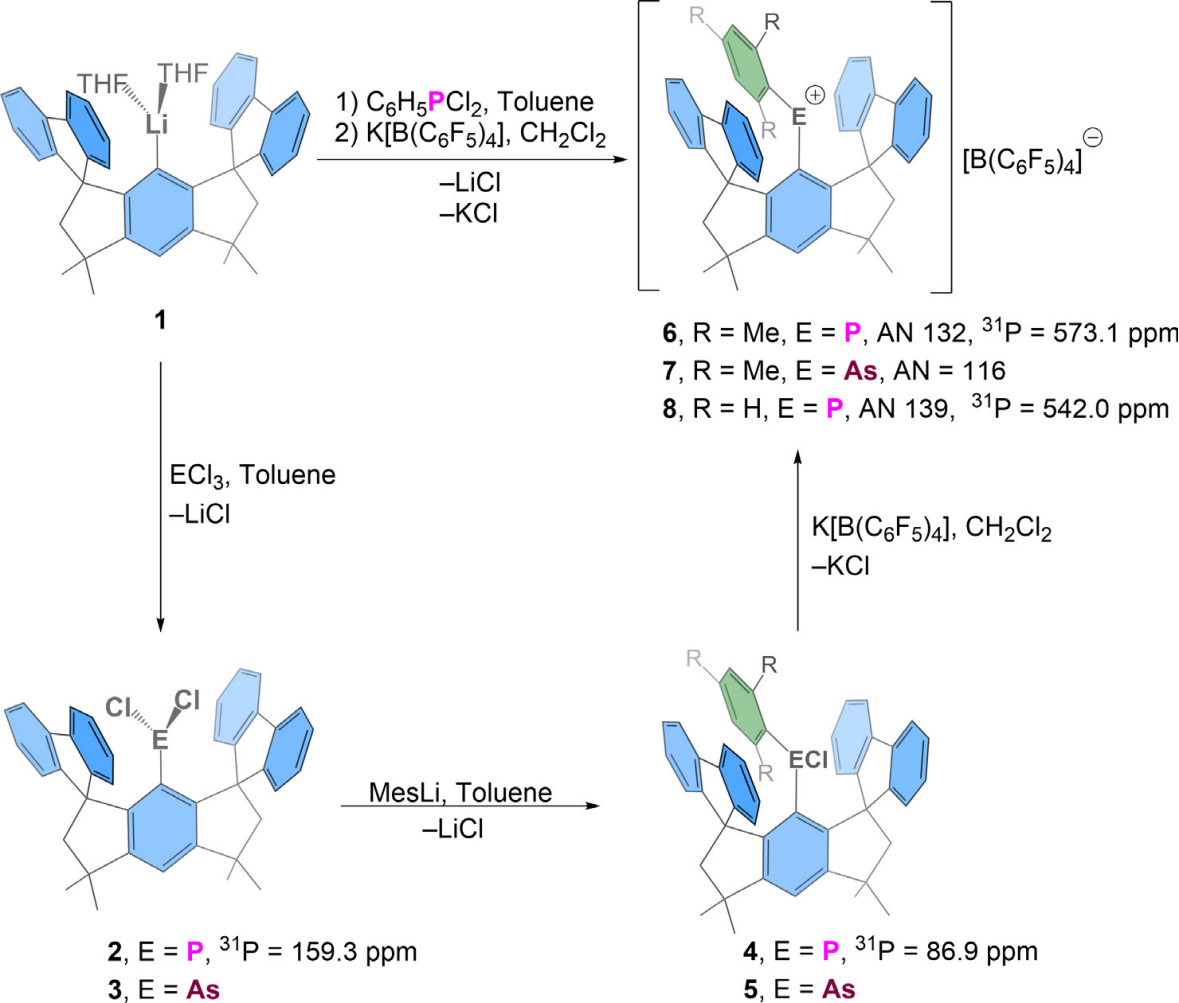
Synthesis of the pnictogenium ions **6**–**8**, their Gutmann‐Beckett acceptor numbers (AN) and ^31^P NMR chemical shifts of newly prepared compounds.

Inspection of the solid‐state structures of **6**–**8** (Figure 3) determined by single crystal X‐ray diffraction reveals several notable features.^[16]^ Firstly, there is clear separation between the cations and the weekly coordinating counter‐anion or solvent molecules (1,2‐F_2_C_6_H_4_ co‐crystallized with **8**). Secondly, the pnictogen atoms are essentially dicoordinated by just two aromatic substituents. The closest non‐bonding atoms relative to the pnictogen atoms are C55 (2.771(2) Å) for **6**, C35 (2.735(2) Å) for **7**, and C50 (2.674(2) Å) for **8** which are well outside the sum of the covalent radii of the respective elements (1.80(3) Å for C, P; 1.92(4) Å for C, As).^[17]^ We note that the shorter contacts observed for **8** can be explained by the smaller positive inductive effects of the phenyl substituent compared with the electron richer mesityl. Thus in **8** the positive charge at phosphorus is slightly less compensated, leading to shorter contacts with one of the flanking fluorenyl groups. Such contacts, although attractive, can be classified as dispersive interactions^[18]^ since they are within the van der Waals range (ca. 3.6 Å).^[19]^ In all three structures, the pnictogen atom deviates from co‐planarity with the central ring of Ar^1^. This is reflected by the deviation from the expected 180° value of the angle between the C10‐C15‐plane and the C10‐E bond [165.1(1)° for **6**, 163.8(1)° for **7** and 166.8(1)° for **8**]. The C‐E‐C bond angles for **6** (109.8(1)°) and **7** (108.6(1)°) are very similar and contrasting that of **8** (101.85°). The significantly larger C‐P‐C angle value of **6** compared with that of **8** is thought to arise from the steric pressure exerted by the mesityl group. The C−P bond lengths found in the phosphenium ions **6** (1.772(2) Å, 1.828(1) Å) and **8** [1.781(2) Å, 1.827(2) Å] although shorter than in **2** (1.864(2) Å) or the average P−C single bond lengths in Ph_3_P (1.830(2) Å)^[20]^ compare well to those found in [MesP(N(*i*‐Pr)_2_]^+^ (1.787(6) Å)^[21]^ or [Fc_2_P]^+^ (average 1.758(6) Å)^[6]^ and can be regarded as short single bonds. For comparison the C=P double bond length in the *p*‐phosphaquinone reported by Yoshifuji et al. is 1.705(2) Å,^[22]^ in the simple phosphalkene 2,4,6‐(*t*‐Bu)_3_C_6_H_2_P=CH_2_ is 1.643(3) Å,^[23]^ and in the phosphalkene reported by Cowley et al. is 1.686(5) Å.^[24]^ The C−As bonds lengths of **7** (1.921(2), 1.960(2) Å) are shorter than those found in **3** (1.984(2) Å) or the average C−As bond lengths in Ph_3_As (1.957(7) Å),^[25]^ but considerably longer than the C=As double bond found in the arsaalkene reported by Cowley et al. (1.807(3) Å).^[24]^ This indicates that in all cases the positive charge is not dispersed by electromeric conjugation with the aromatic rings.

**Figure 3:**
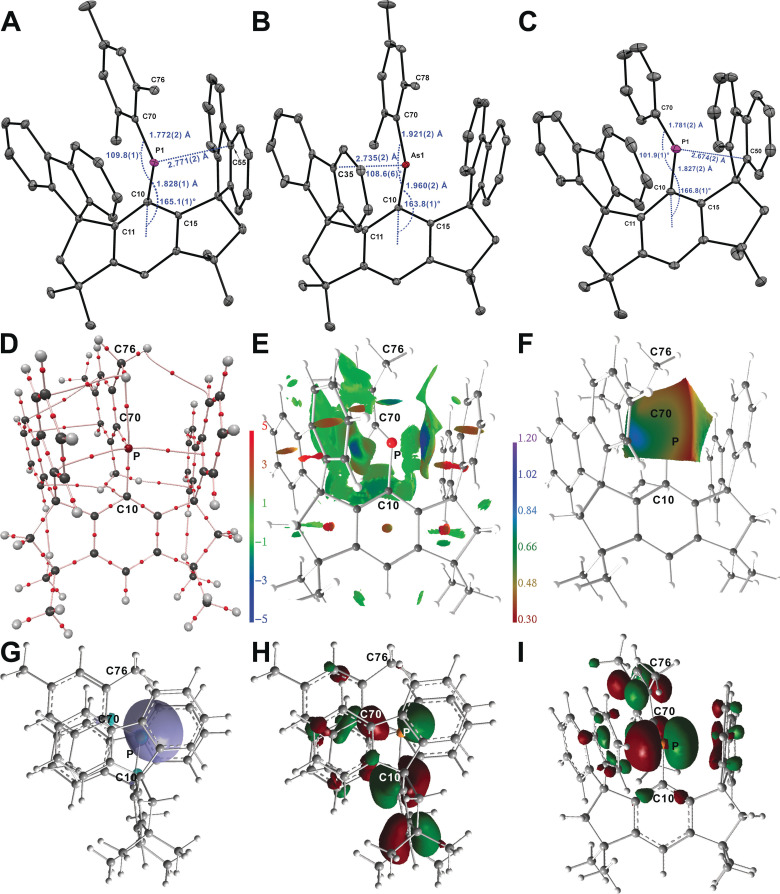
(A–C) Molecular structures of pnictogenium ions **6**–**8**. Thermal ellipsoids are set at 30 % probability. (D–I) Real‐space and orbital‐based bonding discriminators for **6** derived from DFT. (D) AIM bond topology; (E) NCI iso‐surface (*s*(**r**)=0.5); (F) ELI‐D distribution mapped on the ELI‐D basin surface of the P atoms LP; (G) iso‐surface (0.04 a.u.) of the P(LP) NBO; (H,I) iso‐surfaces (0.04 a.u.) of the HOMO−3 and LUMO.

The formation of phosphenium ions **6** and **8** was readily confirmed by ^31^P NMR spectroscopy. The ^31^P NMR (CD_2_Cl_2_) spectra showed resonance signals at 573.1 (**6**) and 542.0 ppm (**8**) that are characteristic for the highly deshielded low coordinate phosphorus nuclei. In fact the chemical shift of **6** is the most extreme value recorded for phosphenium ions, the next in line being reported for amido‐phosphenium ions containing one carbon substituent like [FcPNMe_2_]^+^ (558.5 ppm)^[26]^ or [*t*‐BuPNMe_2_]^+^ (513.2 ppm).^[27]^ The counterintuitive difference between the ^31^P chemical shifts of **6** (containing the electron richer mesityl substituent) and **8** substituted by a phenyl ring could be a consequence of shorter contacts between phosphorus and the fluorenyl flanking rings in **8**, in which case the P atom approaches more the shielding fluorenyl group thus causing the ^31^P resonance signal to shift lower by ca. 30 ppm. Nonetheless the ^31^P NMR chemical shifts of **6** and **8** are in very good agreement with the calculated ones (584.7 and 566.5 ppm, respectively). They are much smaller compared to the values calculated for [Me_2_P]^+^ (1497.1 ppm) where only minimal inductive effects are expected, but closer to that calculated for [Ph_2_P]^+^ (705.4 ppm). We believe the difference in this case arises due to magnetic shielding provided by the ring currents of two flanking fluorenyl groups. The ^1^H NMR spectra of **6**–**8** display sharp signals at room temperature indicating singlet electronic ground states. After assignment of the resonances, it was obvious that the structures from the solid state are retained in solution. The mesityl and phenyl moieties are sandwiched between the flanking fluorenyl groups and the protons located in the proximity of aromatic ring currents give resonances at characteristically low chemical shifts caused by magnetic shielding.

The Gutmann‐Beckett method,[^28^, ^29^, ^30^] which relies on monitoring by ^31^P NMR the change of the chemical shift of Et_3_PO after coordination to a Lewis acid, was used to probe the Lewis acidity of **6**–**8**. The acceptor numbers calculated based on this change (CD_2_Cl_2_) for **6** (132), **7** (117) and **8** (137) are, as expected, considerably larger than for B(C_6_F_5_)_3_ (82, CD_2_Cl_2_),^[31]^ [Et_3_Si]^+^ (105, C_6_D_6_)^[32]^ or [Fc_2_P]^+^ (116, CD_2_Cl_2_, Supporting information). The phosphenium ions **6** and **8** have marginally larger acceptor numbers than some recently reported doubly charged phosphorandiylium species (117, 129, CD_2_Cl_2_)^[33]^ and are only surpassed by [Me_5_C_5_P]^2+^ (163, 1,2‐F_2_C_6_H_4_)[^34^, ^35^] and the triply cationic salt [2,6‐Me_2_C_6_H_3_SiTerPy][OTf]_3_ (154, CD_3_CN; TerPy=terpyridine).^[36]^ We note that the Lewis acidity of phenyl substituted **8** is slightly larger than that of **6** in agreement with smaller stabilizing inductive effects present in the phenyl‐substituted **8**.

Density functional theory (DFT) calculations were conducted for **6**–**8** in order to elucidate fine electronic details of the chemical environment around the central P or As atoms (Figure 3 D–I and Supporting information). The Atoms‐In‐Molecules (AIM) molecular graphs^[37]^ disclose two secondary P/As⋅⋅⋅C_π_ interactions next to the primary P/As−C bonds. The P/As⋅⋅⋅C_π_ bond paths, however, are curved and in some cases seemingly violate the topological rules by ending in another bond critical point (bcp, see red spots in Figure 3 D), indicating very weak interactions. In the corresponding virial field function, which is experimentally not accessible but typically provides a more stable bond topology in case of weak contacts, the topology is straight forward as all bond paths end in atoms (Figure S88–90). With 0.16 to 0.19 e Å^−3^ for the electron density (ED, *ρ*(*r*)_bcp_), a Laplacian of the ED being positive and close to zero (∇^2^ *ρ*(*r*)_bcp_=1.0(1) e Å^−5^), and the kinetic energy density over ED ratio (*G*/*ρ*(*r*)_bcp_=0.44(1) a.u.) being significantly larger than the total energy density over ED ratio (*H*/*ρ*(*r*)_bcp_=−0.06(2) a.u.), these contacts are almost completely electrostatic, (Table S9). The even weaker and purely electrostatic LP(P)⋅⋅⋅H${}_{\mathrm{CH}{}_{3}}$ hydrogen bonds observed in compounds **6** and **8** already exhibit a positive *H*/*ρ*(*r*)_bcp_. The non‐covalent bond characteristics of the P/As⋅⋅⋅C_π_ contacts are also corroborated by the high bond ellipticities (*ϵ*=0.5 to 2.2), indicating a pronounced “smearing” of the ED. Concurring, the non‐covalent interactions index surfaces (NCI)^[38]^ and the electron localizability indicator (ELI‐D)[^39^, ^40^] distribution on the lone‐pair (LP) ELI‐D basins surface, both show extended areas of non‐covalent P/As⋅⋅⋅C_π_ interactions (Figure 3 E,F and Figure S88–90), thereby exceeding the localized atom‐to‐atom bonding picture given by AIM topology. Within the ELI‐D framework, the lone‐pair basins of **6**, **7**, and **8** show comparable electron populations like the P−C bonding basins (all between 2.3 and 2.6 e), but 2–3 times larger basin volumes and significantly higher localizabilities since they are not involved in primary chemical bonding (and thus electron sharing).

In equal manner, virtually identical natural bond orbital (NBO)[^41^, ^42^] occupancies of 1.94(1) e are observed for the LP and bonding natural orbitals in all three compounds (Table S10). NBO also provides considerable occupancies for lone vacancies (LV), reflecting the formally “unfilled” valence non‐bonding orbitals (LUMOs), of 0.50 to 0.55 e. Notably, LP and LV occupancies together resemble the ELI‐D populations of the P/As (LP) basins quite well, reflecting similarities and differences between orbital‐based and real‐space approaches. LP and LV NBOs both stem 100 % from P/As natural atomic orbitals (NAO), but are composed of different types: whereas LVs have pure p‐character, LPs have 71–78 % s‐character. In the heavier congener, **7**, the As(LP) NBO is slightly stabilized compared to **6** or **8**, whereas the As−C bonding orbitals are slightly destabilized compared to the P‐C NBOs, making As(LP) and As‐C almost degenerate, (Table S10). Comparably, the HOMO–LUMO band‐gap is 257.3 kJ mol^−1^ for compounds **6** and **8**, but 280.9 kJ mol^−1^ for **7**, and the LP is represented by HOMO−3 in **6** and **8**, but HOMO−4 in **7** (Table S12). The Wiberg bond indexes calculated for the C−E bonds are in all cases close to 1 (0.837–0.989, Table S10) suggesting single bond characters. Natural population analysis (NPA) provides atomic and fragmental charges (Table S11). The pnictogen atom carries the positive charge of circa 1.2 e in all three compounds.

Time dependent density functional theory (TD‐DFT) calculations of **6**–**8** are in good agreement with the experimentally observed UV‐vis spectra. There are two calculated absorption maxima for **6** at 599 and 397 nm, for **7** at 536 and 387 nm and for **8** at 586 and 370 nm (Table S13, Figure S92). The larger absorption maxima are associated with HOMO→LUMO transitions. The HOMOs of **6**–**8** are exclusively situated at the flanking fluorenyl groups. The smaller absorption maxima represent HOMO−9 to HOMO−5→LUMO transitions. The HOMO−9 to HOMO−5 have shared contributions of the fluorenyl groups as well as the phenyl and mesityl groups, which explains the different colour of the phosphorus compounds **6** and **8** (Figures S93–S95).

An alternative method to assess the strength of isolated Lewis acids on the basis of DFT involves the calculation of the fluoride ion affinity (FIA).^[43]^ The FIA values calculated for **6** (638.9 kJ mol^−1^), **7** (638.9 kJ mol^−1^) and **8** (630.9 kJ mol^−1^) are significantly larger than that of SbF_5_ (480 kJ mol^−1^), which qualifies them as Lewis super acids.

Essentially dicoordinate and donor‐free phosphenium and arsenium ions were prepared and fully characterized. The kinetic stabilization is achieved by judicious employment of a bulky and rigid aryl substituent and a smaller but editable aryl group (mesityl, phenyl). Compounds **6**–**8**, possessing a vacant p‐orbital and an electron lone pair with predominantly s‐character, represent the very first examples of free lighter pnictogenium ions,^[10]^ isolated in the absence of electromeric conjugation. Unlike neutral carbenes, **6**–**8** are Lewis super acids, due to the positive charge that is not compensated by intra‐ or intermolecular donor functionalities.

## Conflict of interest

The authors declare no conflict of interest.

## Supporting information

As a service to our authors and readers, this journal provides supporting information supplied by the authors. Such materials are peer reviewed and may be re‐organized for online delivery, but are not copy‐edited or typeset. Technical support issues arising from supporting information (other than missing files) should be addressed to the authors.

Supporting Information
