# Supplementary material for: Cationic Carbene Analogues: Donor‐Free Phosphenium and Arsenium Ions
Source: Angew Chem Int Ed Engl. 2021 Jul 26;60(35):19133–8. doi: 10.1002/anie.202107975 (PMC8456819; doi:10.1002/anie.202107975)
Supplement: Supplementary file 1 — Supporting Information [file ANIE-60-19133-s001.pdf]

Angewandte Chemie
Eine Zeitschrift der Gesellschaft Deutscher Chemiker

Supporting Information

Cationic Carbene Analogues: Donor-Free Phosphenium and Arsenium Ions

Marian Olaru, Stefan Mebs,* and Jens Beckmann**

anie_202107975_sm_miscellaneous_information.pdf

Contents

Experimental procedures 2
General information 2
Synthesis and characterization of Ar^1LiTHF_2 (**1**) 3
Synthesis and characterization of Ar^1PCl_2 (**2**) 6
Synthesis and characterization of Ar^1AsCl_2 (**3**) 11
Synthesis and characterization of Ar^1MesPCl (**4**) 15
Synthesis and characterization of Ar^1MesAsCl (**5**) 21
Synthesis and characterization of $[Ar^1MesP][B(C_6F_5)_4]$ (**6**) 26
Synthesis and characterization of $[Ar^1MesAs][B(C_6F_5)_4]$ (**7**) 35
Synthesis and characterization of $[Ar^1PhP][B(C_6F_5)_4]$ (**8**) 42
UV-Vis spectra 50
Thermal stability tests 52
Table S1 53
Table S2 53
Characterization of **9** 54
Characterization of **10** 67
Reactivity towards water 80
Characterisation of **11** 80
Characterization of **12** 80
Determination of the Gutmann-Beckett acceptor numbers 80
Table S3 80
X-Ray diffraction studies 81
Table S4 82
Table S5 83
Table S6 84
Table S7 85
Computational methods 88
Table S8 89
Table S9 90
Table S10 91
Table S11 91
Table S12 92
Table S13 97
Additional references 101

Experimental procedures

General information

Unless otherwise stated, all reactions, manipulations, work-up and purifications were performed under inert argon atmosphere using anhydrous solvents. Reagents used in this work including PCl_3, $PhPCl_2$, $AsCl_3$ and dimethyl-5-bromoisophthalate were obtained commercially and were used as received. Ar^1Br Ar^1 = dispiro[fluorene-9,3'-(1',1',7',7'-tetramethyl-s-hydrindacen-4'-yl],[1] MesLi (Mes = 2,4,6-trimethylphenyl),[2] $K[B(C_6F_5)_4]$[3] were prepared following the published procedures. Anhydrous dichloromethane, hexane, tetrahydrofuran and toluene were collected from an SPS800 mBraun solvent purification system and stored over 3 Å molecular sieves. Deuterated solvents were degassed and dried over 3 Å molecular sieves under argon. Other solvents, such as 1,2-$F_2C_6H_4$, were dried directly over 3 Å molecular sieves.

Unless otherwise noted, NMR spectra were recorded at room temperature on Bruker Avance Neo 600 MHz spectrometers. ^1H, $^{13}C\{^1H\}$, ^{11}B, ^{31}P, and ^{19}F NMR spectra are reported on the δ scale (ppm) and are referenced against $SiMe_4$, $BF_3{\cdot}Et_2O$ (15% in $CDCl_3$), H_3PO_4 (85% in water), and $CFCl_3$, respectively. ^1H and $^{13}C\{^1H\}$ chemical shifts are reported relative to the residual peak of the solvent ($CDHCl_2$: 5.32 ppm, for CD_2Cl_2; C_4HD_7O: 1.72 for THF-*d8*) in the ^1H NMR spectra, and to the peak of the deuterated solvent (CD_2Cl_2: 53.84 ppm; THF-*d8*: 67.21 ppm) in the $^{13}C\{^1H\}$ NMR spectra.[5] The assignment of the ^1H and $^{13}C\{^1H\}$ resonance signals was made in accordance with the COSY, HSQC, HMBC and NOESY spectra.

The ESI HRMS spectra were measured on a Bruker Impact II spectrometer. Acetonitrile or dichloromethane/acetonitrile solutions ($c = 1\cdot10^{-5}$ mol·L^{-1}) were injected directly into the spectrometer at a flow rate of 3 μL·min^{-1}. Nitrogen was used both as a drying gas and for nebulization with flow rates of approximately 5 L·min^{-1} and a pressure of 5 psi. Pressure in the mass analyzer region was usually about $1\cdot10^{-5}$ mbar. Spectra were collected for 1 min and averaged. The nozzle-skimmer voltage was adjusted individually for each measurement.

UV-Vis spectra were recorded on a Shimadzu UV-2700 spectrometer.

Synthesis and characterization of Ar^1LiTHF_2 (**1**)[6]

To a pre-cooled (−80 °C) solution of Ar^1Br (4.00 g, 6.74 mmol) in THF (60 mL), *t*-BuLi (10.5 mL, 20 mmol) was added. The reaction mixture was stirred 30 minutes at −80 °C, then the cooling bath was removed and the reaction mixture was allowed to reach room temperature where it was kept for 30 minutes. After cooling to 0 °C, the solid was isolated by cannula filtration and washed with THF (2×5mL). The product was dried at reduced pressure to obtain **1** (3.166 g, 70%) as a white solid that was stored in the glovebox. Compound **1** is poorly soluble in THF (*ca.* 4 mg/mL or less), benzene or toluene and insoluble in hexane. Because of the poor solubility of **1** compared to that of highly soluble Ar^1H, the amount of Ar^1H observed by NMR can be deceivably high. Ar^1H formed in very small amounts due to the fact that molecular sieves cannot remove completely the water from THF-*d8*.[7] Crystals were obtained by heating a suspension of **1** in THF.

^{1}H NMR (600 MHz, THF-*d8*): δ = 7.47 (dd, $^3J(^1H–^1H)$ = 7 Hz, $^4J(^1H–^1H)$ = 2 Hz, 4H, H14, H21), 7.13 (dd, $^3J(^1H–^1H)$ = 7 Hz, $^4J(^1H–^1H)$ = 2 Hz, 4H, H11, H18), 7.01 (m, 8H, H12, H13, H19, H20), 6.75 (s, 1H, H4), 2.28 (s, 4H, H6a, H6b), 1.52 (s, 12H, H8, H9). **^{13}C{^{1}H} NMR (151 MHz, THF-*d8*)**: δ = 160.41 (s, C10, C17), 157.79 (s, C2), 146.93 (s, C3), 141.10 (s, C15, C16), 127.88 (s, C12, C19), 126.55 (s, C11, C18), 126.16 (s, C13, C20), 119.46 (s, C14, C21), 111.04 (s, C4), 68.35 (s, C5), 57.84 (s, C6), 43.02 (s, C7), 34.27 (s, C8, C9), C1 was not observed.

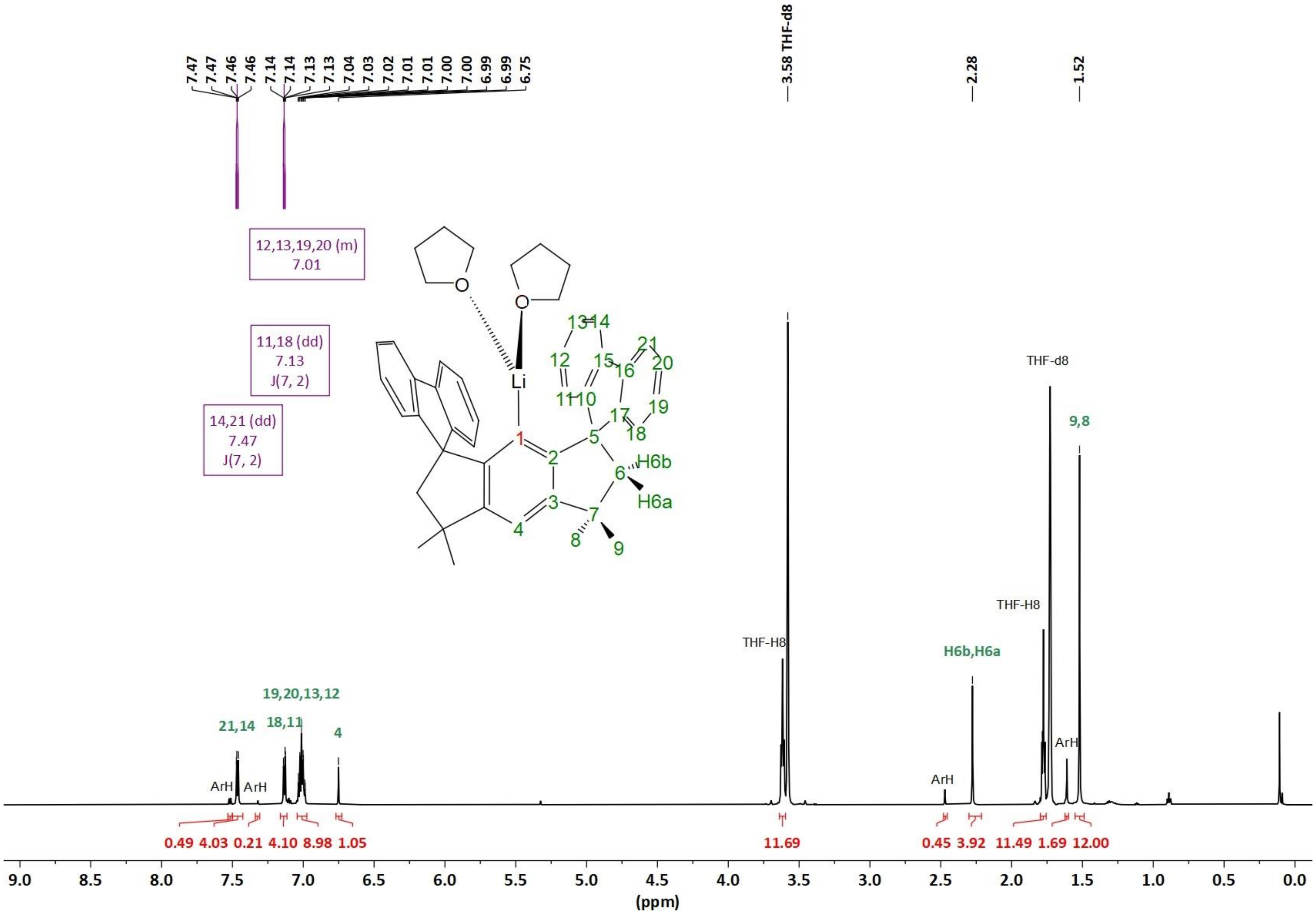

Figure S1.
^{1}H NMR (THF-*d8*, 600 MHz) spectrum of **1**.

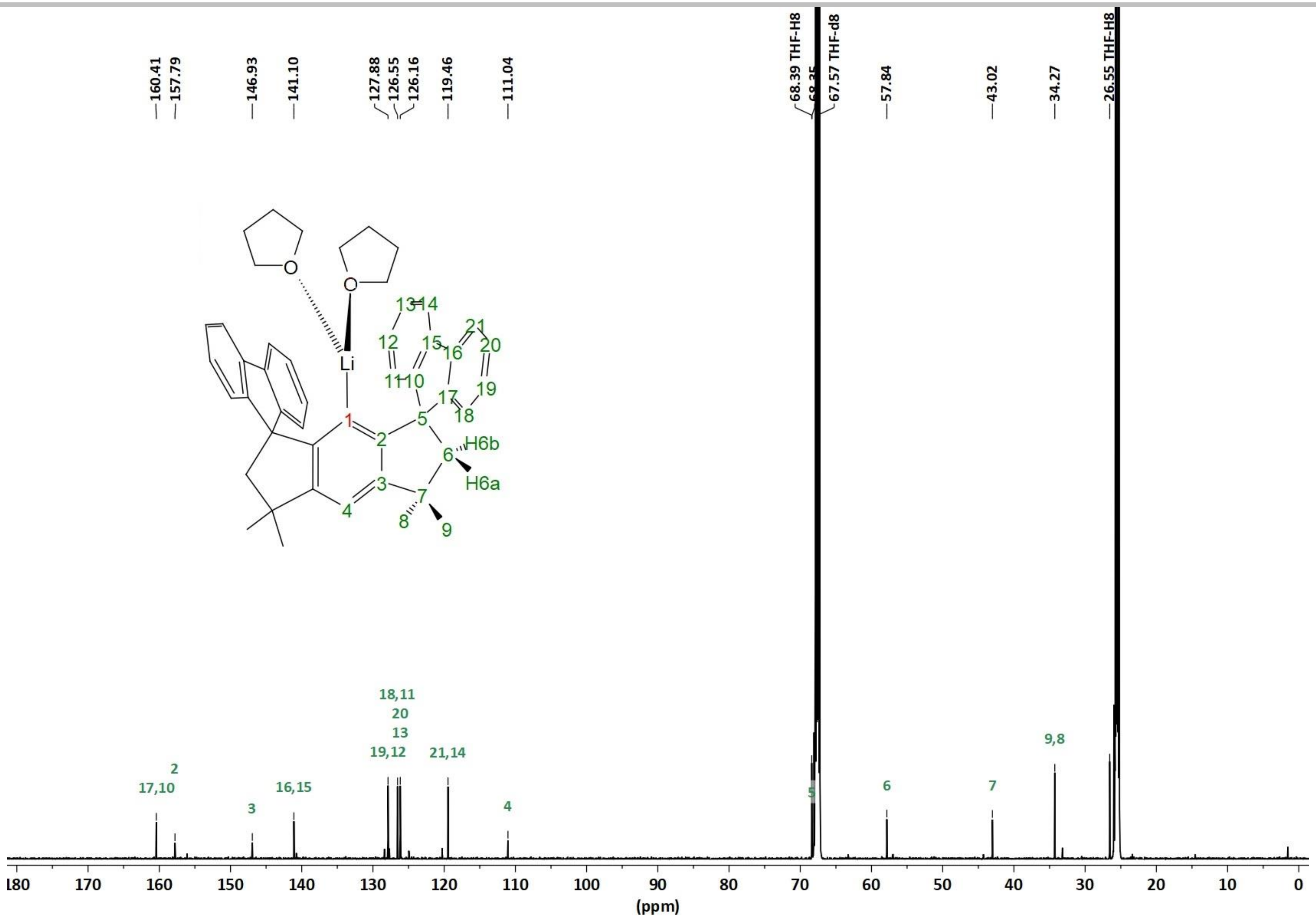

Figure S2.
$^{13}C\{^1H\}$ NMR (THF-*d8*, 151 MHz) spectrum of **1**.

Synthesis and characterization of Ar^1PCl_2 (**2**)

To a suspension of **1** (0.665 g, 1.00 mmol) in toluene (6 mL) was added PCl_3 (0.1 mL, 0.157 g, 1.15 mmol) at room temperature. The reaction mixture was stirred for 15 minutes then hexane was added (20 mL) and the solid decanted, washed with hexane (10 mL), then dried. Dichloromethane (25 mL) was added and the turbid solution was filtered to remove LiCl. After evaporation of the solvent the solid product was recrystallized from the minimum amount of boiling toluene (ca. 18 mL) to obtain **2** (0.283, 46%) as a white solid. An additional crop of product (64 mg, total yield 57%) was obtained by layering hexane (25 mL) over the toluene mother liquor.

Mp. >360 °C. **^{1}H NMR (600 MHz, CD_2Cl_2)**: δ = 7.63 (d, $^3J(^1H–^1H)$ = 7 Hz, 4H, H14, H21), 7.56 (s, 1H, H4), 7.25 (m, br, 4H, H13, H20), 7.16 (m, 8H, H11, H12, H18, H19), 2.44 (s, 4H, H6a, H6b), 1.58 (s, 12H, H8, H9). **^{13}C{^{1}H} NMR (151 MHz, CD_2Cl_2)**: δ = 158.48 (s, br, C3), 156.09 (s, C17, C10), 148.41 (s, br, C2), 140.32 and 140.29 (s, C15 and C16), 133.89 (d, $^1J(^{13}C–^{31}P)$ = 90 Hz, C1), 127.85 (s, C12, C19), 127.49 (s, C13, C20), 125.22 (s, br, C11, C18), 123.48 (s, C4), 120.55 (s, br, C14, C21), 64.91 (d, $^3J(^{13}C–^{31}P)$ = 12 Hz, C5), 58.87 (s, br, C6), 43.50 (s, C7), 32.81 (s, C8, C9). **^{31}P NMR (243 MHz, CD_2Cl_2)**: δ = 159.29 (s). **HRMS ESI (m/z):** $[M+H]^+$ calculated. for $C_{40}H_{34}Cl_2P$, 615.17697; found, 615.17628.

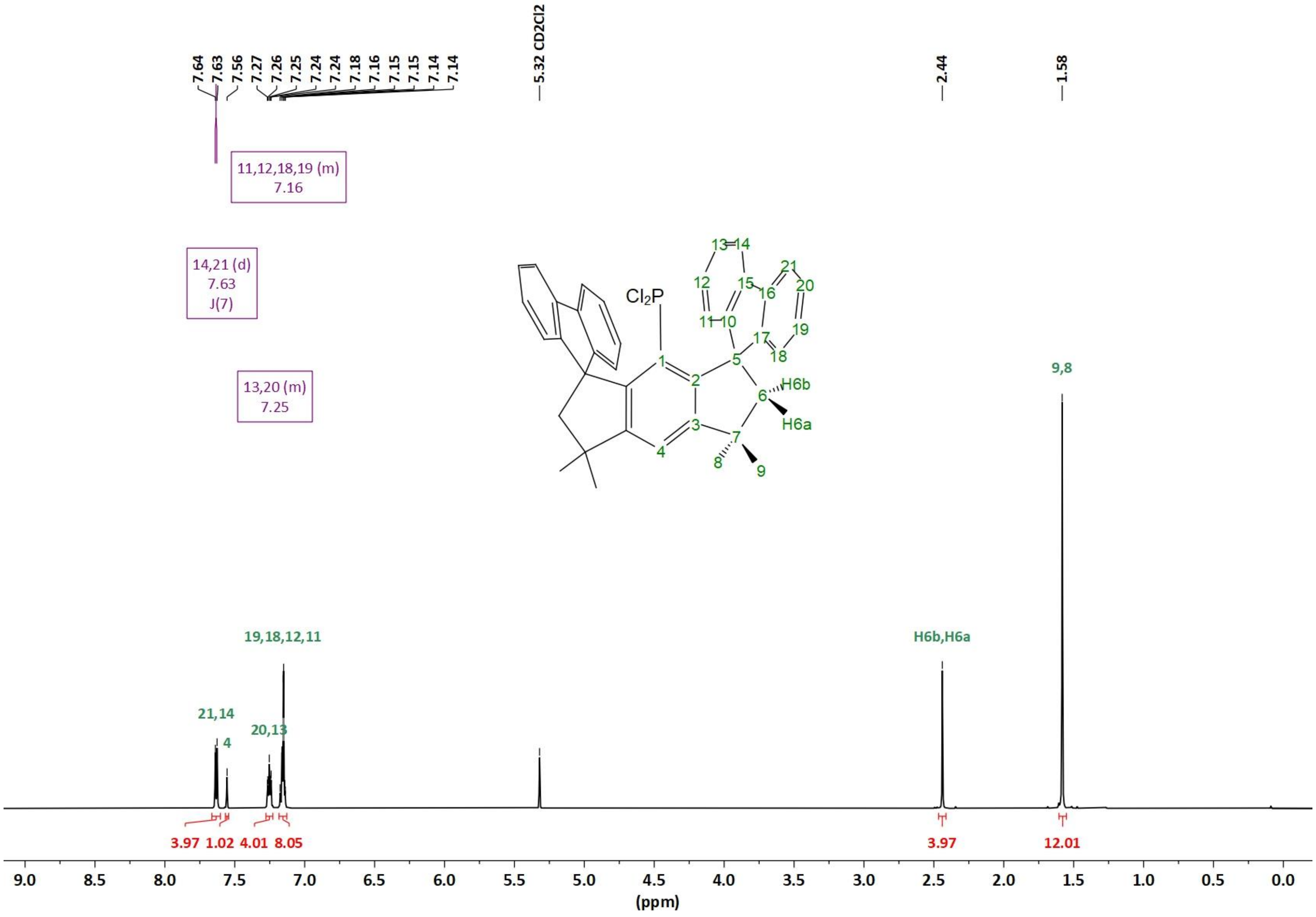

Figure S3.
^{1}H NMR (CD_2Cl_2, 600 MHz) spectrum of **2**.

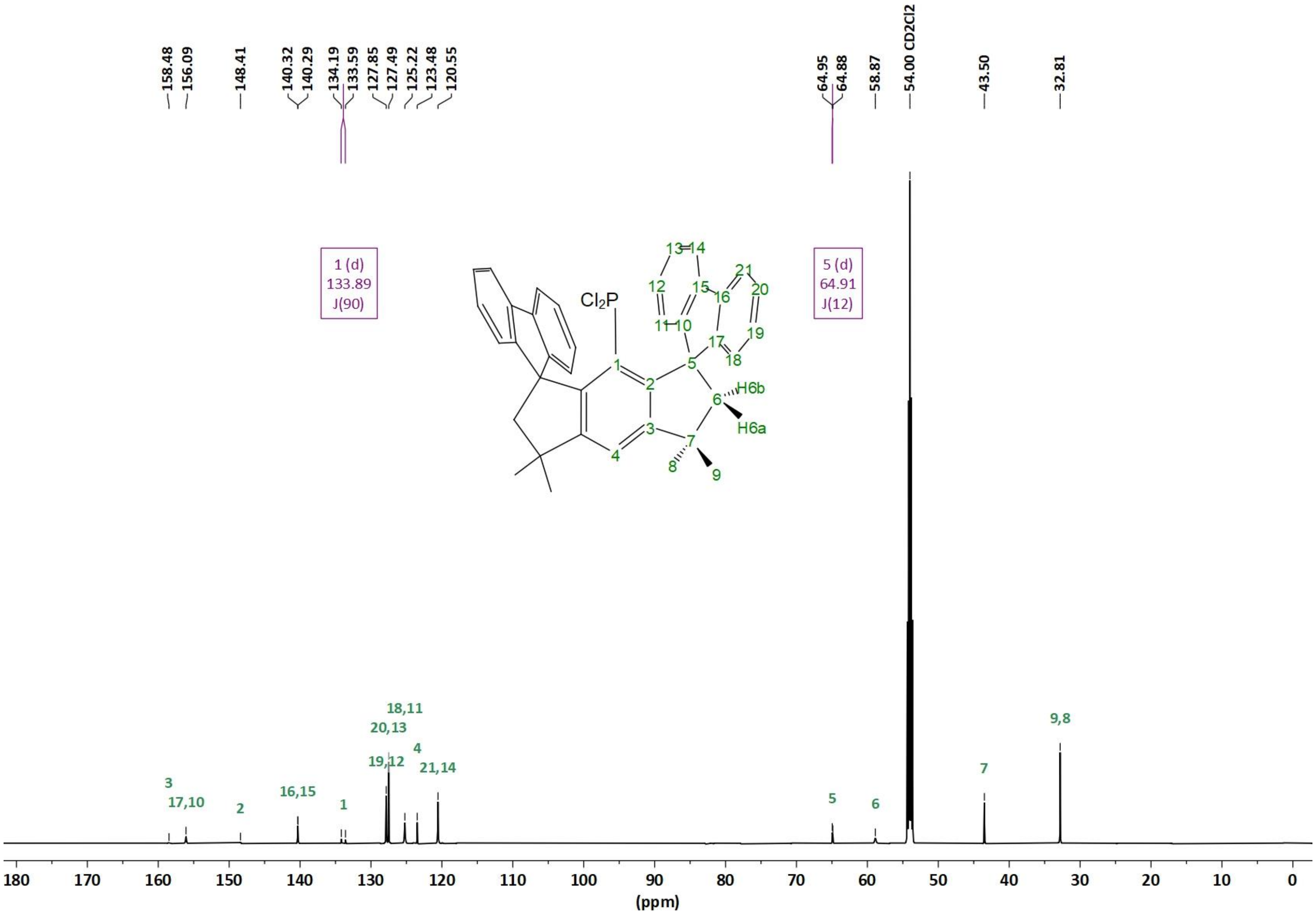

Figure S4.
$^{13}C\{^{1}H\}$ NMR (CD_2Cl_2, 151 MHz) spectrum of **2**.

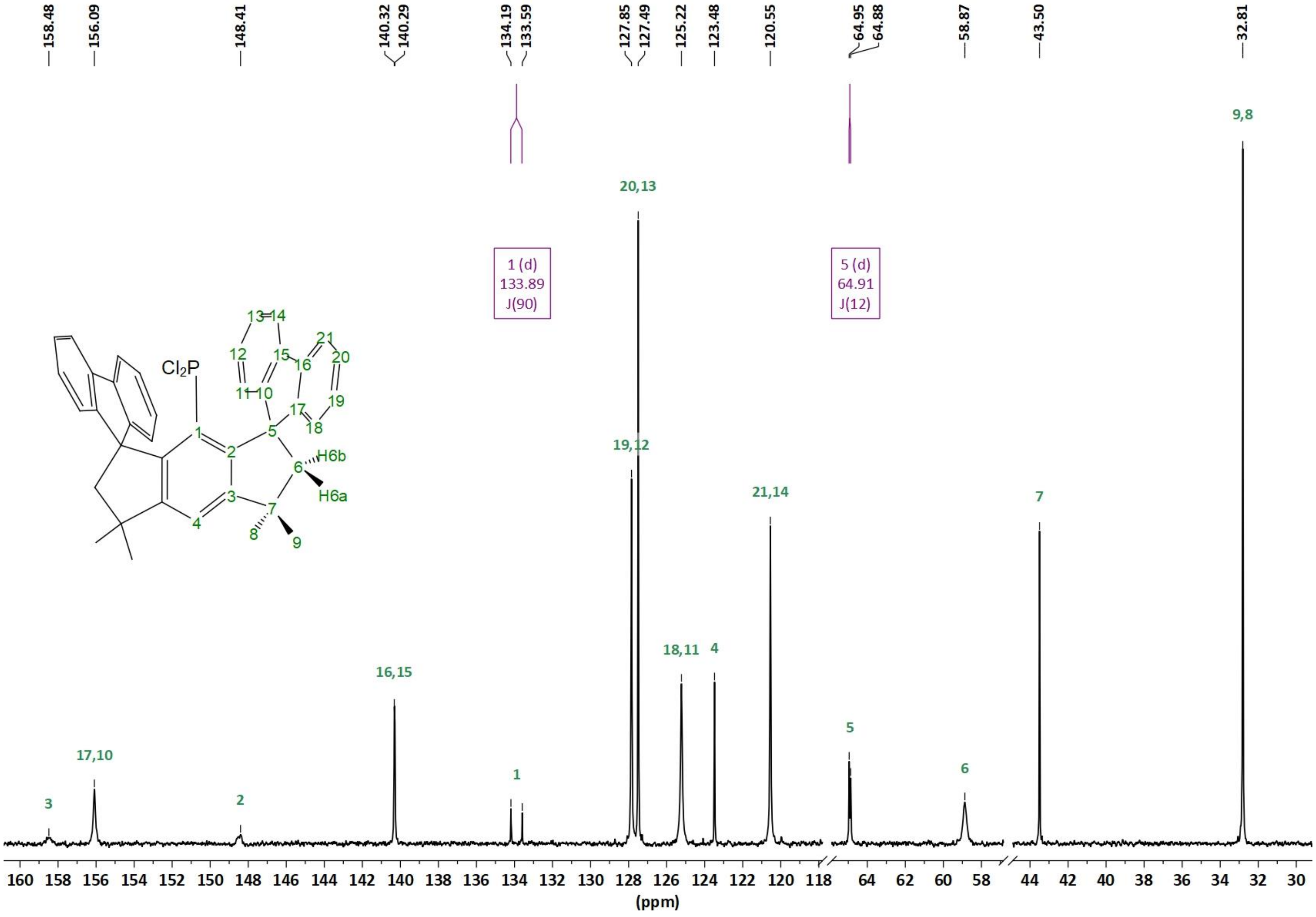

Figure S5.

Detailed $^{13}C\{^1H\}$ NMR (CD_2Cl_2, 151 MHz) spectrum of **2**.

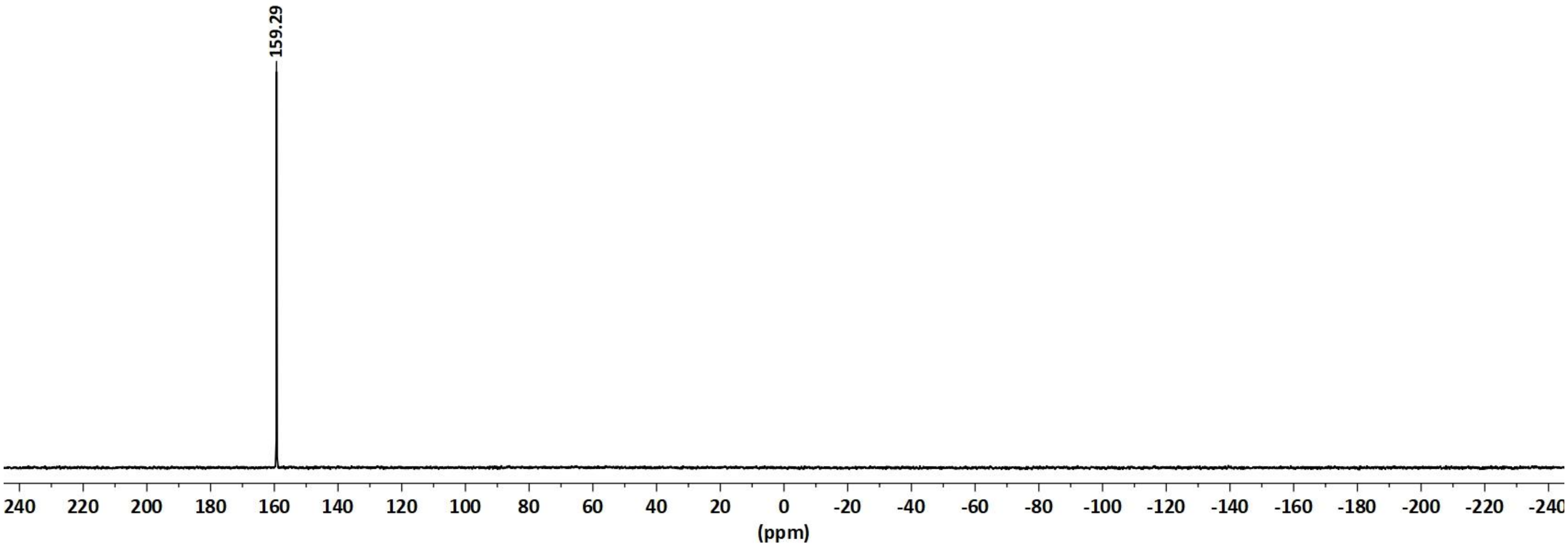

Figure S6.
^{31}P NMR (CD_2Cl_2, 243 MHz) spectrum of **2**.

Synthesis and characterization of Ar^1AsCl_2 (**3**)

To a suspension of **1** (0.665 g, 1.00 mmol) in toluene (6 mL) was added $AsCl_3$ (0.1 mL, 0.216 g, 1.20 mmol) at room temperature. The reaction mixture was stirred for 15 minutes then hexane was added (20 mL) and the solid decanted, washed with hexane (10 mL) then dried. Dichloromethane (28 mL) was added and the turbid solution was filtered to remove LiCl. After evaporation of the solvent the solid product was recrystallized from the minimum amount of boiling toluene (ca.22 mL) to obtain **3** (0.262, 40%) as a white solid. An additional crop of product (0.165 g, total yield 65%) was obtained by layering hexane (25 mL) over the toluene mother liquor.

Mp. 349–351 °C. **^{1}H NMR (600 MHz, CD_2Cl_2)**: δ = 7.63 (d, $^3J(^1H–^1H)$ = 8 Hz, 4H, H14, H21), 7.51 (s, 1H, H4), 7.27 (ddd, $^3J(^1H–^1H)$ = 8 Hz, $^3J(^1H–^1H)$ = 6 Hz, $^4J(^1H–^1H)$ = 2 Hz, 4H, H13, H20), 7.19 (m, 8H, H11, H12, H18, H19), 2.42 (s, 4H, H6a, H6b), 1.58 (s, 12H, H8, H9). **^{13}C{^{1}H} NMR (151 MHz, CD_2Cl_2)**: δ = 158.36 (s, br, C3), 155.91 (s, C17, C10), 148.62 (s, br, C2), 140.35 (s, C1), 140.24 (s, C15, C16), 128.13 (s, C12, C19), 127.87 (s, C13, C20), 125.51 (s, C11, C18), 122.64 (s, C4), 120.80 (s, C14, C21), 65.05 (s, C5), 59.25 (s, C6), 43.84 (s, C7), 32.81 (s, C8, C9). **HRMS ESI (m/z):** $[M-Cl]^+$ calculated. for $C_{40}H_{33}ClAs$, 623.14813; found, 623.14716; $[M+Na]^+$ calculated. for $C_{40}H_{33}Cl_2AsNa$, 681.10675; found, 681.10547.

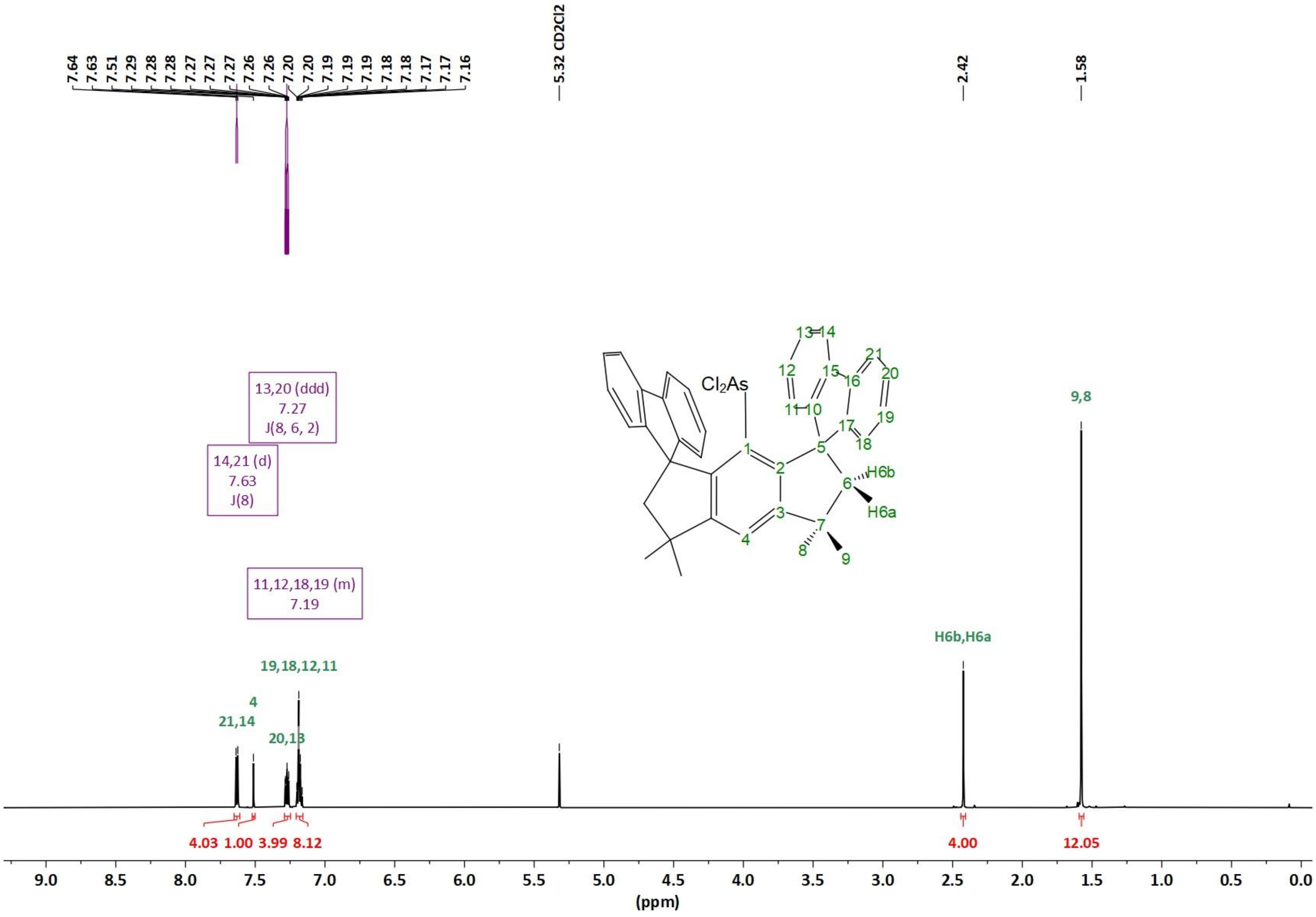

Figure S7.
^{1}H NMR (CD_2Cl_2, 600 MHz) spectrum of **3**.

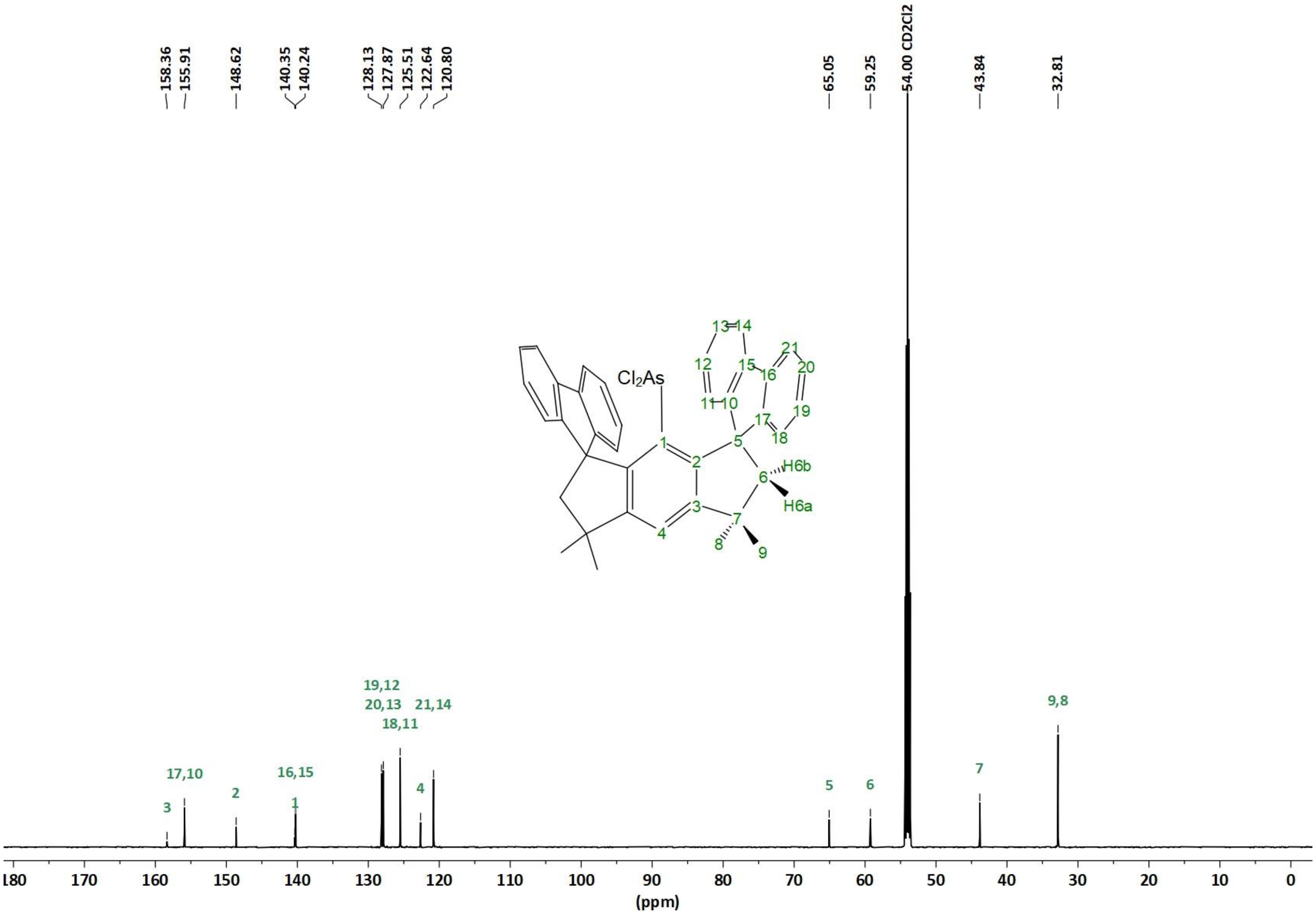

Figure S8.

$^{13}C\{^1H\}$ NMR (CD_2Cl_2, 151 MHz) spectrum of **3**.

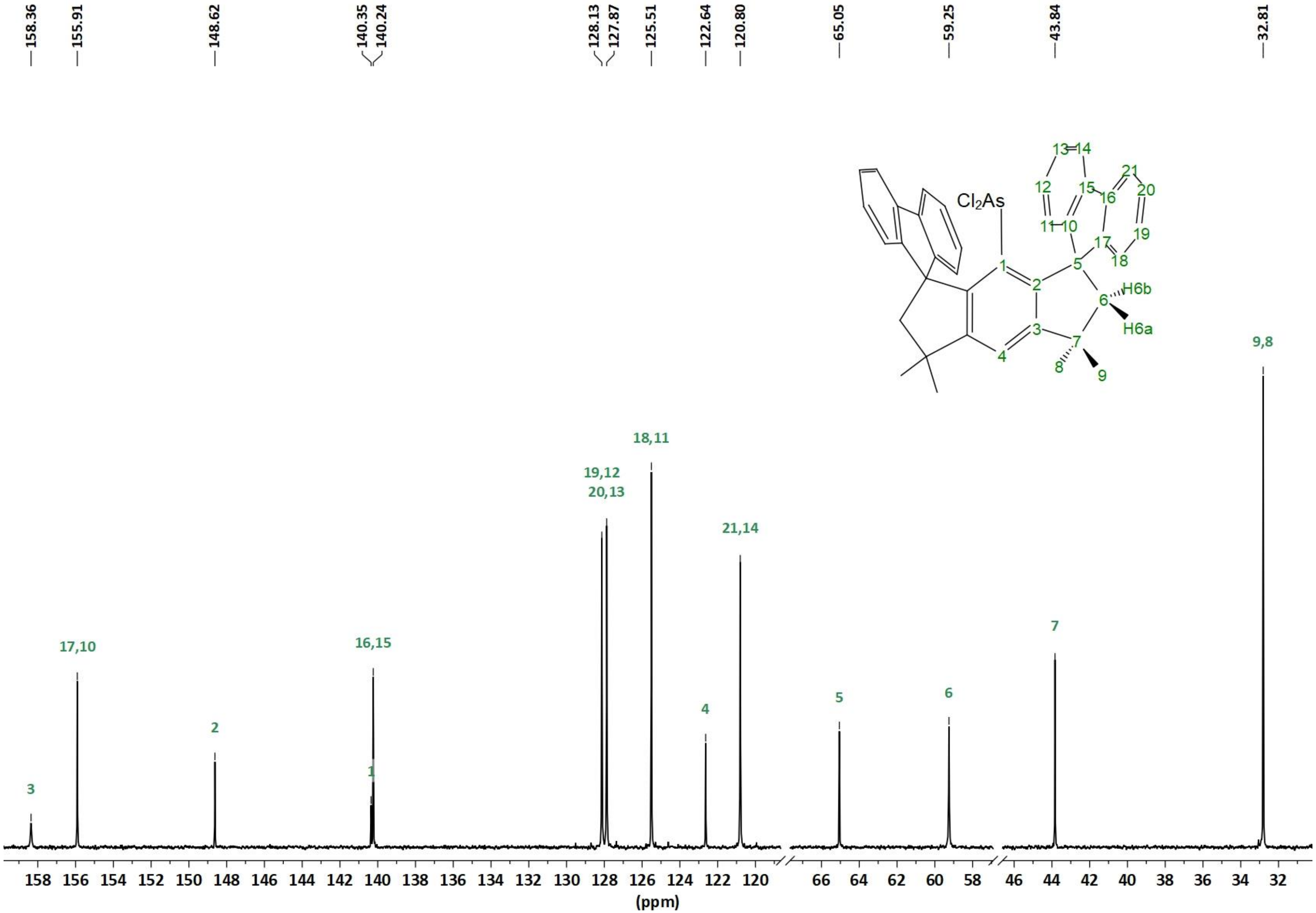

Figure S9.
Detailed $^{13}C\{^{1}H\}$ NMR (CD_2Cl_2, 151 MHz) spectrum of **3**.

Synthesis and characterization of $Ar^1MesPCl$ (**4**)

To a solid mixture of **2** (0.250 g, 0.41 mmol) and MesLi (72 mg, 0.57 mmol) was added toluene (4 mL) and the suspension was stirred at 60 °C. The reaction was monitored by NMR (*ca.* every 12 h), and extra portions of MesLi (55 mg, 25 mg, 40 mg) were added as needed.[1] When full conversion of the starting **2** was observed, the reaction mixture was diluted with toluene (6 mL) and hexane (10 mL) and then filtered under argon to remove unreacted MesLi and LiCl. The solvent from the filtrate solution was removed by rotary evaporation (the product is air stable and can be worked up in air from here on). The resulting product was washed with MeCN (3×3mL) and acetone (3×3mL) then dried thoroughly at 120 °C (10^{-3} mbar) to obtain **4** (0.268 g, 93%) as an off-white solid.

Mp. 267–269 °C. **^{1}H NMR (600 MHz, CD_2Cl_2)**: δ = 7.51 (s, 1H, H4), 7.45 (d, $^3J(^1H–^1H)$ = 7 Hz, 2H, H21), 7.35 (d, $^3J(^1H–^1H)$ = 7 Hz, 2H, H14), 7.22 (d, $^3J(^1H–^1H)$ = 8 Hz, 2H, H18), 7.17 (td, $^3J(^1H–^1H)$ = 7 Hz, $^4J(^1H–^1H)$ = 1 Hz, 2H, H20), 7.13 (td, $^3J(^1H–^1H)$ = 7 Hz, $^4J(^1H–^1H)$ = 1 Hz, 2H, H13), 7.09 (m, 4H, H11, H19), 7.05 (td, $^3J(^1H–^1H)$ = 7 Hz, $^4J(^1H–^1H)$ = 1 Hz, 2H, H12), 5.85 (d, br, $^4J(^1H–^{31}P)$ = 4 Hz, 2H, H24, H26), 2.35 (d, $^2J(^1H–^1H)$ = 14 Hz, 2H, H6a), 2.23 (d, $^2J(^1H–^1H)$ = 14 Hz, 2H, H6b), 1.95 (d, br, $^6J(^1H–^{31}P)$ = 2 Hz, 3H, H30), 1.56 (s, 6H, H9), 1.54 (s, 6H, H8), 1.08 (d, $^4J(^1H–^{31}P)$ = 3 Hz, 6H, H28, H29). **$^{13}C\{^1H\}$ NMR (151 MHz, CD_2Cl_2)**: δ = 158.45 (d, $^3J(^{13}C–^{31}P)$ = 5 Hz, C3), 154. 68 (s, C17), 154.40 (s, C10), 146.18 (d, $^2J(^{13}C–^{31}P)$ = 19 Hz, C2), 140.65 (d, $^5J(^{13}C–^{31}P)$ = 3 Hz, C16), 139.91 (s, C15), 139.79 and 139.77 (s, C23 and C27), 137.54 (d, $^4J(^{13}C–^{31}P)$ = 2 Hz, C25), 137.11 (d, $^1J(^{13}C–^{31}P)$ = 64 Hz, C1), 130.12 (s, C24, C26), 128.90 (d, $^1J(^{13}C–^{31}P)$ = 61 Hz, C22), 127.12 and 127.10 (s, C12, C19, C20), 126.39 (s, C18), 126.30 (s, C13), 124.95 (s, C11), 121.85 (s, C4), 120.35 (s, C21), 120.14 (s, C14), 65.85 (d, $^3J(^{13}C–^{31}P)$ = 7 Hz, C5), 60.47 (s, C6), 42.85 (s, C7), 33.20 (s, C9), 32.81 (s, C8), 23.57 (d, $^3J(^{13}C–^{31}P)$ = 12 Hz, C28, C29), 20.70 (s, C30). **^{31}P NMR (243 MHz, CD_2Cl_2)**: δ = 86.86 (s). **HRMS ESI (m/z):** $[M+H]^+$ calculated. for $C_{49}H_{45}ClP$, 699.29419; found, 699.29237; $[M+Na]^+$ calculated. for $C_{49}H_{44}ClPNa$, 721.27614; found, 721.27444; $[M-Cl]^+$ calculated. for $C_{49}H_{44}P$, 663.31751; found, 663.31618.

[1] For unidentified reasons, the reactions producing ArMesECl (E = P, As) were found to stall after *ca.* 12 hours at various conversion ratios between starting $ArECl_2$ and the product. Prolonged reaction times did not help driving these reactions to completion, neither did increasing the temperature. By adding extra portions of MesLi, the reactions reach completion.

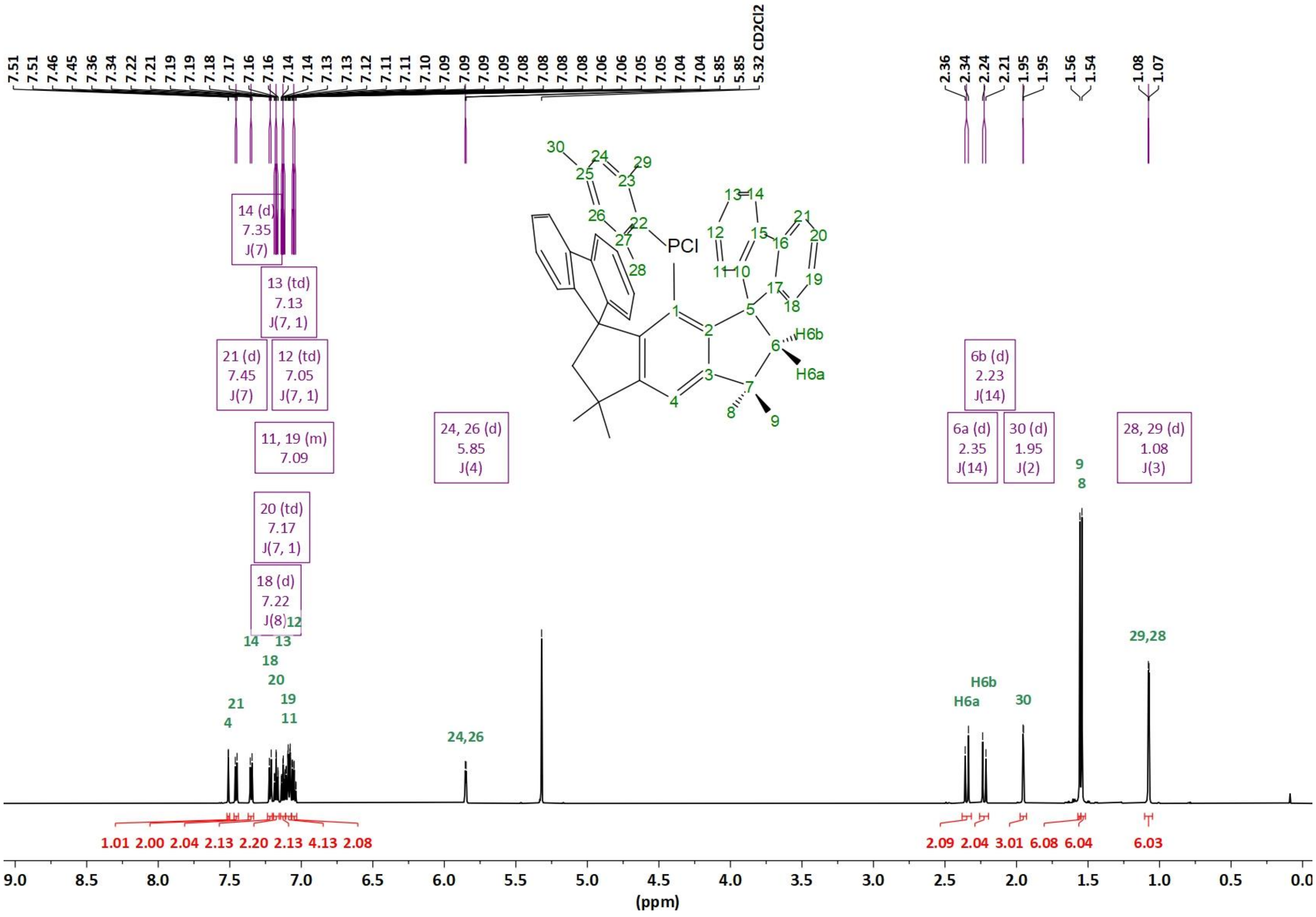

Figure S10.
^{1}H NMR (CD_2Cl_2, 600 MHz) spectrum of **4**.

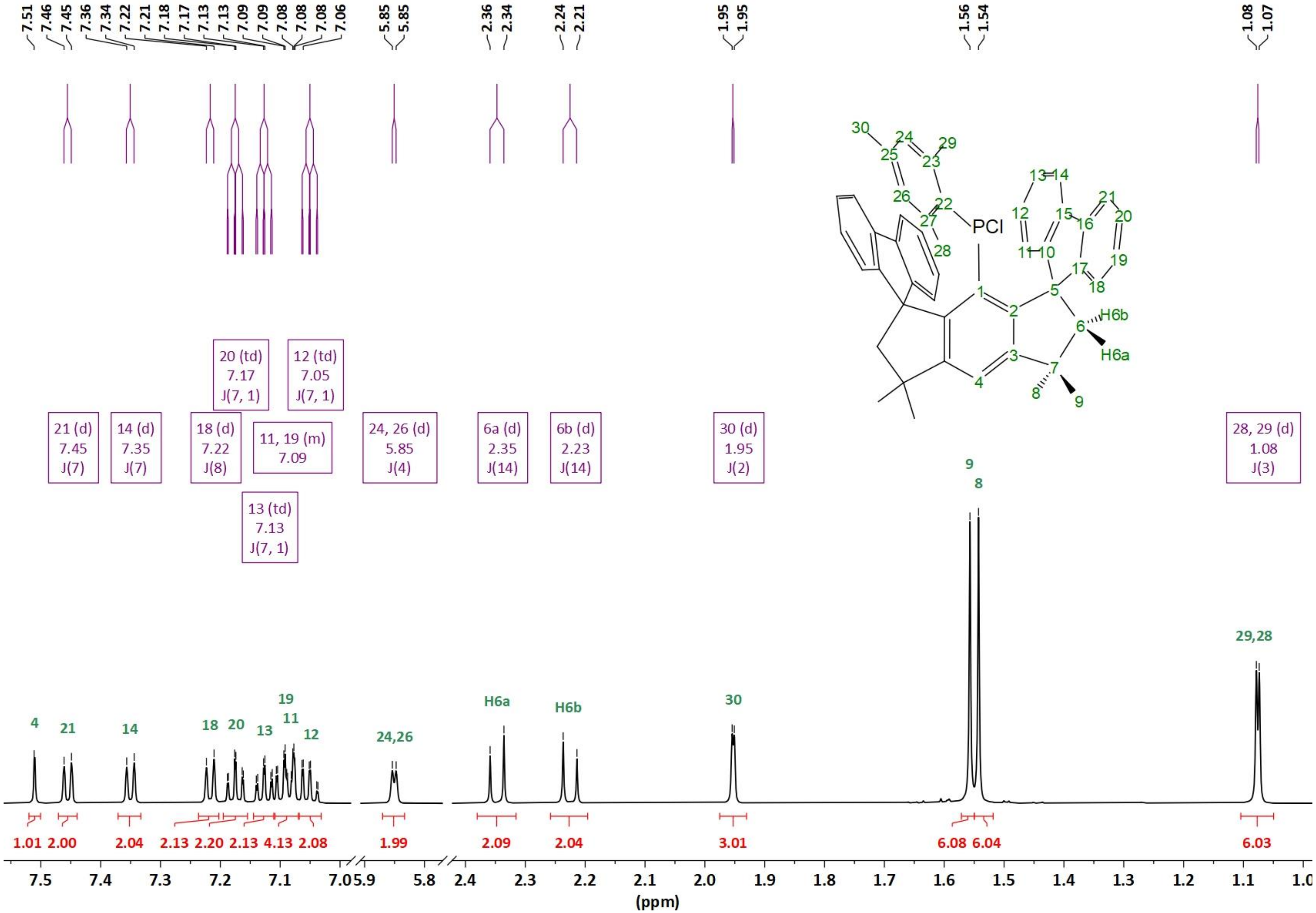

Figure S11.
Detailed ^{1}H NMR (CD_2Cl_2, 600 MHz) spectrum of **4**.

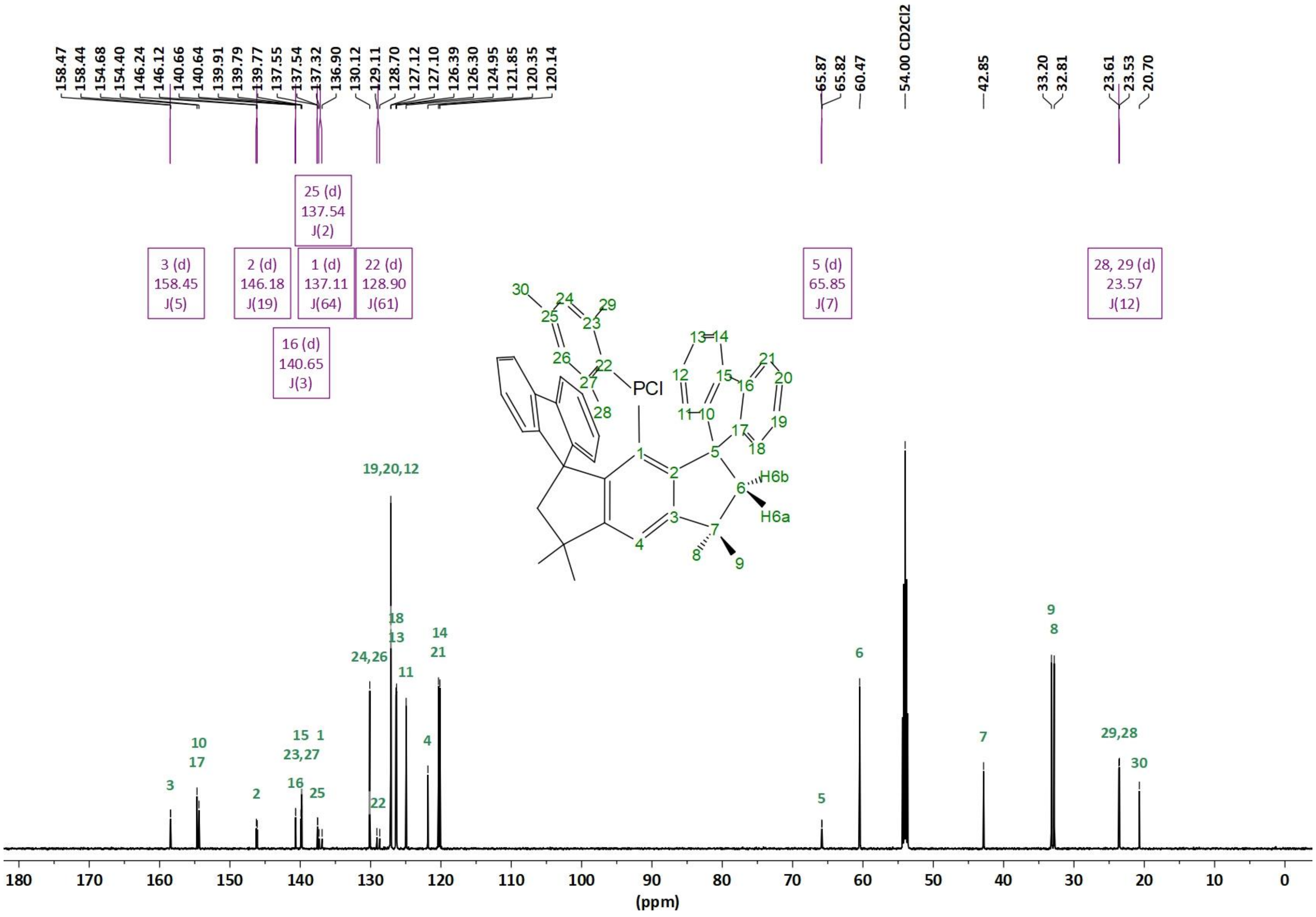

Figure S12.
$^{13}C\{^{1}H\}$ NMR (CD_2Cl_2, 151 MHz) spectrum of **4**.

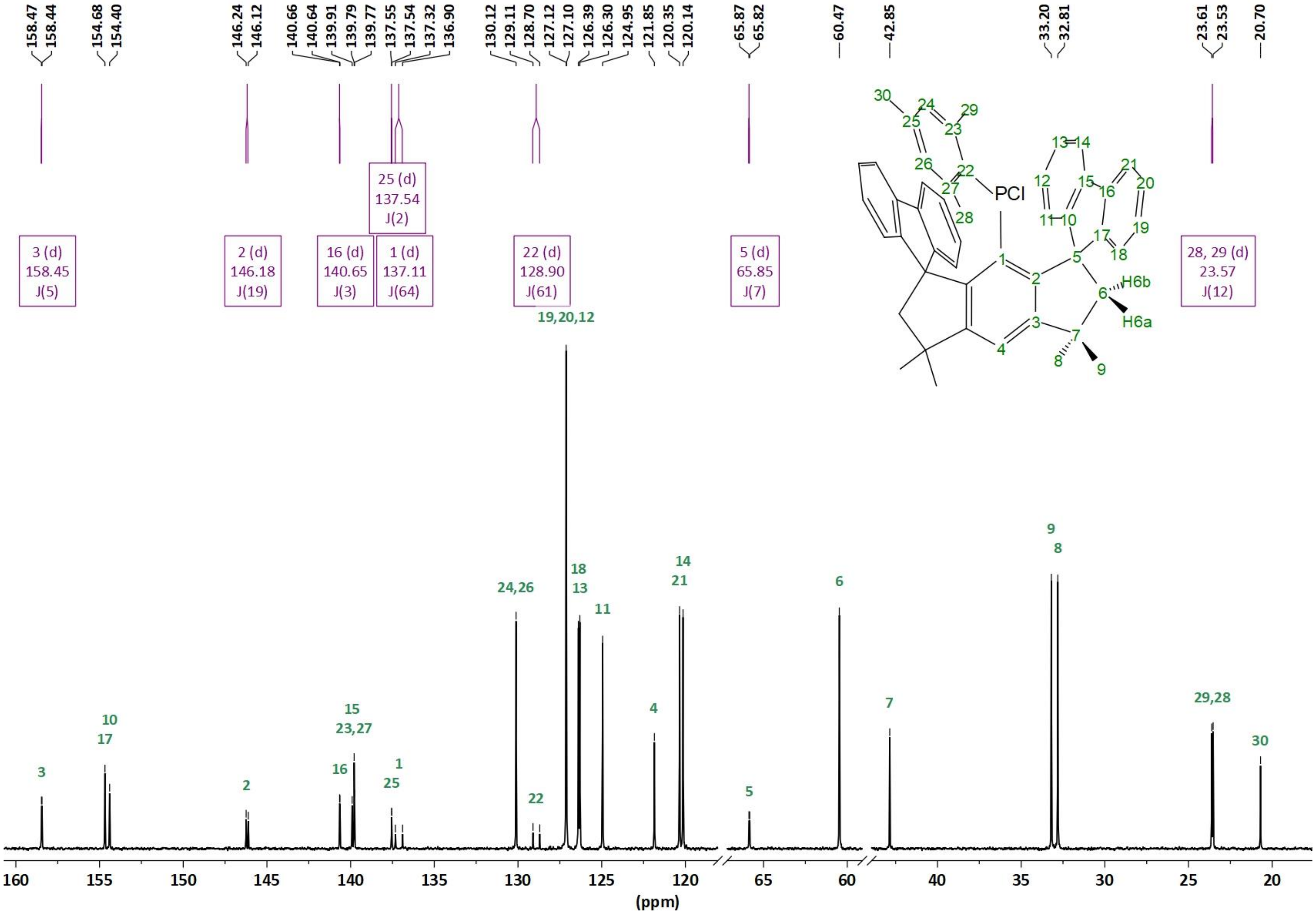

Figure S13.

Detailed $^{13}C\{^{1}H\}$ NMR (CD_2Cl_2, 151 MHz) spectrum of **4**.

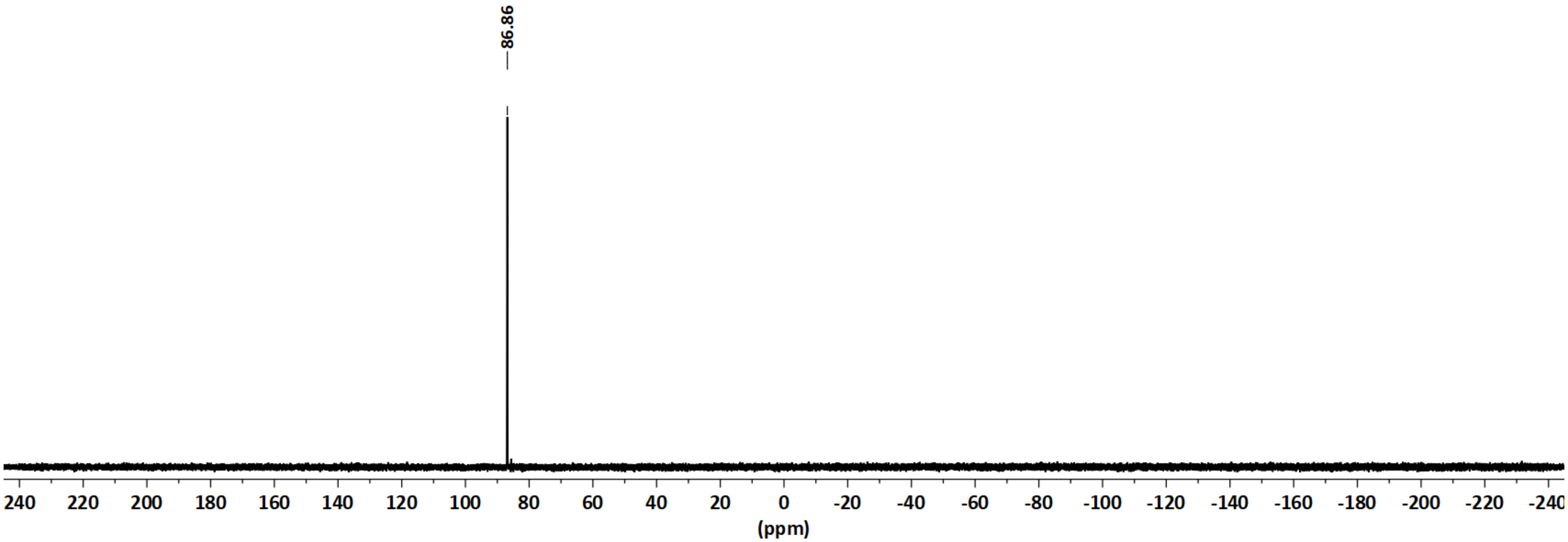

Figure S14.
^{31}P NMR (CD_2Cl_2, 243 MHz) spectrum of **4**.

Synthesis and characterization of Ar^1MesAsCl (**5**)

To a solid mixture of **3** (0.230 g, 0.35 mmol) and MesLi (63 mg, 0.50 mmol) was added toluene (4 mL) and the suspension was stirred at 60 °C. The reaction was monitored by NMR (*ca.* every 12 h), and extra portions of MesLi (45 mg, 50 mg) were added as needed.† When full conversion of the starting **3** was observed, the reaction mixture was diluted with toluene (6 mL) and hexane (15 mL), then filtered under argon to remove unreacted MesLi and LiCl. The solvent from the filtrate solution was removed by rotary evaporation (the product is air stable and can be worked up in air from here on). The resulting product was washed with MeCN (3×3mL) and acetone (3×3mL) then dried thoroughly at 120 °C (10^{-3} mbar) to obtain **5** (0.175 g, 67%) as a white solid.

Mp. 264–266 °C. **^{1}H NMR (600 MHz, CD_2Cl_2)**: δ = 7.48 (s, 1H, H4), 7.42 (d, $^{3}J(^{1}H–^{1}H)$ = 7 Hz, 2H, H21), 7.29 (d, $^{3}J(^{1}H–^{1}H)$ = 8 Hz, 4H, H14, H18), 7.19 (td, $^{3}J(^{1}H–^{1}H)$ = 7 Hz, $^{4}J(^{1}H–^{1}H)$ = 1 Hz, 2H, H20), 7.13 (m, 6H, H11, H13, H19), 7.09 (t, $^{3}J(^{1}H–^{1}H)$ = 8 Hz, 2H, H12), 5.83 (s, br, 2H, H24, H26), 2.37 (d, $^{2}J(^{1}H–^{1}H)$ = 14 Hz, 2H, H6a), 2.17 (d, $^{2}J(^{1}H–^{1}H)$ = 14 Hz, 2H, H6b), 1.97 (s, 3H, H30), 1.55 (s, 6H, H9), 1.54 (s, 6H, H8), 0.98 (s, 6H, H28, H29). **^{13}C{^{1}H} NMR (151 MHz, CD_2Cl_2)**: δ = 158.19 (s, C3), 154.38 (s, C17, C10), 146.26 (s, C2), 141.99 (s, C1), 140.52 and 140.49 (s, C16, C23 and C27), 139.64 (s, C15), 137.48 (s, C25), 134.78 (s, C22), 129.79 (s, C24, C26), 127.36, 127.32, 127.26 (s, C12, C19, C20), 126.81 (s, C18), 126.54 (s, C13), 125.20 (s, C11), 121.30 (s, C4), 120.48 (s, C21), 120.25 (s, C14), 66.77 (s, C6), 43.04 (s, C7), 33.38 (s, C9), 32.67 (s, C8), 22.91 (s, C29, C28), 20.70 (s, C30). **HRMS ESI (m/z):** $[M+Na]^+$ calculated. for $C_{49}H_{44}ClAsNa$, 765.22397; found, 765.22349; $[M-Cl]^+$ calculated. for $C_{49}H_{44}As$, 707.26535; found, 707.26483.

† For unidentified reasons, the reactions producing ArMesECl (E = P, As) were found to stall after *ca.* 12 hours at various conversion ratios between starting $ArECl_2$ and the product. Prolonged reaction times did not help driving these reactions to completion, neither did increasing the temperature. By adding extra portions of MesLi, the reactions reach completion.

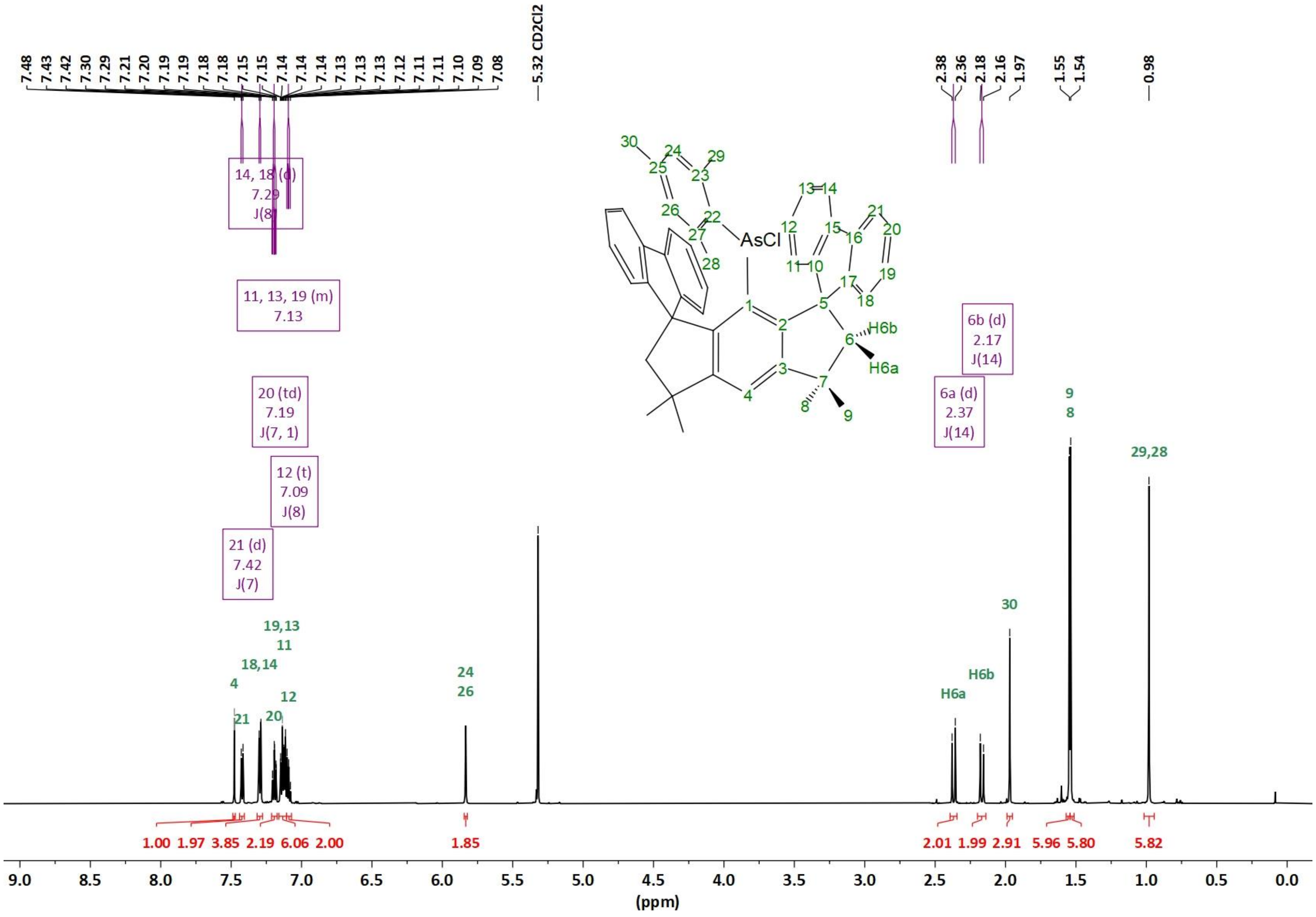

Figure S15.
^{1}H NMR (CD_2Cl_2, 600 MHz) spectrum of **5**.

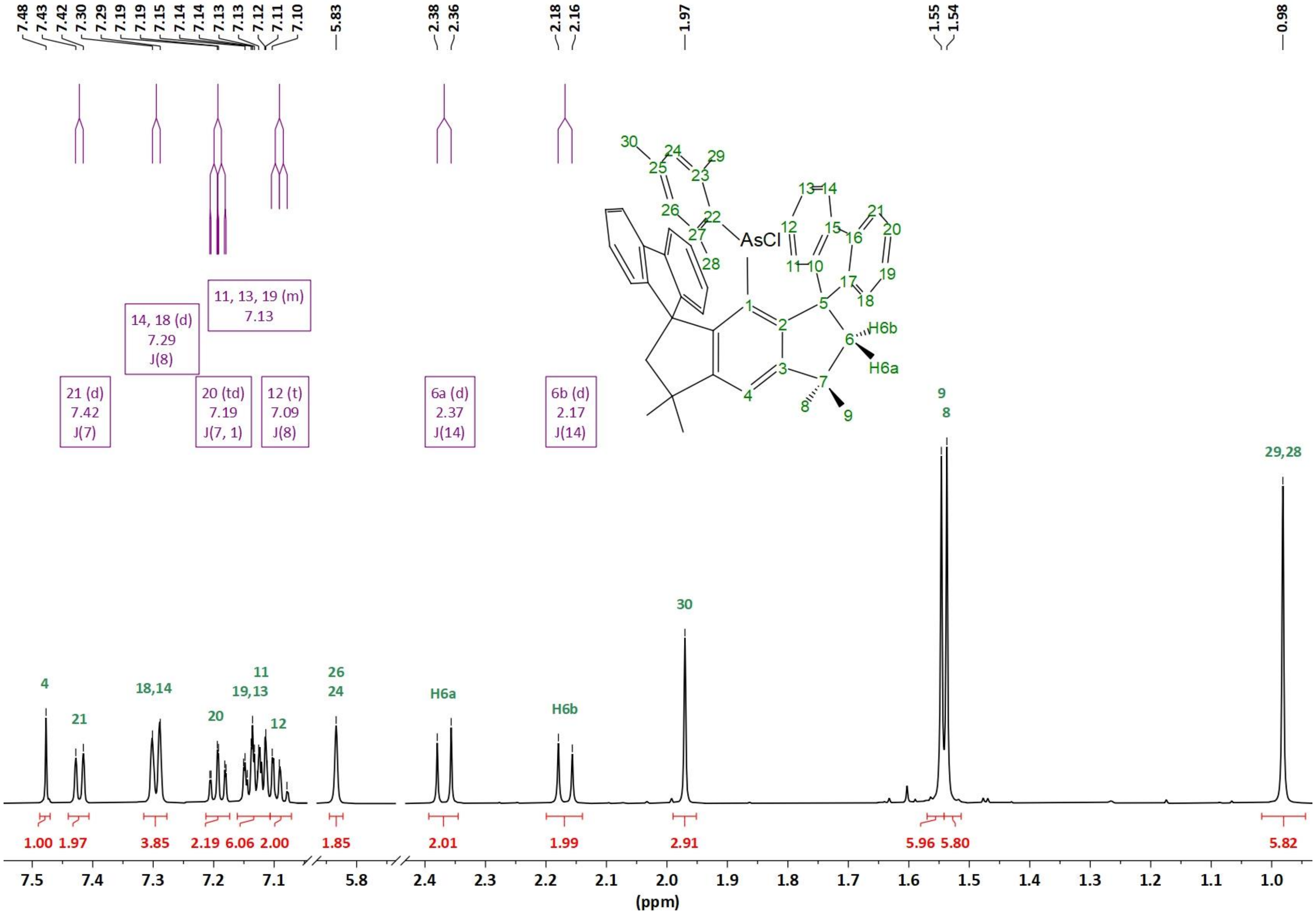

Figure S16.

Detailed ^{1}H NMR (CD_2Cl_2, 600 MHz) spectrum of **5**.

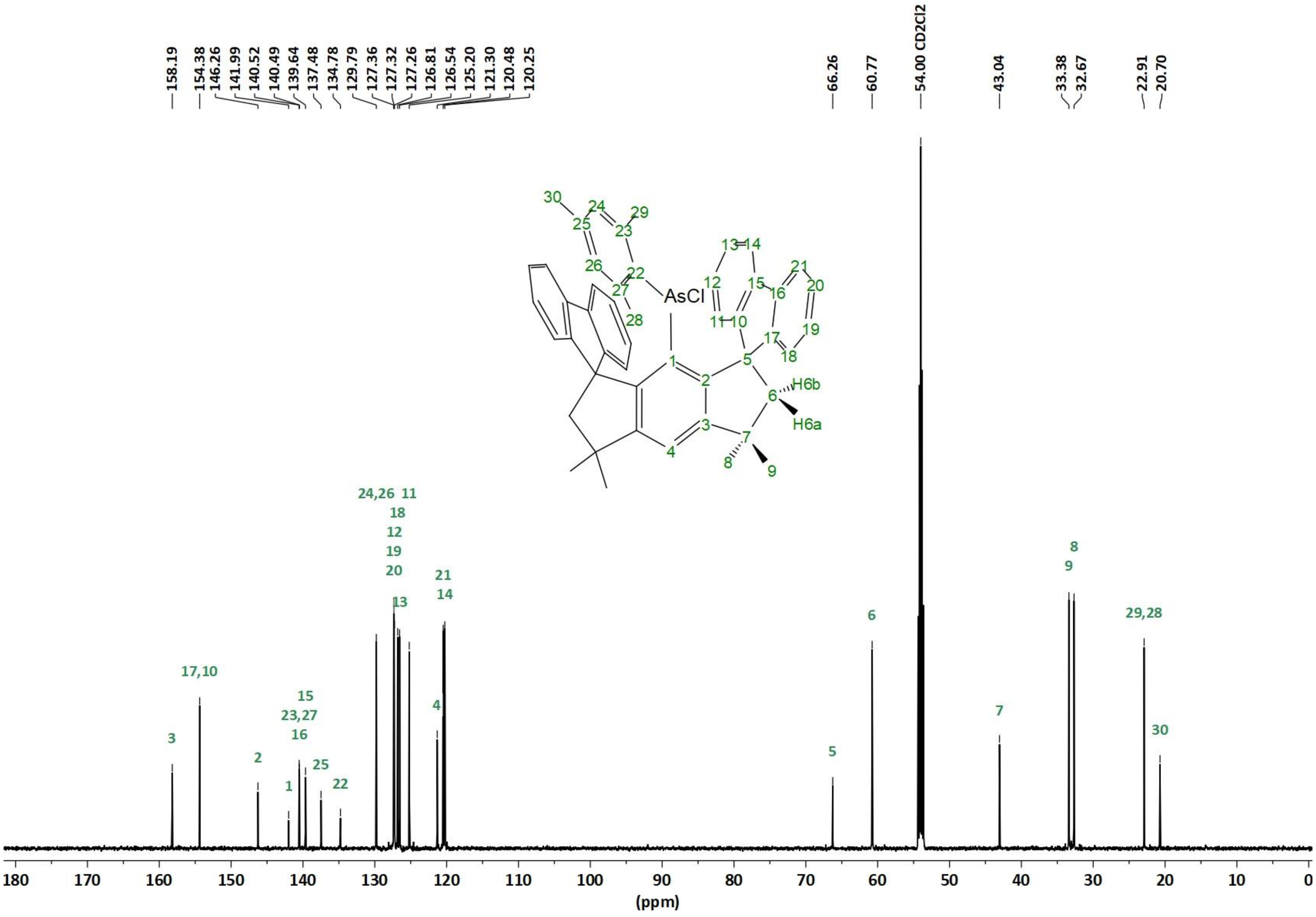

Figure S17.
$^{13}C\{^{1}H\}$ NMR (CD_2Cl_2, 151 MHz) spectrum of **5**.

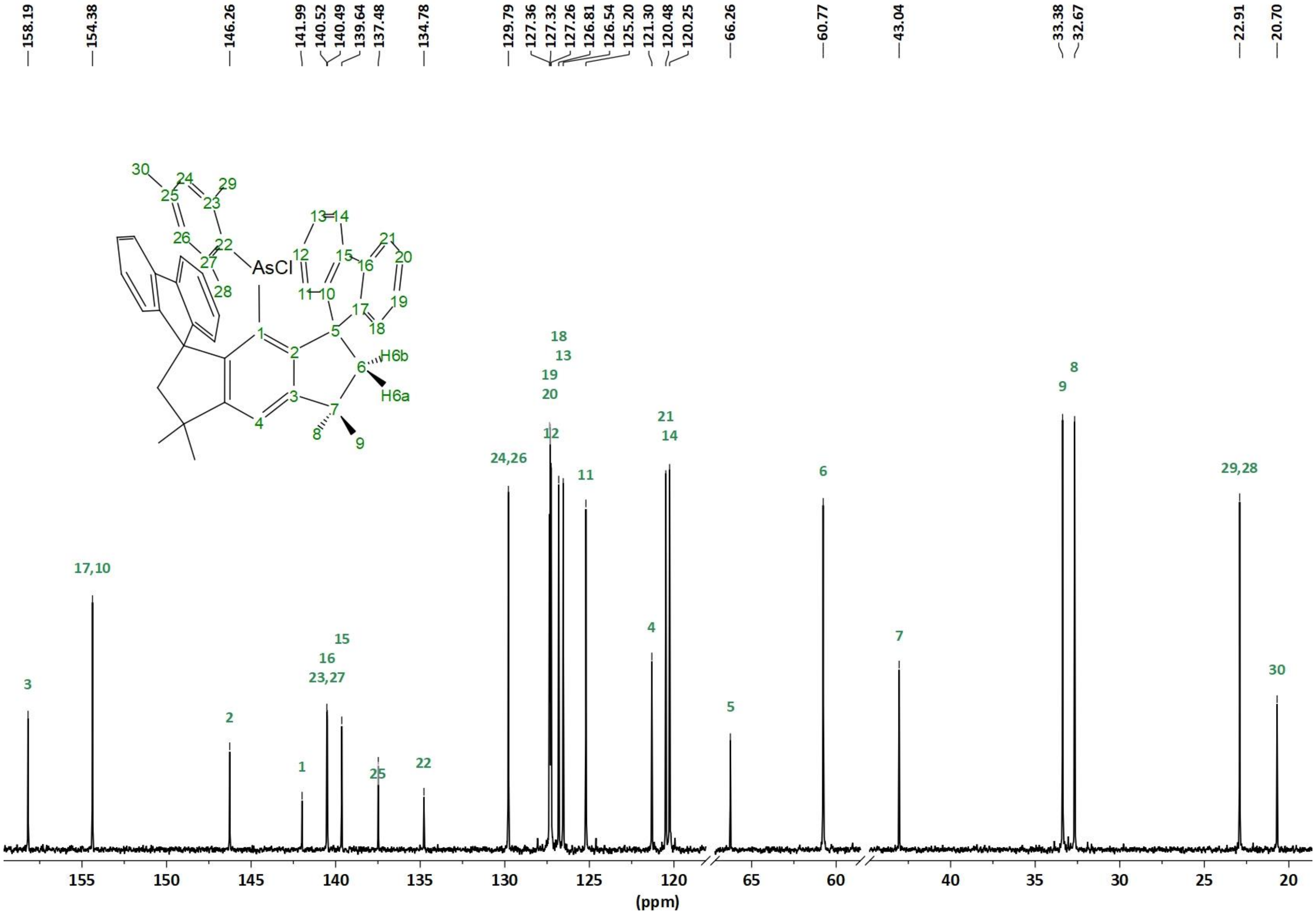

Figure S18.

Detailed $^{13}C\{^{1}H\}$ NMR (CD_2Cl_2, 151 MHz) spectrum of **5**.

Synthesis and characterization of [Ar^{1}MesP][B(C_6F_5)$_4$] (**6**)

To a solid mixture of **4** (1.00 g, 1.43 mmol) and K[B(C_6F_5)$_4$] (1.027 g, 1.43 mmol) was added CH_2Cl_2 (17 mL) and the reaction mixture was stirred at room temperature for at least 30 minutes. The color of the solution changed immediately to dark blue-green. The reaction mixture was filtered under argon through a PTFE syringe filter to remove KCl. Hexane (48 mL) was layered over the CH_2Cl_2 filtrate. The crystalline product was washed with toluene (3×10 mL) and dried at reduced pressure. The title compound was obtained as a dark red (almost black) solid (1.250 g, 65%).

Mp. 220–222 °C. **^{1}H NMR (600 MHz, CD_2Cl_2)**: δ = 7.52 (s, 1H, H4), 7.43 (td, $^3J(^1H–^1H)$ = 8 Hz, $^4J(^1H–^1H)$ = 1 Hz, 2H, H20), 7.39 (d, $^3J(^1H–^1H)$ = 6 Hz, 2H, H21), 7.37 (t, $^3J(^1H–^1H)$ = 7 Hz, 2H, H19), 7.32 (d, $^3J(^1H–^1H)$ = 8 Hz, 2H, H11), 7.27 (d, $^3J(^1H–^1H)$ = 8 Hz, 2H, H18), 7.12 (td, $^3J(^1H–^1H)$ = 7 Hz, $^4J(^1H–^1H)$ = 2 Hz, 2H, H12), 7.00 (d, $^3J(^1H–^1H)$ = 7 Hz, 2H, H14), 6.97 (td, $^3J(^1H–^1H)$ = 7 Hz, $^4J(^1H–^1H)$ = 1 Hz, 2H, H13), 6.81 (s, br, 1H, H24), 6.03 (s, br, 1H, H26), 2.63 (d, $^2J(^1H–^1H)$ = 14 Hz, 2H, H6a), 2.53 (d, $^2J(^1H–^1H)$ = 14 Hz, 2H, H6b), 2.17 (s, 3H, H30), 2.02 (s, br, 3H, H29), 1.76 (s, 6H, H8), 1.64 (s, 6H, H9), 0.70 (s, 3H, H28). **$^{13}C\{^1H\}$ NMR (151 MHz, CD_2Cl_2)**: δ = 159.69 (s, C25), 156.26 (s, C3), 154.66 (d, $^2J(^{13}C–^{31}P)$ = 45 Hz, C23), 153.58 (d, $^1J(^{13}C–^{31}P)$ = 90 Hz, C22), 151.48 (s, C10), 149.08 (s, C27), 148.73 (d, br, $^1J(^{13}C–^{19}F)$ = 240 Hz, C_6F_5), 148.43 (s, C17), 147.20 (s, C2), 143.52 (s, C16), 138.82 (dm, br, $^1J(^{13}C–^{19}F)$ = 245 Hz, C_6F_5), 138.22 (s, C15), 136.88 (d, $^1J(^{13}C–^{19}F)$ = 246 Hz, C_6F_5), 135.60 (d, $^1J(^{13}C–^{31}P)$ = 73 Hz, C1), 133.03 (s, C20), 131.91 (s, C26), 131.43 (s, C24), 131.31 (s, C19), 129.80 (s, C12), 128.91 (s, C13), 128.86 (s, C18), 126.09 (s, C11), 124.60 (s, br, *i*-C_6F_5), 123.08 (s, C4), 123.47 (s, C21), 121.33 (s, C14), 63.02 (s, C5), 53.92 (s, C6), 46.35 (s, C7), 33.06 (s, C9), 32.68 (s, C8), 23.93 (d, $^3J(^{13}C–^{31}P)$ = 37 Hz, C29), 23.29 (s, C30), 22.25 (s, C28). **^{31}P NMR (243 MHz, CD_2Cl_2)**: δ = 573.08 (s). **^{19}F NMR (565 MHz, CD_2Cl_2)**: δ = −133.06 (br, 8F, *o*-C_6F_5), −163.69 (t, $^3J(^{19}F–^{19}F)$ = 20 Hz, 4F, *p*-C6F5), −167.52 (t, br, $^3J(^{19}F–^{19}F)$ = 20 Hz, 8F, *m*-C_6F_5). **^{11}B NMR (193 MHz, CD_2Cl_2)**: δ = −16.64 (s). **HRMS ESI (m/z):** $[M+H_2O]^+$ calculated. for $C_{49}H_{46}PO$, 681.32808; found, 681.32753.

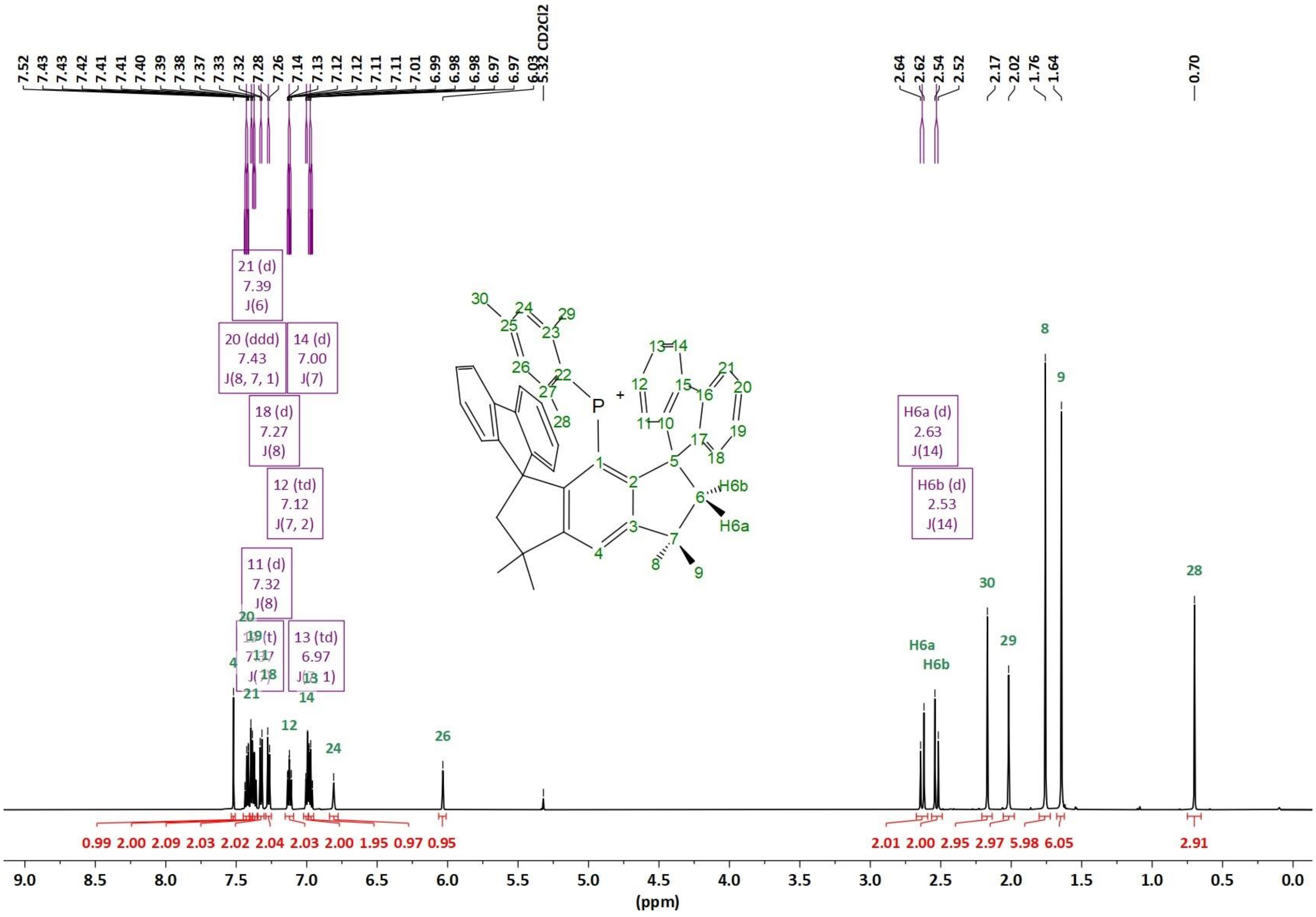

Figure S19.
^{1}H NMR (CD_2Cl_2, 600 MHz) spectrum of **6**.

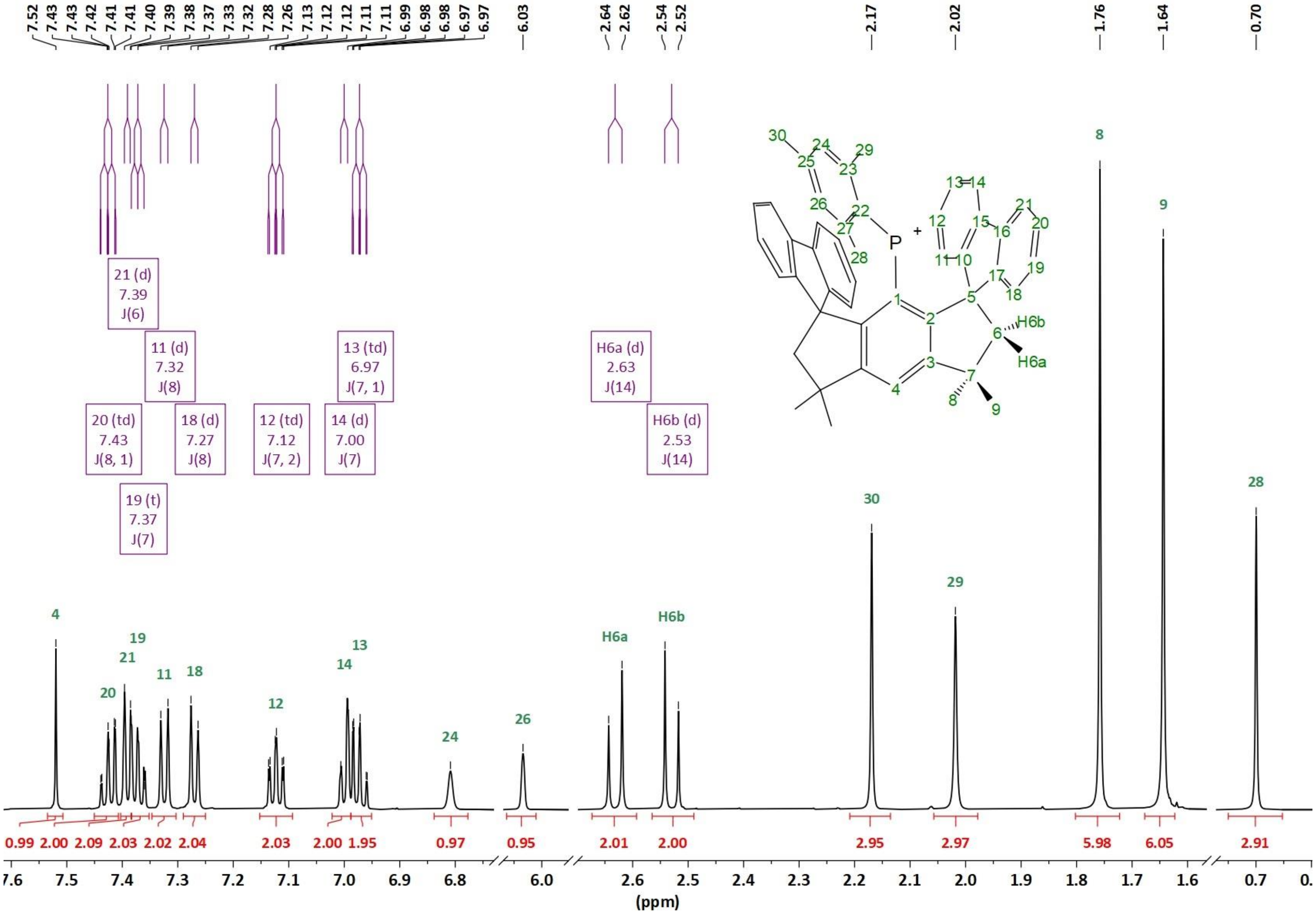

Figure S20.
Detailed ^{1}H NMR (CD_2Cl_2, 600 MHz) spectrum of **6**.

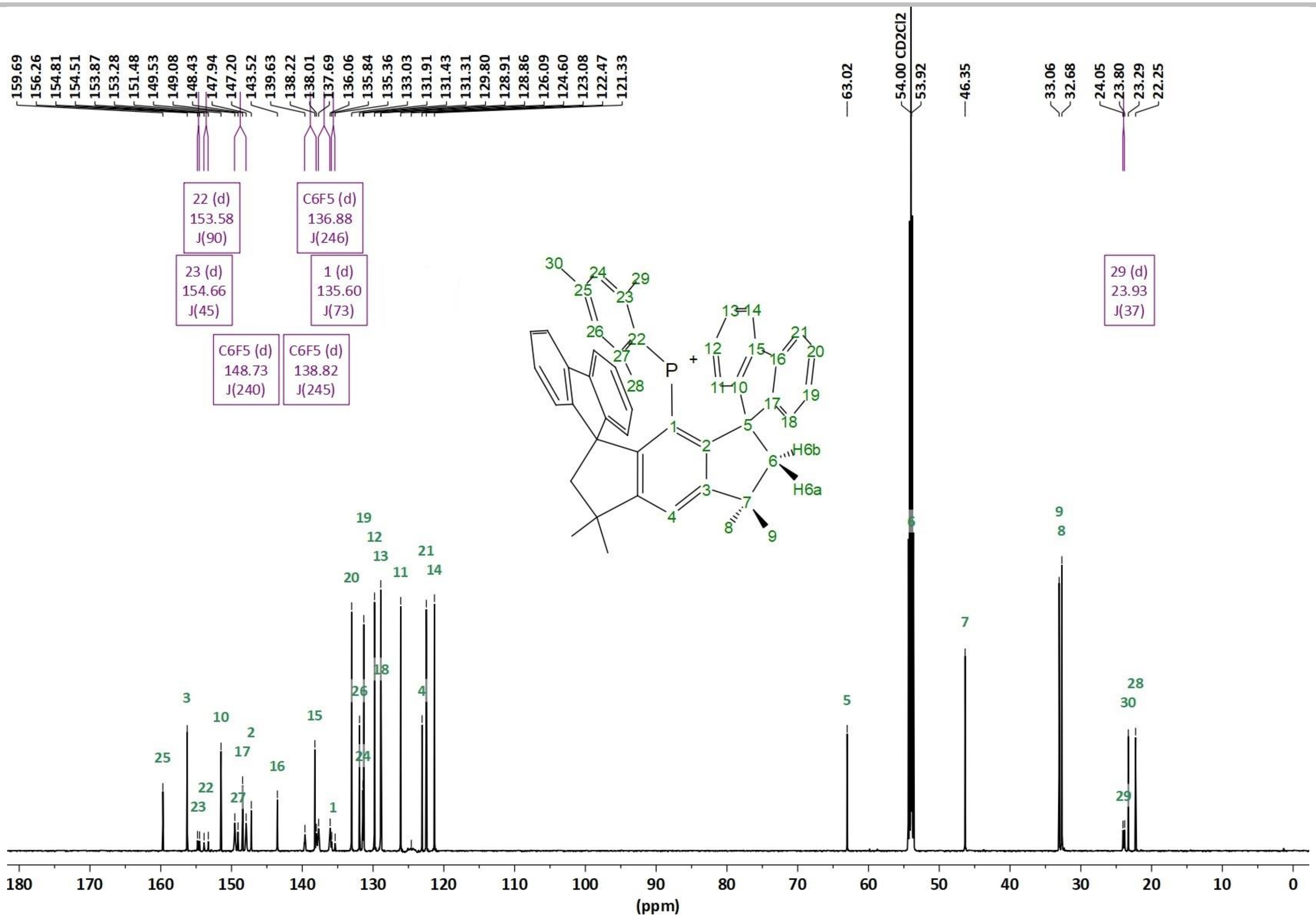

Figure S21.
$^{13}C\{^{1}H\}$ NMR (CD_2Cl_2, 151 MHz) spectrum of **6**.

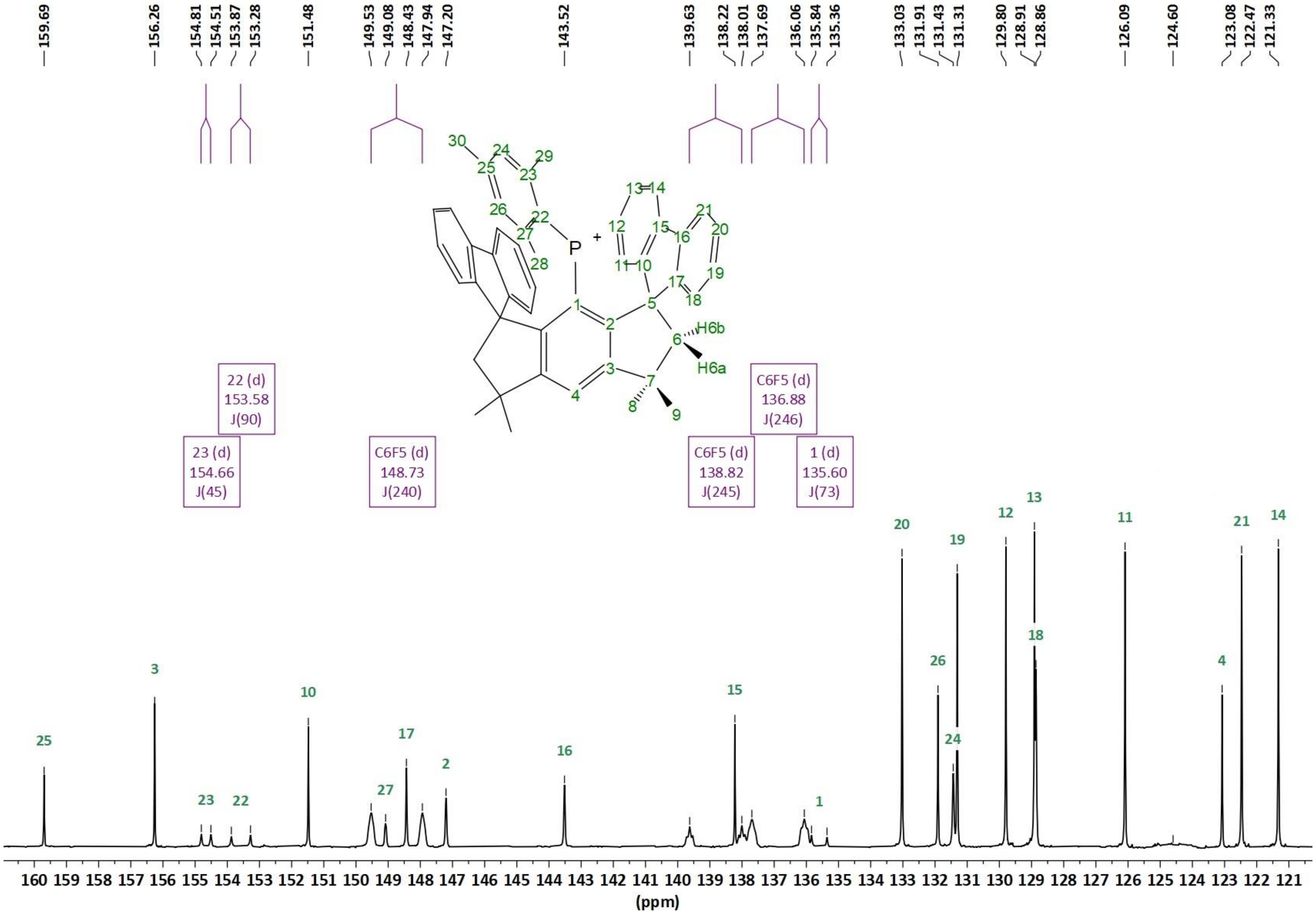

Figure S22.
Detailed $^{13}C\{^{1}H\}$ NMR (CD_2Cl_2, 151 MHz) spectrum (aromatic area) of **6**.

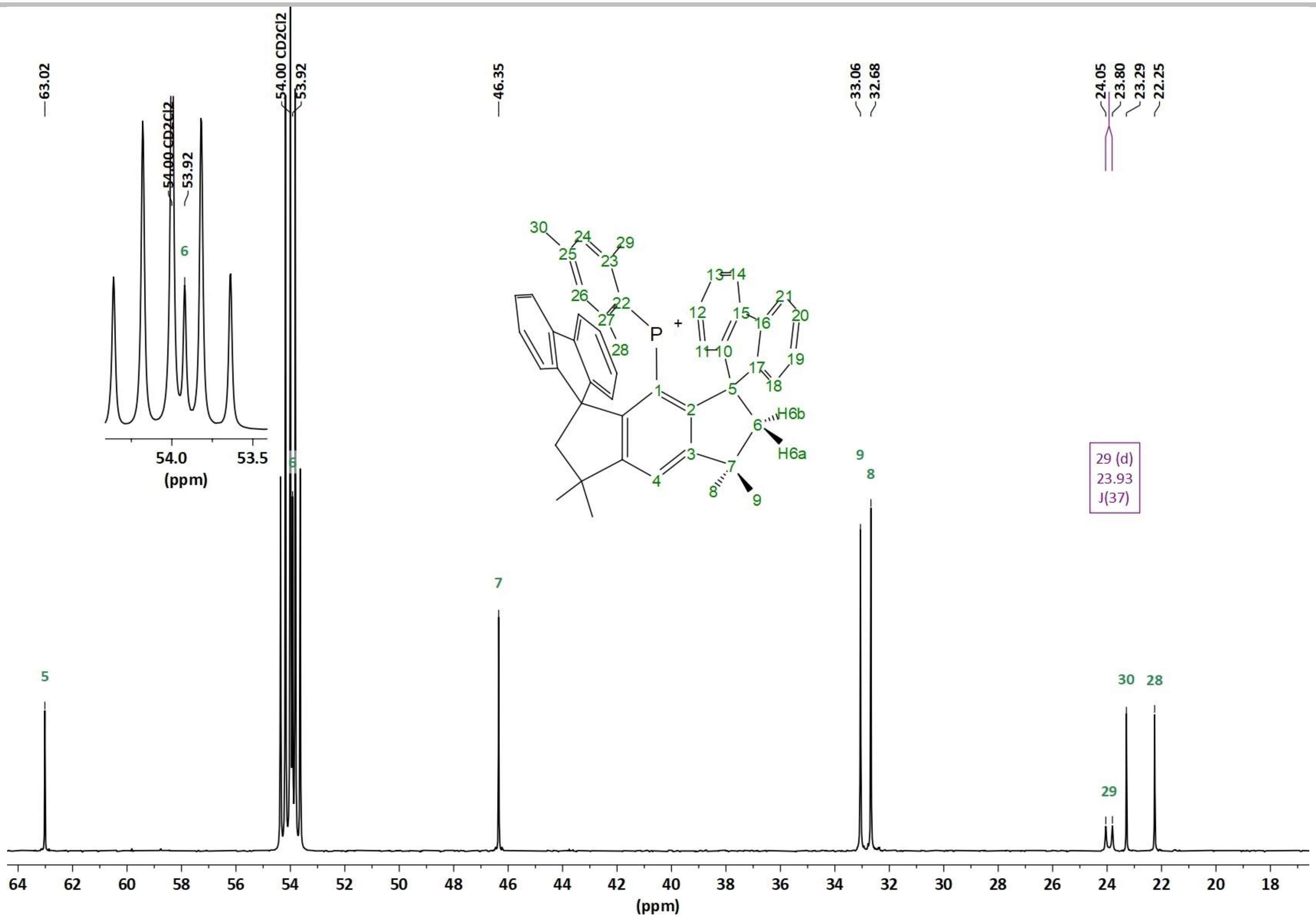

Figure S23.
Detailed $^{13}C\{^1H\}$ NMR (CD_2Cl_2, 151 MHz) spectrum (aliphatic area) of **6**.

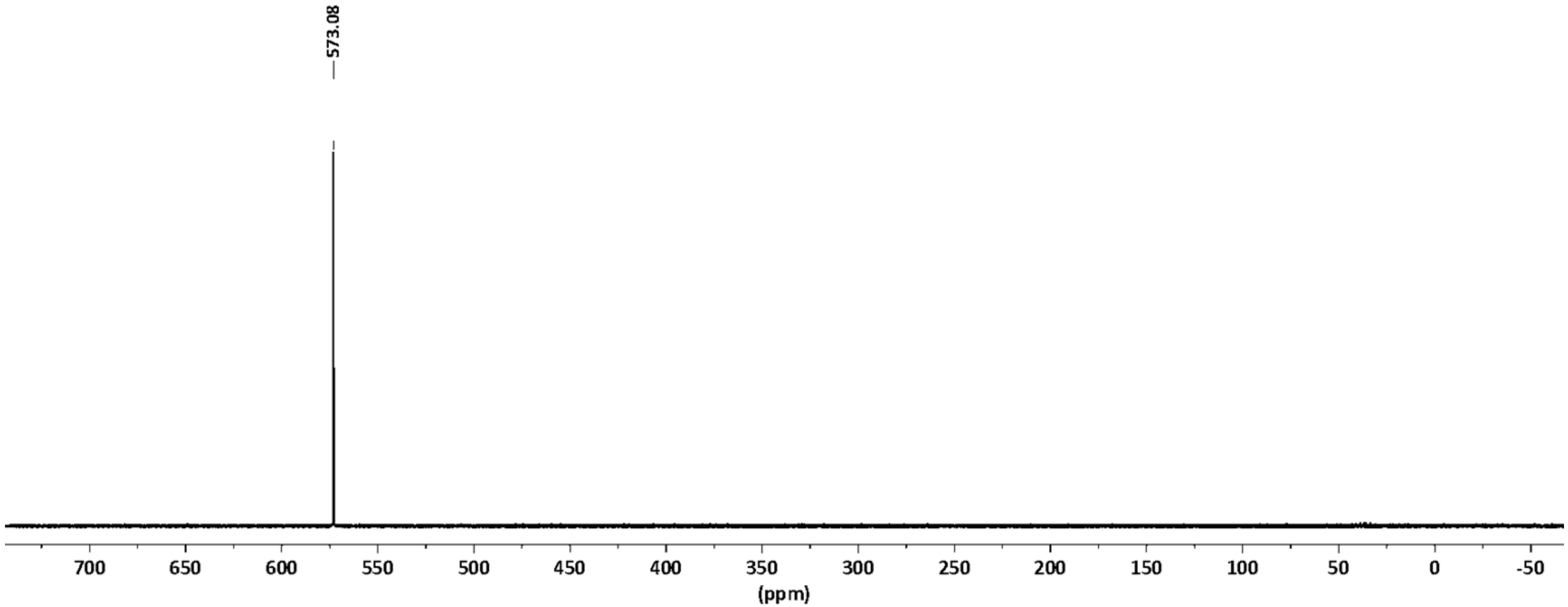

Figure S24.
^{31}P NMR (CD_2Cl_2, 243 MHz) spectrum of **6**.

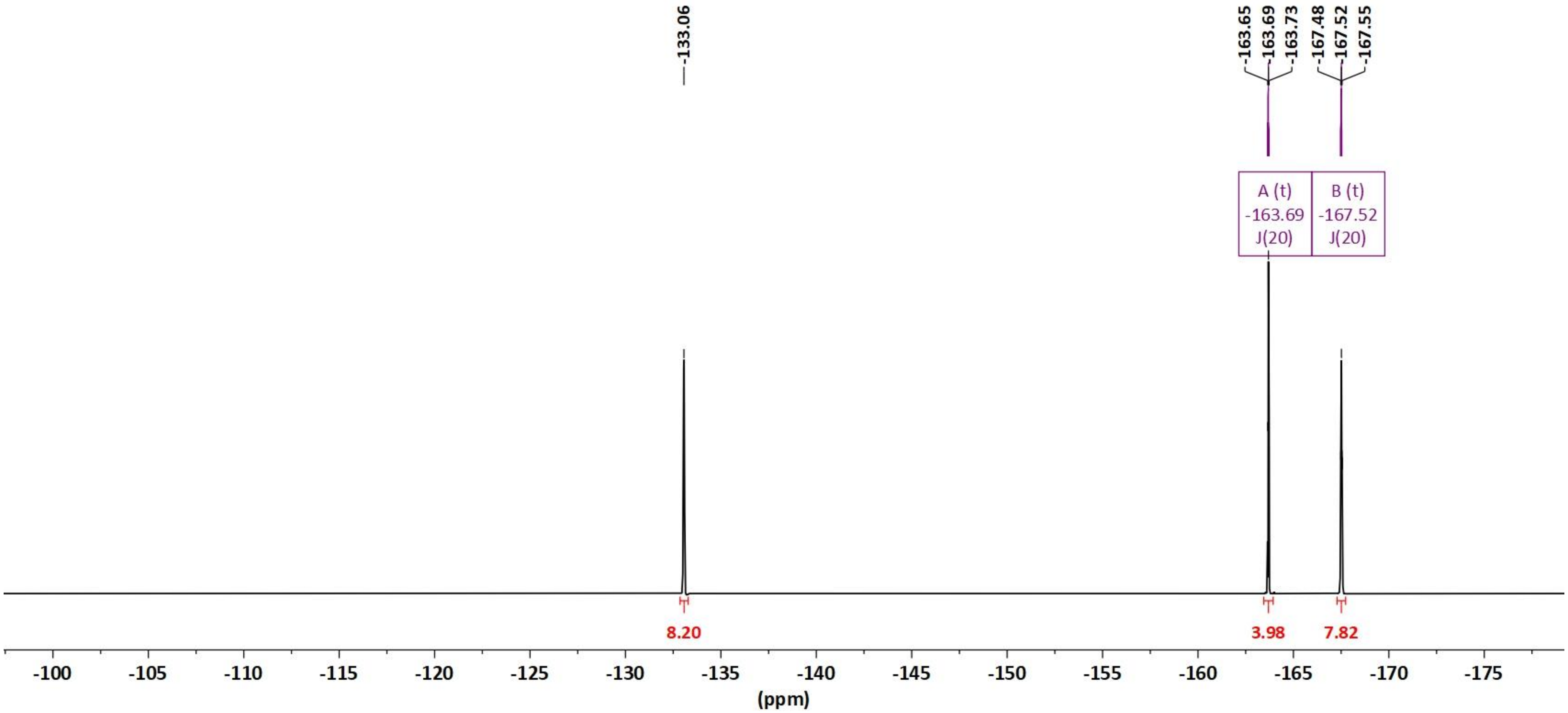

Figure S25.
^{19}F NMR (CD_2Cl_2, 565 MHz) spectrum of **6**.

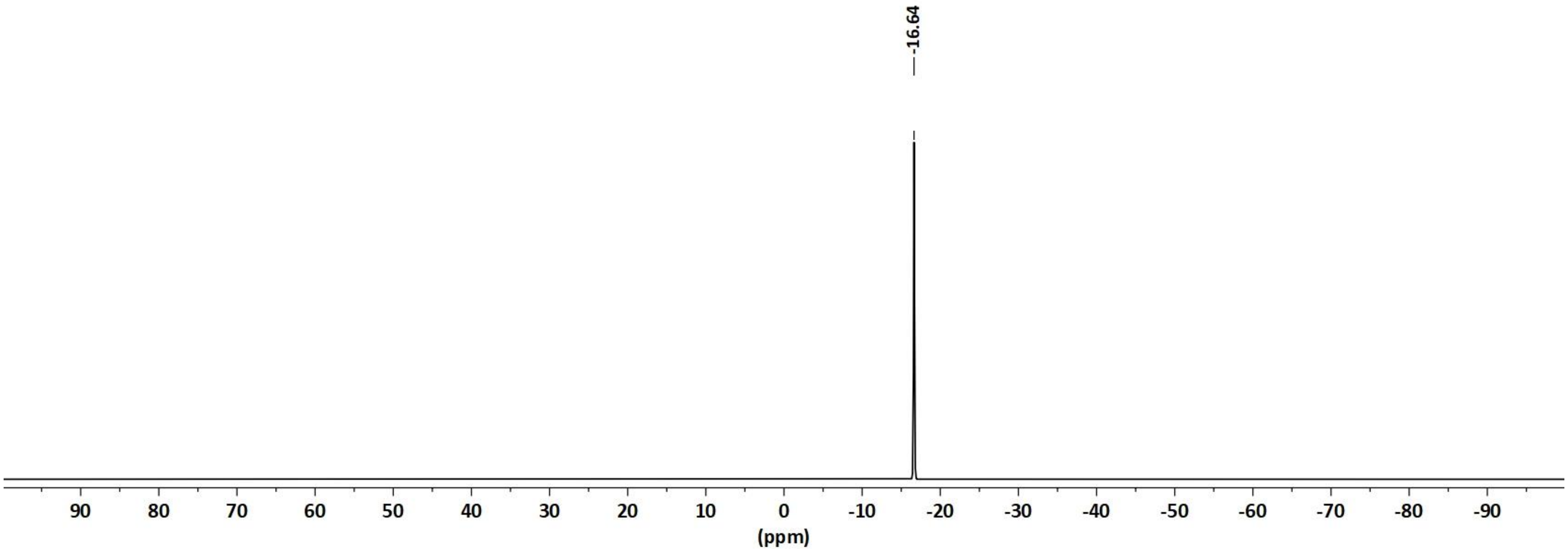

Figure S26.
^{11}B NMR (CD_2Cl_2, 193 MHz) spectrum of **6**.

Synthesis and characterization of [Ar^{1}MesAs][B(C_6F_5)$_4$] (**7**)

To a solid mixture of **5** (1.00 g, 1.34 mmol) and K[B(C_6F_5)$_4$] (0.966 g, 1.34 mmol) was added CH_2Cl_2 (20 mL) and the reaction mixture was stirred at room temperature for at least 30 minutes. The color of the solution changed immediately to blood-red. The reaction mixture was filtered under argon through a PTFE syringe filter to remove KCl. Hexane (65 mL) was layered over the CH_2Cl_2 filtrate. The crystalline product was washed with toluene (3×10 mL) and dried at reduced pressure. The title compound was obtained as a dark red solid (1.440 g, 78%).

Mp. 259–261 °C. **^{1}H NMR (600 MHz, CD_2Cl_2)**: δ = 7.47 (s and t overlapped, 3H, H4, H20), 7.41 (m, 4H, H19, H21), 7.38 (d, $^3J(^1H–^1H)$ = 8 Hz, 2H, H18), 7.34 (d, $^3J(^1H–^1H)$ = 8 Hz, 2H, H11), 7.17 (t, $^3J(^1H–^1H)$ = 7 Hz, 2H, H12), 6.98 (t, $^3J(^1H–^1H)$ = 7 Hz, 2H, H13), 6.88 (d, $^3J(^1H–^1H)$ = 8 Hz, 2H, H14), 6.83 (s, br, 1H, H24), 6.12 (s, br, 1H, H26), 2.66 (d, $^2J(^1H–^1H)$ = 15 Hz, 2H, H6a), 2.56 (d, $^2J(^1H–^1H)$ = 15 Hz, 2H, H6b), 2.17 (s, 3H, H29), 2.15 (s, br, 3H, H30), 1.77 (s, 6H, H8), 1.65 (s, 6H, H9), 0.70 (s, 3H, H28). **$^{13}C\{^1H\}$ NMR (151 MHz, CD_2Cl_2)**: δ = 161.30 (s, C22), 156.09 (s, C3), 152.13 (s, C25), 151.39 (s, C10), 150.21 (s, C2), 149.52 (s, C17), 148.72 (d, br, $^1J(^{13}C–^{19}F)$ = 242 Hz, C_6F_5), 147.09 (s, C23), 147.05 (s, C27), 144.43 (s, C1), 142.28 (s, C16), 138.81 (dm, br, $^1J(^{13}C–^{19}F)$ = 243 Hz, C_6F_5), 137.08 (s, C15), 136.88 (d, $^1J(^{13}C–^{19}F)$ = 246 Hz, C_6F_5), 133.53 (s, C20), 131.95 (s, C19), 131.41 (s, C26), 130.82 (s, C24), 130.34 (s, C12), 129.07 (s, C18), 128.98 (s, C13), 126.02 (s, C11), 124.63 (s, br, *i*-C_6F_5), 123.88 (s, C21), 122.12 (s, C4), 121.51 (s, C14), 63.37 (s, C5), 53.17 (s, C6), 46.68 (s, C7), 33.20 (s, C9), 32.60 (s, C8), 24.16 (s, C29), 22.55 (s, C30), 21.45 (s, C28). **^{19}F NMR (565 MHz, CD_2Cl_2)**: δ = −133.06 (br, 8F, *o*-C_6F_5), −163.69 (t, $^3J(^{19}F–^{19}F)$ = 20 Hz, 4F, *p*-C6F5), −167.52 (t, br, $^3J(^{19}F–^{19}F)$ = 20 Hz, 8F, *m*-C_6F_5). **^{11}B NMR (193 MHz, CD_2Cl_2)**: δ = −16.64 (s). **HRMS ESI (m/z):** $[M]^+$ calculated. for $C_{49}H_{44}As$, 707.26535; found, 707.26484.

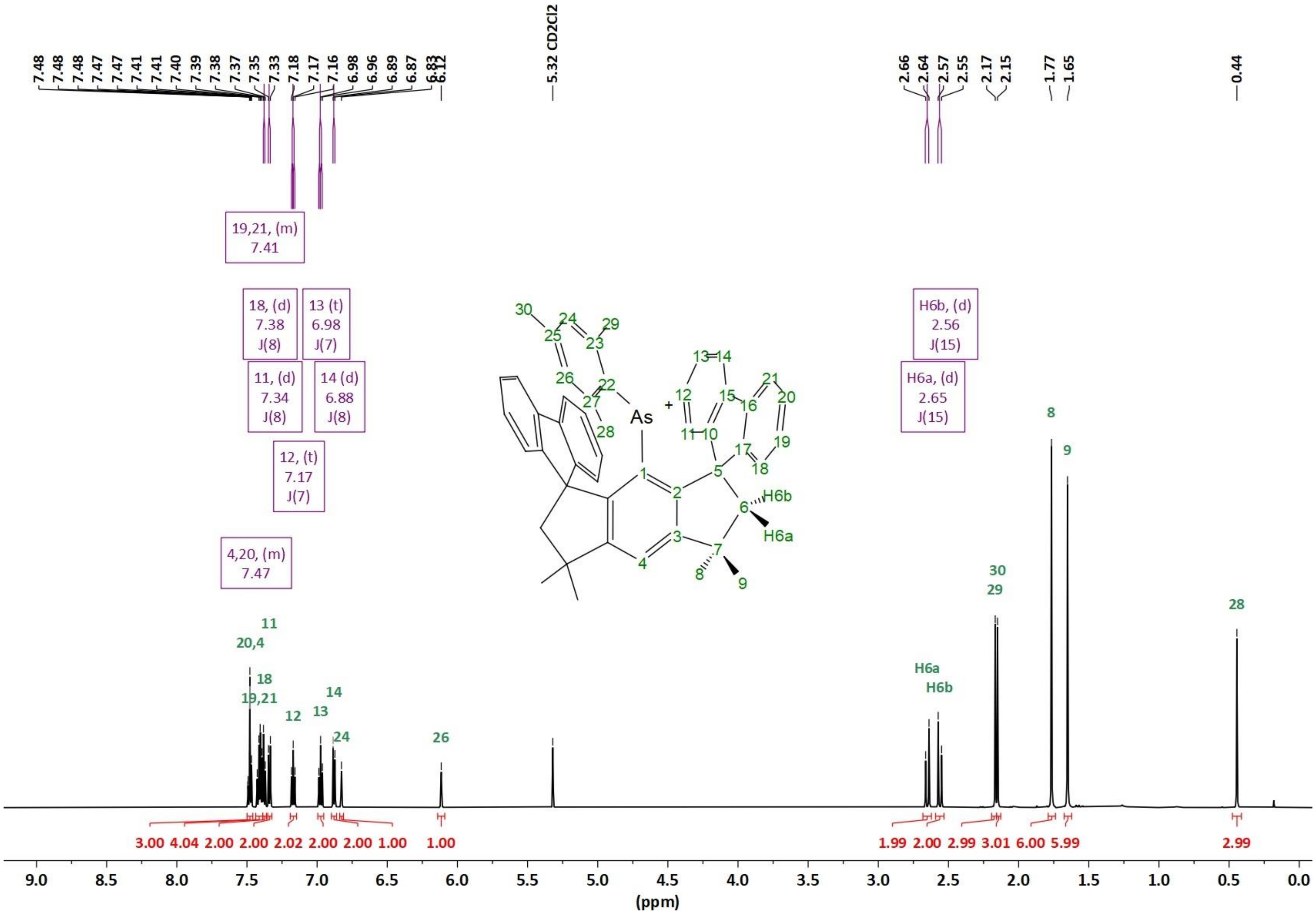

Figure S27.
^{1}H NMR (CD_2Cl_2, 600 MHz) spectrum of **7**.

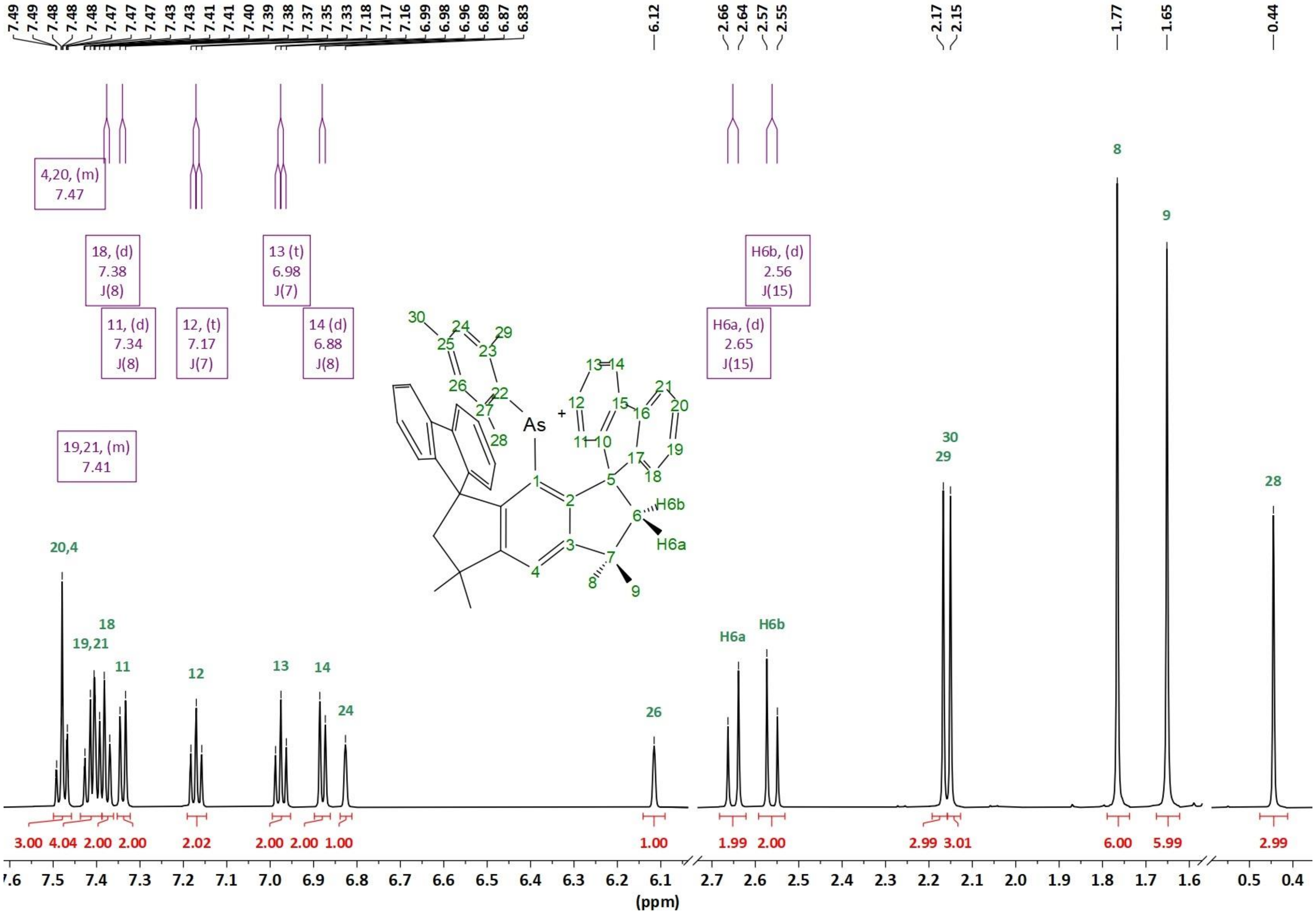

Figure S28.
Detailed ^{1}H NMR (CD_2Cl_2, 600 MHz) spectrum of **7**.

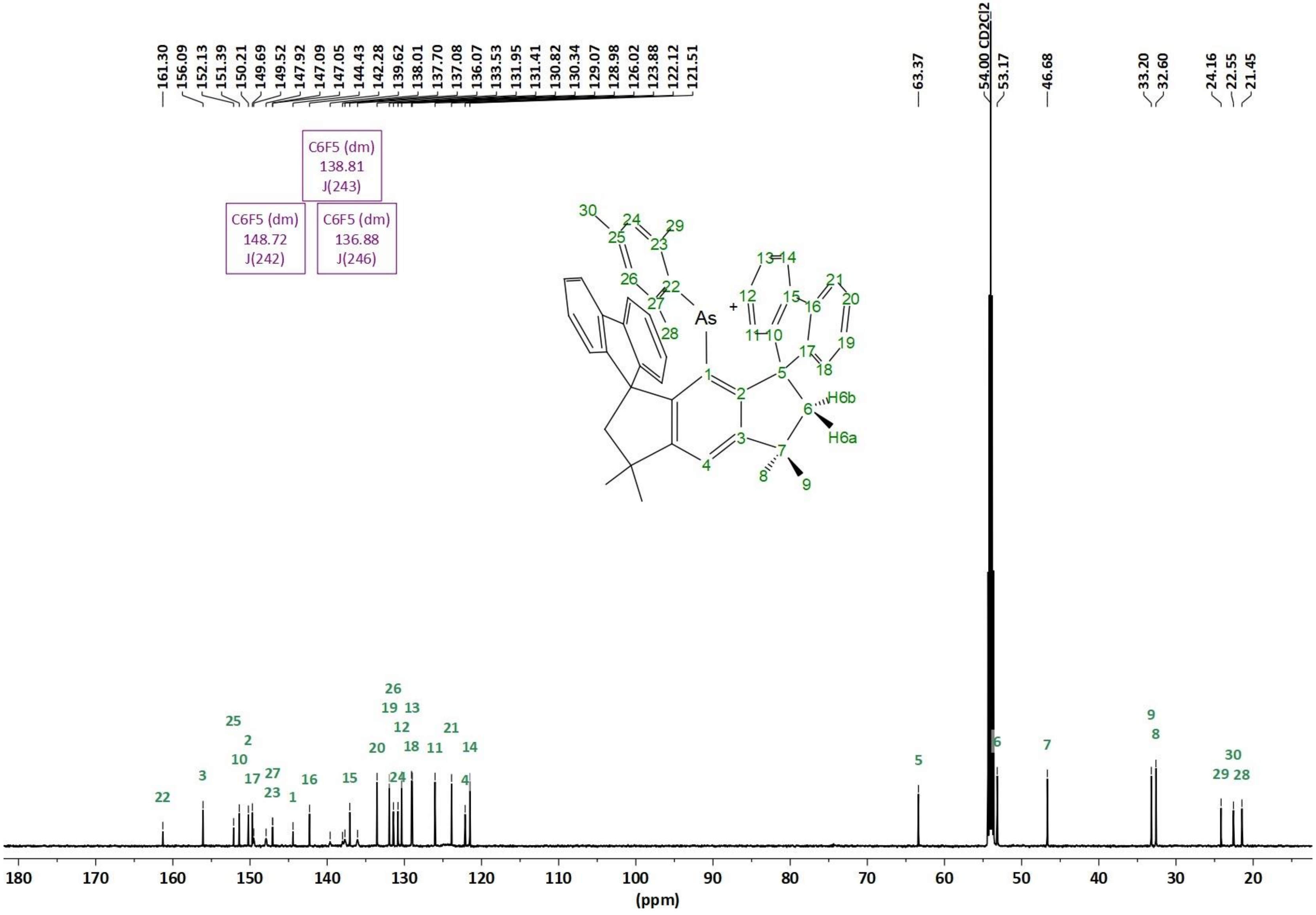

Figure S29.
$^{13}C\{^1H\}$ NMR (CD_2Cl_2, 151 MHz) spectrum of **7**.

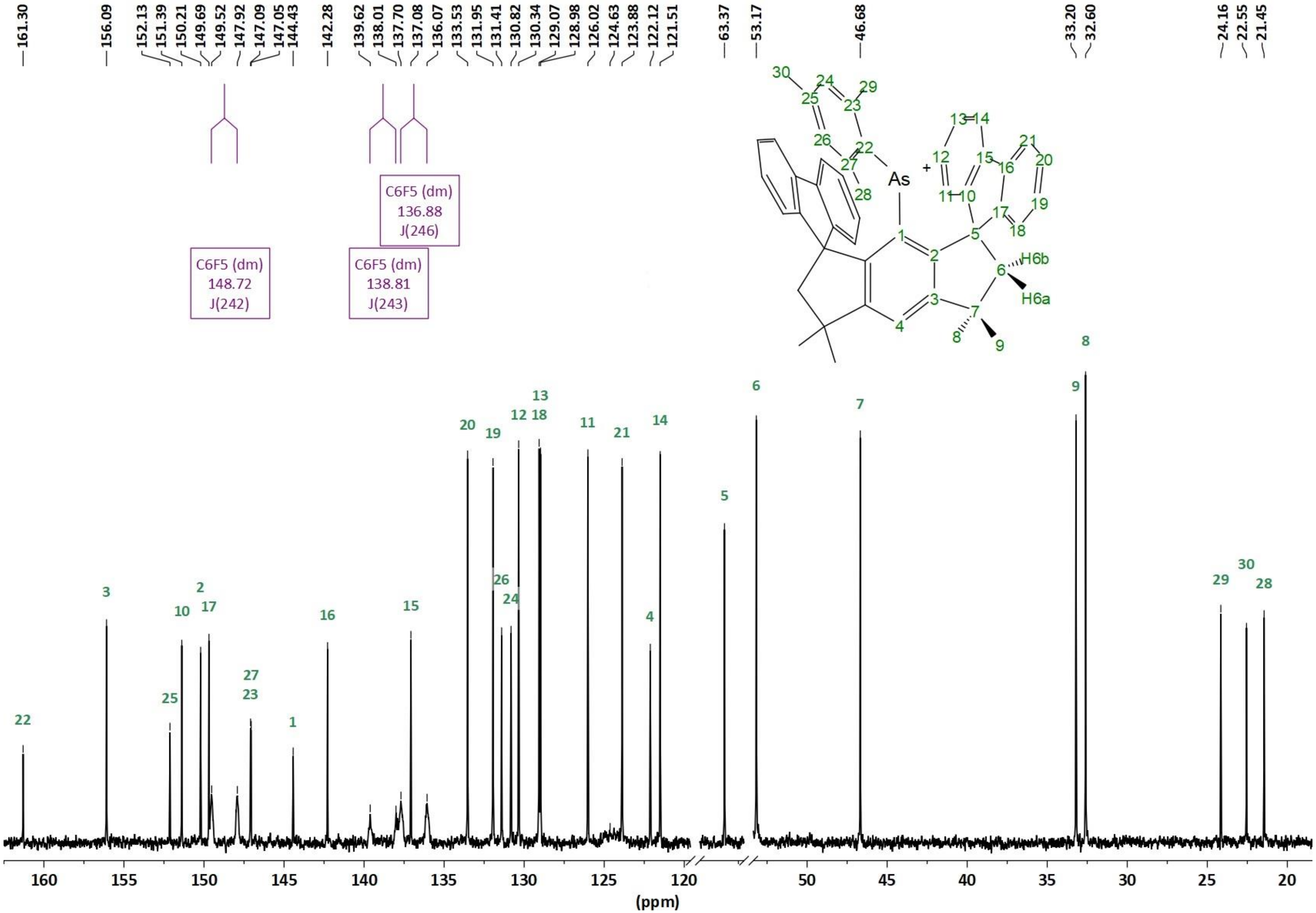

Figure S30.
Detailed $^{13}C\{^{1}H\}$ NMR (CD_2Cl_2, 151 MHz) spectrum of **7**.

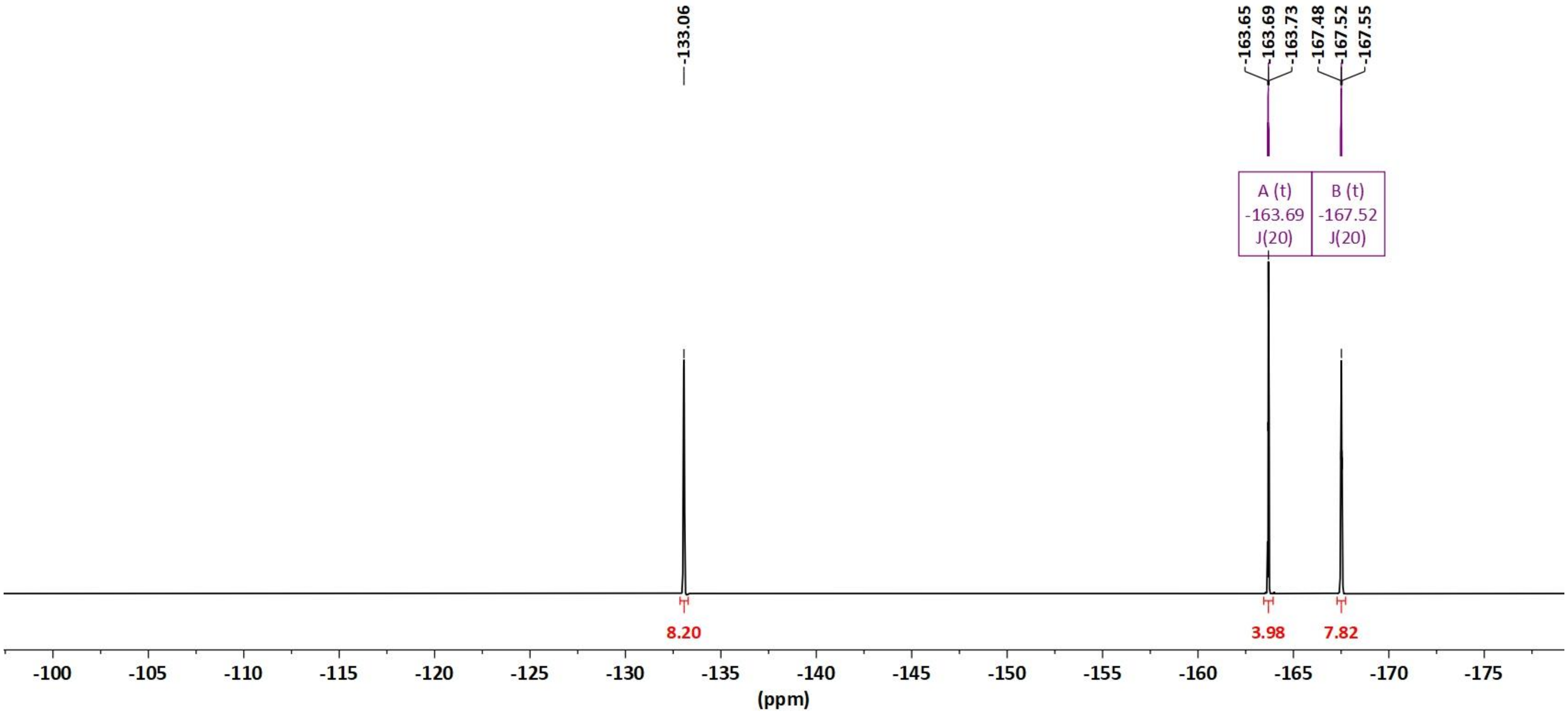

Figure S31.

^{19}F NMR (CD_2Cl_2, 565 MHz) spectrum of **7**.

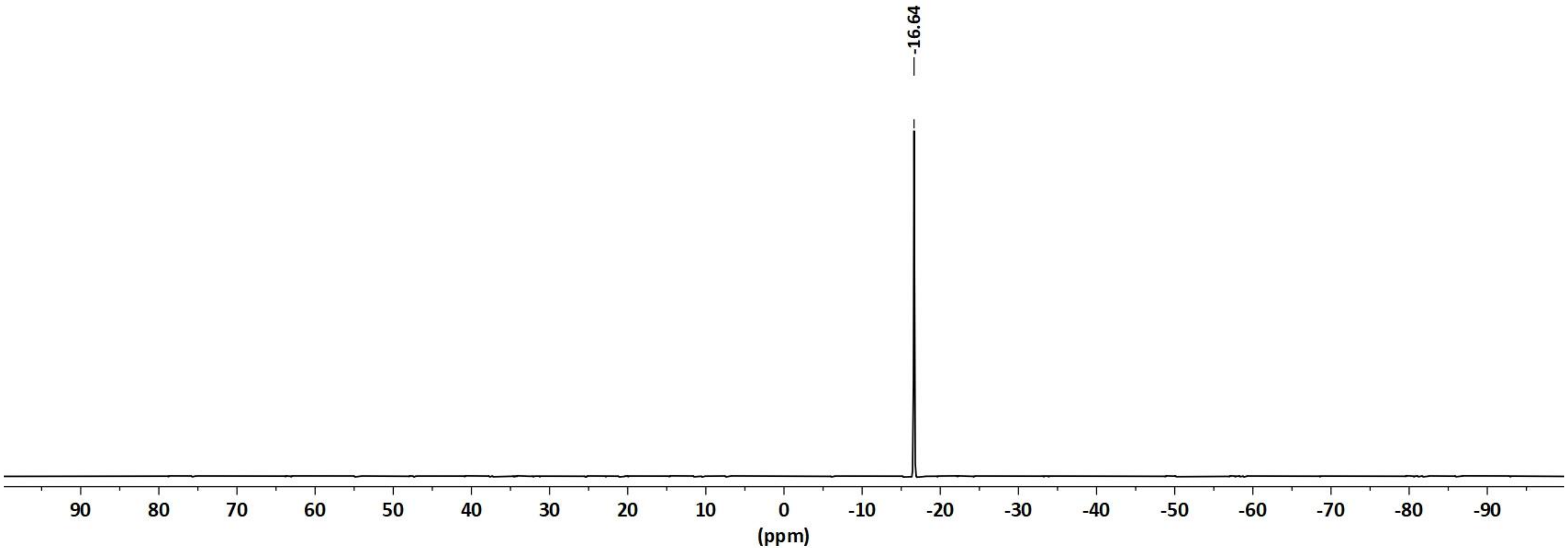

Figure S32.
^{11}B NMR (CD_2Cl_2, 193 MHz) spectrum of **7**.

Synthesis and characterization of [Ar^{1}PhP][B(C_6F_5)$_4$] (**8**)

To a suspension of **1** (0.740 g, 1.11 mmol) in toluene (5 mL) was added $PhPCl_2$ (0.220 g, 1.23 mmol). The reaction mixture was stirred at room temperature for 45 minutes, then all volatiles were removed at reduced pressure, and the remaining solid dried 45 minutes at 60 °C (10^{-3} mbar). K[B(C_6F_5)$_4$] (0.800 g, 1.11 mmol) followed by CH_2Cl_2 (10 mL) were added and the reaction mixture was stirred at room temperature for 45 minutes. The color of the solution changed immediately to dark purple. The reaction mixture was filtered under argon through a PTFE syringe filter to remove LiCl and KCl. Hexane (30 mL) was layered over the CH_2Cl_2 filtrate. The crystalline product was washed with warm (60 °C) toluene (5×15 mL) and dried at reduced pressure (at 60 °C, 10^{-3} mbar). The title compound was obtained as a dark red (almost black) solid (0.868 g, 60%).

Mp. 208–210 °C. **^{1}H NMR (600 MHz, CD_2Cl_2)**: δ = 7.56 (ddd, $^3J(^1H–^1H)$ = 8 Hz, $^3J(^1H–^1H)$ = 7 Hz, $^4J(^1H–^1H)$ = 1 Hz, 2H, H20), 7.49 (s, 1H, H4), 7.45 (d, $^3J(^1H–^1H)$ = 8 Hz, 2H, H18), 7.38 (ddd, $^3J(^1H–^1H)$ = 8 Hz, $^3J(^1H–^1H)$ = 7 Hz, $^4J(^1H–^1H)$ = 1 Hz, 2H, H19), 7.33 (td, $^3J(^1H–^1H)$ = 8 Hz, $^4J(^1H–^1H)$ = 1 Hz, 1H, H24), 7.28 (m, 5H, H11, H21, H25), 7.08 (td, $^3J(^1H–^1H)$ = 8 Hz, $^4J(^1H–^1H)$ = 1 Hz, 2H, H12), 7.05 (m, br, 1H, H23), 6.75 (td, $^3J(^1H–^1H)$ = 7 Hz, $^4J(^1H–^1H)$ = 1 Hz, 2H, H13), 6.52 (d, $^3J(^1H–^1H)$ = 8 Hz, 2H, H14), 6.28 (td, $^3J(^1H–^1H)$ = 8 Hz, $^4J(^1H–^1H)$ = 1 Hz, 1H, H26), 4.94 (d, br, $^3J(^1H–^1H)$ = 8 Hz, 1H, H27), 2.64 (d, $^2J(^1H–^1H)$ = 15 Hz, 2H, H6a), 2.52 (d, $^2J(^1H–^1H)$ = 15 Hz, 2H, H6b), 1.79 (s, 6H, H8), 1.64 (s, 6H, H9). **^{13}C{^{1}H} NMR (151 MHz, CD_2Cl_2)**: δ = 154.68 (s, C3), 151.63 (s, C10), 151.50 (s, C2), 149.86 (d, $^1J(^{13}C–^{31}P)$ = 72 Hz, C22), 148.78 (s, C17), 148.72 (d, br, $^1J(^{13}C–^{19}F)$ = 241 Hz, *C*$_6$F$_5$), 145.60 (s, C16), 139.62 (s, C25), 138.81 (dm, br, $^1J(^{13}C–^{19}F)$ = 245 Hz, *C*$_6$F$_5$), 136.87 (d, $^1J(^{13}C–^{19}F)$ = 246 Hz, *C*$_6$F$_5$), 136.83 (s, C15), 136.41 (s, C27), 135.20 (s, C20), 131.83 (s, C19), 131.26 (d, $^1J(^{13}C–^{31}P)$ = 64 Hz, C1), 130.87 (s, C18), 130.15 (s, C12), 129.49 (d, $^3J(^{13}C–^{31}P)$ = 11 Hz, C24), 129.00 (s, C13), 128.46 (s, C26), 125.51 (s, C11), 124.48 (s, br, *i*-C_6F_5), 123.57 (s, C21), 122.87 (s, C4), 121.32 (s, C14), 62.36 (s, C5), 53.20 (s, C6), 46.87 (s, C7), 33.42 (s, C9), 32.14 (s, C8). **^{31}P NMR (243 MHz, CD_2Cl_2)**: δ = 541.97 (s). **^{19}F NMR (565 MHz, CD_2Cl_2)**: δ = −133.06 (br, 8F, *o*-C_6F_5), −163.61 (t, $^3J(^{19}F–^{19}F)$ = 20 Hz, 4F, *p*-C6F5), −167.45 (s, br, 8F, *m*-C_6F_5). **^{11}B NMR (193 MHz, CD_2Cl_2)**: δ = −16.64 (s). **HRMS ESI (m/z):** [M+H_2O]$^+$ calculated. for $C_{46}H_{40}PO$, 639.28113; found, 639.28124.

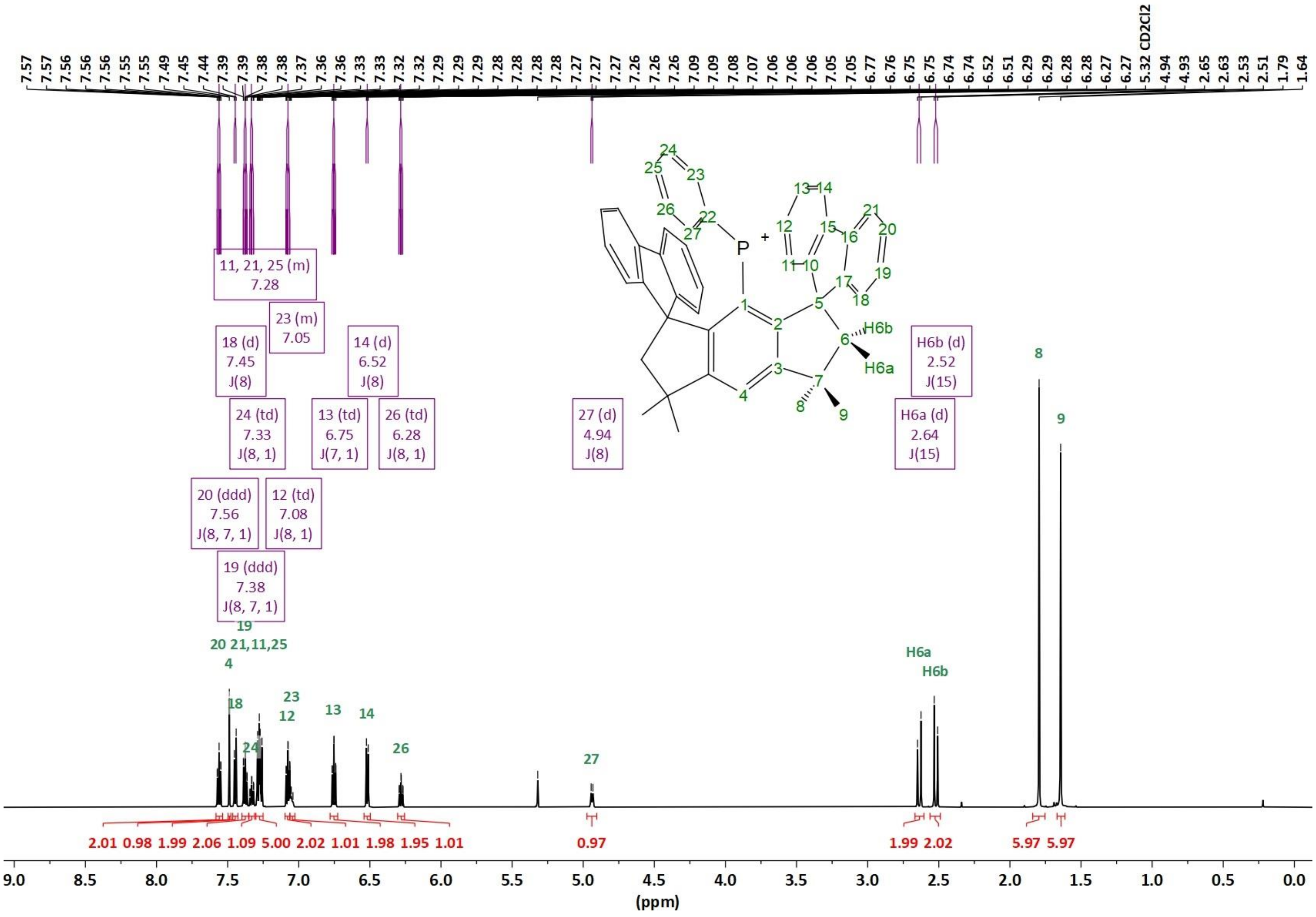

Figure S33.
^{1}H NMR (CD_2Cl_2, 600 MHz) spectrum of **8**.

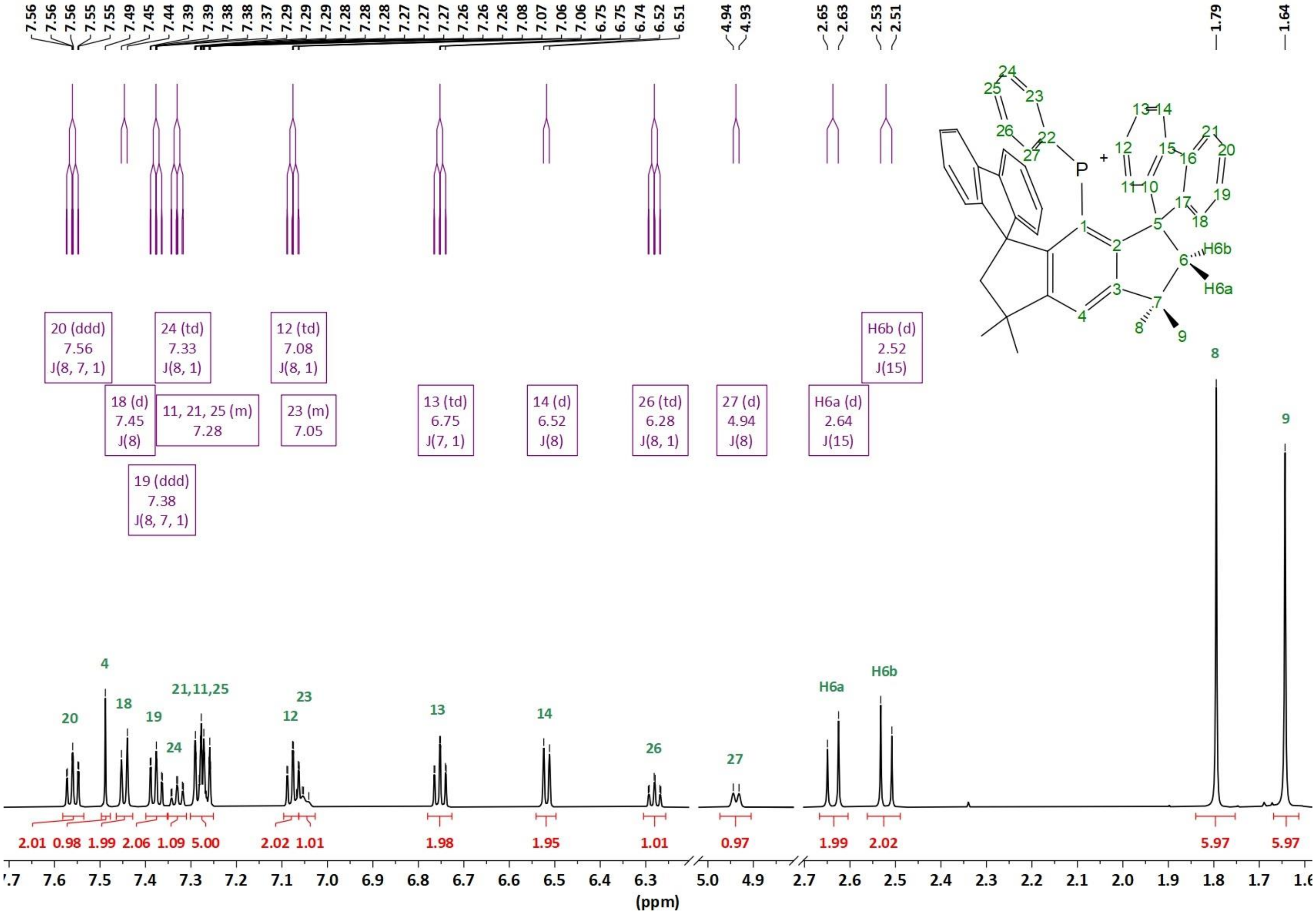

Figure S34.
Detailed ^{1}H NMR (CD_2Cl_2, 600 MHz) spectrum of **8**.

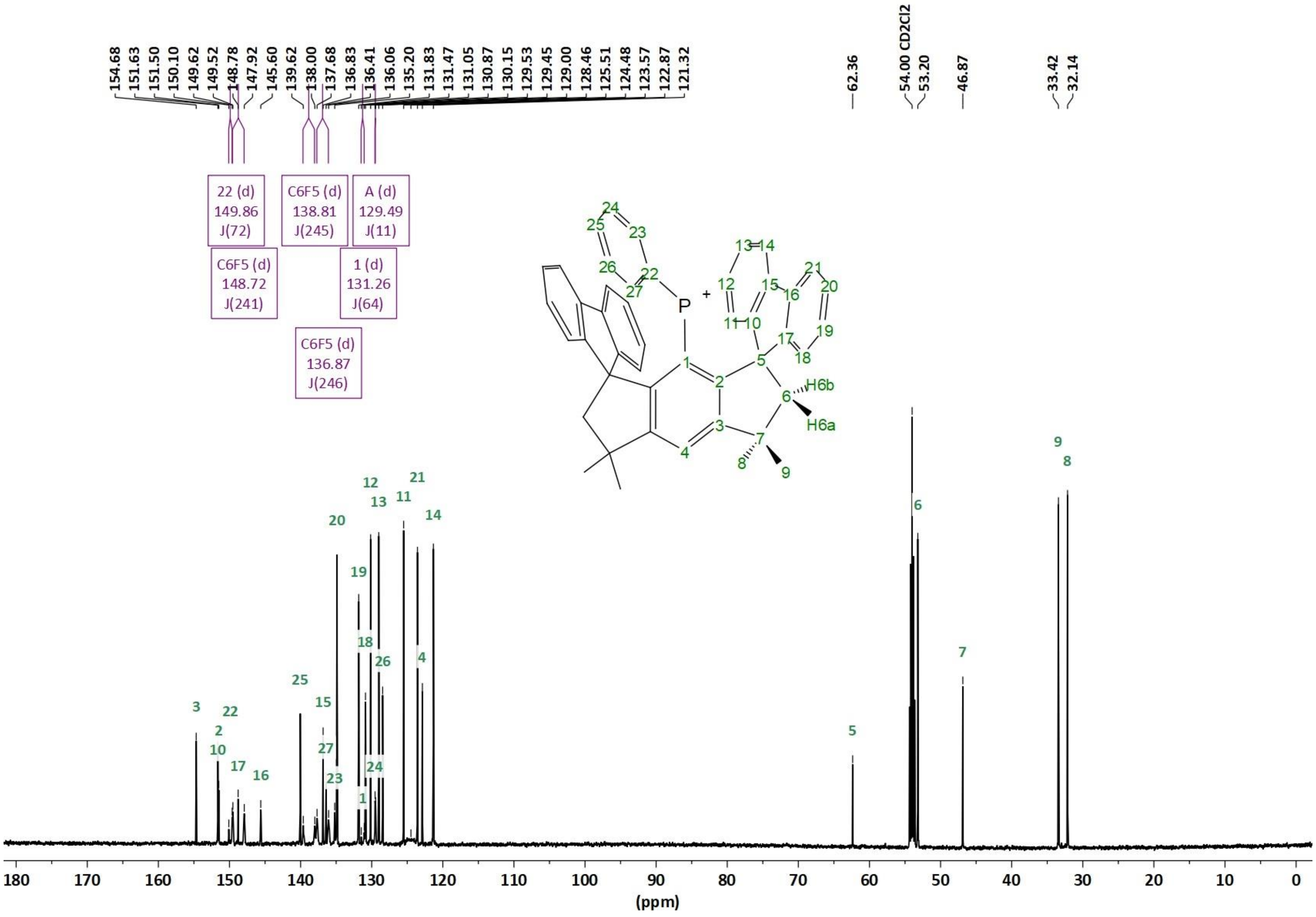

Figure S35.
$^{13}C\{^{1}H\}$ NMR (CD_2Cl_2, 151 MHz) spectrum of **8**.

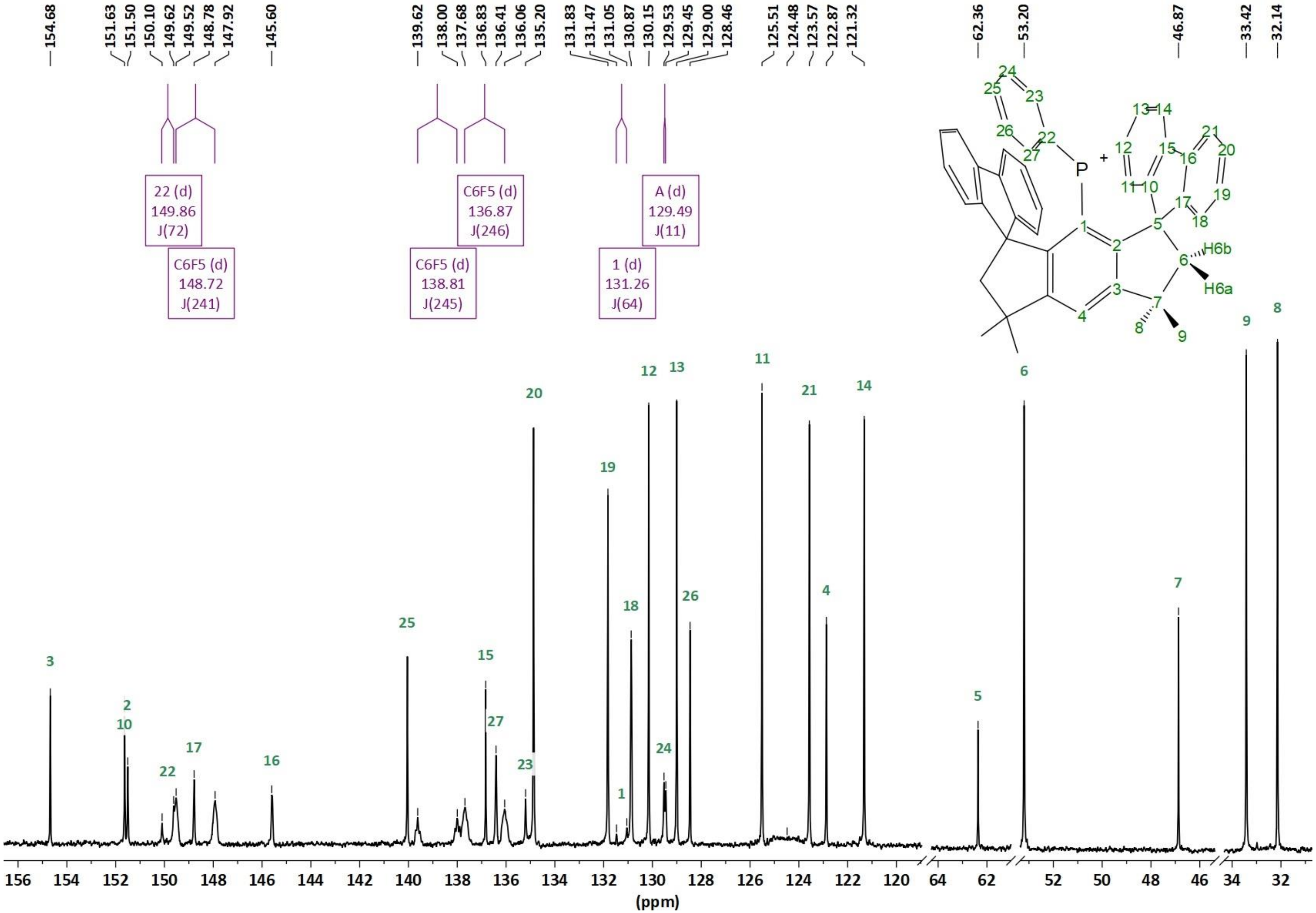

Figure S36.
Detailed $^{13}C\{^1H\}$ NMR (CD_2Cl_2, 151 MHz) spectrum (aromatic area) of **8**.

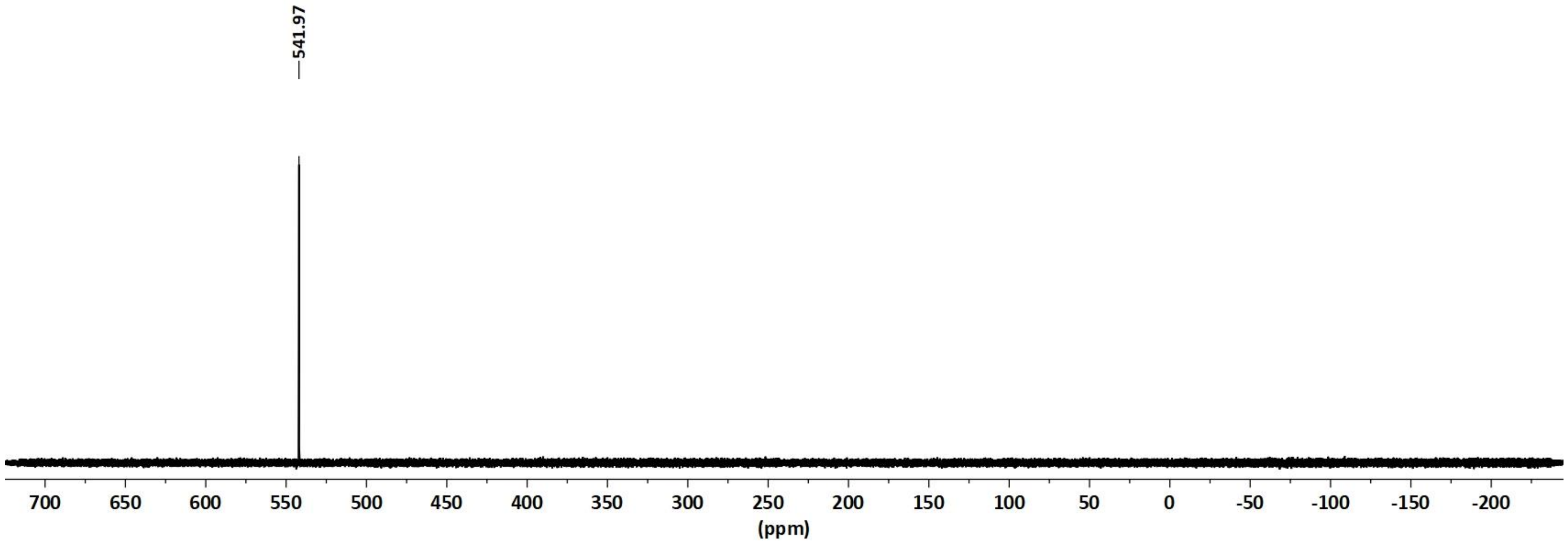

Figure S37.
^{31}P NMR (CD_2Cl_2, 243 MHz) spectrum of **8**.

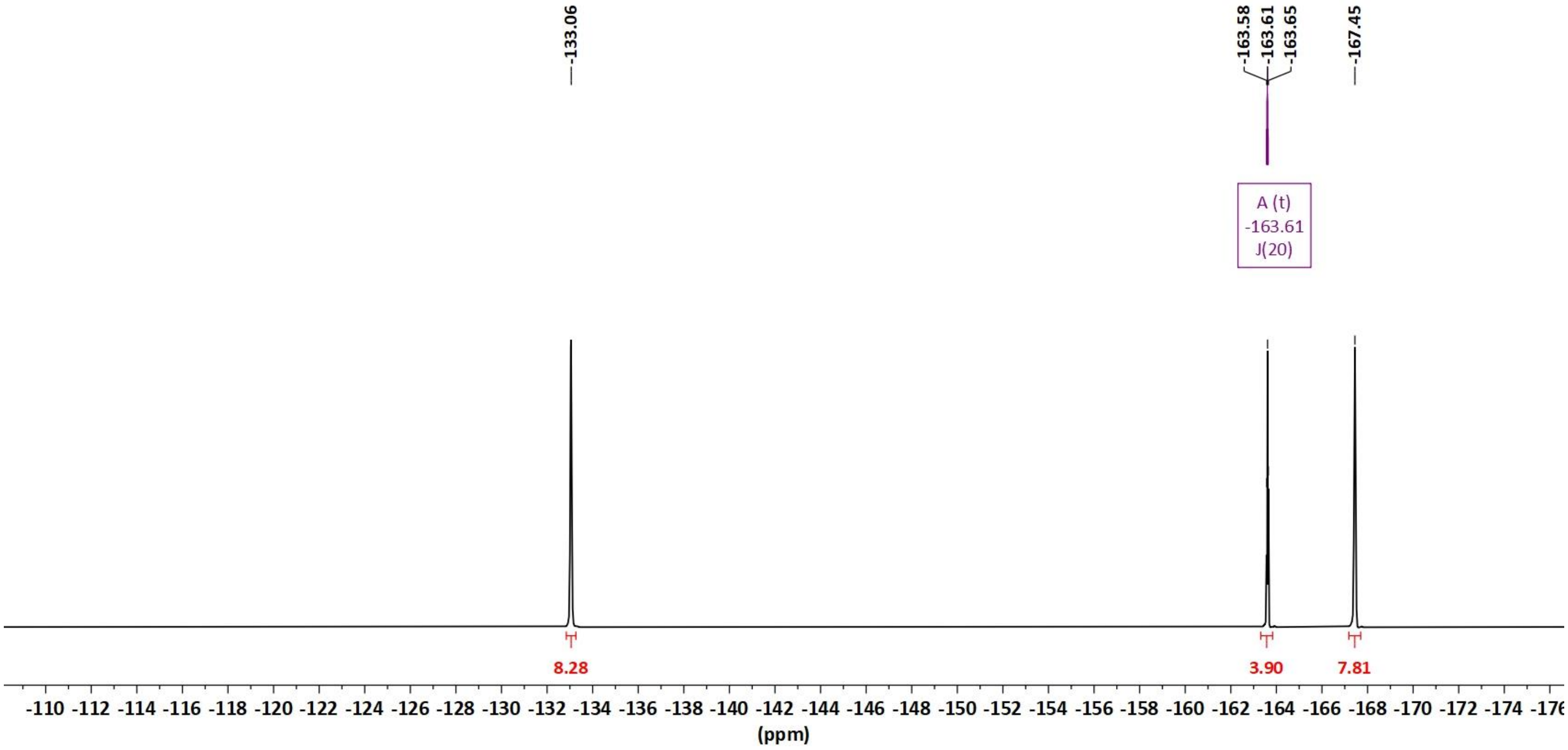

Figure S38.
^{19}F NMR (CD_2Cl_2, 565 MHz) spectrum of **8**.

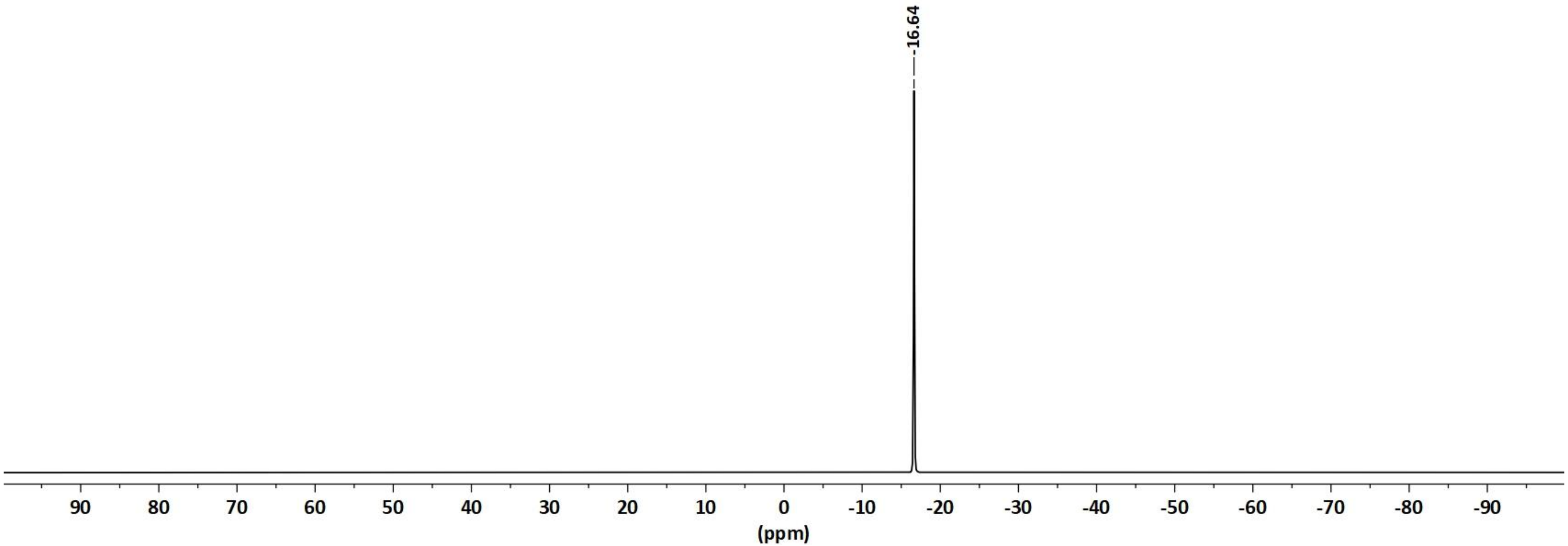

Figure S39.
^{11}B NMR (CD_2Cl_2, 193 MHz) spectrum of **8**.

UV-Vis spectra

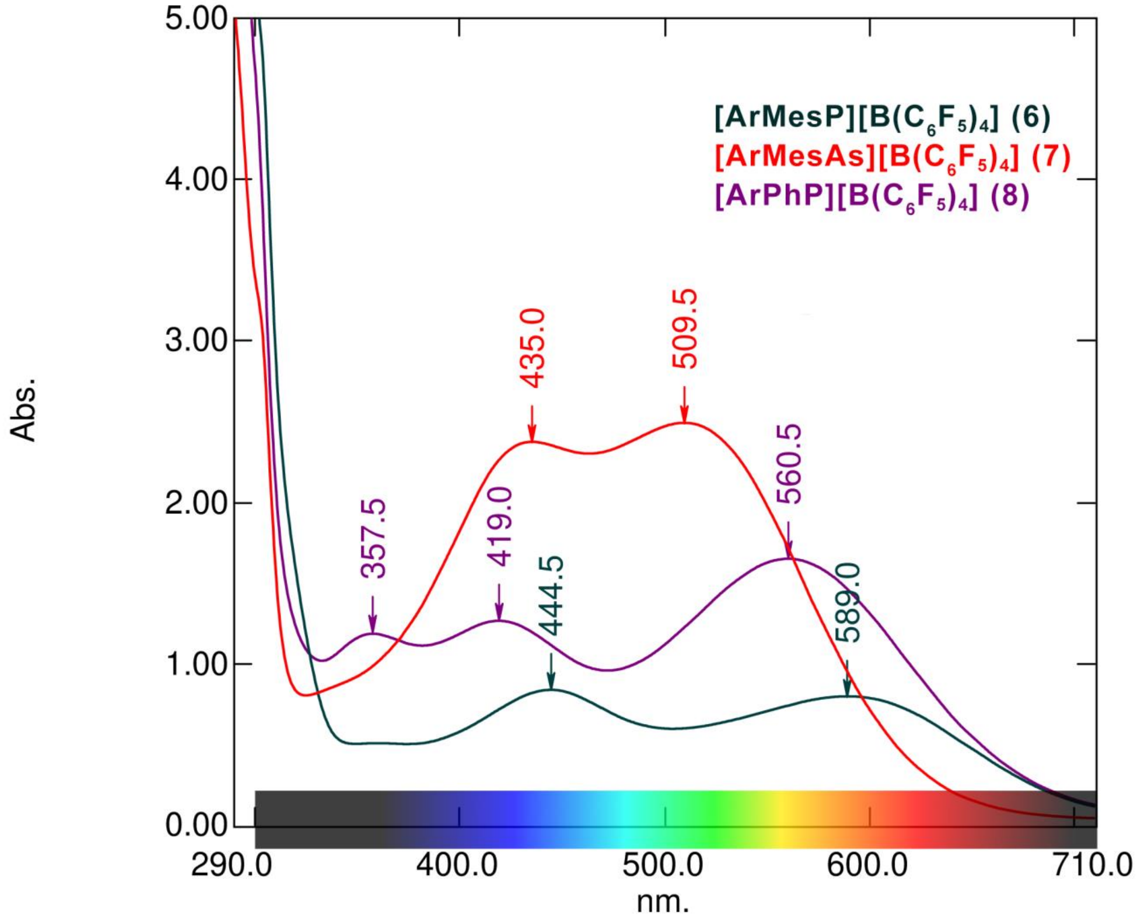

Figure S40.

UV-Vis spectra of **6**, **7**, and **8**.

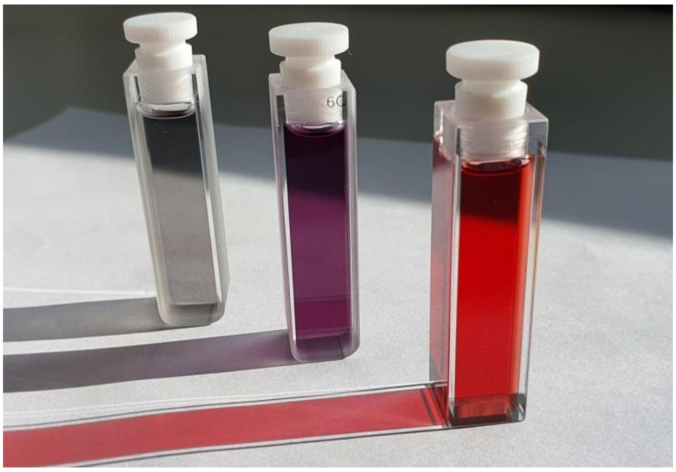

Figure S41.

Left to right: diluted solutions (CH_2Cl_2) of **6, 8** and **7**.

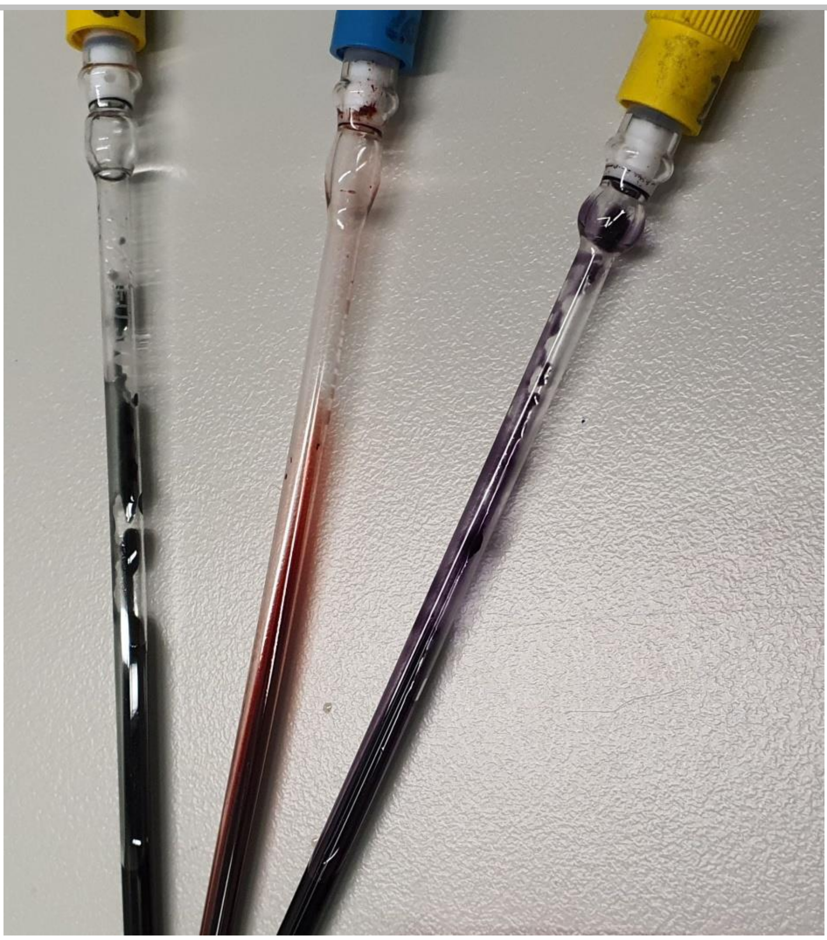

Figure S42.
Left to right: concentrated solutions (CD_2Cl_2) of **6**, **7**, and **8**.

Thermal stability tests

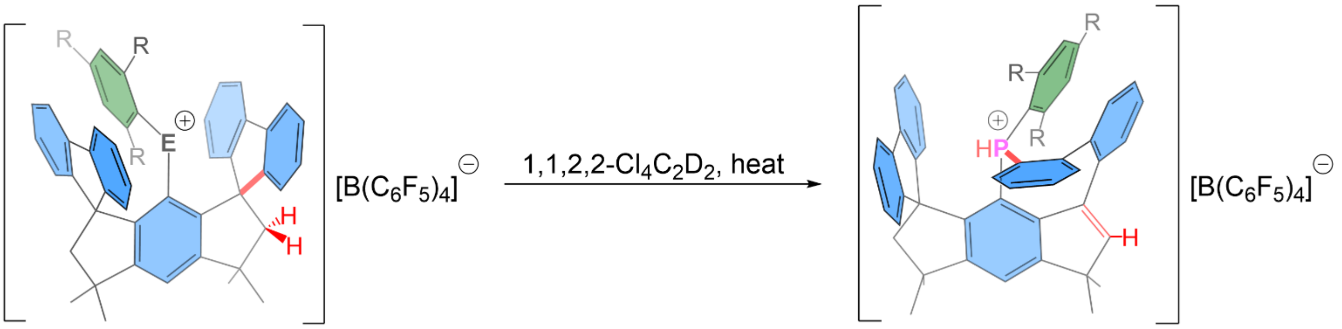

6, R = Me, E = P
7, R = Me, E = As
8, R = H, E = P

9, R = Me
10, R = H

Under argon atmosphere a J. Young NMR tube was loaded with **6**–**8** (*ca.* 23 mg) and 1,1,2,2-tetrachloroethan-d2 (0.5 mL). The solutions were heated at different temperatures and the progression of the reactions was monitored by ^{1}H and ^{31}P NMR. Only **6** and **8** decomposed thermally between 60–150 °C. The progression of the reactions giving **9** and **10** as the major decomposition products is summarized in Table S1 and S2. Arsenium compound **7** was stable for at least 1.5 h at 150 °C and did not show any decomposition products after prolonged heating (12–24 h) at lower temperatures (60, 100, 120 °C) either.

Table S1.
Thermal decomposition of **6**.

Temperature [°C]	time [h]	molar ratio between	
		**6**	**9**
60	15	1	0.03
80	7	1	0.13
100	8	1	0.78
100	24	1	3.10
125	24	0	1

at 150 °C a sample decomposed completely in circa 3 h.

Table S2.
Thermal decomposition of **8**.

Temperature [°C]	time [h]	molar ratio between	
		**8**	**10**
60	15	1	0.03
80	7	1	0.03
100	8	1	0.10
125	14	1	2.6
125	36	0	1

at 150 °C a sample decomposed completely in circa 5 h.

Characterization of **9**

^{1}H NMR (600 MHz, CD_2Cl_2): δ = 7.94 (d, $^3J(^1H–^1H)$ = 8 Hz, 1H, H40), 7.69 (td, $^3J(^1H–^1H)$ = 8 Hz, $^4J(^1H–^1H)$ = 1 Hz, 1H, H39), 7.60 (d, $^5J(^1H–^{31}P)$ = 2 Hz, 1H, H4), 7.57 (m, 3H, H15, H38, H43), 7.48 (d, $J(^1H–^1H)$ = 8 Hz, 1H, H37), 7.28 (ddd, $^3J(^1H–^1H)$ = 8 Hz, $^4J(^1H–^1H)$ = 1 Hz, $^4J(^1H–^{31}P)$ = 5 Hz, 1H, H14), 7.25 (m, 2H, H16, H46), 7.17 (pseudo-doublet, 2H, H22, H23), 7.03 (d, $^1J(^1H–^{31}P)$ = 488 Hz, 1H, P*H*), 6.92 (m, 2H, H44, H45), 6.89 (m, 1H, H21), 6.60 (s, 1H, H8), 6.51 (d, br, $^4J(^1H–^{31}P)$ = 5 Hz, 1H, H26), 6.50 (d, $^3J(^1H–^1H)$ = 8 Hz, 1H, H20), 6.32 (dd, $^4J(^1H–^{31}P)$ = 5 Hz, $^4J(^1H–^1H)$ = 2 Hz, 1H, H28), 5.84 (dd, $^3J(^1H–^{31}P)$ = 18 Hz, $^3J(^1H–^1H)$ = 8 Hz, 1H, H17), 2.74 (d, $^2J(^1H–^1H)$ = 14 Hz, 1H, H34b), 2.65 (d, $^2J(^1H–^1H)$ = 14 Hz, 1H, H34a), 2.09 (s, 3H, H31), 1.71 (s, 3H, H48), 1.70 (s, 3H, H32), 1.61 (s, 3H, H49), 1.50 (s, 3H, H11), 1.42 (s, 3H, H30), 1.29 (s, 3H, H10). **^{13}C{^{1}H} NMR (151 MHz, CD_2Cl_2)**: δ = 157.12 (d, $^3J(^{13}C–^{31}P)$ = 11 Hz, C3), 155.41 (s, C8), 154.17 (d, $^3J(^{13}C–^{31}P)$ = 10 Hz, C5), 152.87 (s, C36), 152.03 (s, C47), 149.77 (d, $^2J(^{13}C–^{31}P)$ = 7 Hz, C2), 147.94 (d, br, $^1J(^{13}C–^{19}F)$ = 241 Hz, *C*$_6$F$_5$), 144.55 (d, $^4J(^{13}C–^{31}P)$ = 3 Hz, C27), 144.35 (d, $^2J(^{13}C–^{31}P)$ = 9 Hz, C13), 143.42 (d, $^2J(^{13}C–^{31}P)$ = 7 Hz, C6), 142.94 (d, $^2J(^{13}C–^{31}P)$ = 10 Hz, C29), 140.28 (d, $^2J(^{13}C–^{31}P)$ = 12 Hz, C25), 140.07 (d, $^3J(^{13}C–^{31}P)$ = 4 Hz, C7), 139.59 (d, $^5J(^{13}C–^{31}P)$ = 3 Hz, C41), 138.62 (s, C42), 138.27 (d, $^3J(^{13}C–^{31}P)$ = 6 Hz, C19), 137.97 (dm, br, $^1J(^{13}C–^{19}F)$ = 245 Hz, *C*$_6$F$_5$), 136.04 (dm, br, $^1J(^{13}C–^{19}F)$ = 245 Hz, *C*$_6$F$_5$), 134.31 (d, $^4J(^{13}C–^{31}P)$ = 2 Hz, C15), 133.12 (s, C23), 132.91 (s, C18), 131.74 (d, $^2J(^{13}C–^{31}P)$ = 16 Hz, C17), 131.47 (d, $^2J(^{13}C–^{31}P)$ = 12 Hz, C28), 131.06 (d, $^3J(^{13}C–^{31}P)$ = 10 Hz, C14), 129.84 (d, $^3J(^{13}C–^{31}P)$ = 12 Hz, C26), 129.41 (s, C39), 129.34 (s, C22), 129.13 (s, C38), 129.09 (d, $^3J(^{13}C–^{31}P)$ = 15 Hz, C16), 128.89 (s, C21), 128.69 (s, C45), 128.55 (s, C44), 128.14 (s, C20), 125.75 (s, C37), 124.67 (s, C46), 123.47 (s, br, *i*-C_6F_5), 122.40 (d, $^3J(^{13}C–^{31}P)$ = 3 Hz, C4), 121.16 (s, C40), 121.03 (d, $^1J(^{13}C–^{31}P)$ = 90 Hz, C12), 120.30 (s, C43), 108.29 (d, $^1J(^{13}C–^{31}P)$ = 83 Hz, C24), 107.46 (d, $^1J(^{13}C–^{31}P)$ = 77 Hz, C1), 63.29 (s, C33), 55.61 (s, C34), 48.36 (s, C9), 43.34 (s, C35), 33.29 (s, C49), 31.56 (s, C48), 23.98 (s, C11), 23.74 (s, C10), 22.09 (d, $^3J(^{13}C–^{31}P)$ = 6Hz, C32), 21.39 (d, $^3J(^{13}C–^{31}P)$ = 11 Hz, C30), 20.75 (s, 31). **^{31}P NMR (243 MHz, CD_2Cl_2)**: δ = −14.00 (dd, br, $^1J(^{31}P–^1H)$ = 488 Hz, $^3J(^{31}P–^1H)$ = 18 Hz). **^{19}F NMR (565 MHz, CD_2Cl_2)**: δ = −132.41 (br, 8F, *o*-C_6F_5), −162.26 (t, 4F, $^3J(^{19}F–^{19}F)$ = 21 Hz, *p*-C_6F_5), −166.09 (s, br, 8F, *m*-C_6F_5). **^{11}B NMR (193 MHz, CD_2Cl_2)**: δ = −16.65 (s). **HRMS ESI (m/z):** $[M]^+$ calculated. for $C_{49}H_{44}P$, 663.31751; found, 663.31639.

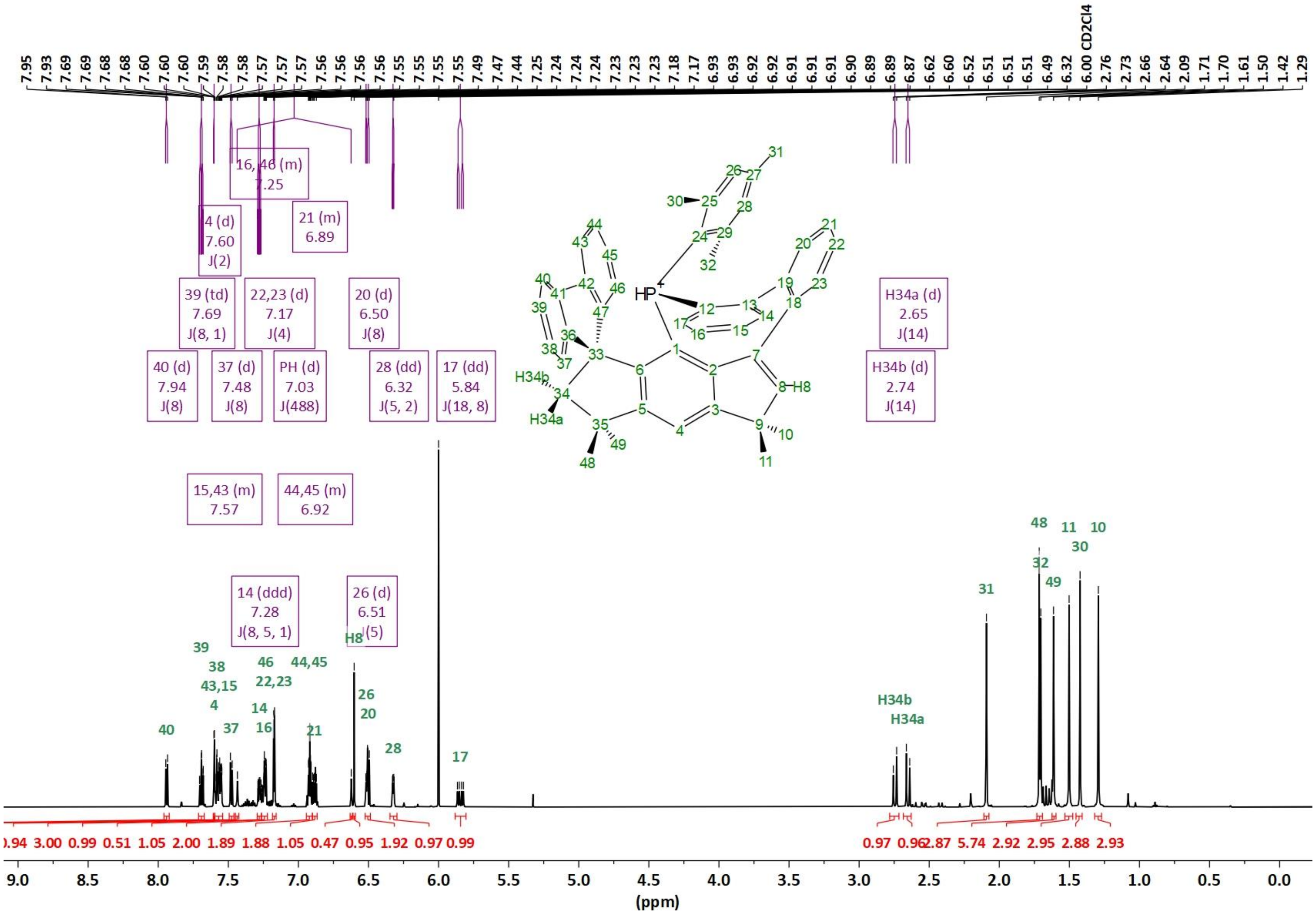

Figure S43.
^{1}H NMR (1,1,2,2-$C_2D_2Cl_4$, 600 MHz) spectrum of **9**.

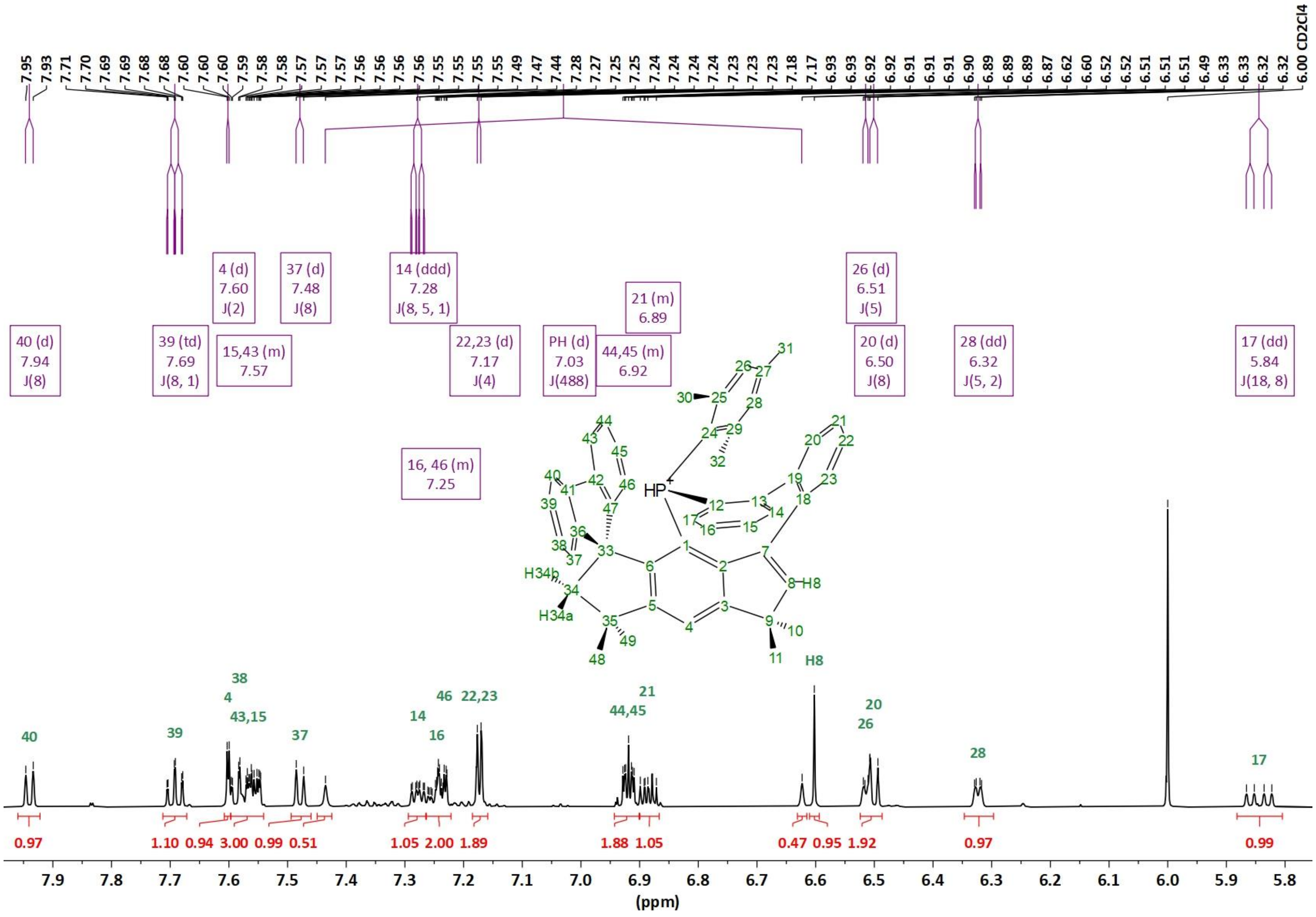

Figure S44.
Detailed ^{1}H NMR (1,1,2,2-$C_2D_2Cl_4$, 600 MHz) spectrum (aromatic area) of **9**.

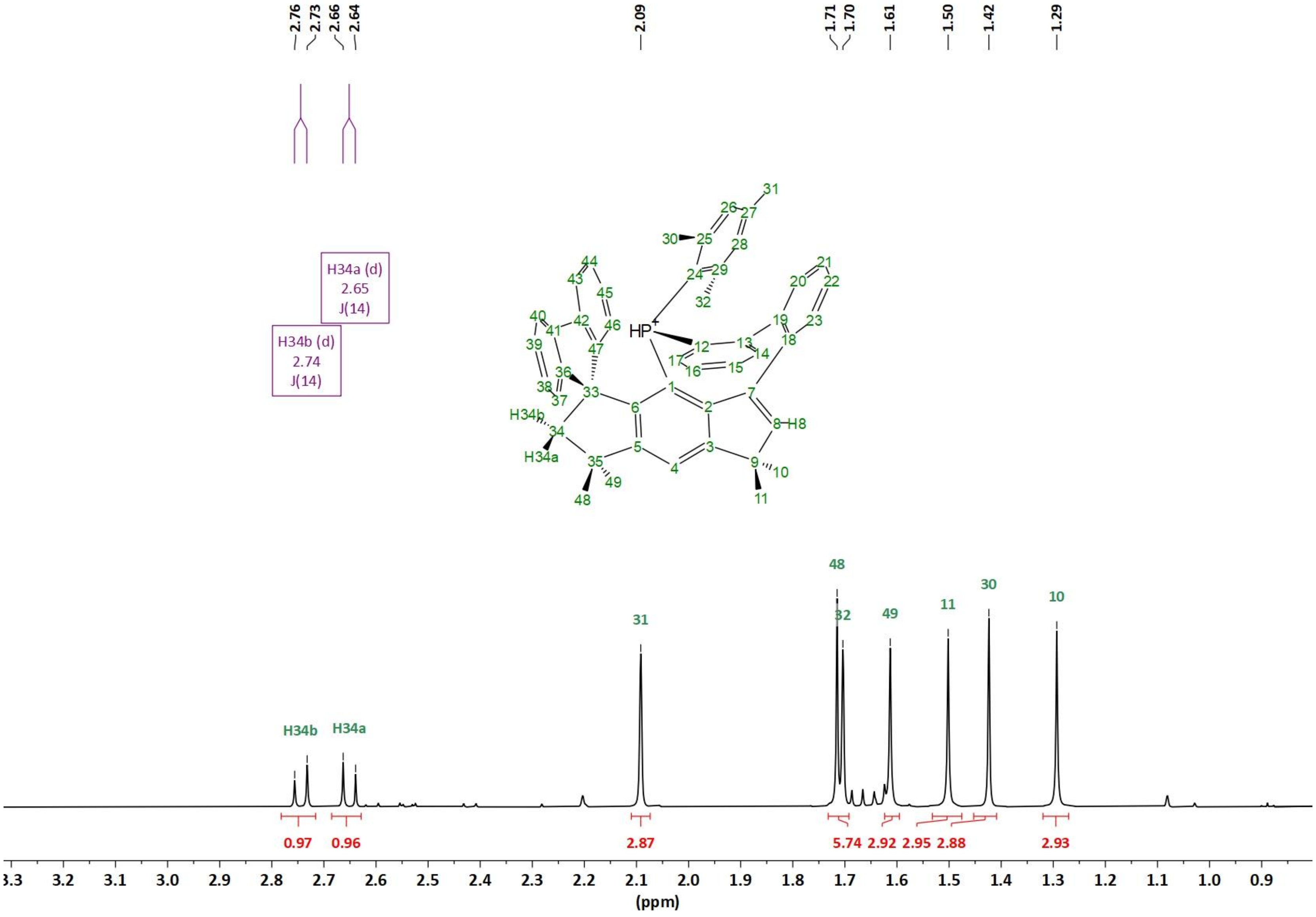

Figure S45.
Detailed ^{1}H NMR (1,1,2,2-$C_2D_2Cl_4$, 600 MHz) spectrum (aliphatic area) of **9**.

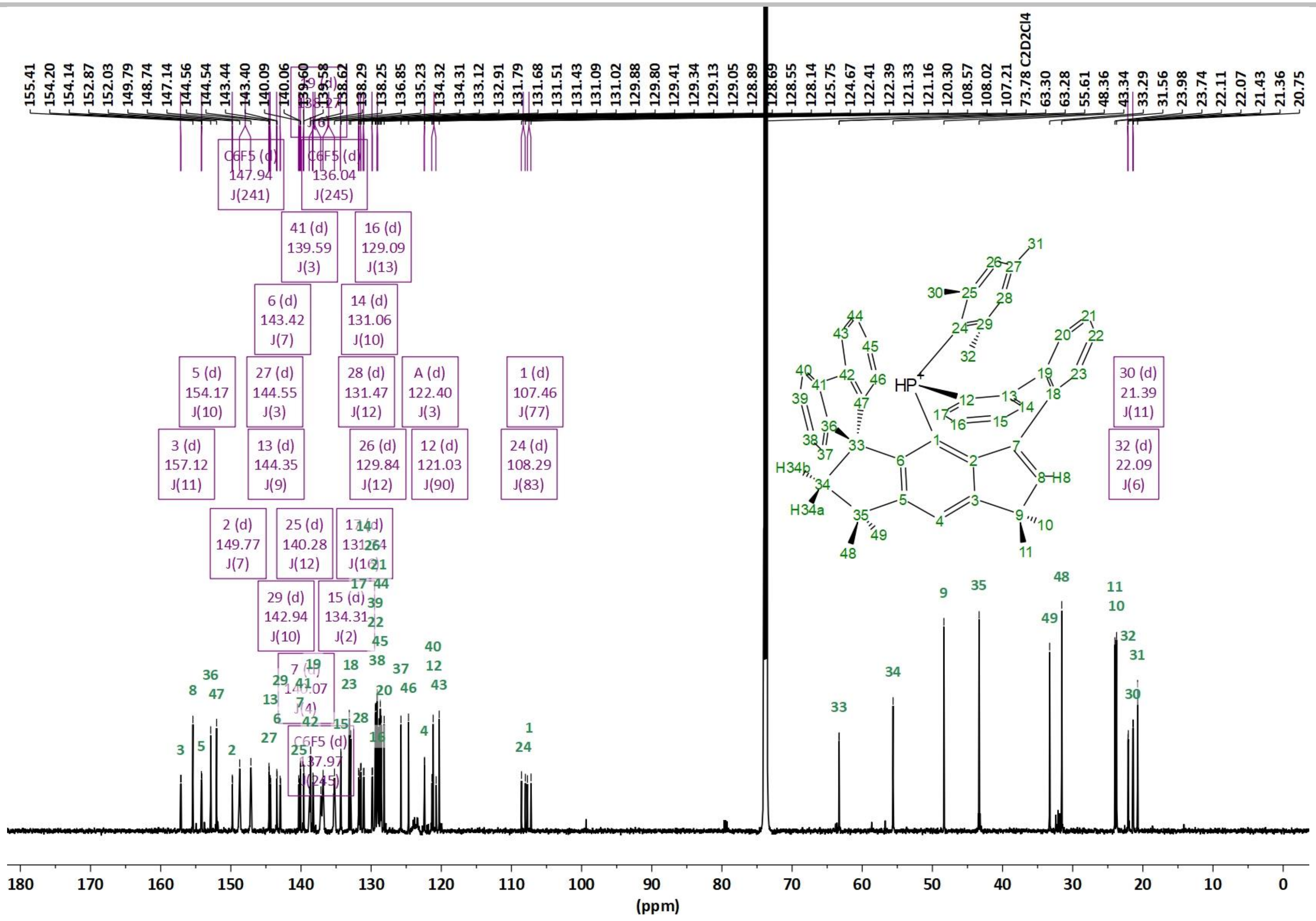

Figure S46.
$^{13}C\{^1H\}$ NMR (1,1,2,2-$C_2D_2Cl_4$, 151 MHz) spectrum of **9**.

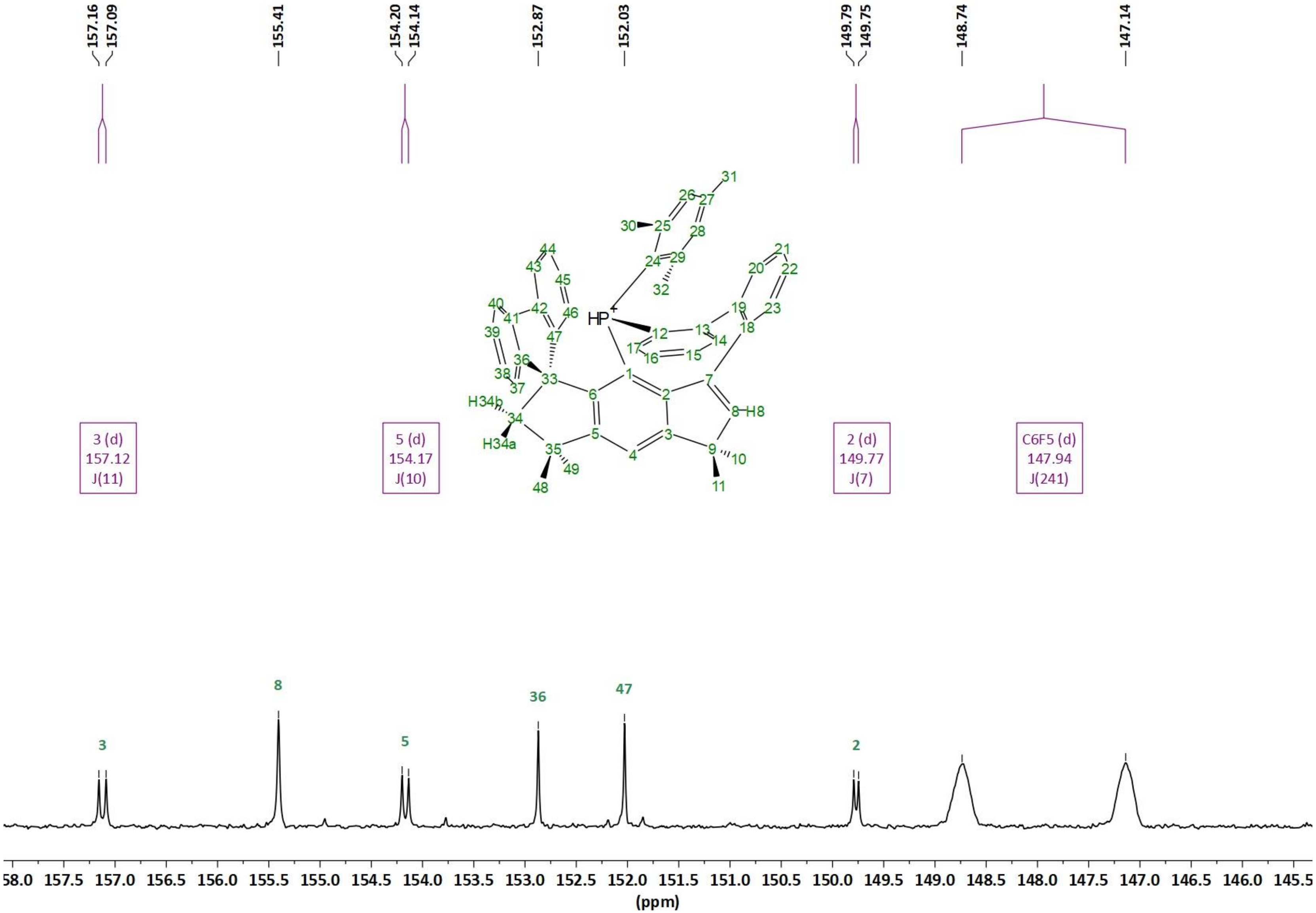

Figure S47.
Detailed $^{13}C\{^{1}H\}$ NMR (1,1,2,2-$C_2D_2Cl_4$, 151 MHz) spectrum (aromatic area) of **9**.

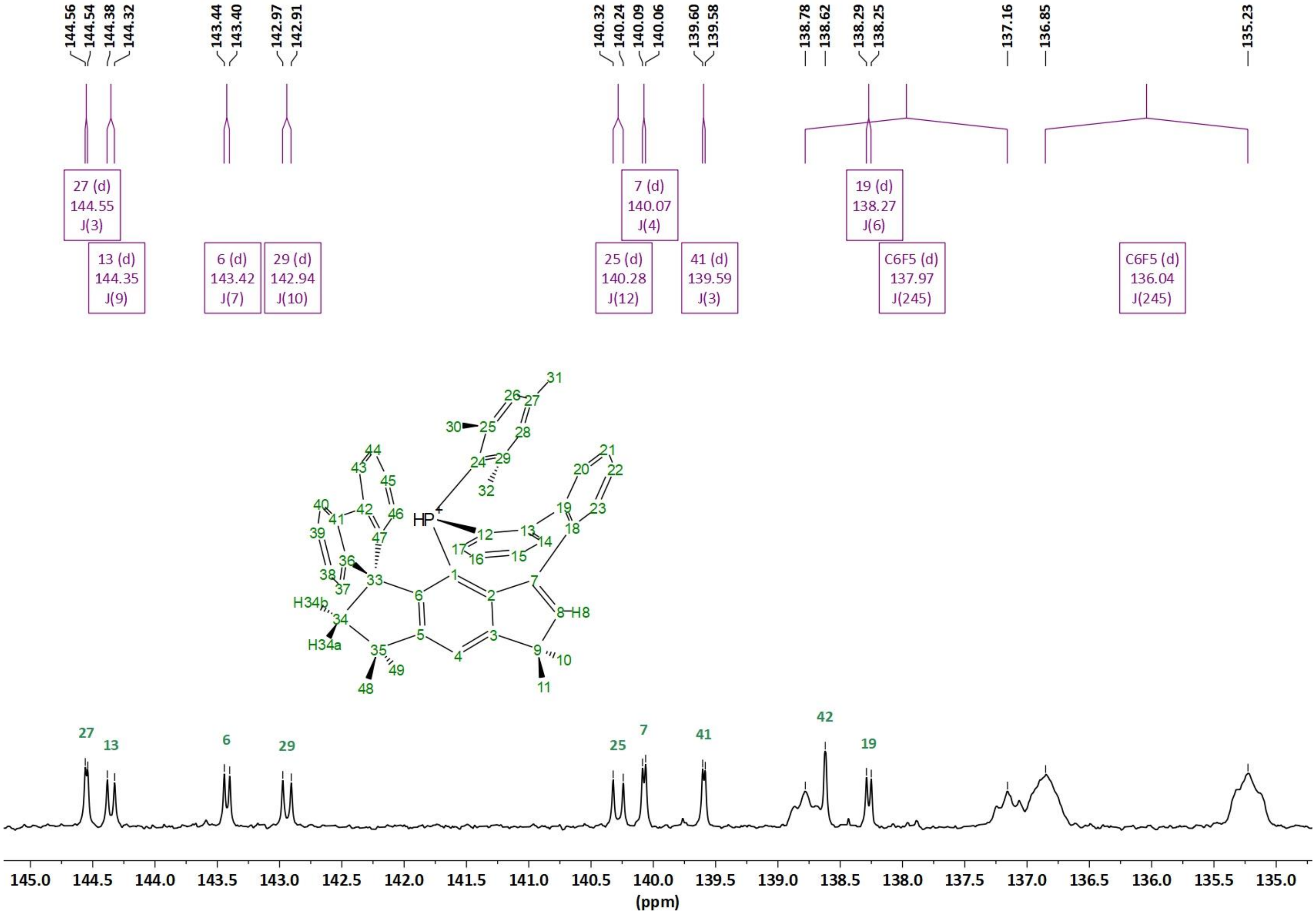

Figure S48.

Detailed $^{13}C\{^{1}H\}$ NMR (1,1,2,2-$C_2D_2Cl_4$, 151 MHz) spectrum (aromatic area) of **9**.

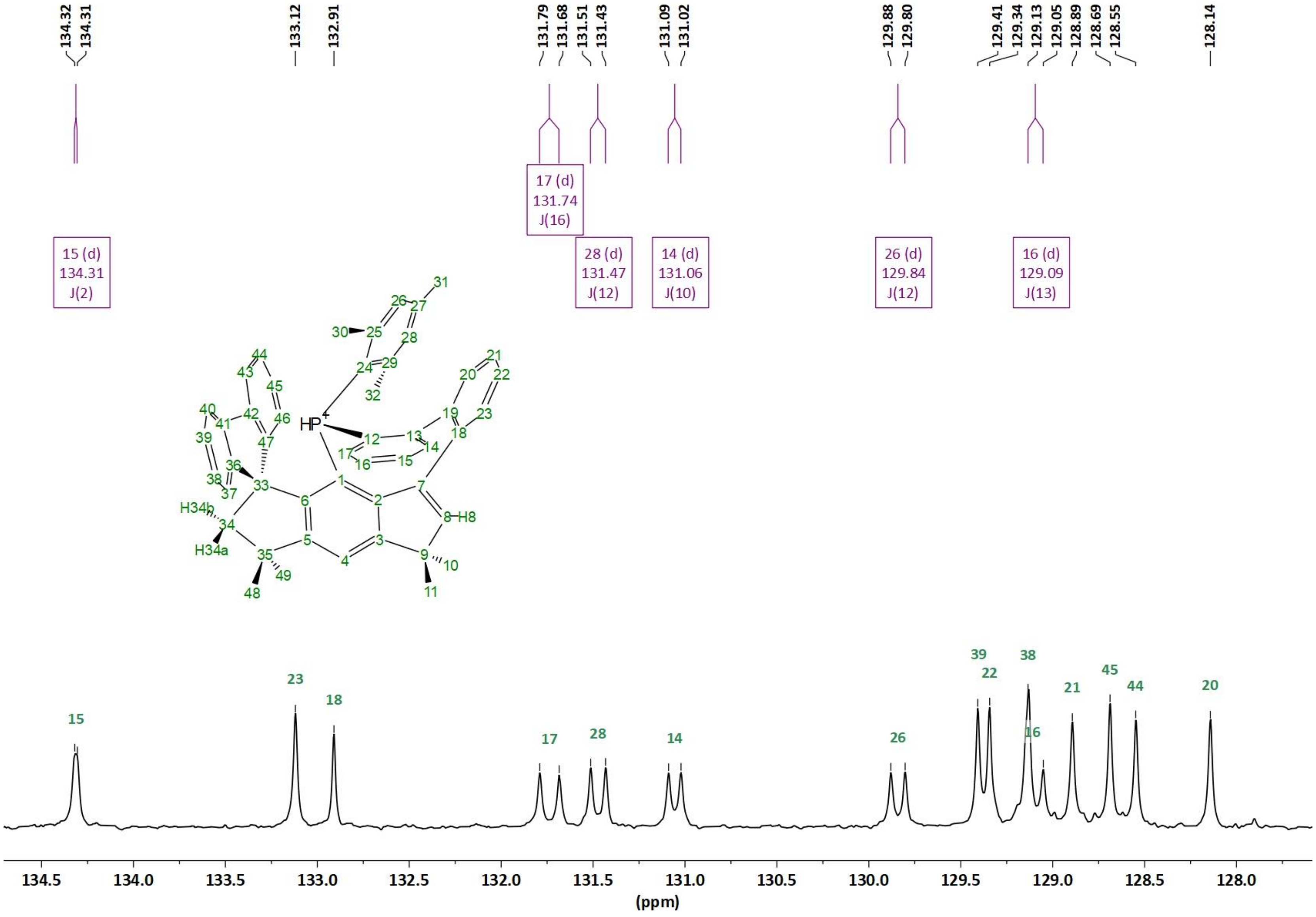

Figure S49.

Detailed $^{13}C\{^1H\}$ NMR (1,1,2,2-$C_2D_2Cl_4$, 151 MHz) spectrum (aromatic area) of **9**.

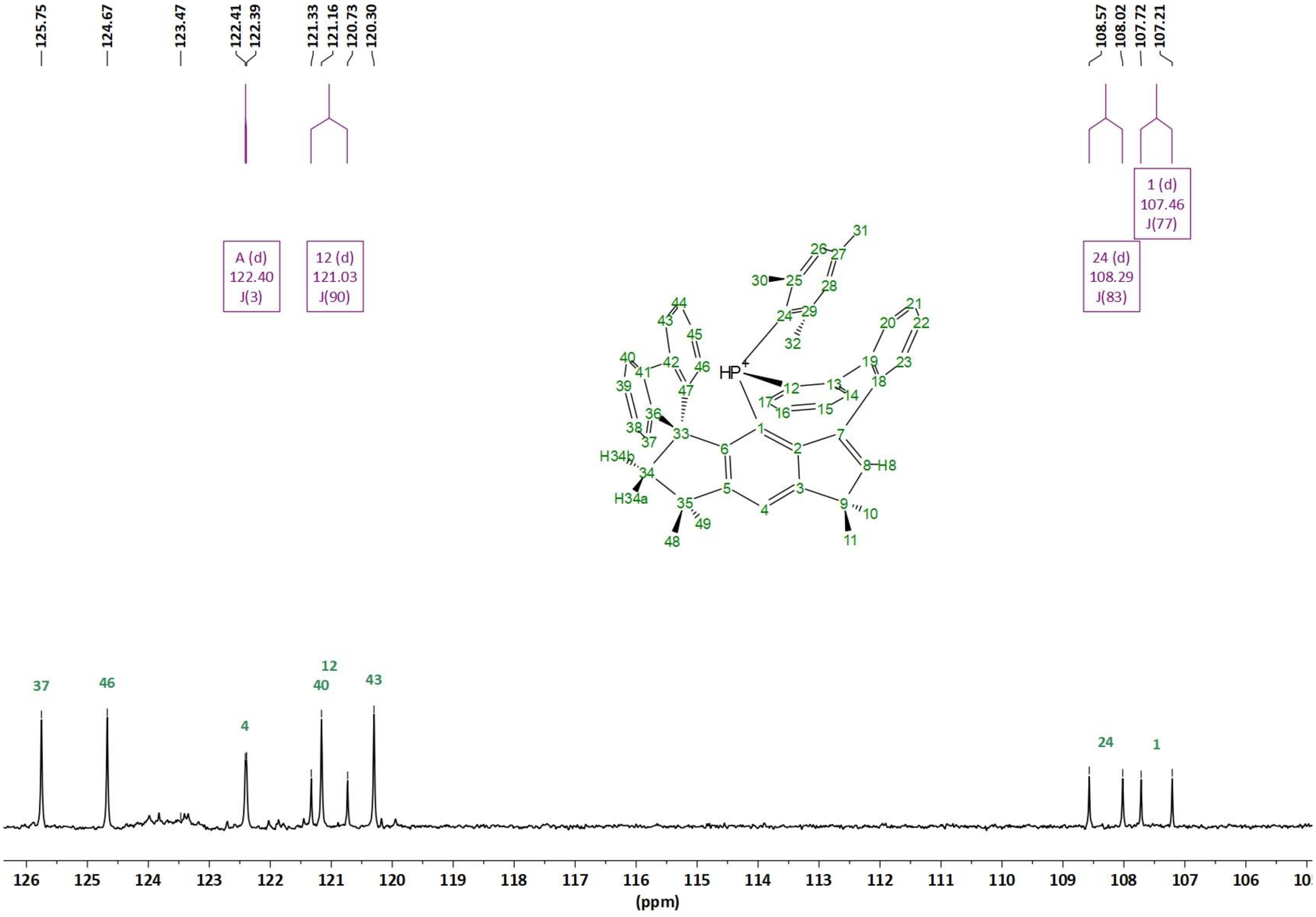

Figure S50.
Detailed $^{13}C\{^1H\}$ NMR (1,1,2,2-$C_2D_2Cl_4$, 151 MHz) spectrum (aromatic area) of **9**.

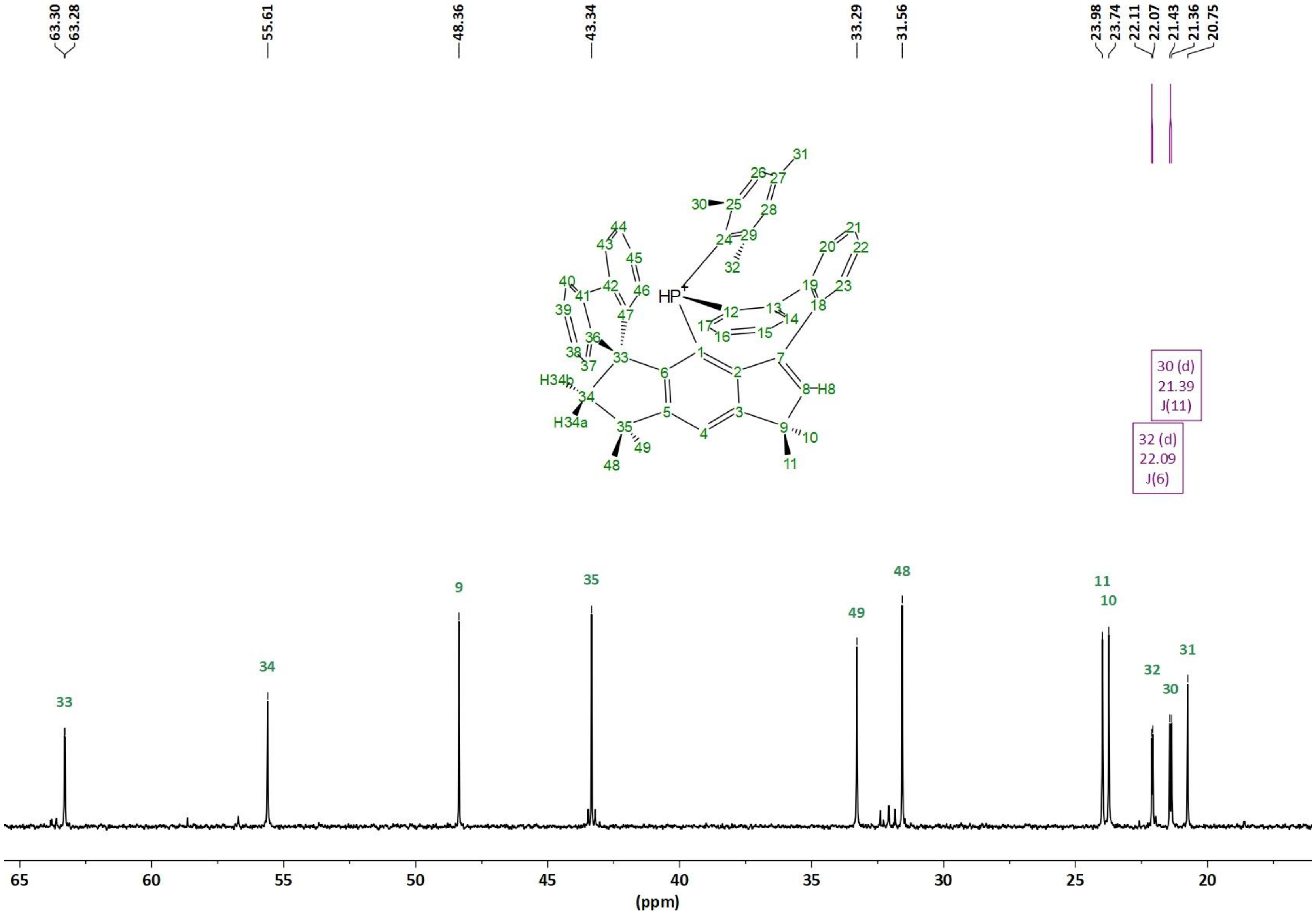

Figure S51.

Detailed $^{13}C\{^1H\}$ NMR (1,1,2,2-$C_2D_2Cl_4$, 151 MHz) spectrum (aliphatic area) of **9**.

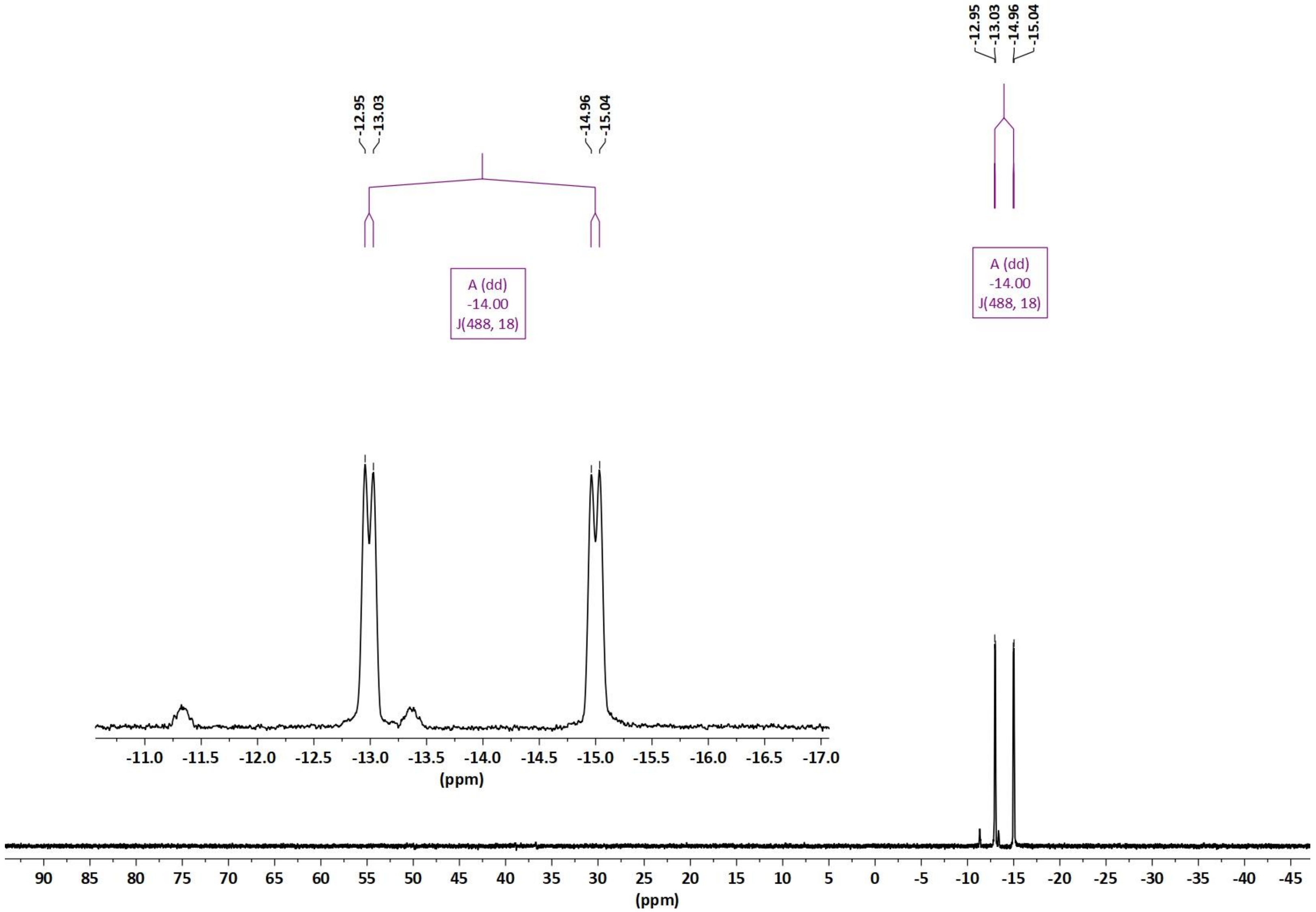

Figure S52.
^{31}P NMR (1,1,2,2-$C_2D_2Cl_4$, 243 MHz) spectrum of **9**.

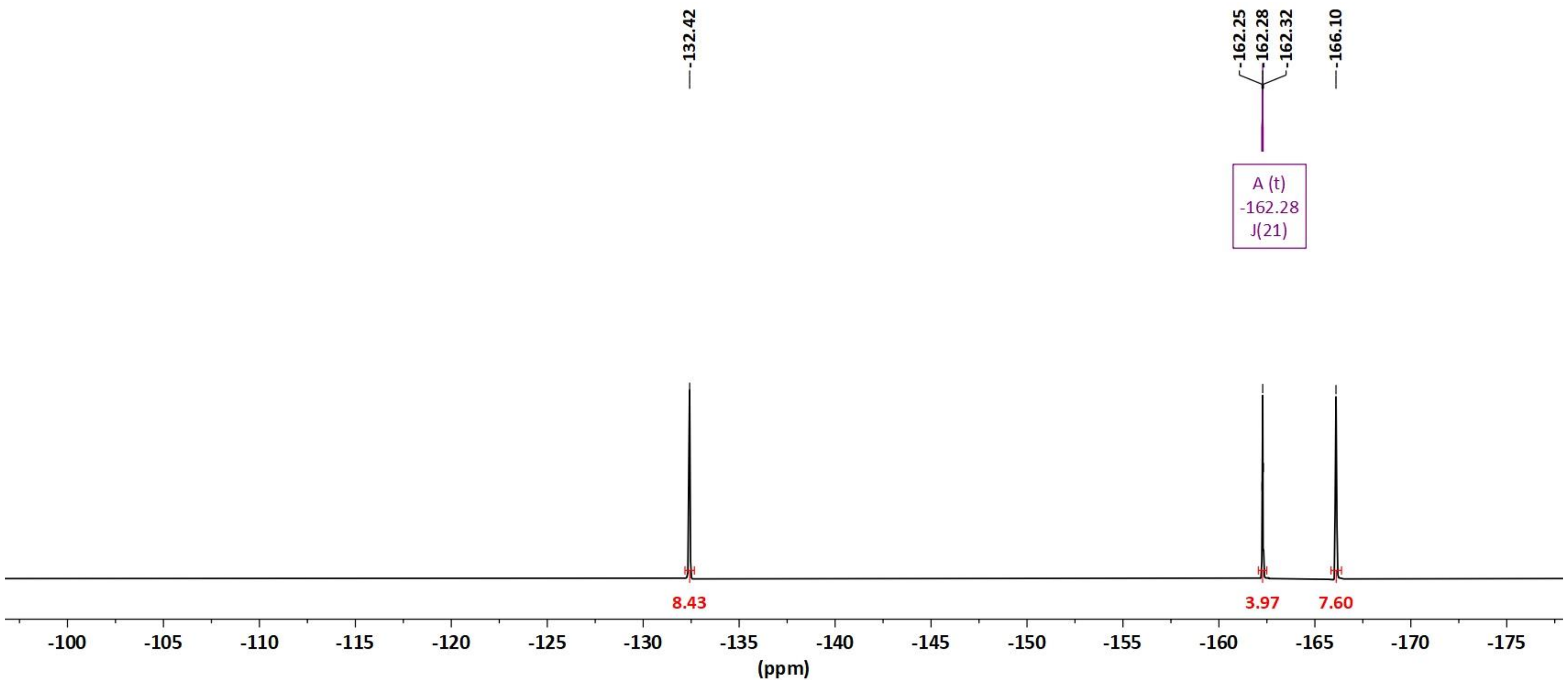

Figure S53.
^{19}F NMR (1,1,2,2-$C_2D_2Cl_4$, 565 MHz) spectrum of **9**.

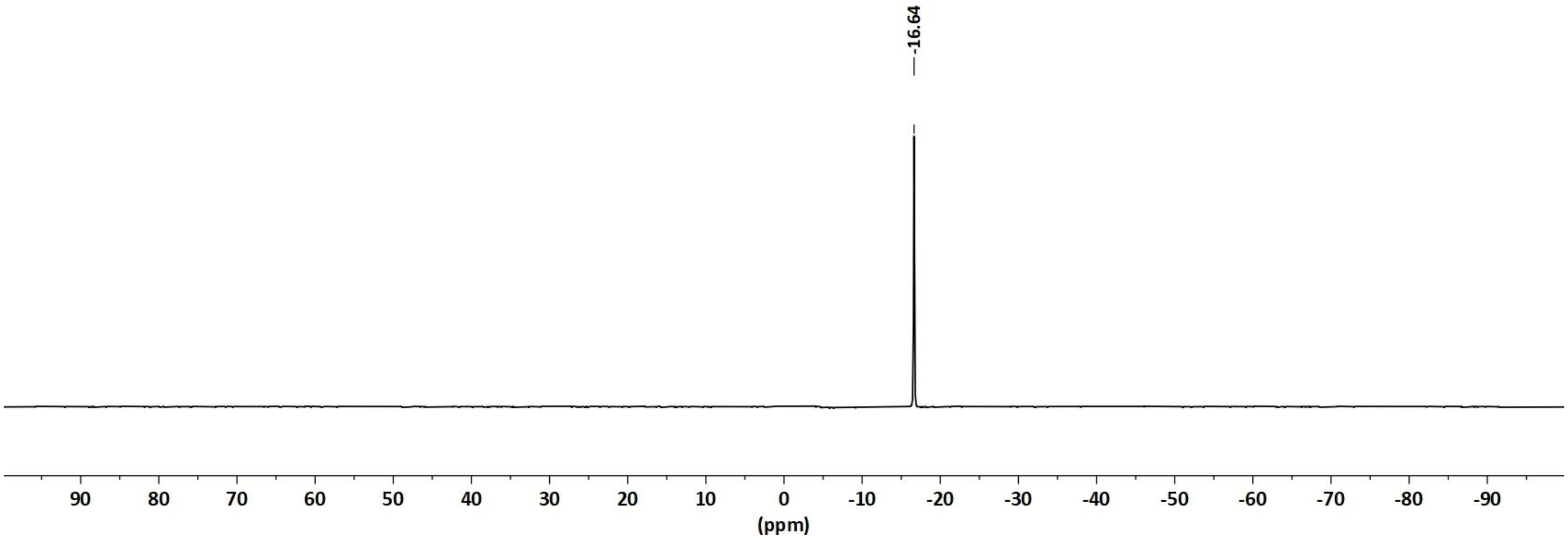

Figure S54.

^{11}B NMR (1,1,2,2-$C_2D_2Cl_4$, 193 MHz) spectrum of **9**.

Characterization of **10**

^{1}H NMR (600 MHz, CD_2Cl_2): δ = 7.91 (d, $^{3}J(^{1}H–^{1}H)$ = 8 Hz, 1H, H40), 7.67 (m, 2H, H15, H39), 7.65 (d, $^{5}J(^{1}H–^{31}P)$ = 2 Hz, 1H, H4), 7.56 (td, $^{3}J(^{1}H–^{1}H)$ = 8 Hz, $^{4}J(^{1}H–^{1}H)$ = 1 Hz, 1H, H38), 7.47 (dd, $^{3}J(^{1}H–^{1}H)$ = 8 Hz, $^{4}J(^{1}H–^{1}H)$ = 1 Hz, 1H, H43), 7.46 (d, $^{3}J(^{1}H–^{1}H)$ = 8 Hz, 1H, H37), 7.42 (ddd, $^{3}J(^{1}H–^{1}H)$ = 7 Hz, $^{4}J(^{1}H–^{1}H)$ = 3 Hz, $^{4}J(^{1}H–^{31}P)$ = 5 Hz, 1H, H14), 7.32 (tdd, $^{3}J(^{1}H–^{1}H)$ = 8 Hz, $^{4}J(^{1}H–^{31}P)$ = 3 Hz, $^{4}J(^{1}H–^{1}H)$ = 1 Hz, 1H, H16), 7.27 (m, overlapped with part of the P*H* doublet, 1H, H27), 7.14 (dt, $^{3}J(^{1}H–^{1}H)$ = 8 Hz, $^{4}J(^{1}H–^{1}H)$ = 1 Hz, 1H, H46), 7.06 (td, $^{3}J(^{1}H–^{1}H)$ = 8 Hz, $^{4}J(^{1}H–^{1}H)$ = 1 Hz, 1H, H22), 6.98 (m, 3H, H23, H26, H28), 6.86 (m, 1H, H44), 6.85 (d, $^{1}J(^{1}H–^{31}P)$ = 494 Hz, 1H, P*H*), 6.84 (m, 1H, H21), 6.77 (td, $^{3}J(^{1}H–^{1}H)$ = 8 Hz, $^{4}J(^{1}H–^{1}H)$ = 1 Hz, 1H, H45), 6.58 (d, $^{3}J(^{1}H–^{1}H)$ = 8 Hz, 1H, H29), 6.56 (d, $^{3}J(^{1}H–^{1}H)$ = 8 Hz, 1H, H25), 6.56 (s, overlapped with part of H25, 1H, H8), 6.52 (d, $^{3}J(^{1}H–^{1}H)$ = 8 Hz, 1H, H20), 6.06 (ddd, $^{3}J(^{1}H–^{31}P)$ = 18 Hz, $^{3}J(^{1}H–^{1}H)$ = 8 Hz, $^{4}J(^{1}H–^{1}H)$ = 1 Hz, 1H, H17), 2.69 (d, $^{2}J(^{1}H–^{1}H)$ = 14 Hz, 1H, H34b), 2.63 (d, $^{2}J(^{1}H–^{1}H)$ = 14 Hz, 1H, H34a), 1.71 (s, 3H, H48), 1.60 (s, 3H, H49), 1.56 (s, 3H, H11), 1.31 (s, 3H, H10). **$^{13}C\{^{1}H\}$ NMR (151 MHz, CD_2Cl_2)**: δ = 156.85 (d, $^{3}J(^{13}C–^{31}P)$ = 10 Hz, C3), 155.29 (s, C8), 154.28 (d, $^{3}J(^{13}C–^{31}P)$ = 10 Hz, C5), 152.79 (s, C36), 152.54 (s, C47), 150.20 (d, $^{2}J(^{13}C–^{31}P)$ = 7 Hz, C2), 147.94 (d, br, $^{1}J(^{13}C–^{19}F)$ = 241 Hz, C_6F_5), 145.75 (d, $^{2}J(^{13}C–^{31}P)$ = 9 Hz, C13), 145.55 (d, $^{2}J(^{13}C–^{31}P)$ = 7 Hz, C6), 139.69 (d, $^{3}J(^{13}C–^{31}P)$ = 4 Hz, C7), 139.47 (d, $^{5}J(^{13}C–^{31}P)$ = 3 Hz, C41), 138.33 (s, C42), 137.97 (dm, br, $^{1}J(^{13}C–^{19}F)$ = 245 Hz, C_6F_5), 137.77 (d, $^{3}J(^{13}C–^{31}P)$ = 5 Hz, C19), 136.04 (dm, br, $^{1}J(^{13}C–^{19}F)$ = 247 Hz, C_6F_5), 135.54 (s, C15), 134.01 (s, C18), 133.54 (d, $^{4}J(^{13}C–^{31}P)$ = 3 Hz, C27), 133.33 (d, $^{2}J(^{13}C–^{31}P)$ = 16 Hz, C17), 132.69 (s, C23), 131.40 (d, $^{3}J(^{13}C–^{31}P)$ = 10 Hz, C14), 131.09 (d, $^{2}J(^{13}C–^{31}P)$ = 13 Hz, C25, C29), 130.09 (s, C20), 129.80 (s, C22), 129.24 (s, C39), 129.16 (s, C21), 129.02 (s, C38), 128.98 (s, C45), 128.87 (d, $^{3}J(^{13}C–^{31}P)$ = 15 Hz, C16), 128.57 (s, C44), 128.25-128.15 (two d overlapped C28, C26), 125.35 (s, C37), 124.70 (s, br, *i*-C_6F_5), 124.94 (s, C46), 123.04 (s, C4), 121.49 (s, C40), 120.53 (s, C43), 117.22 (d, $^{1}J(^{13}C–^{31}P)$ = 89 Hz, C12), 114.25 (d, $^{1}J(^{13}C–^{31}P)$ = 86 Hz, C24), 102.03 (d, $^{1}J(^{13}C–^{31}P)$ = 82 Hz, C1), 63.16 (s, C33), 55.86 (s, C34), 48.45 (s, C9), 43.38 (s, C35), 32.93 (s, C49), 31.56 (s, C48), 23.97 (s, C10), 23.53 (s, C11). **^{31}P NMR (243 MHz, CD_2Cl_2)**: δ = −3.42 (dd, br, $^{1}J(^{31}P–^{1}H)$ = 494 Hz, $^{3}J(^{31}P–^{1}H)$ = 17 Hz). **^{19}F NMR (565 MHz, CD_2Cl_2)**: δ = −132.41 (br, 8F, *o*-C_6F_5), −162.26 (t, 4F, $^{3}J(^{19}F–^{19}F)$ = 21 Hz, *p*-C6F5), −166.09 (s, br, 8F, *m*-C_6F_5). **^{11}B NMR (193 MHz, CD_2Cl_2)**: δ = −16.65 (s). **HRMS ESI (m/z):** $[M]^{+}$ calculated. for $C_{46}H_{38}P$, 621.27056; found, 621.26948.

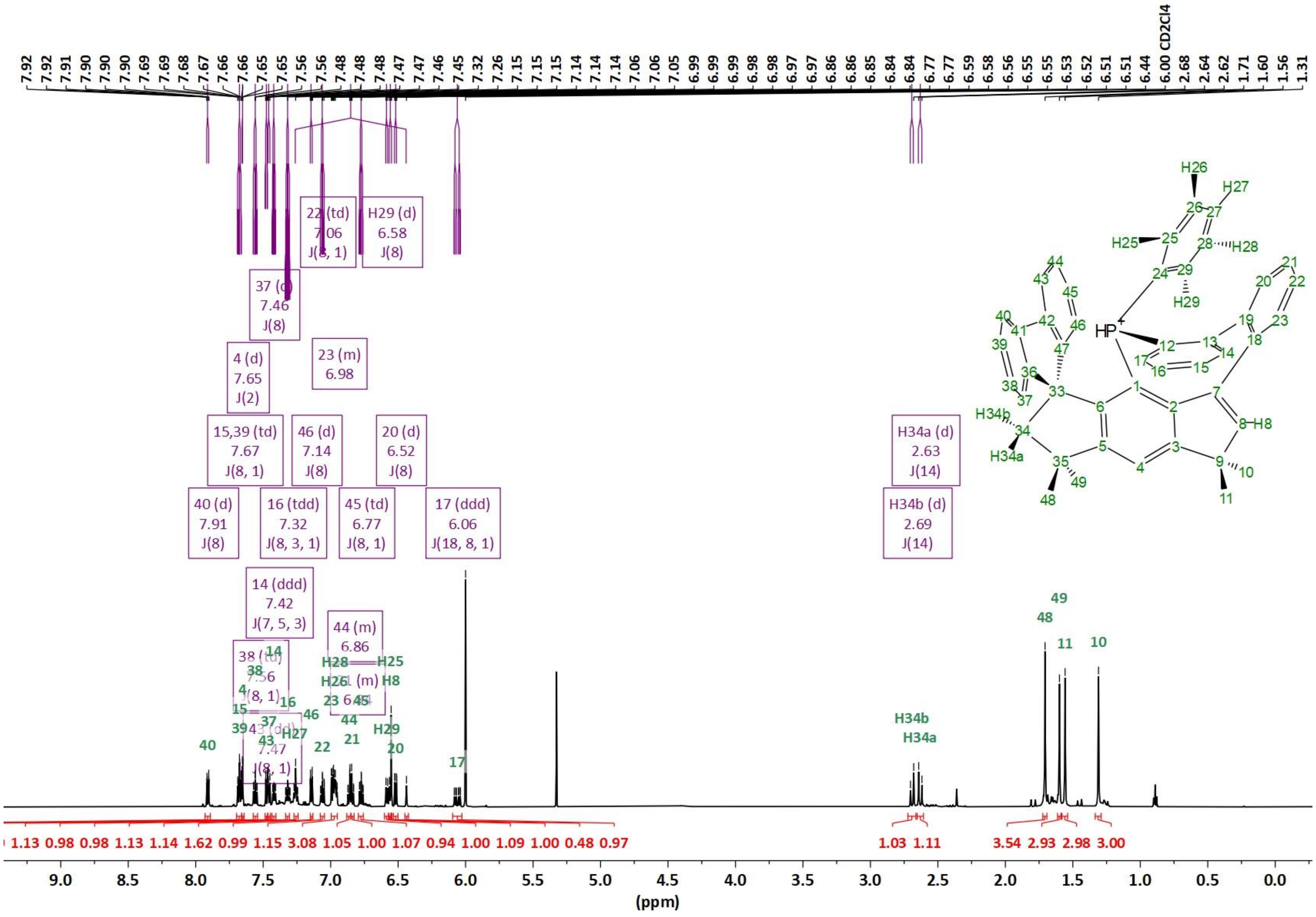

Figure S55.
^{1}H NMR (1,1,2,2-$C_2D_2Cl_4$, 600 MHz) spectrum of **10**.

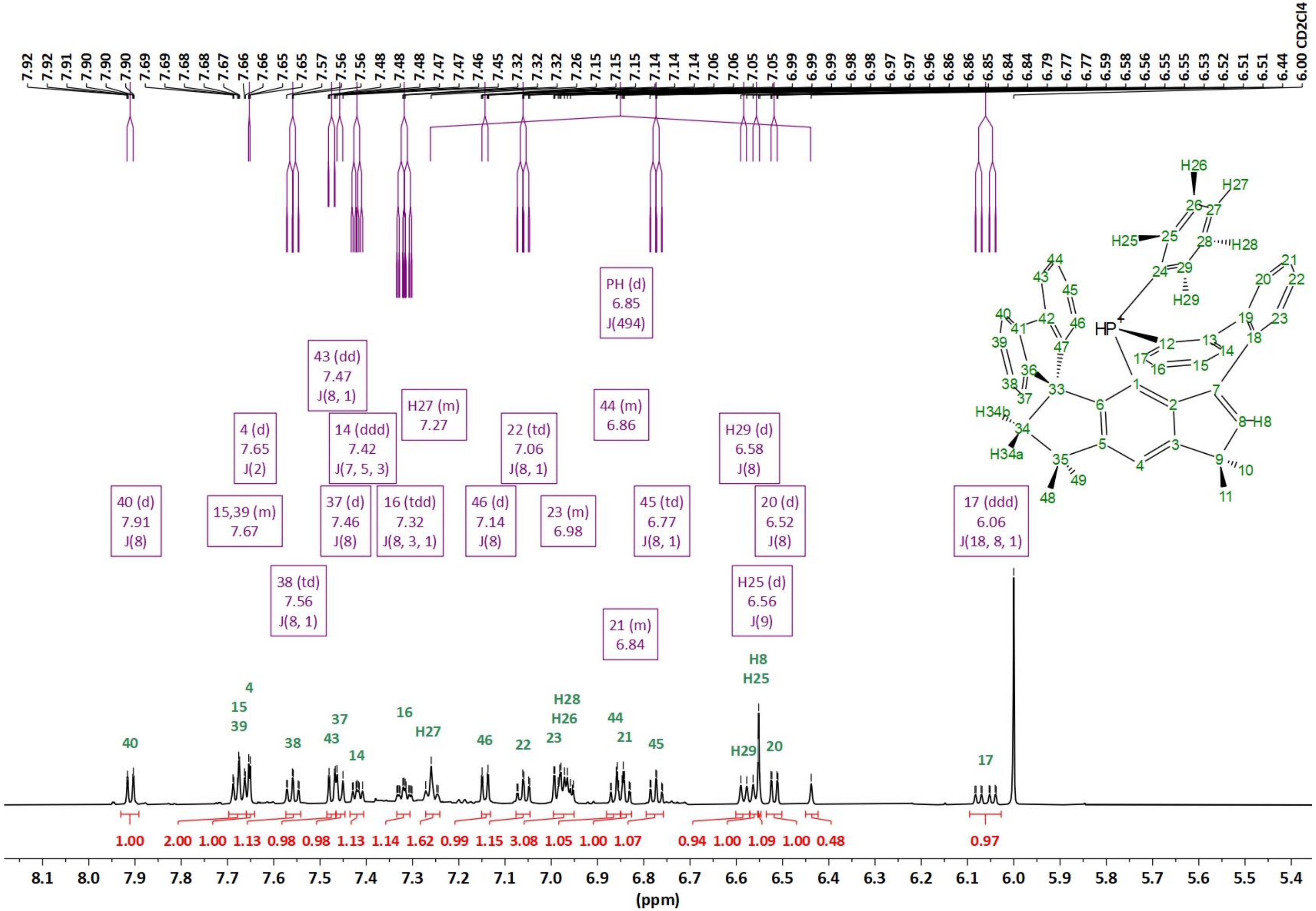

Figure S56.
Detailed ^{1}H NMR (1,1,2,2-$C_2D_2Cl_4$, 600 MHz) spectrum (aromatic area) of **10**.

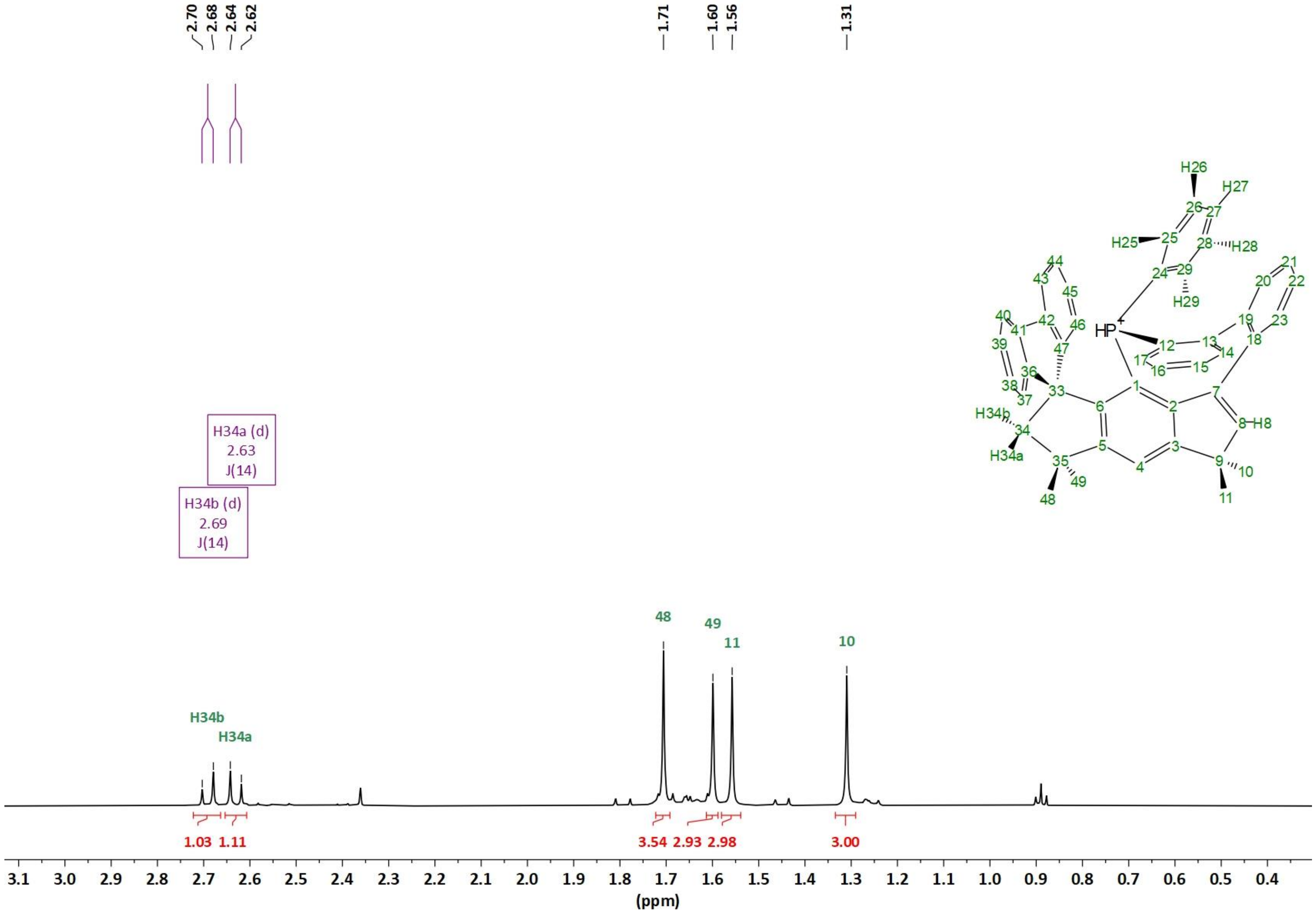

Figure S57.
Detailed ^{1}H NMR (1,1,2,2-$C_2D_2Cl_4$, 600 MHz) spectrum (aliphatic area) of **10**.

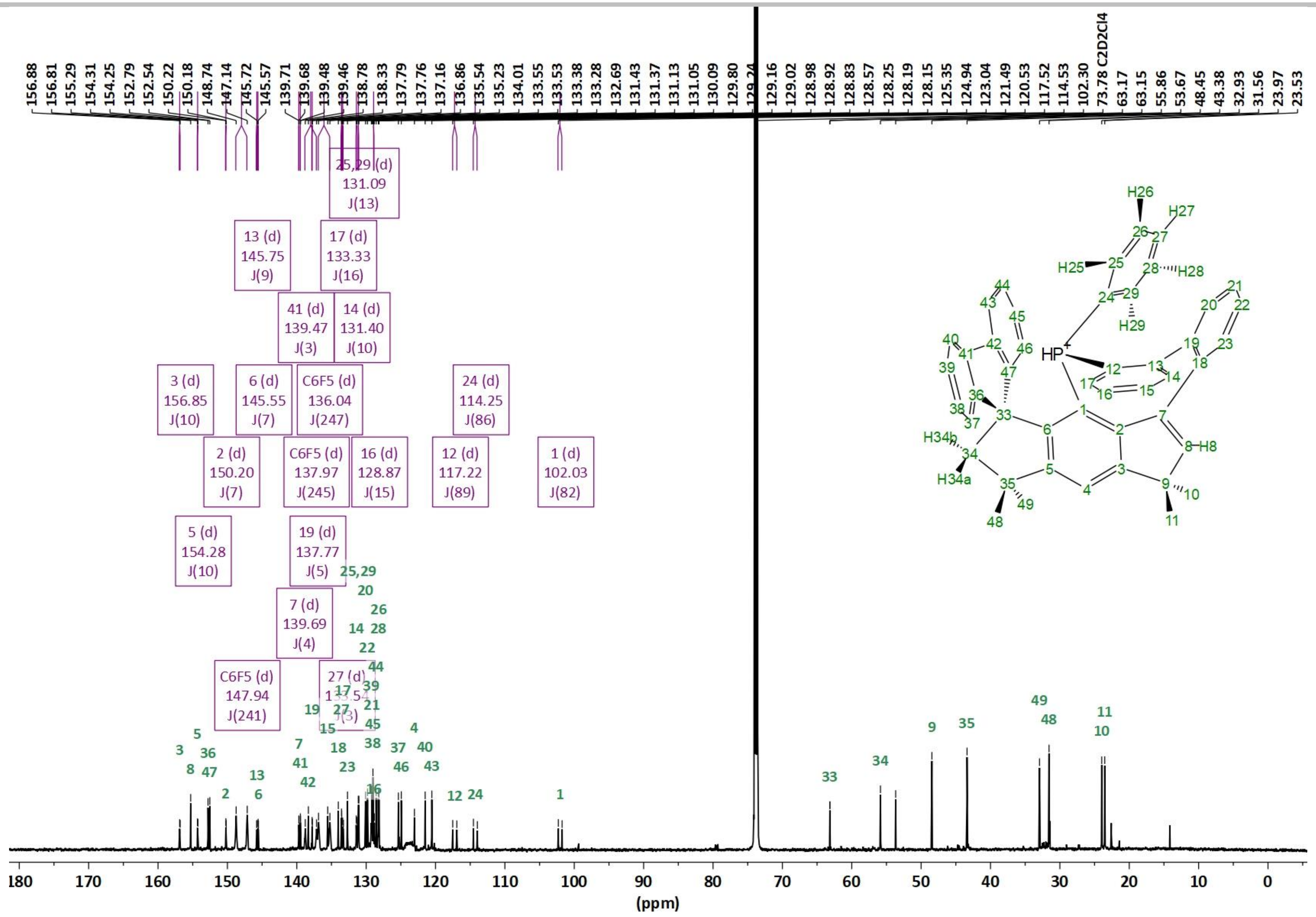

Figure S58.
$^{13}C\{^{1}H\}$ NMR (1,1,2,2-$C_2D_2Cl_4$, 151 MHz) spectrum of **10**.

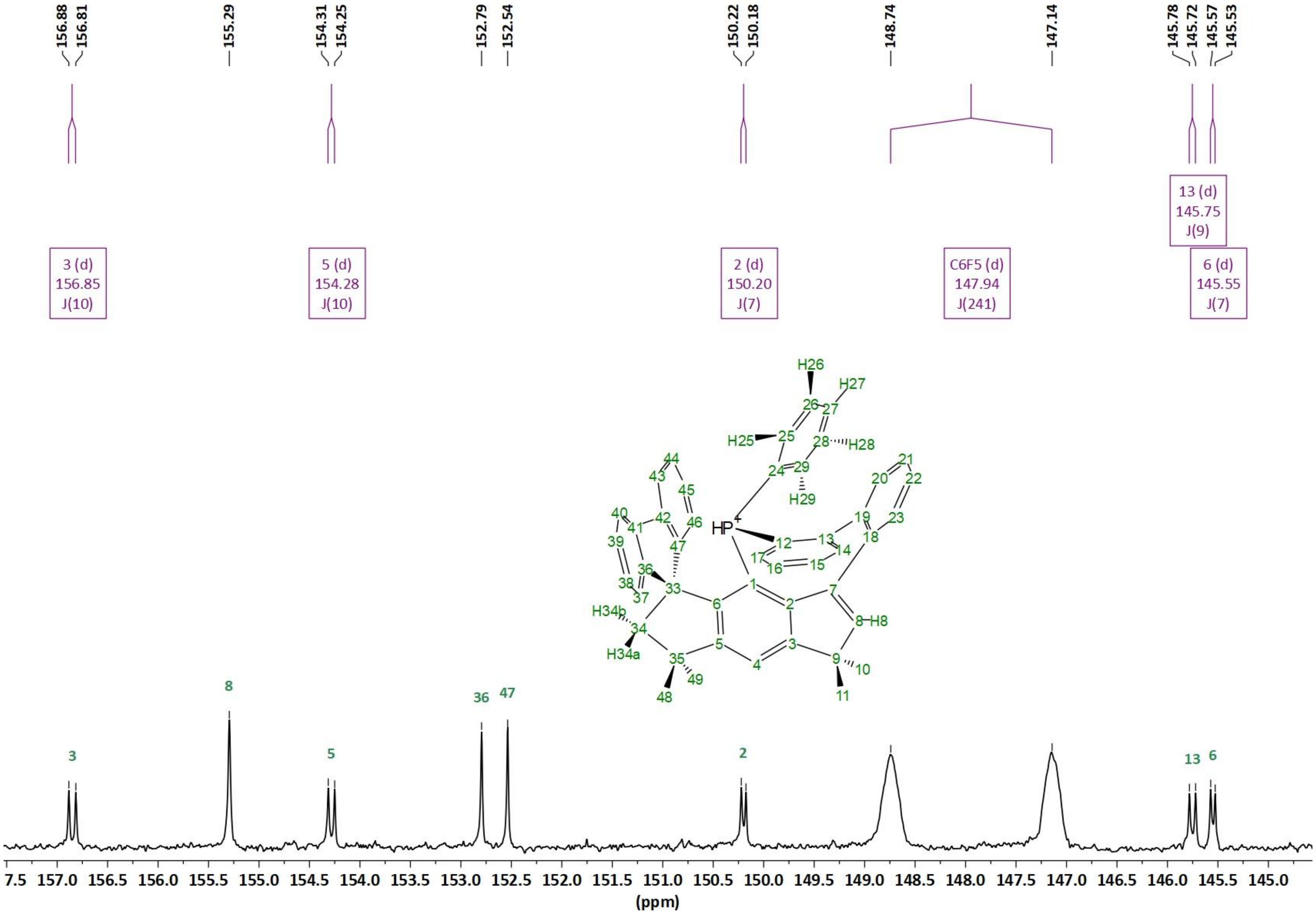

Figure S59.
Detailed $^{13}C\{^{1}H\}$ NMR (1,1,2,2-$C_2D_2Cl_4$, 151 MHz) spectrum (aromatic area) of **10**.

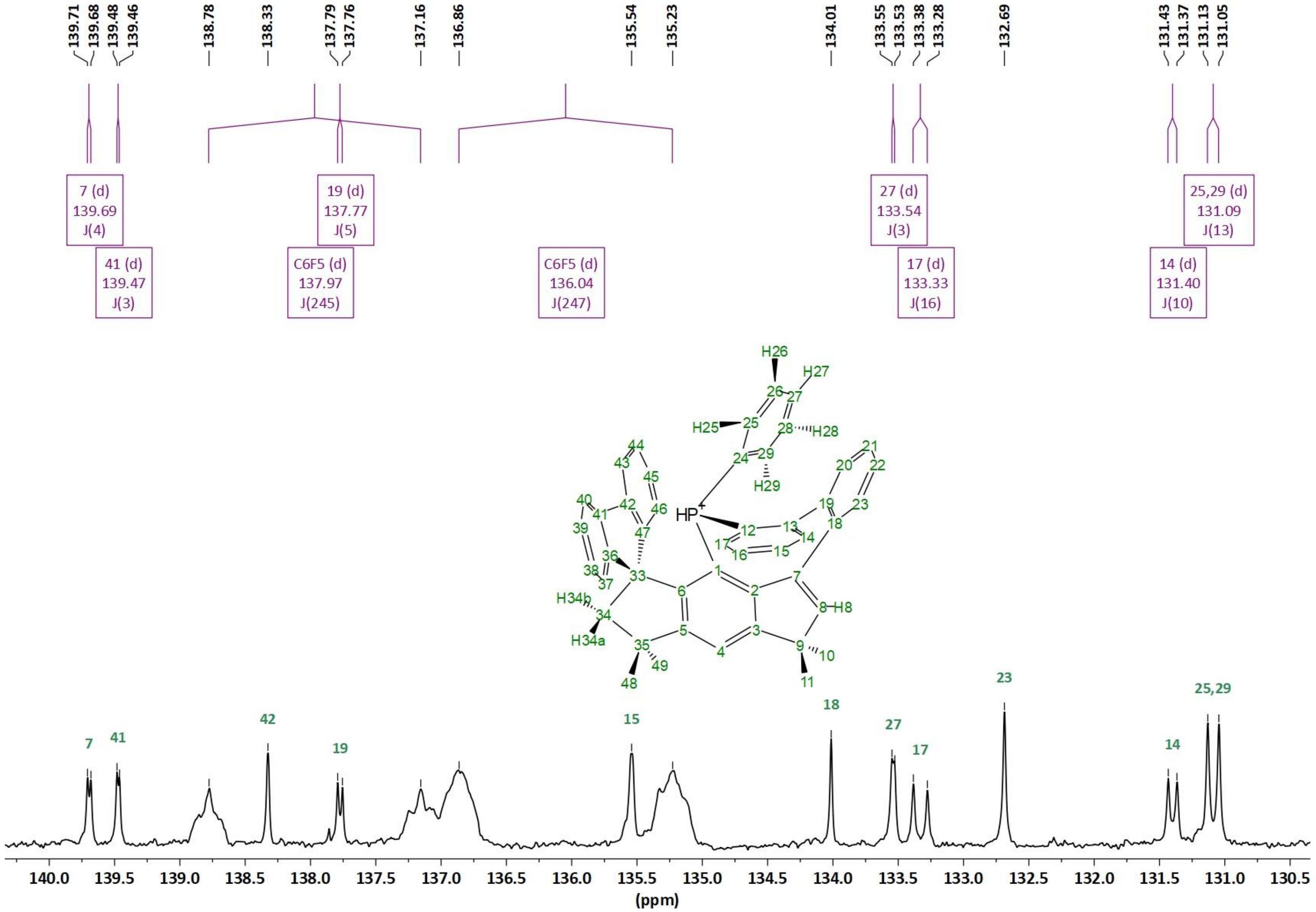

Figure S60.
Detailed $^{13}C\{^{1}H\}$ NMR (1,1,2,2-$C_2D_2Cl_4$, 151 MHz) spectrum (aromatic area) of **10**.

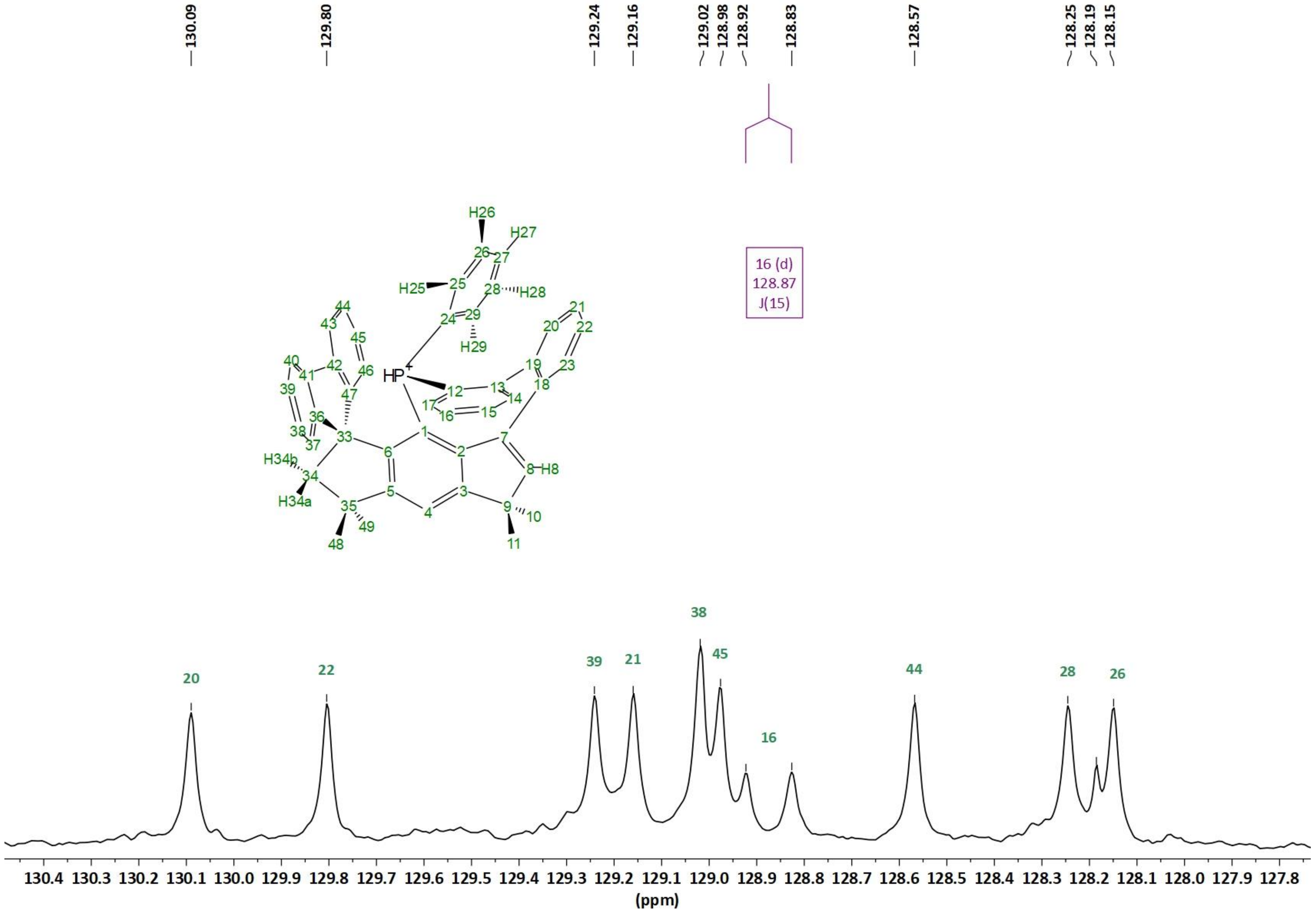

Figure S61.
Detailed $^{13}C\{^{1}H\}$ NMR (1,1,2,2-$C_2D_2Cl_4$, 151 MHz) spectrum (aromatic area) of **10**.

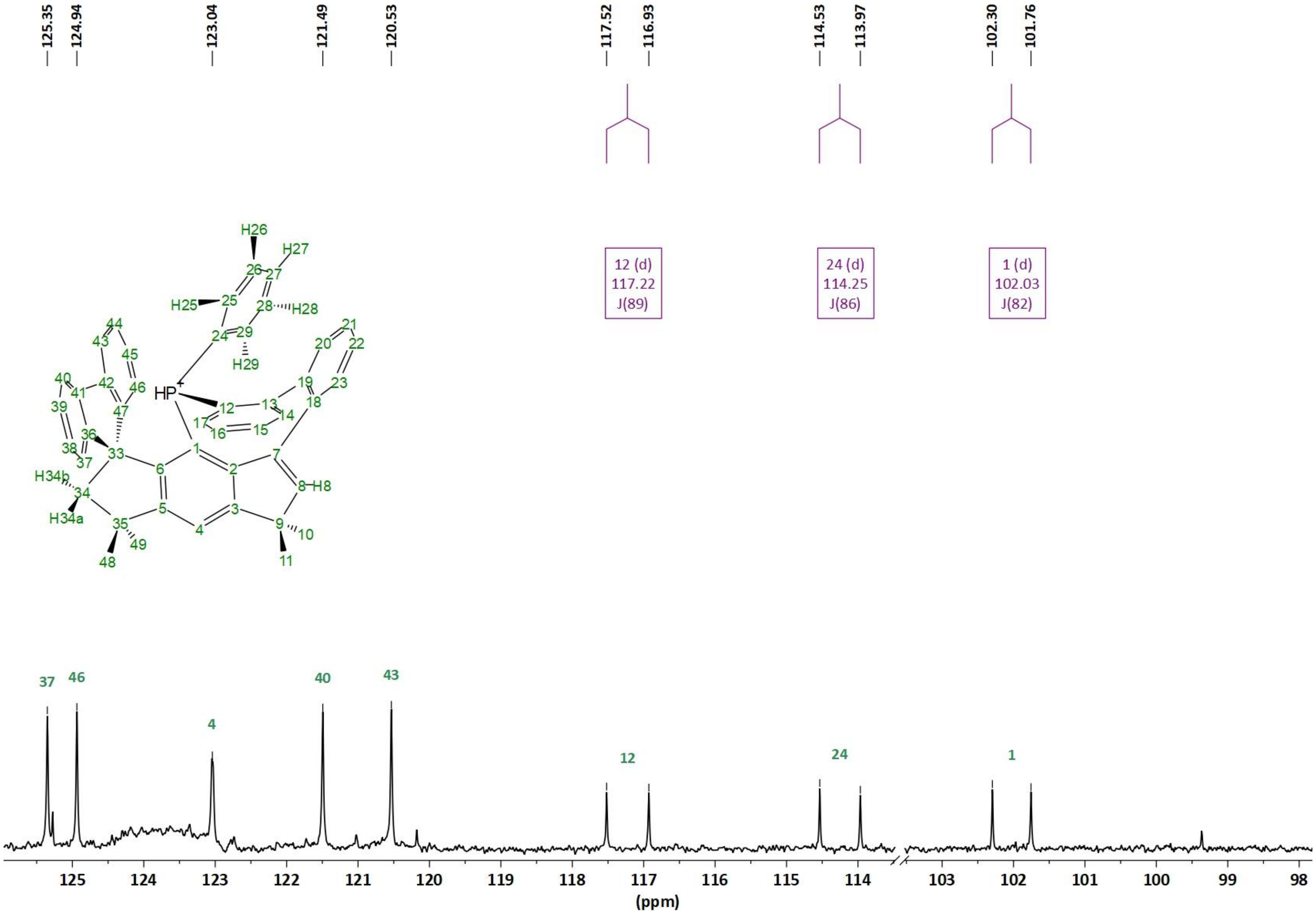

Figure S62.
Detailed $^{13}C\{^{1}H\}$ NMR (1,1,2,2-$C_2D_2Cl_4$, 151 MHz) spectrum (aromatic area) of **10**.

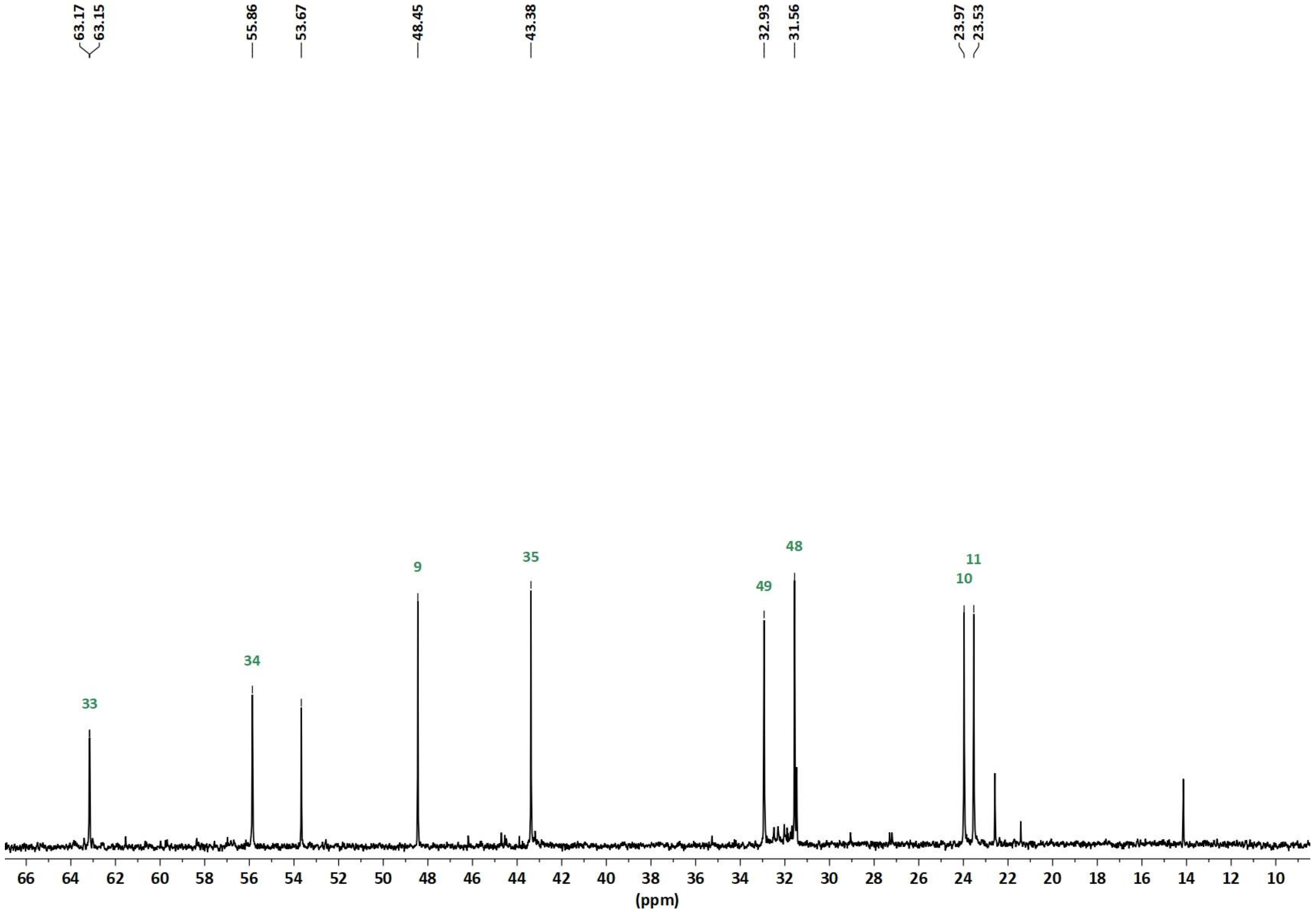

Figure S63.
Detailed $^{13}C\{^{1}H\}$ NMR (1,1,2,2-$C_2D_2Cl_4$, 151 MHz) spectrum (aliphatic area) of **10**.

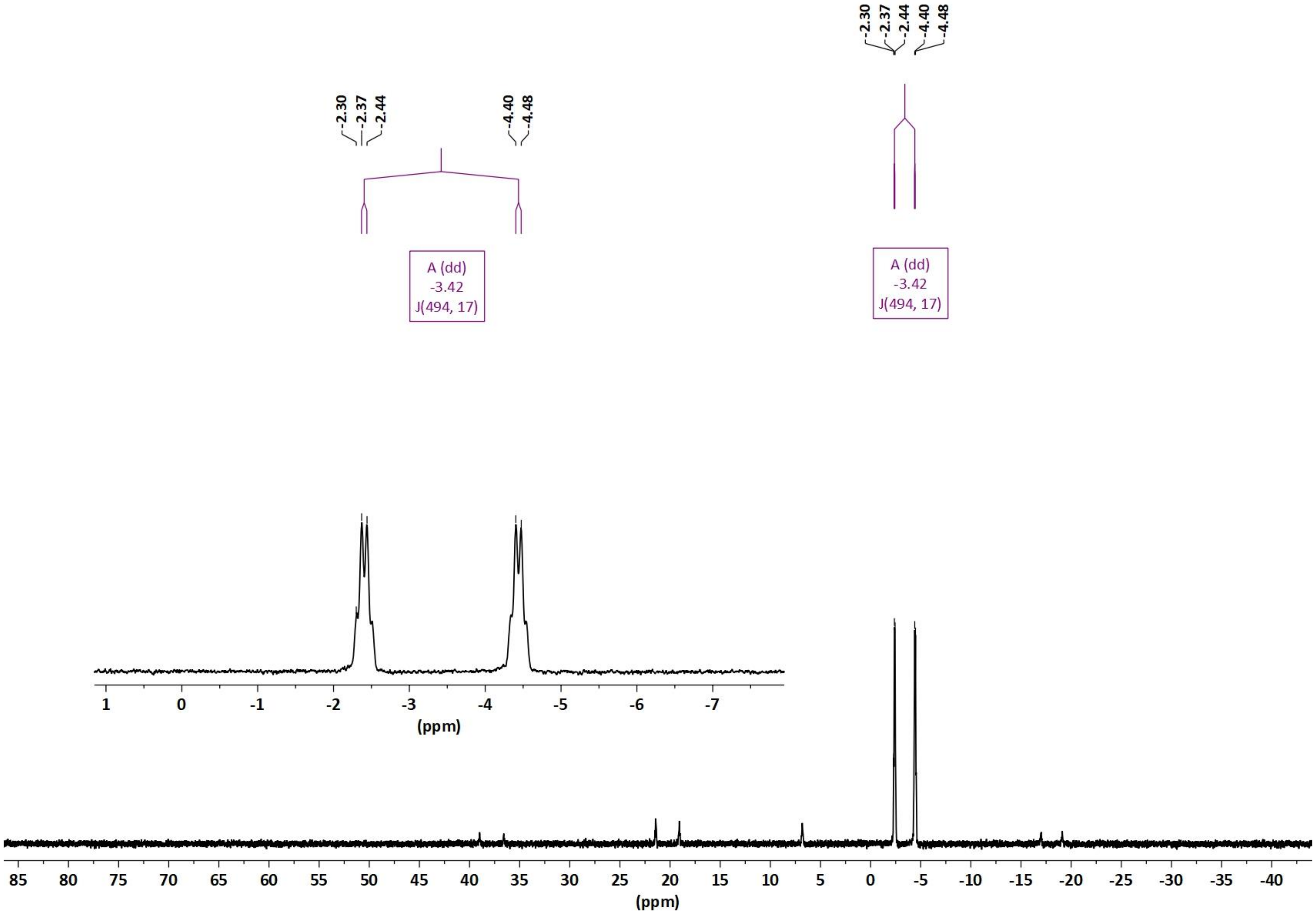

Figure S64.
^{31}P NMR (1,1,2,2-$C_2D_2Cl_4$, 243 MHz) spectrum of **10**.

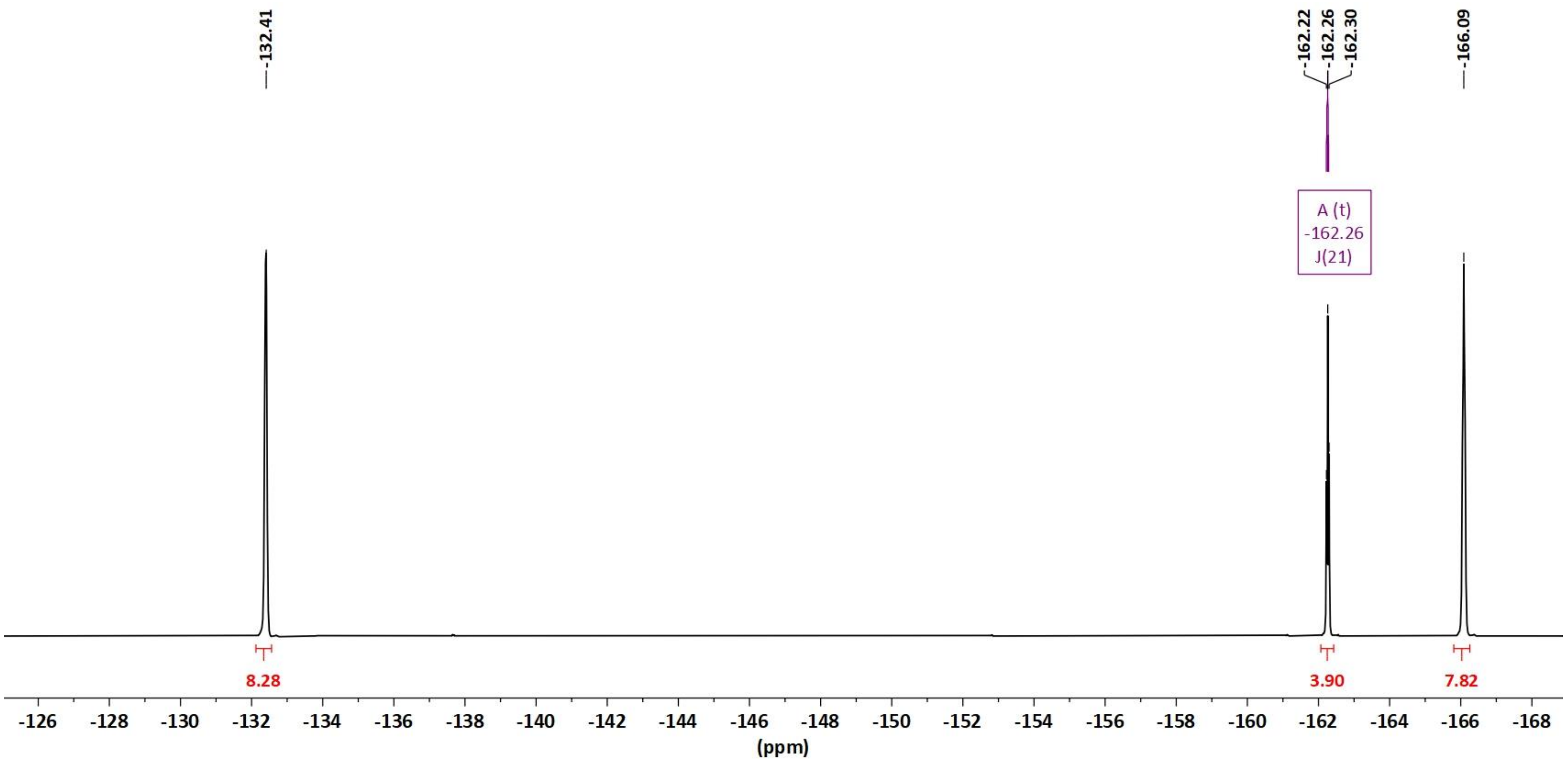

Figure S65.
^{19}F NMR (1,1,2,2-$C_2D_2Cl_4$, 565 MHz) spectrum of **10**.

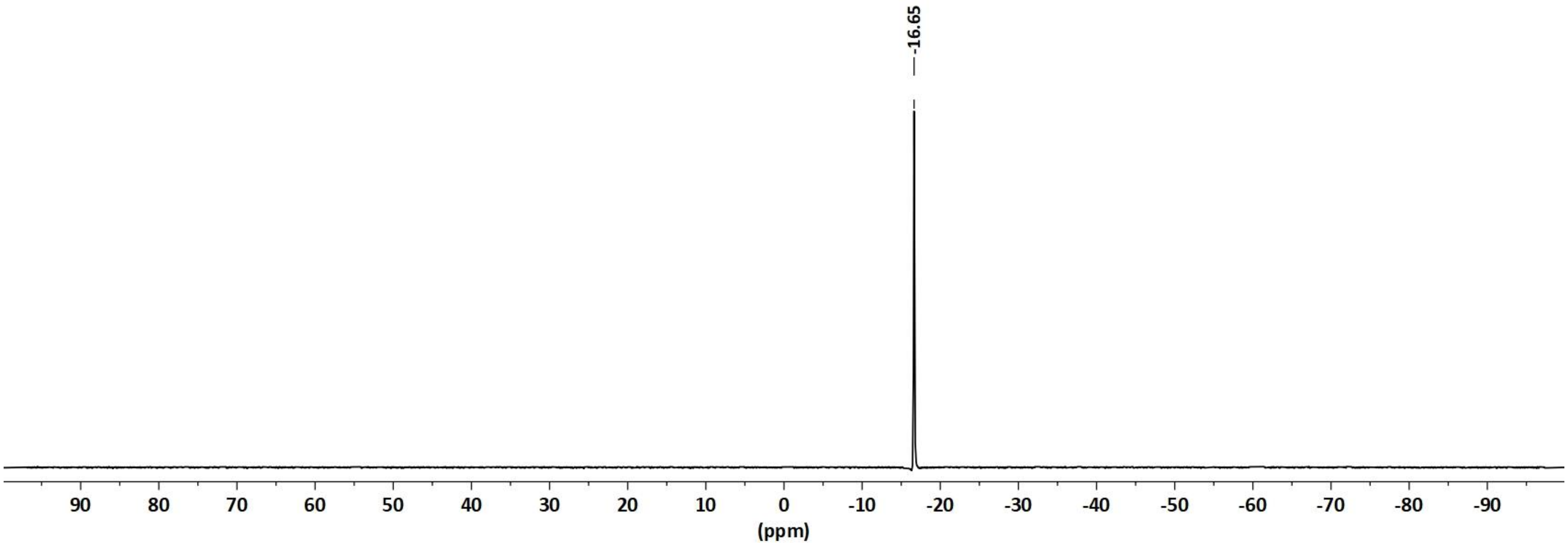

Figure S66.
^{11}B NMR (1,1,2,2-$C_2D_2Cl_4$, 193 MHz) spectrum of **10**.

Reactivity towards water

When solutions of **6** or **8** (in CD_2Cl_2) were exposed to moisture, a quick discoloration of the solutions was observed as $[Ar^1Ar^2P(H)OH][B(C_6F_5)_4]$ (**11**, Ar^2 = Mes; **12** Ar^2 = Ph) formed. Compound **7** reacted much slower (incomplete reaction after 7 days at rt) and gave a mixture of products.

Characterization of **11**

^{1}H NMR (600 MHz, CD_2Cl_2): δ = 7.91 (d, $^5J(^{31}P–^1H)$ = 2 Hz, 1H, H4), 7.60 (d, $^3J(^1H–^1H)$ = 8 Hz, 2H, H21), 7.47 (d, $^3J(^1H–^1H)$ = 7 Hz, 2H, H14), 7.35 (m, 2H, H20), 7.29 (m, 8H, H12, H13, H18, H19), 7.23 (d, $^3J(^1H–^1H)$ = 7 Hz, 2H, H11), 6.92 (d, $^1J(^{31}P–^1H)$ = 540 Hz, 1H, P*H*), 6.19 (d, $^3J(^{31}P–^1H)$ = 6 Hz, 2H, H24, H26), 2.49 (d, $^2J(^1H–^1H)$ = 14 Hz, 2H, H6a), 2.40 (d, $^2J(^1H–^1H)$ = 14 Hz, 2H, H6b), 1.63 (s, 6H, H9), 1.62 (s, 6H, H8), 1.10 (s, 6H, H28, H29). **^{13}C{^{1}H} NMR (151 MHz, CD_2Cl_2)**: δ = 161.03 (d, $^3J(^{13}C–^{31}P)$ = 11 Hz, C3), 153.25 (s, C10), 152.88 (s, C17), 148.70 (d, br, $^1J(^{13}C–^{19}F)$ = 242 Hz, *C*$_6$F$_5$), 147.57 (d, $^2J(^{13}C–^{31}P)$ = 9 Hz, C2), 147.29 (d, $^4J(^{13}C–^{31}P)$ = 3 Hz, C25), 144.27 (d, $^2J(^{13}C–^{31}P)$ = 14 Hz, C23, C27), 139.60 (s, C16), 139.57 (s, C15), 138.79 (d, br, $^1J(^{13}C–^{19}F)$ = 247 Hz, *C*$_6$F$_5$), 136.86 (d, br, $^1J(^{13}C–^{19}F)$ = 247 Hz, *C*$_6$F$_5$), 131.34 (d, $^3J(^{13}C–^{31}P)$ = 14 Hz, C24, C26), 129.61 (s, C20), 129.53 (s, C19), 129.10 (s, C12), 128.90 (s, C13), 128.45 (s, C4), 125.16 (s, C18), 123.64 (s, C11), 122.40 (s, C21), 122.33 (s, C14), 117.39 (d, $^1J(^{13}C–^{31}P)$ = 86 Hz, C1), 107.76 (d, $^1J(^{13}C–^{31}P)$ = 107 Hz, C22), 64.90 (d, $^3J(^{13}C–^{31}P)$ = 4 Hz, C5), 59.81 (s, C6), 43.73 (s, C7), 32.78 (d, C9), 32.40 (s, C8), 22.03 (d, $^3J(^{13}C–^{31}P)$ = 8 Hz, C28, C29), 21.36 (s, C30). **^{31}P NMR (243 MHz, CD_2Cl_2)**: δ = 37.24 (d, $^1J(^{31}P-^1H)$ = 540 Hz). **^{19}F NMR (565 MHz, CD_2Cl_2)**: δ = −133.09 (br, 8F, *o*-C_6F_5), −163.72 (t, $^3J(^{19}F–^{19}F)$ = 20 Hz, 4F, *p*-C6F5), −167.56 (t, br, $^3J(^{19}F–^{19}F)$ = 19 Hz, 8F, *m*-C_6F_5). **^{11}B NMR (193 MHz, CD_2Cl_2)**: δ = −16.66 (s). **HRMS ESI (m/z):** $[M]^+$ calculated. for $C_{49}H_{46}PO$, 681.32808; found, 681.32753.

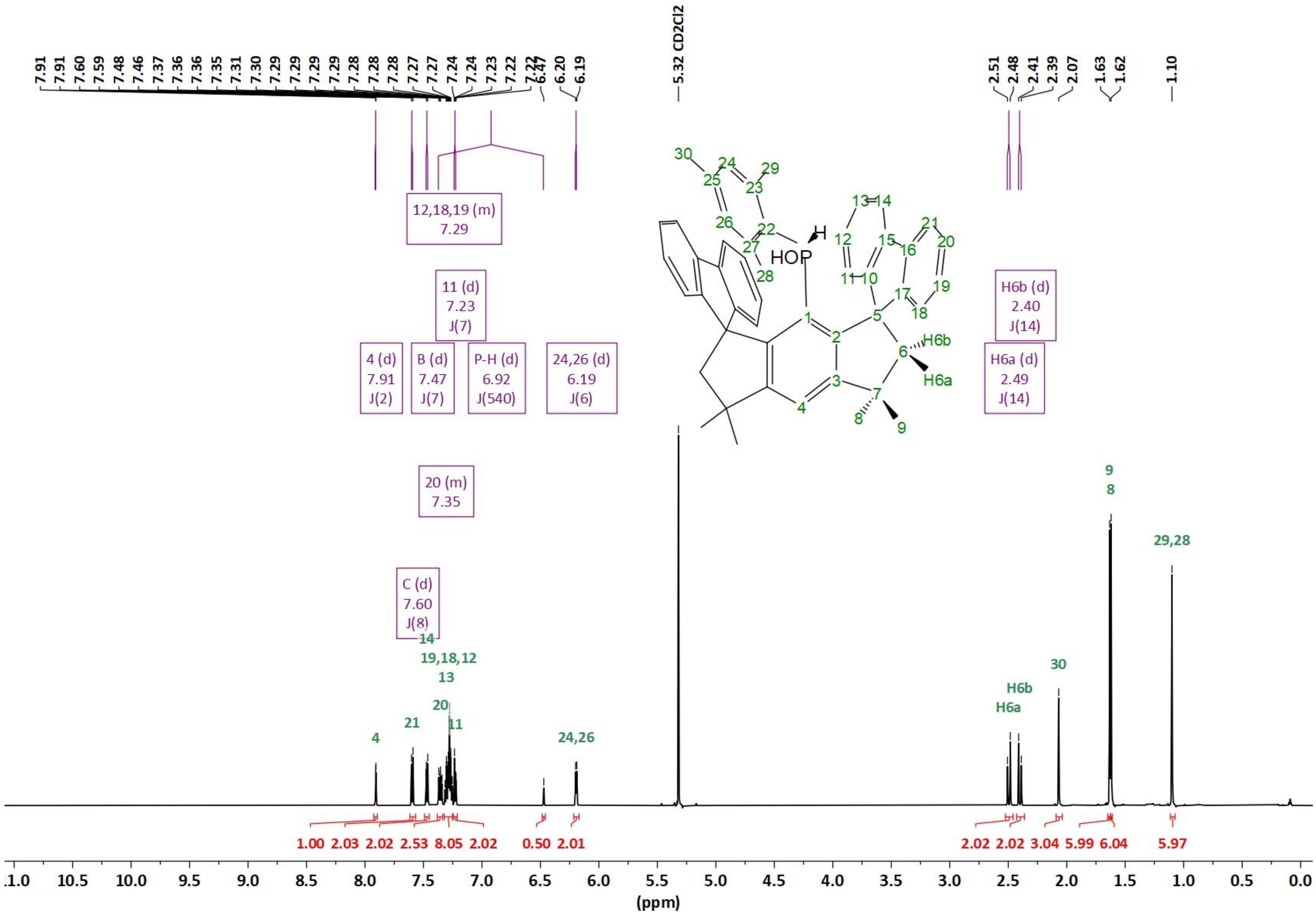

Figure S67.
^{1}H NMR (CD_2Cl_2, 600 MHz) spectrum of **11**.

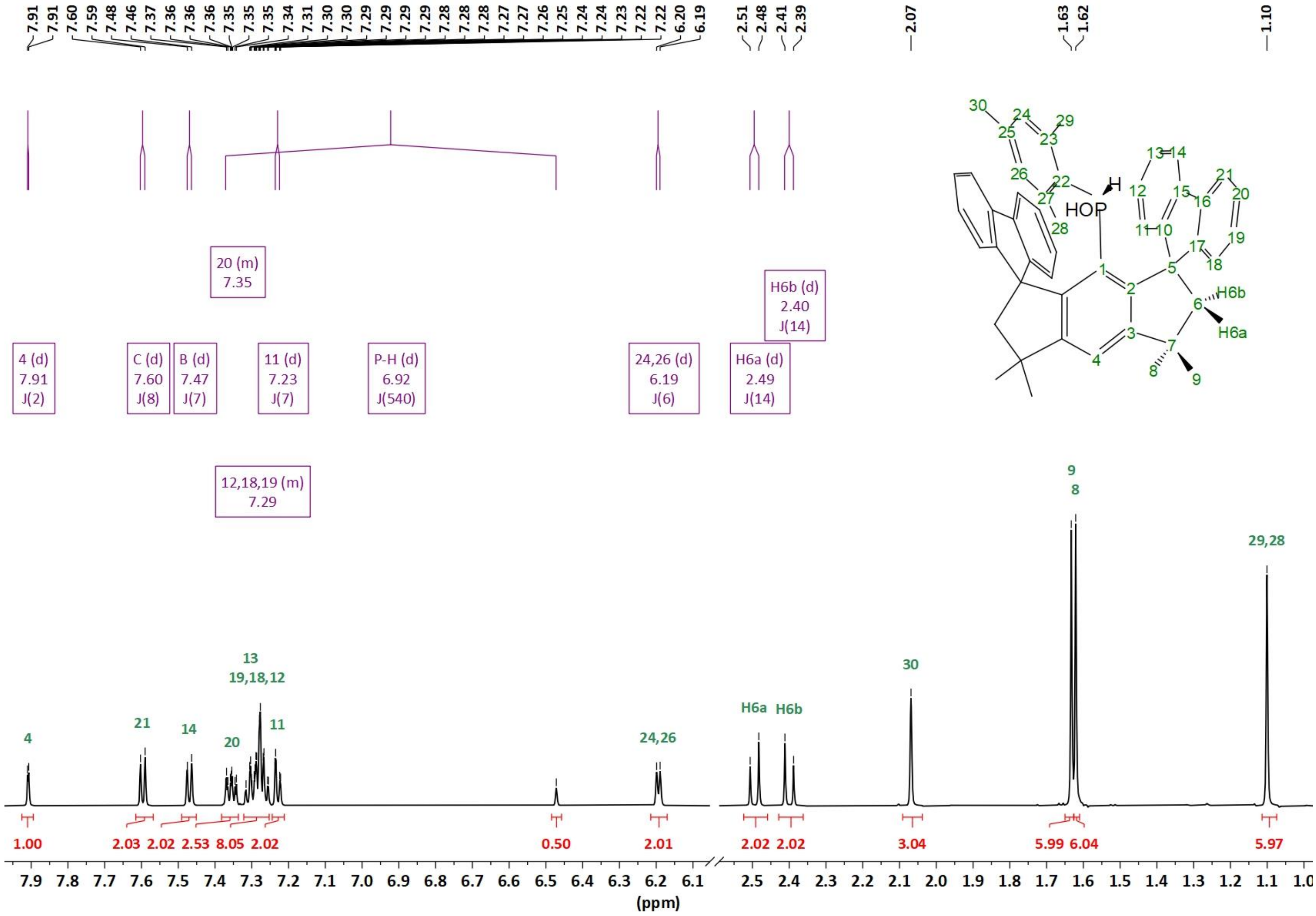

Figure S68.

Detailed ^{1}H NMR (CD_2Cl_2, 600 MHz) spectrum of **11**.

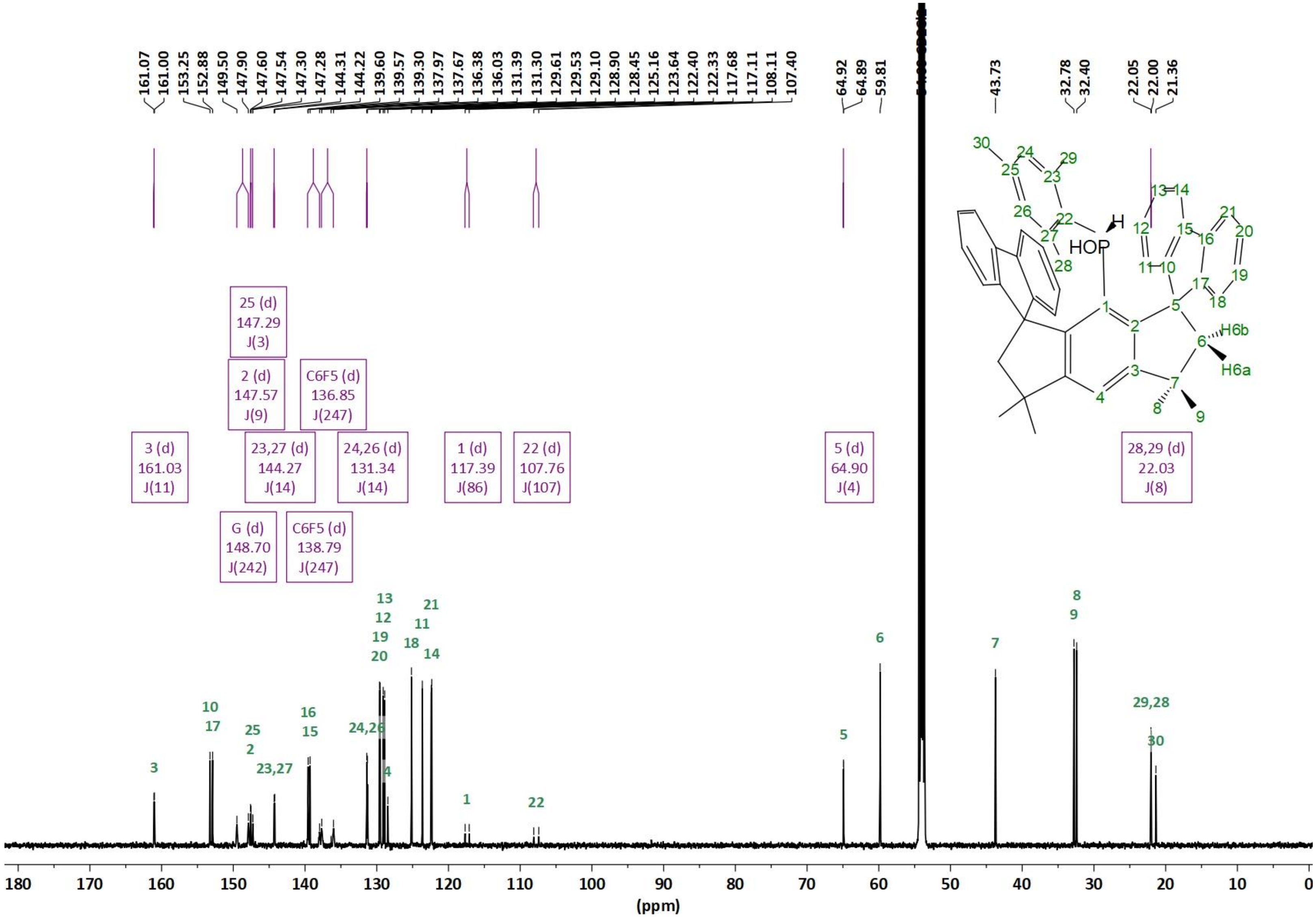

Figure S69.
$^{13}C\{^1H\}$ NMR (CD_2Cl_2, 151 MHz) spectrum of **11**.

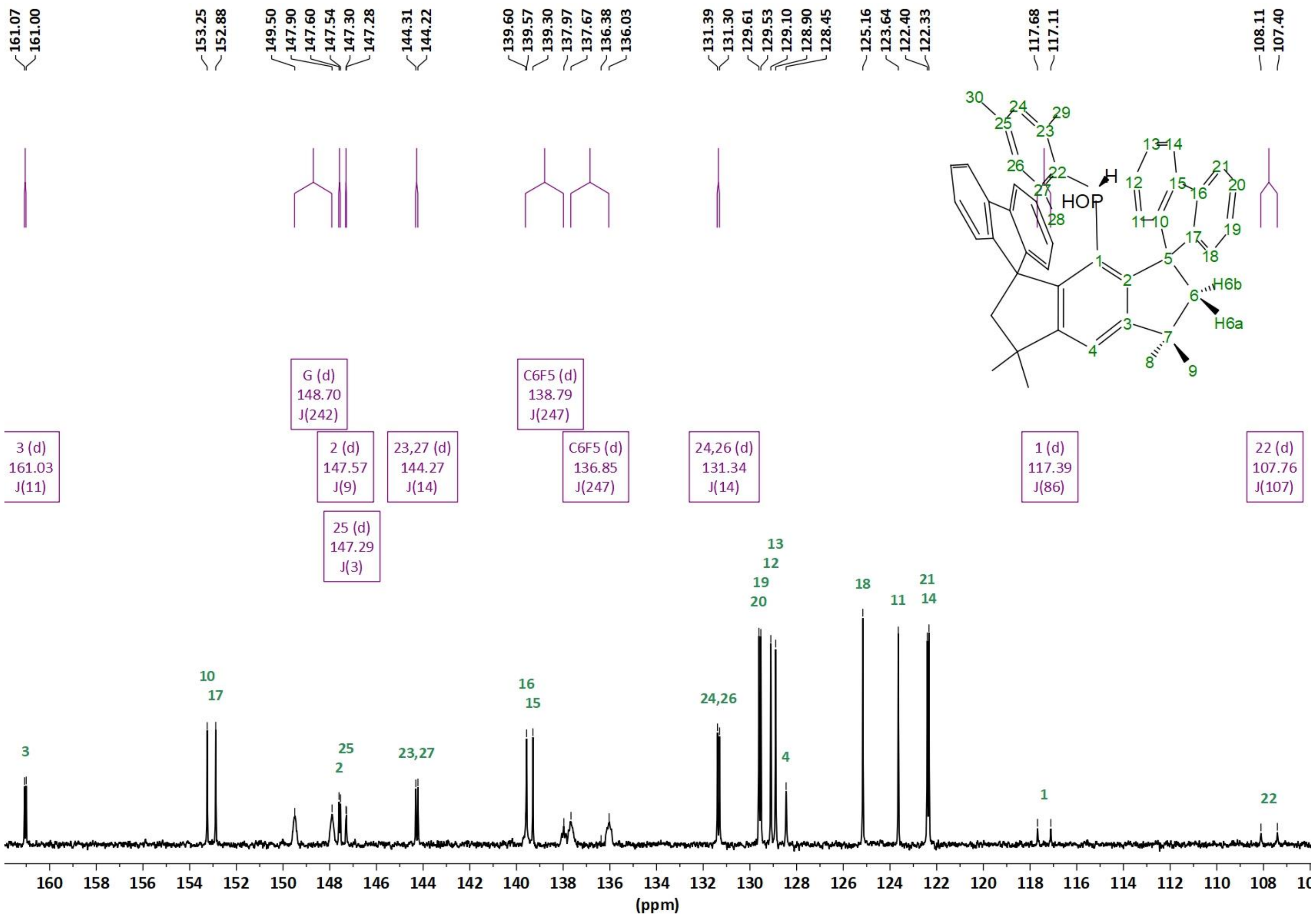

Figure S70.

Detailed $^{13}C\{^{1}H\}$ NMR (CD_2Cl_2, 151 MHz) spectrum (aromatic area) of **11**.

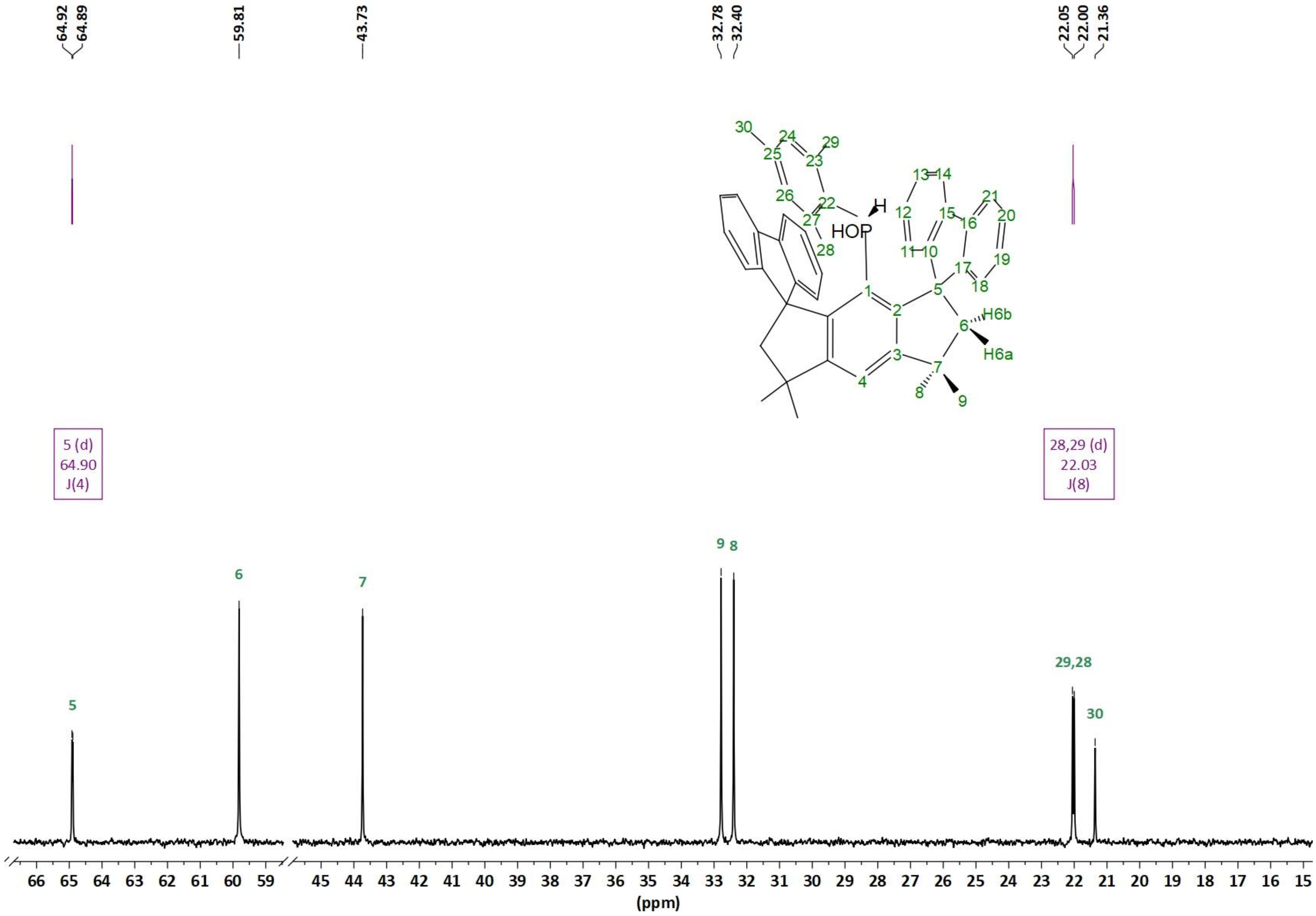

Figure S71.
Detailed $^{13}C\{^{1}H\}$ NMR (CD_2Cl_2, 151 MHz) spectrum (aliphatic area) of **11**.

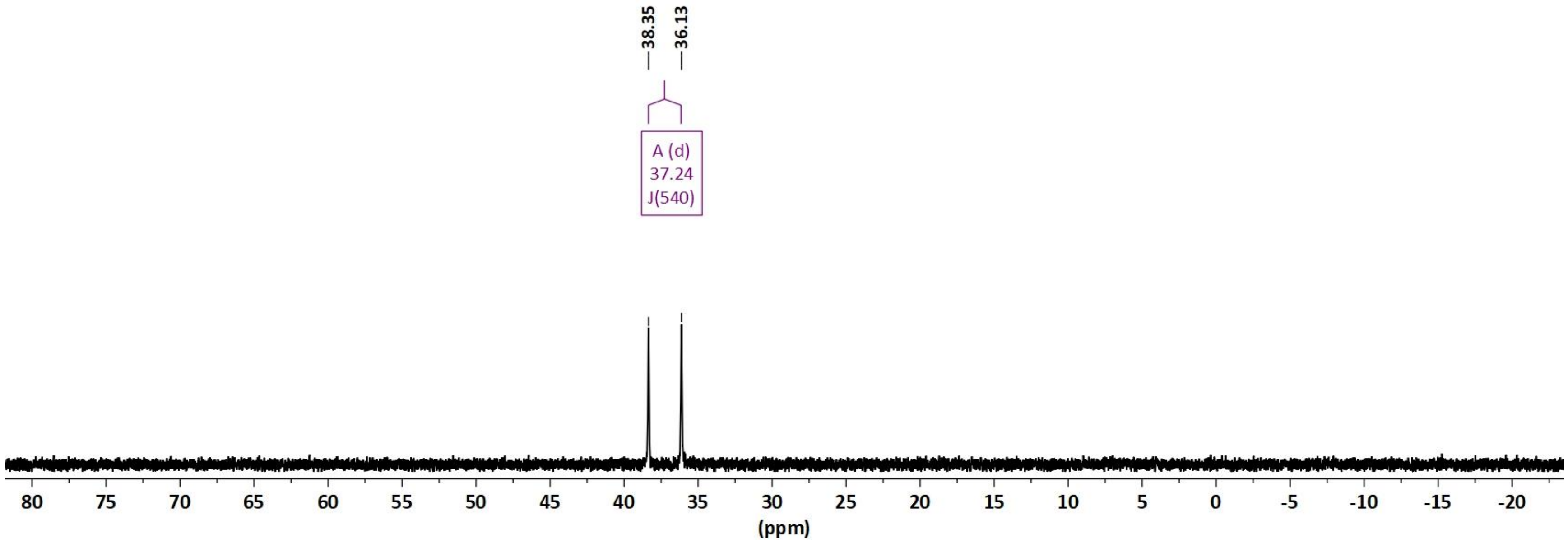

Figure S72.
^{31}P NMR (CD_2Cl_2, 243 MHz) spectrum of **11**.

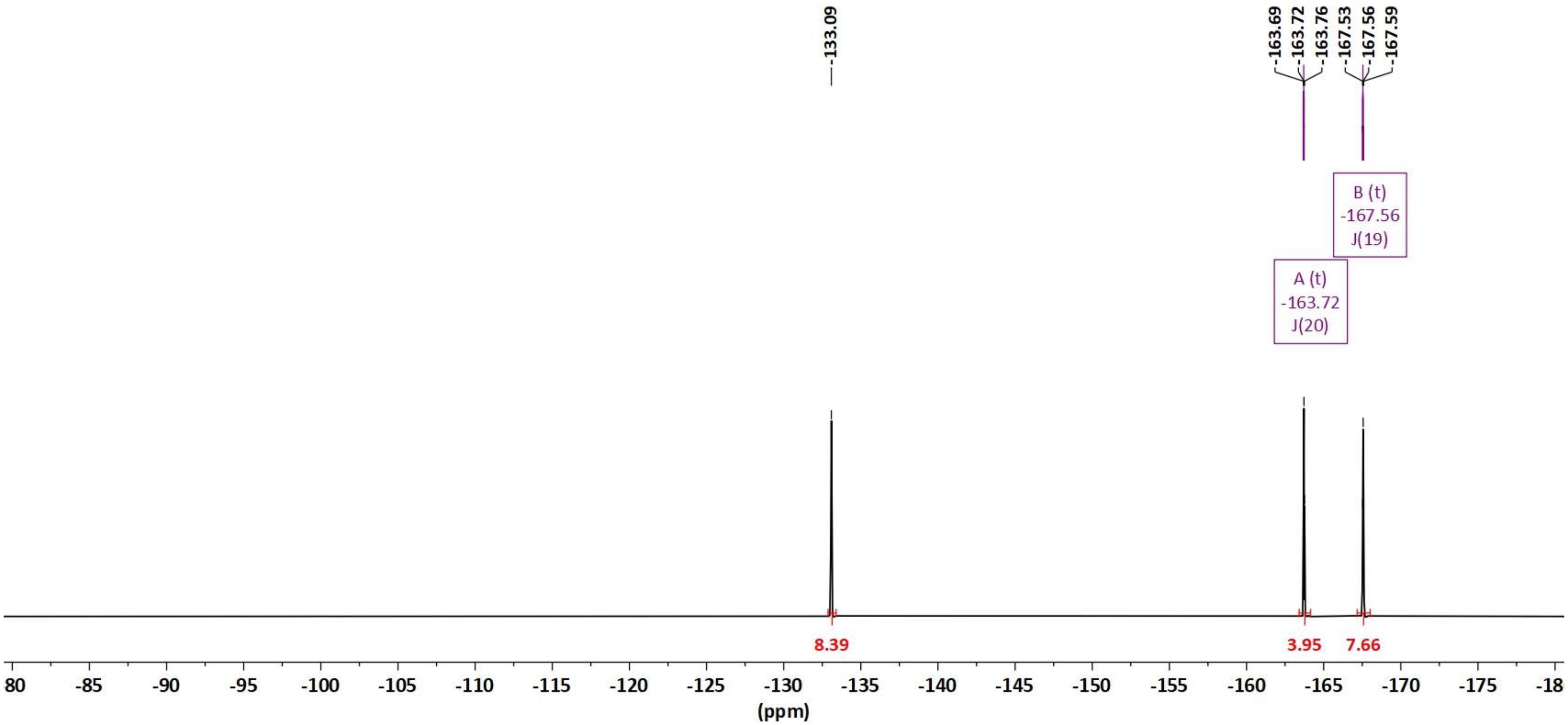

Figure S73.
^{19}F NMR (CD_2Cl_2, 565 MHz) spectrum of **11**.

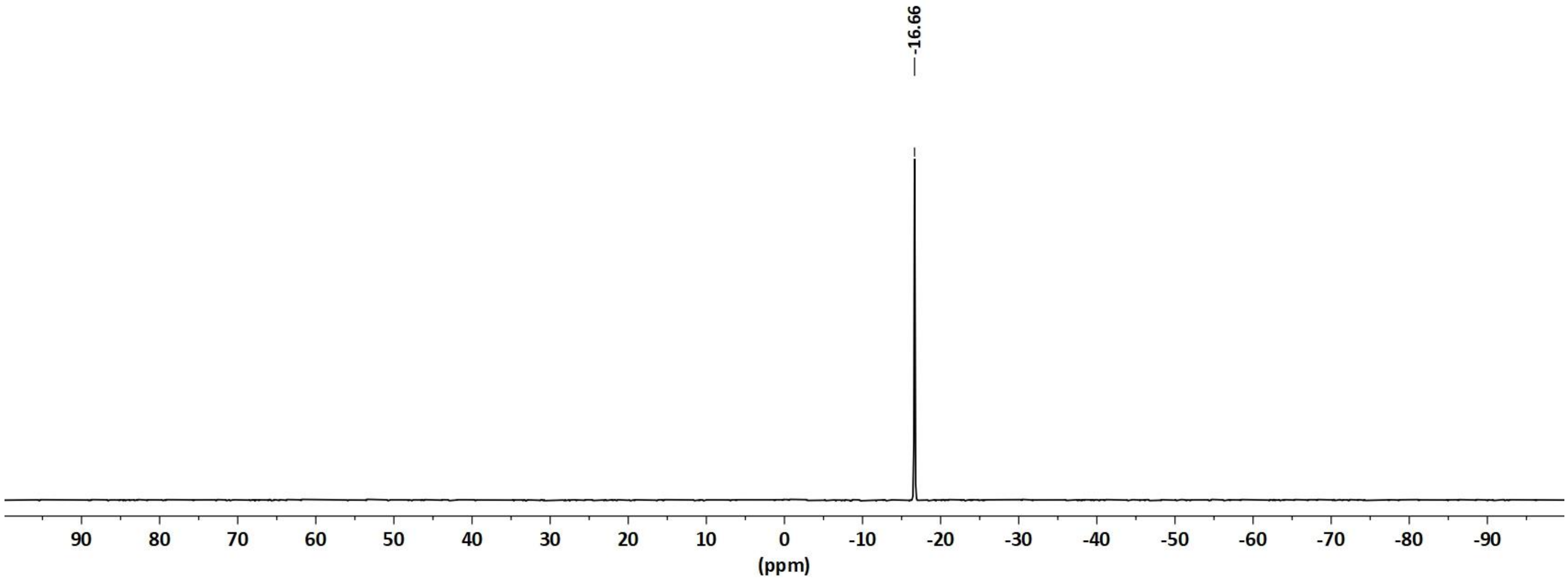

Figure S74.
^{11}B NMR (CD_2Cl_2, 193 MHz) spectrum of **11**.

Characterization of **12**

^{1}H NMR (600 MHz, CD_2Cl_2): δ = 7.94 (d, $^{5}J(^{31}P–^{1}H)$ = 2 Hz, 1H, H4), 7.55 (d, $^{3}J(^{1}H–^{1}H)$ = 7 Hz, 2H, H21), 7.38 (d, $^{3}J(^{1}H–^{1}H)$ = 7 Hz, 2H, H14), 7.35 (t, $^{3}J(^{1}H–^{1}H)$ = 7 Hz, 1H, H25), 7.29 (m, 6H, H19, H20), 7.20-7.05 (m, br, 6H, H11, H12, H13), 7.01 (m, 2H, H24, H26), 6.65 (d, $^{1}J(^{31}P–^{1}H)$ = 582 Hz, 1H, P*H*), 6.32, 6.29 (two doublets, $^{3}J(^{1}H–^{1}H)$ = 8 Hz, 2H, H23, H27), 2.58 (d, $^{2}J(^{1}H–^{1}H)$ = 14 Hz, 2H, H6a), 2.53 (d, $^{2}J(^{1}H–^{1}H)$ = 14 Hz, 2H, H6b), 1.69 (s, 6H, H8), 1.68 (s, 6H, H9). **^{13}C{^{1}H} NMR (151 MHz, CD_2Cl_2)**: δ = 160.15 (d, $^{3}J(^{13}C–^{31}P)$ = 11 Hz, C3), 153.5 (s, C17), 152.7 (s, br, C10), 150.87 (d, $^{2}J(^{13}C–^{31}P)$ = 9 Hz, C2), 148.72 (d, br, $^{1}J(^{13}C–^{19}F)$ = 240 Hz, C_6F_5), 139.69 (s, C16), 138.84 (d, br, $^{1}J(^{13}C–^{19}F)$ = 256 Hz, C_6F_5), 138.46 (s, C15), 136.86 (d, br, $^{1}J(^{13}C–^{19}F)$ = 247 Hz, C_6F_5), 135.79 (d, $^{4}J(^{13}C–^{31}P)$ = 3 Hz, C25), 130.73 (d, $^{2}J(^{13}C–^{31}P)$ = 14 Hz, C23, C27), 130.06 (s, br, C20), 129.83 (s, C12), 129.70 (s, C19), 129.55-129.46 (multiple signals overlapped, C13, C24, C26), 128.82 (d, $^{4}J(^{13}C–^{31}P)$ = 3 Hz, C4), 125.14 (s, C11), 125.07 (s, C18), 122.38 (s, C21), 121.70 (s, C14), 117.00 (d, $^{1}J(^{13}C–^{31}P)$ = 102 Hz, C22), 110.45 (d, $^{1}J(^{13}C–^{31}P)$ = 84 Hz, C1), 64.23 (d, $^{3}J(^{13}C–^{31}P)$ = 4 Hz, C5), 57.79 (s, C6), 44.05 (s, C7), 32.98 (s, C9), 32.50 (s, C9). **^{31}P NMR (243 MHz, CD_2Cl_2)**: δ = 37.25 (d, $^{1}J(^{31}P-^{1}H)$ = 583 Hz). **^{19}F NMR (565 MHz, CD_2Cl_2)**: δ = −133.09 (br, 8F, *o*-C_6F_5), −163.67 (t, $^{3}J(^{19}F–^{19}F)$ = 20 Hz, 4F, *p*-C6F5), −167.51 (t, br, $^{3}J(^{19}F–^{19}F)$ = 19 Hz, 8F, *m*-C_6F_5). **^{11}B NMR (193 MHz, CD_2Cl_2)**: δ = −16.64 (s). **HRMS ESI (m/z):** $[M]^+$ calculated. for $C_{46}H_{40}PO$, 639.28113; found, 639.28124.

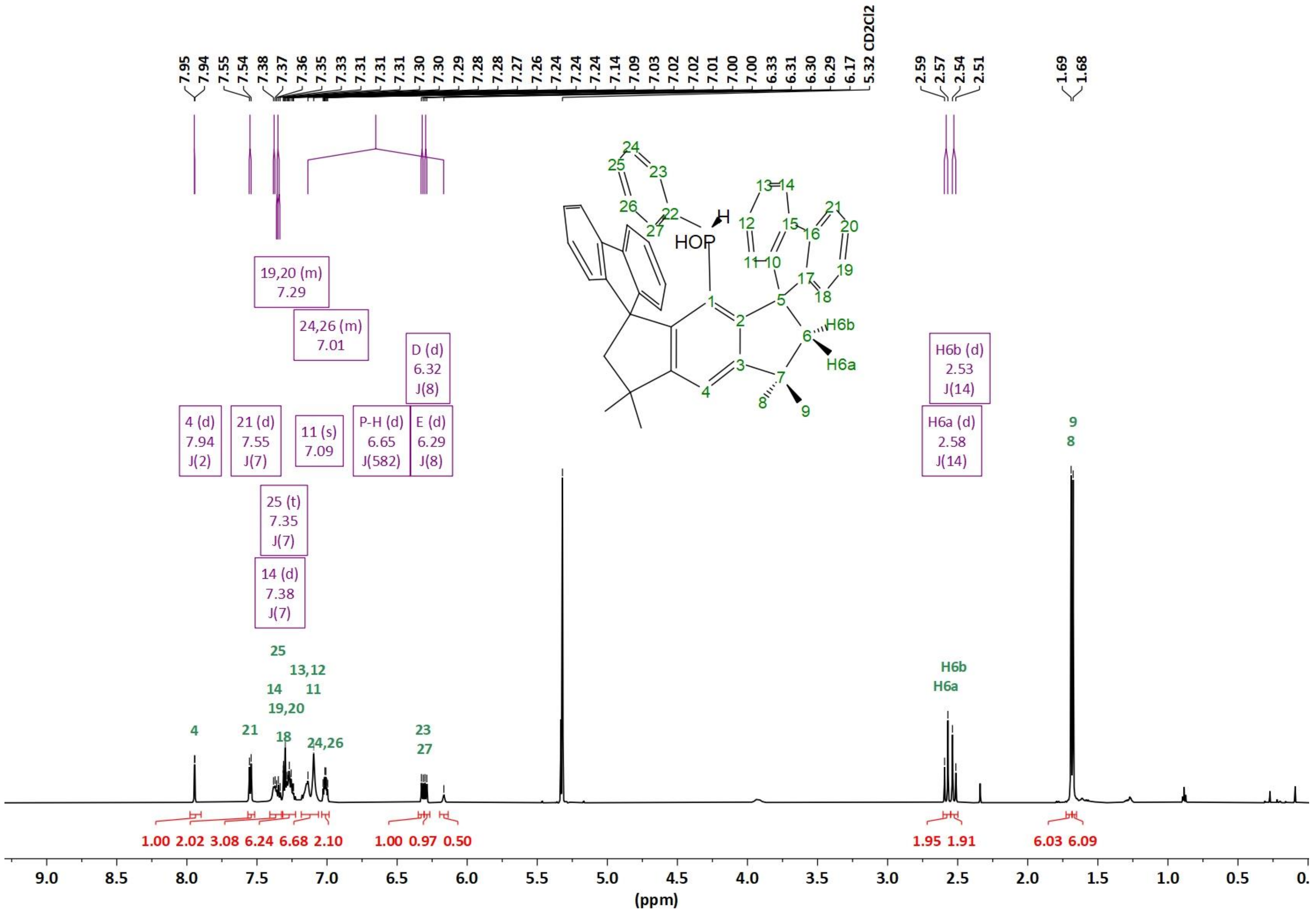

Figure S75.

^{1}H NMR (CD_2Cl_2, 600 MHz) spectrum of **12**.

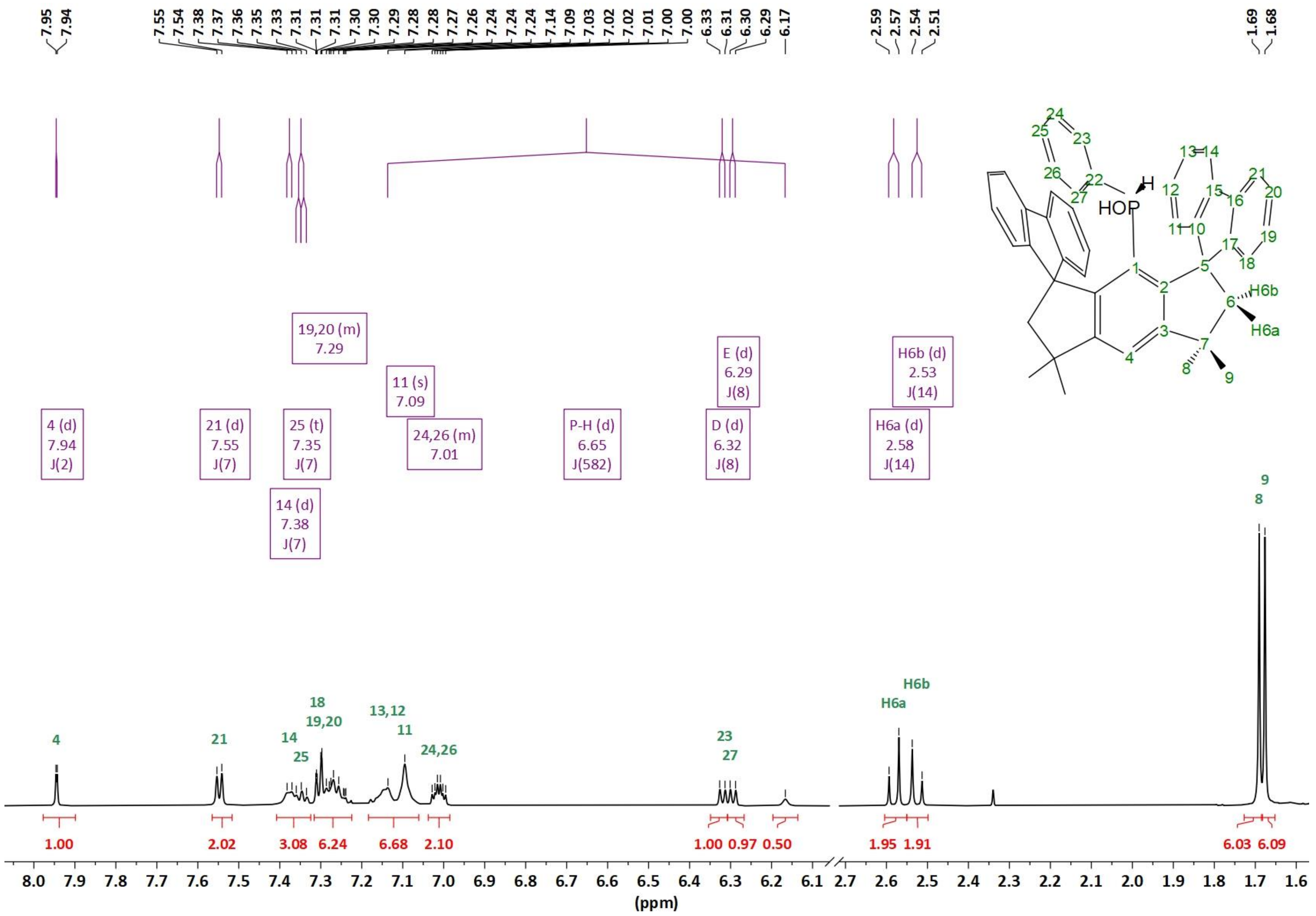

Figure S76.
Detailed ^{1}H NMR (CD_2Cl_2, 600 MHz) spectrum of **12**.

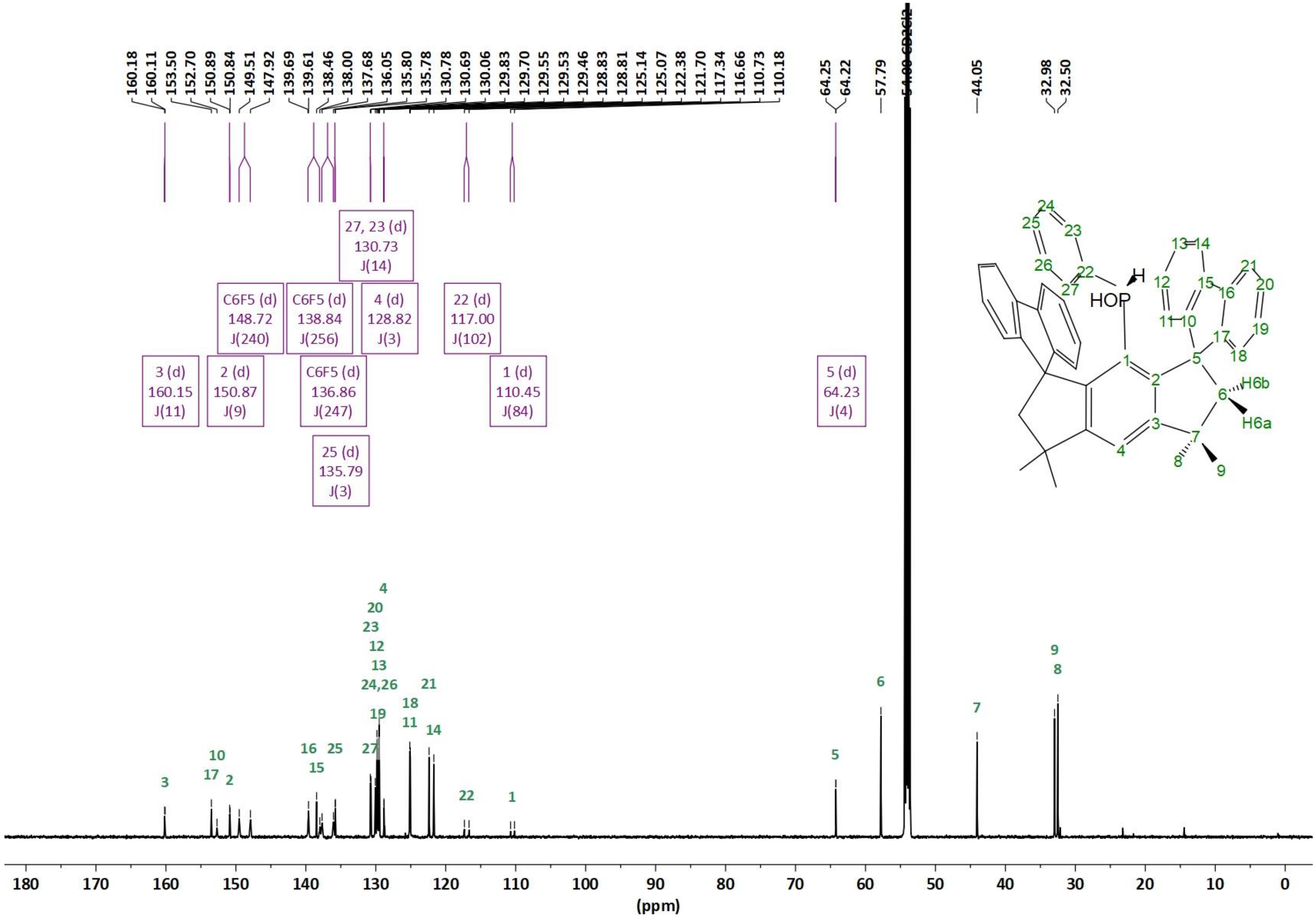

Figure S77.
$^{13}C\{^1H\}$ NMR (CD_2Cl_2, 151 MHz) spectrum of **12**.

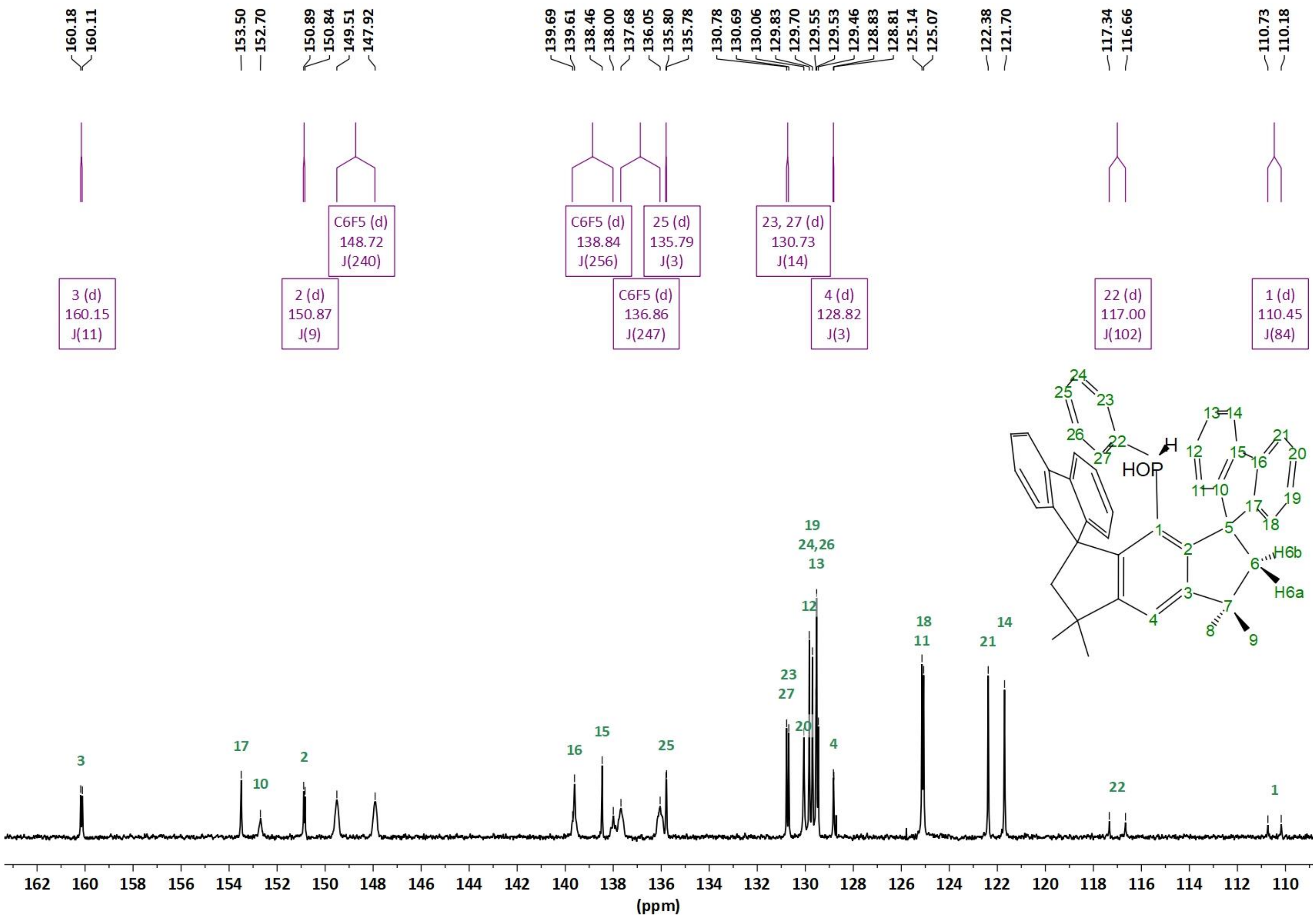

Figure S78.

Detailed $^{13}C\{^{1}H\}$ NMR (CD_2Cl_2, 151 MHz) spectrum (aromatic area) of **12**.

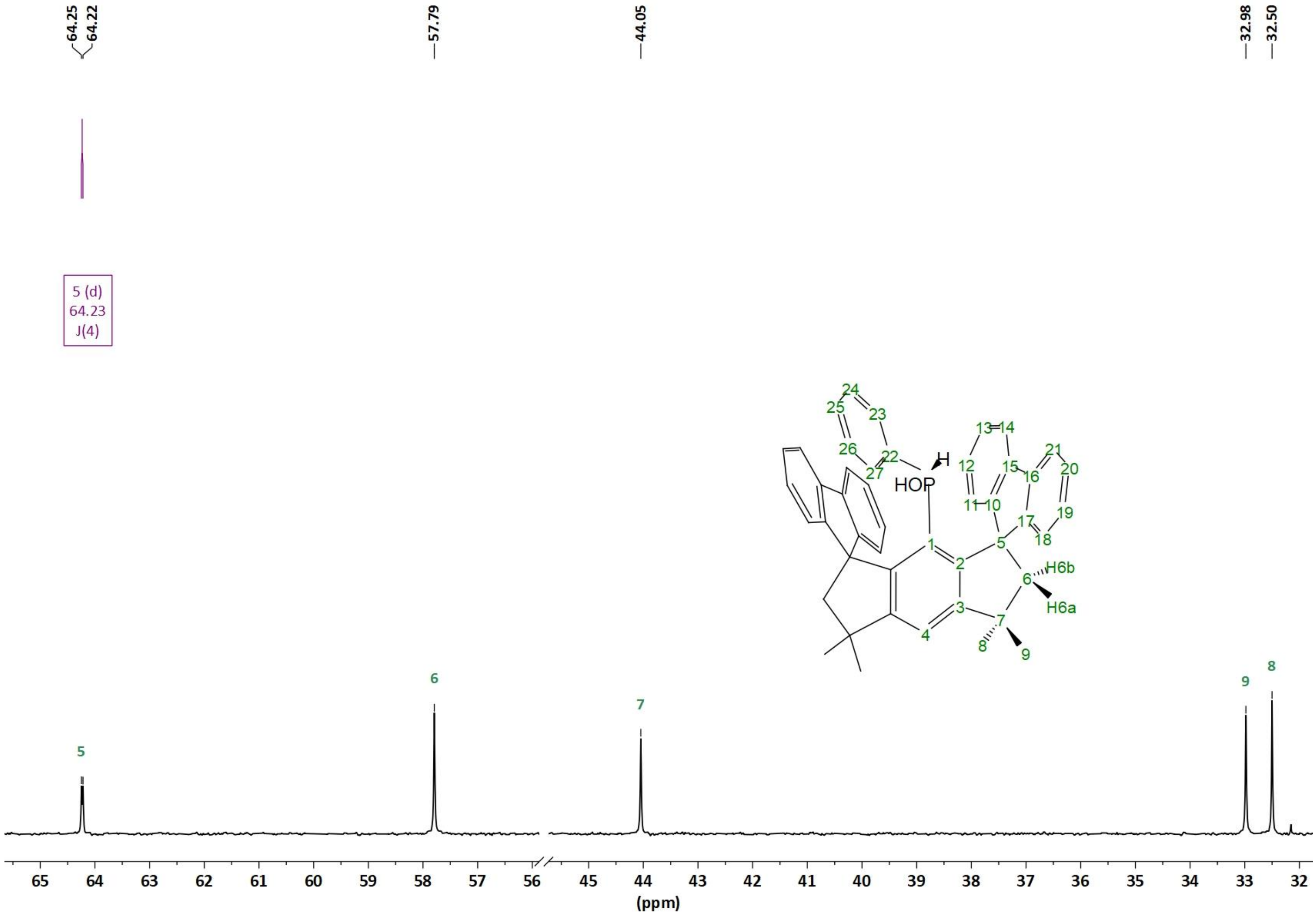

Figure S79.
Detailed $^{13}C\{^1H\}$ NMR (CD_2Cl_2, 151 MHz) spectrum (aliphatic area) of **12**.

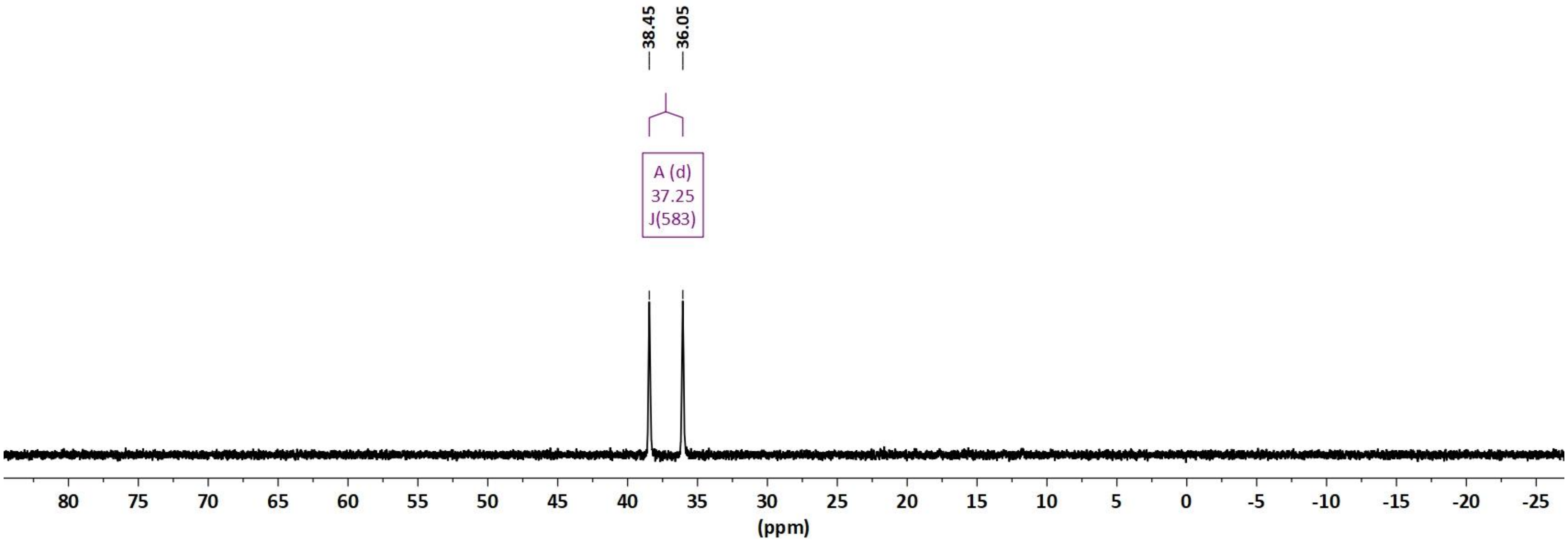

Figure S80.
^{31}P NMR (CD_2Cl_2, 243 MHz) spectrum of **12**.

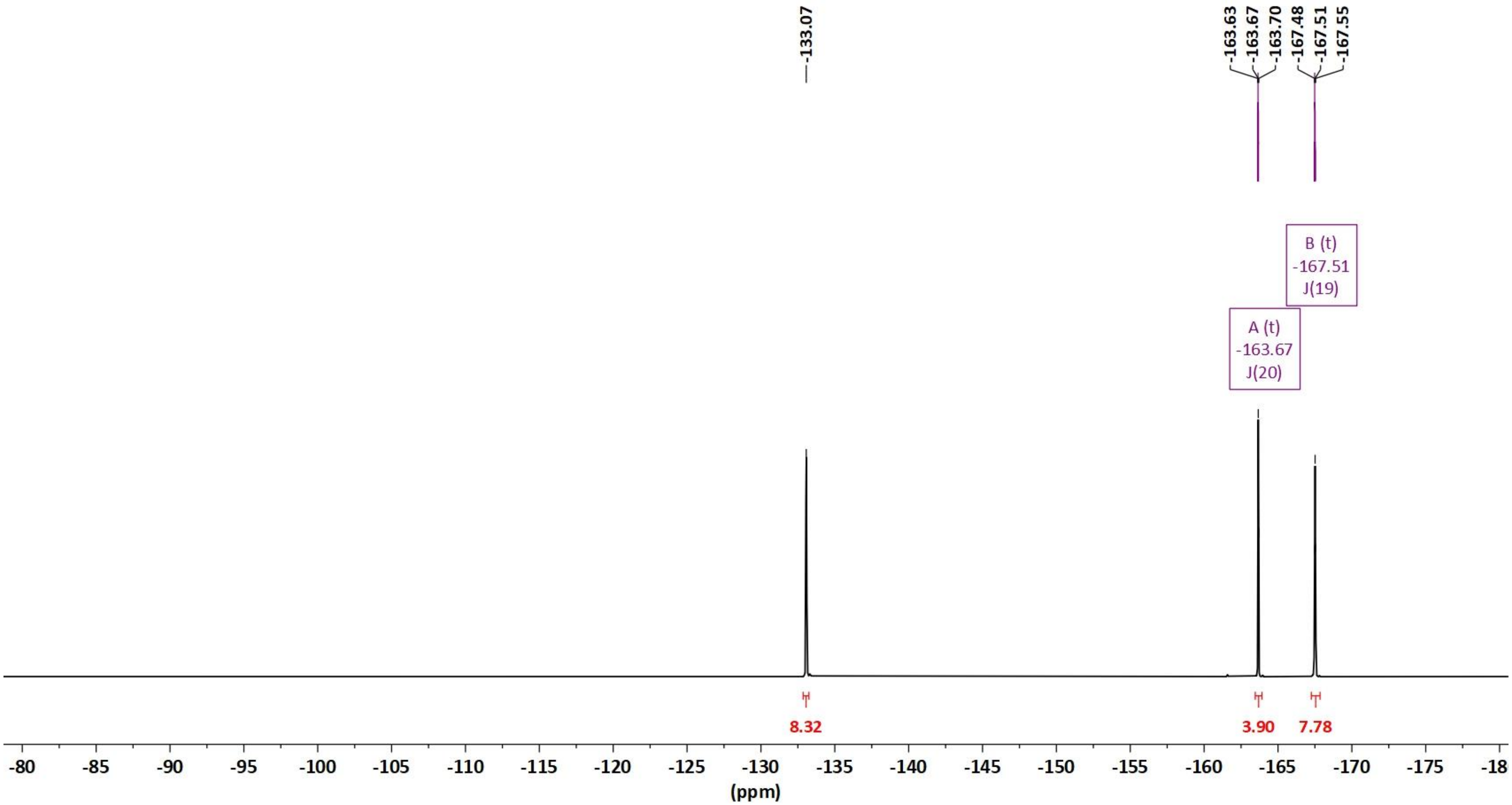

Figure S81.
^{19}F NMR (CD_2Cl_2, 565 MHz) spectrum of **12**.

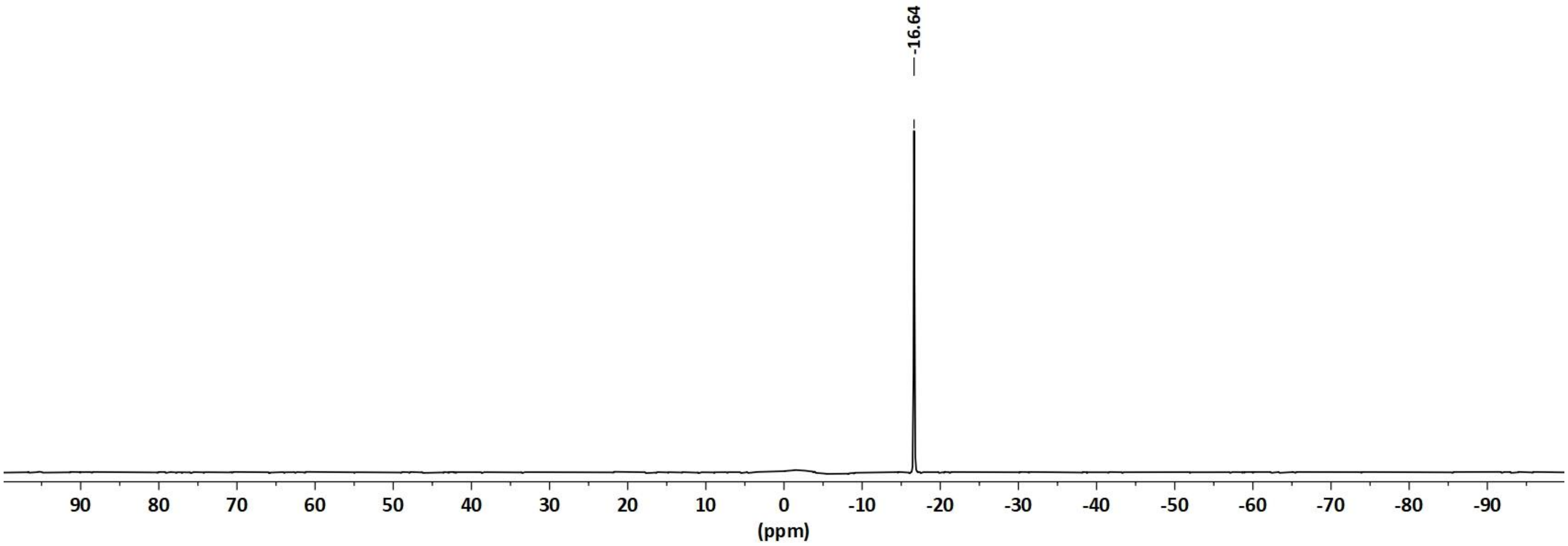

Figure S82.
^{11}B NMR (CD_2Cl_2, 193 MHz) spectrum of **12**.

Determination of the Gutmann-Beckett acceptor numbers

Lewis acidity determination experiments by the Gutmann-Beckett method were carried out according to the reported procedure.[8–10] A J. Young NMR tube was charged with the pnictogenium salt (*ca.* 18-20 mg) and an excess of Et_3PO (*ca.* 4−6 mg, 2−3 eq) in CD_2Cl_2. The ^{31}P NMR spectra were recorded at room temperature (22 °C) and the chemical shifts are listed in the table below. The acceptor numbers (AN) were determined from the equation below:

$$AN = \frac{\delta_{interacting\ OPEt_3} - 41.0}{86.14 - 41.0} \times 100$$

Table S3.

Gutmann-Beckett acceptor numbers

Compound	^{31}P NMR Ar^1Ar^2P (ppm)	^{31}P NMR $OPEt_3$ (ppm)	Acceptor number (AN)
**6**	139.83	100.53	131.9
**7**	/	93.97	117.3
**8**	138.87	102.62	136.5
**[Fc_2P][$BArF_4$]**	148.81	93.49	116.3

X-Ray diffraction studies

Intensity data of **1**–**3** and **6**–**9** was collected on a Bruker Venture D8 diffractometer at 100 K with graphite-monochromated Mo-Kα (0.7107 Å) radiation. All structures were solved by direct methods and refined based on F^2 by use of the SHELX program package as implemented in WinGX[11,12] or OLEX2.[13] All non-hydrogen atoms were refined using anisotropic displacement parameters. Hydrogen atoms attached to carbon atoms were included in geometrically calculated positions using a riding model for **1**–**3** and **6**–**8**. For **9**, H atoms were treated as a mixture of freely refined and geometrically constrained positions within the ridding model. Crystal and refinement data are collected in Tables S4–S6. Figures were created using DIAMOND.[14] Crystallographic data for the structural analyses have been deposited with the Cambridge Crystallographic Data Centre. Copies of this information may be obtained free of charge from The Director, CCDC, 12 Union Road, Cambridge CB2 1EZ, UK (Fax: +44-1223-336033; e-mail: deposit@ccdc.cam.ac.uk or http://www.ccdc.cam.ac.uk).

Table S4.

Crystal data and structure refinement of **1** and **2**.

	**1**	**2**
Formula	$C_{48}H_{49}LiO_2$	$C_{40}H_{33}Cl_2P$
Formula weight, g mol^{-1}	664.81	615.53
Crystal system	monoclinic	monoclinic
Crystal size, mm	0.25 × 0.06 × 0.06	0.1 × 0.1 × 0.2
Space group	$P2_1/c$	C_2/c
a, Å	9.997(4)	22.580(2)
b, Å	17.706(4)	9.4137(7)
c, Å	20.616(5)	14.9519(12)
α, °	90	90
β, °	100.469(14)	109.426(4)
γ, °	90	90
V, Å^3	3588.4(19)	2997.2(4)
Z	4	4
ρ_{calcd}, g cm^{-3}	1.231	1.364
μ (Mo $K\alpha$), mm^{-1}	0.072	0.300
F(000)	1424	1288
θ range, deg	2.32 to 26.37	2.36 to 28.31
Index ranges	$-12 \le h \le 12$	$-30 \le h \le 30$
	$-22 \le k \le 22$	$-12 \le k \le 12$
	$-25 \le l \le 25$	$-19 \le l \le 15$
No. of reflns collected	78909	30425
Completeness to θ_{max}	99.8%	99.9%
No. indep. Reflns	7331	3745
No. obsd reflns with $(I>2\sigma(I))$	6231	3266
No. refined params	464	211
GooF (F^2)	1.199	1.190
R_1 (F) ($I > 2\sigma(I)$)	0.0764	0.0445
wR_2 (F^2) (all data)	0.1903	0.1445
Largest diff peak/hole, e Å^{-3}	0.381 / –0.413	0.488 / –0.538
CCDC number	**2069464**	**2069465**

Table S5.

Crystal data and structure refinement of **3** and **6**

	**3**	**6**
Formula	$C_{40}H_{33}AsCl_2$	$C_{73}H_{44}BF_{20}P$
Formula weight, g mol^{-1}	659.48	1342.86
Crystal system	triclinic	monoclinic
Crystal size, mm	0.35 × 0.20 × 0.15	0.50 × 0.15 × 0.10
Space group	$P\bar{1}$	$P2_1/c$
a, Å	9.5457(8)	18.9770(16)
b, Å	11.3844(10)	16.9307(11)
c, Å	15.9258(16)	18.772(2)
α, °	70.738(4)	90
β, °	79.769(4	101.847(5)
γ, °	75.617(3)	90
V, Å^3	1573.9(3)	5902.8(9)
Z	2	4
ρ_{calcd}, g cm^{-3}	1.392	1.511
μ (Mo $K\alpha$), mm^{-1}	1.278	0.157
F(000)	680	2728
θ range, deg	2.56 to 30.57	2.17 to 33.24
Index ranges	$-13 \le h \le 12$	$-25 \le h \le 25$
	$-16 \le k \le 16$	$-19 \le k \le 22$
	$-22 \le l \le 22$	$-25 \le l \le 25$
No. of reflns collected	30265	77240
Completeness to θ_{max}	99.9%	99.9%
No. indep. Reflns	9613	14639
No. obsd reflns with ($I>2\sigma(I)$)	7618	11440
No. refined params	419	863
GooF (F^2)	1.021	1.022
R_1 (F) ($I > 2\sigma(I)$)	0.0418	0.0403
wR_2 (F^2) (all data)	0.0881	0.0958
Largest diff peak/hole, e Å^{-3}	0.519 / –0.474	0.352 / –0.281
CCDC number	**2069466**	**2069467**

Table S6.

Crystal data and structure refinement of **5** and **6**.

	**7**	**8**·1/2 $C_6H_4F_2$
Formula	$C_{73}H_{44}AsBF_{20}$	$C_{73}H_{40}BF_{21}P$
Formula weight, g mol^{-1}	1386.81	1357.83
Crystal system	monoclinic	triclinic
Crystal size, mm	0.60 × 0.40 × 0.20	0.35 × 0.35 × 0.10
Space group	$P2_1/c$	$P\bar{1}$
a, Å	19.092(2)	12.7985(16)
b, Å	16.9137(17)	14.6649(16)
c, Å	18.6504(17)	16.018(2)
α, °	90	93.084(5)
β, °	101.102(4)	101.177(5)
γ, °	90	93.609(5)
V, Å^3	5909.8(10)	2936.9(6)
Z	4	2
ρ_{calcd}, g cm^{-3}	1.559	1.535
μ (Mo $K\alpha$), mm^{-1}	0.685	0.161
F(000)	2800	1374
θ range, deg	2.56 to 30.57	2.35 to 28.28
Index ranges	$-25 \le h \le 25$	$-17 \le h \le 17$
	$-22 \le k \le 22$	$-19 \le k \le 19$
	$-24 \le l \le 24$	$-21 \le l \le 21$
No. of reflns collected	185883	87055
Completeness to θ_{max}	99.9%	99.9%
No. indep. Reflns	14652	14562
No. obsd reflns with ($I>2\sigma(I)$)	12510	11093
No. refined params	863	878
GooF (F^2)	1.040	1.031
R_1 (F) ($I > 2\sigma(I)$)	0.0357	0.0503
wR_2 (F^2) (all data)	0.0904	0.1273
Largest diff peak/hole, e Å^{-3}	0.629 / –0.564	0.629 / –0.564
CCDC number	**2069469**	**2069468**

Table S7.

Crystal data and structure refinement of **9**.

	**9**
Formula	$C_{76}H_{51}BF_{20}P$
Formula weight, g mol^{-1}	1385.94
Crystal system	triclinic
Crystal size, mm	0.30 × 0.20 × 0.20
Space group	$P\bar{1}$
a, Å	13.1056(15)
b, Å	15.9706(14)
c, Å	16.4405(19)
α, °	87.294(4)
β, °	73.503(4)
γ, °	85.413(4)
V, Å^3	3287.8(6)
Z	2
ρ_{calcd}, g cm^{-3}	1.400
μ (Mo *Kα*), mm^{-1}	0.143
F(000)	1414
θ range, deg	2.00 to 28.36
Index ranges	$-15 \le h \le 17$
	$-21 \le k \le 18$
	$-21 \le l \le 21$
No. of reflns collected	37159
Completeness to θ_{max}	98.0%
No. indep. Reflns	16135
No. obsd reflns with ($I>2\sigma(I)$)	11473
No. refined params	895
GooF (F^2)	1.055
R_1 (F) ($I > 2\sigma(I)$)	0.0622
wR_2 (F^2) (all data)	0.1695
Largest diff peak/hole, e Å^{-3}	0.660 / –0.549
CCDC number	**2069470**

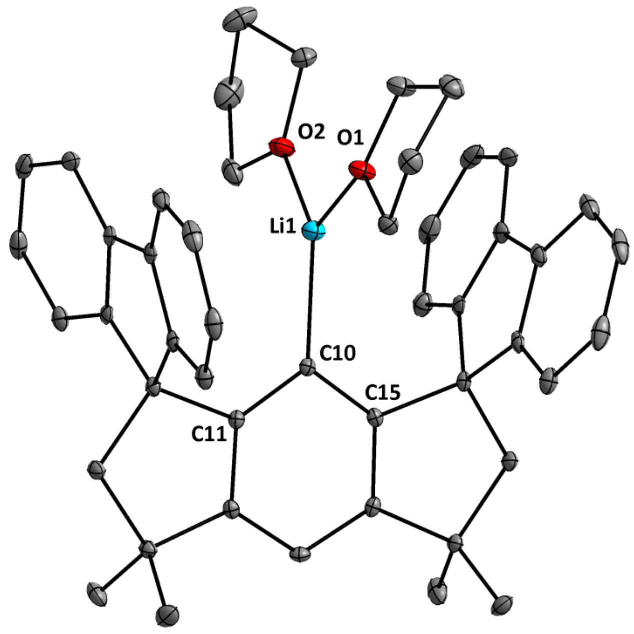

Figure S83.

Molecular structures of **1**. Thermal ellipsoids are set at 30% probability. Hydrogen atoms are omitted for clarity. Selected bond lengths [Å] and angles [°]: O1–Li1 1.944(6), O2–Li1 1.939(6), C10–Li1 2.110(6), O2–Li1–C10 127.9(3), O1–Li1–C10 122.3(3), O2–Li1–O1 109.7(3).

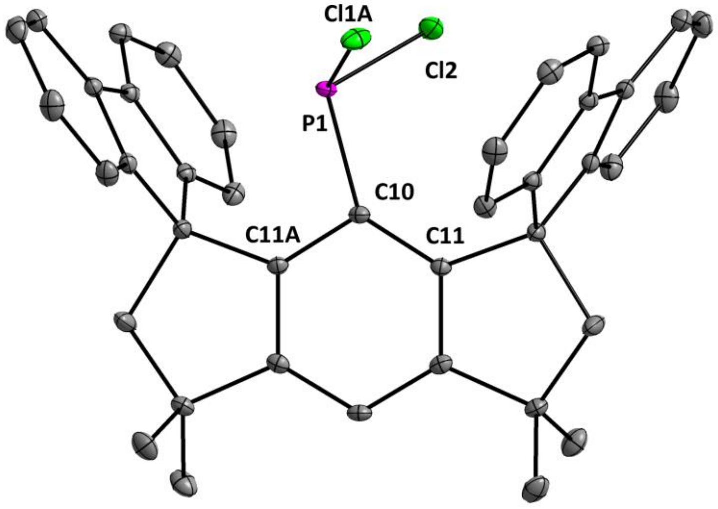

Figure S84.

Molecular structures of **2**. Thermal ellipsoids are set at 30% probability. Hydrogen atoms are omitted for clarity. Selected bond lengths [Å] and angles [°]: P1–C10 1.864(2), P1–Cl1 2.054(1), P1–Cl2 2.053(1), Cl2–P1–Cl1 101.1(1), C10–P1–Cl1 102.1(1), C10–P1–Cl2 105.26(5), C11–C10–P1 105.6(1), C11–C10–P1 137.3(1).

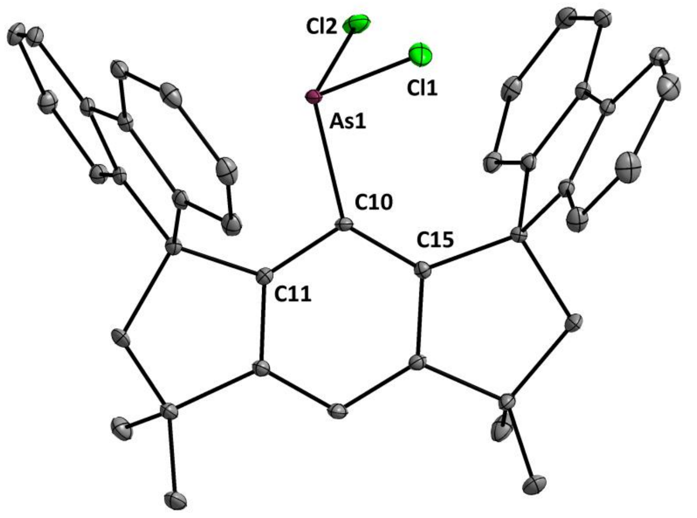

Figure S85.

Molecular structures of **3**. Thermal ellipsoids are set at 30% probability. Hydrogen atoms are omitted for clarity. Selected bond lengths [Å] and angles [°]: As1–C10 1.9839(16), As1–Cl1 2.1778(6), As1–Cl2 2.2015(6), C10–As1–Cl1 104.4(1), C10–As1–Cl2 102.1(1), Cl1–As1–Cl2 100.0(1), C15–C10–As1 135.1(1), C11–C10–As1 107.5(1).

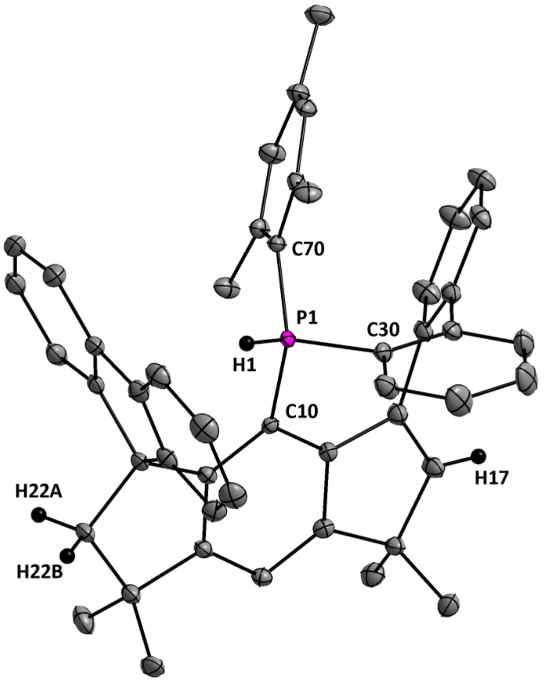

Figure S86.

Molecular structures of **9**. Thermal ellipsoids are set at 30% probability. Most hydrogen atoms are omitted for clarity. Selected bond lengths [Å] and angles [°]: P1–C10 1.792(2), P1–C70 1.782(3), P1–C30 1.795(3), C10–P1–C30 104.72(12), C70–P1–C10 122.32(11), C70–P1–C30 111.11(12).

Computational methods

Starting from the solid-state molecular geometries structural optimizations were conducted for all compounds by density functional theory (DFT) at the B3PW91/6-311+G(2df,p)[15,16] level of theory using Gaussian16.[17] Normal mode (or frequency) analysis proved all relaxed geometries to be local minima. The wavefunction files were employed for a topological analysis of the electron density according to the Atoms-In-Molecules (AIM)[18] space-partitioning scheme using AIM2000[19], whereas DGRID5-1[20] was used to generate and analyze the Electron-Localizability-Indicator (ELI-D)[21,22] related real-space bonding descriptors applying a grid step size of 0.05 a.u.. For ELI-D figures, additional grids of 0.15 a.u. step size were computed. NCI[23] grids were generated with NCIplot[24]. Molecular orbitals (MO) were extracted from the formatted checkpoint files with the cubegen subroutine of Gaussian16. Natural bond orbitals (NBO)[25,26] were calculated with NBO 5.9[27]. Bond paths are displayed with AIM2000, ELI-D and NCI figures are displayed with MolIso[28], MO and NBO images are generated with GaussView 5. Time-dependent density functional theory (TD-DFT) computations including the first 10 singlet and triplet states have been carried out for **6**–**8** at the same level of theory to generate UV/vis spectra. AIM provides a bond paths motif, which resembles and exceeds the Lewis picture of chemical bonding, disclosing all types and strengths of interactions. Additionally, it provides atomic volumes and charges. Analyses of the reduced density gradient, $s(\mathbf{r}) = [1/2(3\pi^2)^{1/3}]|\nabla\rho|/\rho^{4/3}$, according to the NCI method is used to visualize non-covalent bonding aspects. An estimation of different non-covalent contact types according to steric/repulsive ($\lambda_2 > 0$), van der Waals-like ($\lambda_2 \approx 0$), and attractive ($\lambda_2 < 0$) is facilitated by mapping the ED times the sign of the second eigenvalue of the Hessian ($\mathrm{sign}(\lambda_2)\rho$) on the *iso*-surfaces of $s(\mathbf{r})$. AIM and NCI are complemented by the ELI-D, which provides electron populations and volumes of bonding and lone-pair basins and is especially suitable for the analysis of (polar-)covalent bonding aspects. NBO, finally, connects the picture of localized chemical interactions and lone-pairs as visible e.g. in the ELI-D, with the molecular orbital (MO) expression.

Table S8.

Absolute and relative energies of the transition of compounds **6** into **9,** and **7** into the corresponding **arsonium ion** (not observed experimentally).

model	E(a.u.)	compared	ΔE(a.u.)	ΔE(kJ/mol)
**6**	-2234.9907	start-TS	0.0212	55.7
**TS(6)**	-2234.9695	TS-end	-0.0423	-111.1
**9**	-2235.0118	start-end	-0.0211	-55.3
**7**	-4129.5265	start-TS	0.0281	73.7
**TS(7)**	-4129.4985*	TS-end	-0.0121	-31.7
**Arsonium ion**	-4129.5105	start-end	0.0160	42.0

* TS-state search stopped finally with error

For compound **6**, the transition into **9** is exothermic (highlighted in green), whereas the corresponding reaction is endothermic for **7** (highlighted in orange). TS energies highlighted in yellow.

Table S9.

Real-space bonding descriptors for compounds **6**, **7**, and **8**.

**model**	**contact or basin**	**d [Å]**	**$\rho(r)_{bcp}$ [eÅ^{-3}]**	**$\nabla^2\rho(r)_{bcp}$ [eÅ^{-5}]**	**ε**	**$G/\rho(r)_{bcp}$ [a.u.]**	**$H/(r)_{bcp}$ [a.u.]**	**N_{ELI} [e]**	**V_{ELI} [Å^3]**	**γ_{ELI}**
**6**	C_{Mes}–P	1.767	1.19	-4.5	0.13	0.77	-1.03	2.60	7.1	1.80
**6**	C_{Ar1}–P	1.818	1.13	-7.5	0.12	0.52	-0.99	2.27	5.4	1.87
**6**	P–C54/59	2.801	0.18	1.0	2.23	0.45	-0.06			
**6**	P–C33	2.823	0.16	0.9	0.48	0.45	-0.05			
**6**	LP(P)–H83	2.477	0.13	1.3	0.56	0.64	0.06	2.45	13.2	2.42
**7**	C_{Mes}–P	1.907	1.04	-2.5	0.10	0.52	-0.69	2.55	7.5	1.69
**7**	C_{Ar1}–P	1.948	0.98	-2.7	0.09	0.46	-0.65	2.28	6.0	1.75
**7**	P–C59	2.796	0.19	1.1	1.94	0.45	-0.06			
**7**	P–C33	2.841	0.16	1.0	0.91	0.45	-0.04			
**7**	LP(P)–H83	2.523	0.13	1.2	0.47	0.63	0.05	2.61	14.1	1.94
**8**	C_{Mes}–P	1.776	1.19	-4.9	0.08	0.75	-1.03	2.44	5.9	1.83
**8**	C_{Ar1}–P	1.820	1.13	-7.1	0.12	0.56	-1.00	2.25	5.2	1.87
**8**	P–C54	2.762	0.18	0.9	0.83	0.43	-0.08			
**8**	P–C33	2.762	0.18	0.9	0.83	0.43	-0.08			
**8**	LP(P)							2.49	16.2	2.48

For all bonds, d is the geometric contact distance, $\rho(\mathbf{r})_{bcp}$ is the electron density at the bcp, $\nabla^2\rho(\mathbf{r})_{bcp}$ is the corresponding Laplacian, ε is the bond ellipticity, $G/\rho(\mathbf{r})_{bcp}$ and $H/\rho(\mathbf{r})_{bcp}$ are the kinetic and total energy density over $\rho(\mathbf{r})_{bcp}$ ratios, N_{ELI} and V_{ELI} are electron populations and volumes of related ELI-D basins, γ_{ELI} is the ELI-D value at the attractor position

Table S10.

Relevant NBOs of the P and As atoms in models **6**, **7**, and **8** including lone vacancies (LV, unfilled valence nonbonding orbitals), lone-pairs (LP), and C–P bonding orbitals.

6

no.	type	occ. (e)	%P	%s	%*p*	E (a.u.)	WBI
177	LV(P)	0.55	100	0	100	-0.2203	
55	LP(P)	1.93	100	71	29	-0.4870	0.097, 0.086, 0.104
59	C_{Ar1}–P	1.95	32	15	84	-0.5827	0.874
156	C_{Mes}–P	1.95	31	15	84	-0.5975	1.042

7

no.	type	occ. (e)	%P	%s	%*p*	E (a.u.)	WBI
186	LV(As)	0.50	100	0	100	-0.2174	
64	LP(As)	1.95	100	78	22	-0.5220	0.102, 0.087, 0.123
68	C_{Ar1}–As	1.95	31	12	88	-0.5427	0.837
165	C_{Mes}–As	1.95	30	12	88	-0.5535	0.957

8

no.	type	occ. (e)	%P	%s	%*p*	E (a.u.)	WBI
165	LV(P)	0.53	100	0	100	-0.2303	
52	LP(P)	1.95	100	71	29	-0.5007	0.120, 0.119, 0.076
56	C_{Ar1}–P	1.95	32	15	84	-0.5824	0.865
153	C_{Mes}–P	1.95	31	15	84	-0.6034	0.989

The lone vacancies are occupied by circa 0.53(2) e in all three models and have pure p-character. The lone-pairs are occupied by circa 1.94(1) e in all three models and are dominated to about 71-78% by s-type contributions. LV and LP are composed of P atomic orbitals only. In contrast, the energetically low lying C–P bonding orbitals are only to about 31(1)% composed of P atomic orbitals, which have mainly p-type character. WBI is the Wiberg bond index. For the LP(P/As) the three listed WBI refer to the atoms representing the weak secondary P/As···C_π contacts.

Table S11.

NPA charges (in e)

	6	7	8
Ar^1	-0.03	0.05	-0.02
Mes	-0.16	-0.24	-0.21
P/As	1.18	1.19	1.23
Σ	0.99	1.00	1.00

Table S12.
Absolute (in a.u.) and relative (in a.u. and kJ/mol) energies of the frontier orbitals (LUMO+2 to HOMO-4) for compounds **6**, **7**, and **8**.

6

type	no.	E(a.u.)	ΔE(a.u.)	ΔE(kJ/mol)
LUMO+2	179	-0.144		
LUMO+1	178	-0.151	-0.007	-18.4
LUMO	177	-0.225	-0.081	-212.7
HOMO	176	-0.323	-0.179	-470.0
HOMO-1	175	-0.330	-0.186	-488.3
HOMO-2	174	-0.337	-0.193	-506.7
HOMO-3	173	-0.341	-0.197	-517.2
HOMO-4	172	-0.344	-0.200	-525.1
				257.3

7

type	no.	E(a.u.)	ΔE(a.u.)	ΔE(kJ/mol)
LUMO+2	188	-0.146		
LUMO+1	187	-0.153	-0.007	-18.4
LUMO	186	-0.217	-0.071	-186.4
HOMO	185	-0.324	-0.178	-467.3
HOMO-1	184	-0.331	-0.185	-485.7
HOMO-2	183	-0.338	-0.192	-504.1
HOMO-3	182	-0.340	-0.194	-509.3
HOMO-4	181	-0.342	-0.196	-514.6
				280.9

8

type	no.	E(a.u.)	ΔE(a.u.)	ΔE(kJ/mol)
LUMO+2	167	-0.151		
LUMO+1	166	-0.153	-0.002	-5.3
LUMO	165	-0.229	-0.078	-204.8
HOMO	164	-0.327	-0.176	-462.1
HOMO-1	163	-0.334	-0.183	-480.5
HOMO-2	162	-0.341	-0.190	-498.8
HOMO-3	161	-0.348	-0.197	-517.2
HOMO-4	160	-0.351	-0.200	-525.1
				257.3

For compounds **6** and **8**, the lone-pair (LP, highlighted in yellow) is mainly represented by the HOMO-3 orbital (see Figure 3H in the main text), and the HOMO-LUMO band-gap (highlighted in green) is virtually identical, whereas for compound **7**, the LP is represented by the HOMO-4 MO, and the band-gap is circa 10% larger. In all three cases, the LUMO represents the non-bonding π*-orbital at the P atom (highlighted in orange, see Figure 3G in the main text as well as Figures S91b,f,j below).

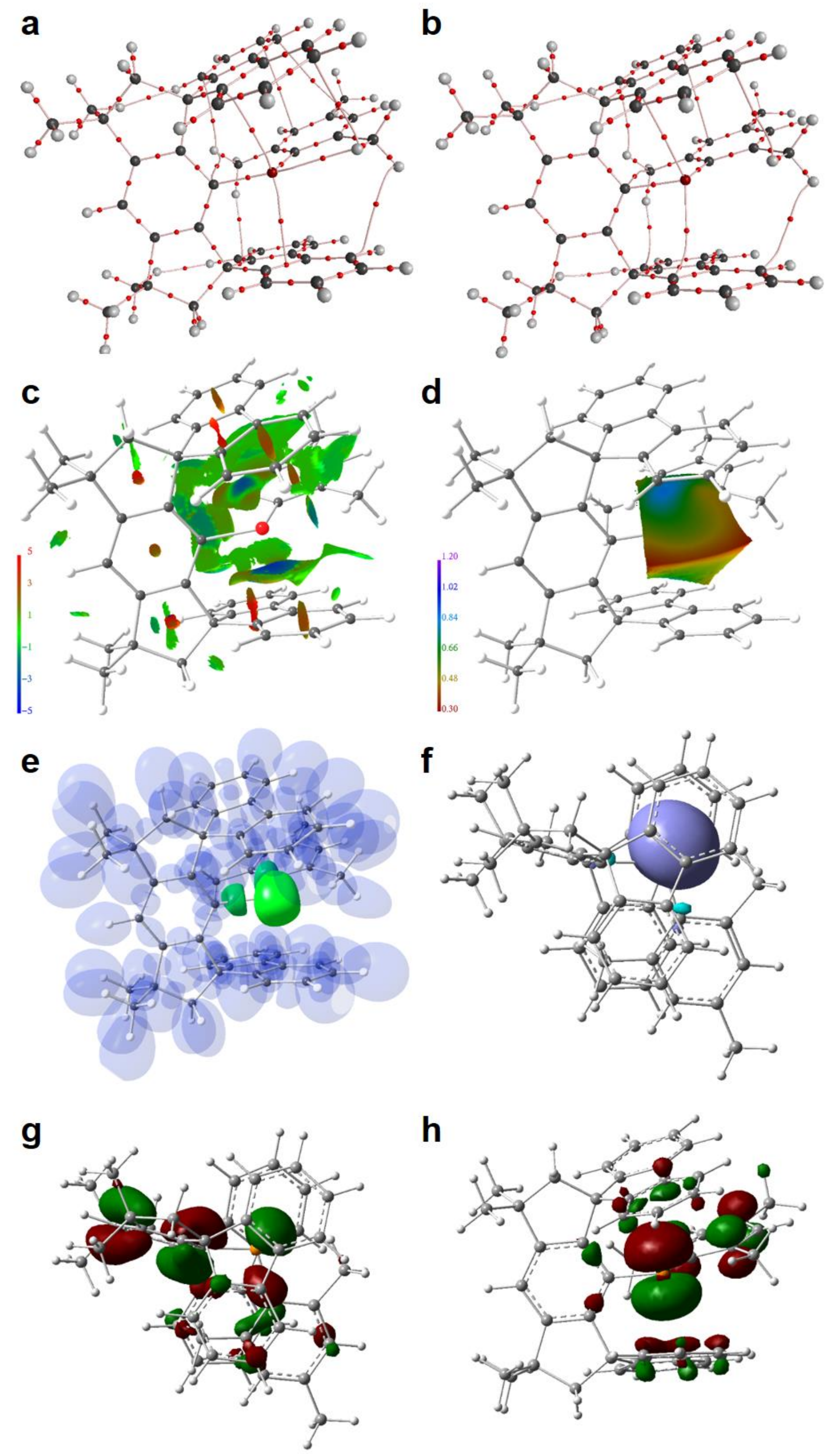

Figure S88.

Real-space and orbital-based bonding discriminators for compound **6** derived from DFT. (a) AIM bond topology, (b) virial field function, (c) NCI *iso*-surface (s(**r**) = 0.5), (d) ELI-D distribution mapped on the ELI-D basin surface of the P atoms LP, (e) ELI-D *iso*-surface (γ = 1.3), (f) *iso*-surface (0.04 a.u.) of the P(LP) NBO, (g,h) *iso*-surfaces (0.04 a.u.) of the HOMO-3 and LUMO.

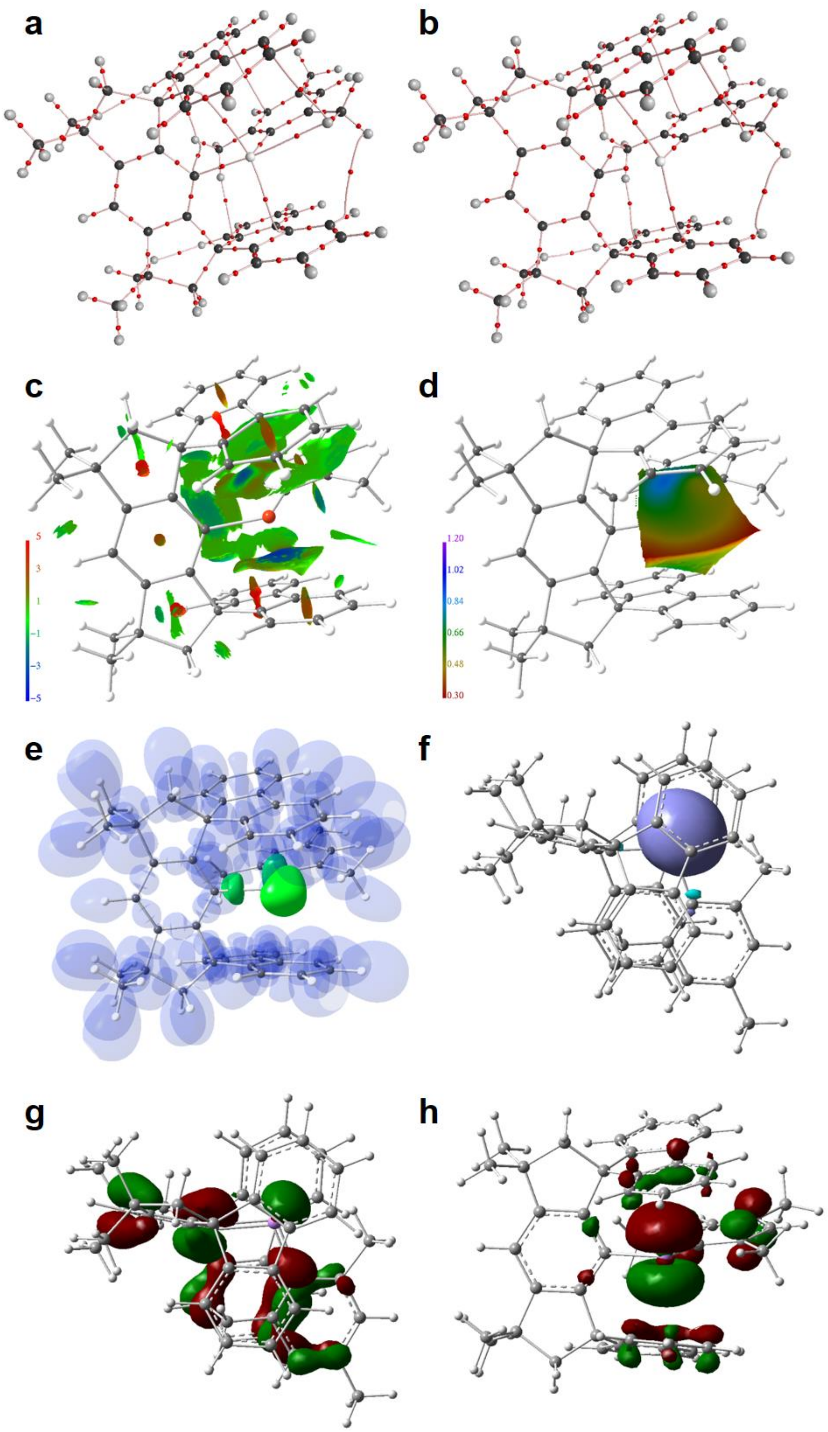

Figure S89.

Real-space and orbital-based bonding discriminators for compound **7** derived from DFT. (a) AIM bond topology, (b) virial field function, (c) NCI *iso*-surface (s(**r**) = 0.5), (d) ELI-D distribution mapped on the ELI-D basin surface of the As atoms LP, (e) ELI-D *iso*-surface (γ = 1.3), (f) *iso*-surface (0.04 a.u.) of the As(LP) NBO, (g,h) *iso*-surfaces (0.04 a.u.) of the HOMO-4 and LUMO.

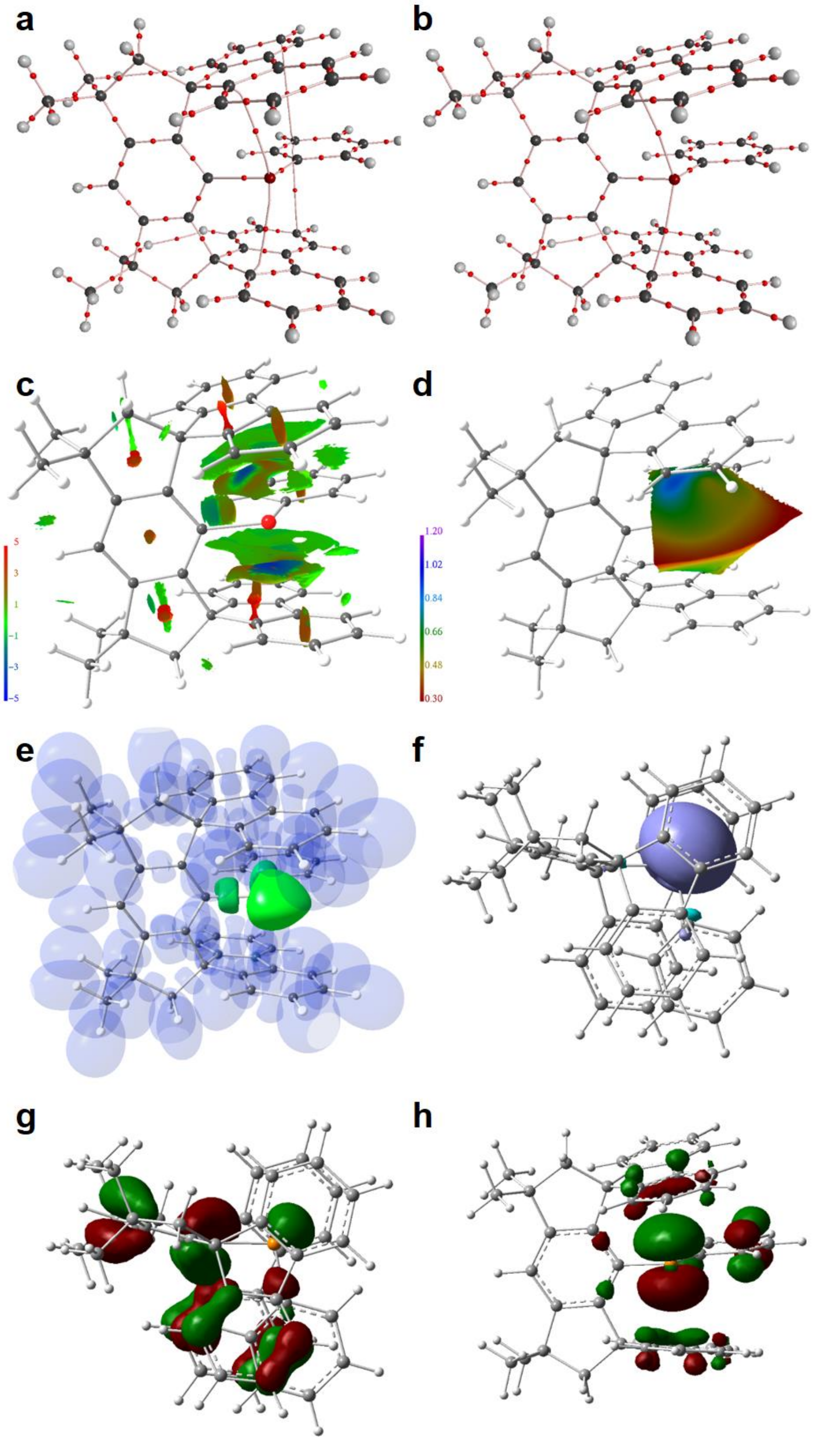

Figure S90.

Real-space and orbital-based bonding discriminators for compound **8** derived from DFT. (a) AIM bond topology, (b) virial field function, (c) NCI *iso*-surface (s(**r**) = 0.5), (d) ELI-D distribution mapped on the ELI-D basin surface of the P atoms LP, (e) ELI-D *iso*-surface (γ = 1.3), (f) *iso*-surface (0.04 a.u.) of the P(LP) NBO, (g,h) *iso*-surfaces (0.04 a.u.) of the HOMO-3 and LUMO.

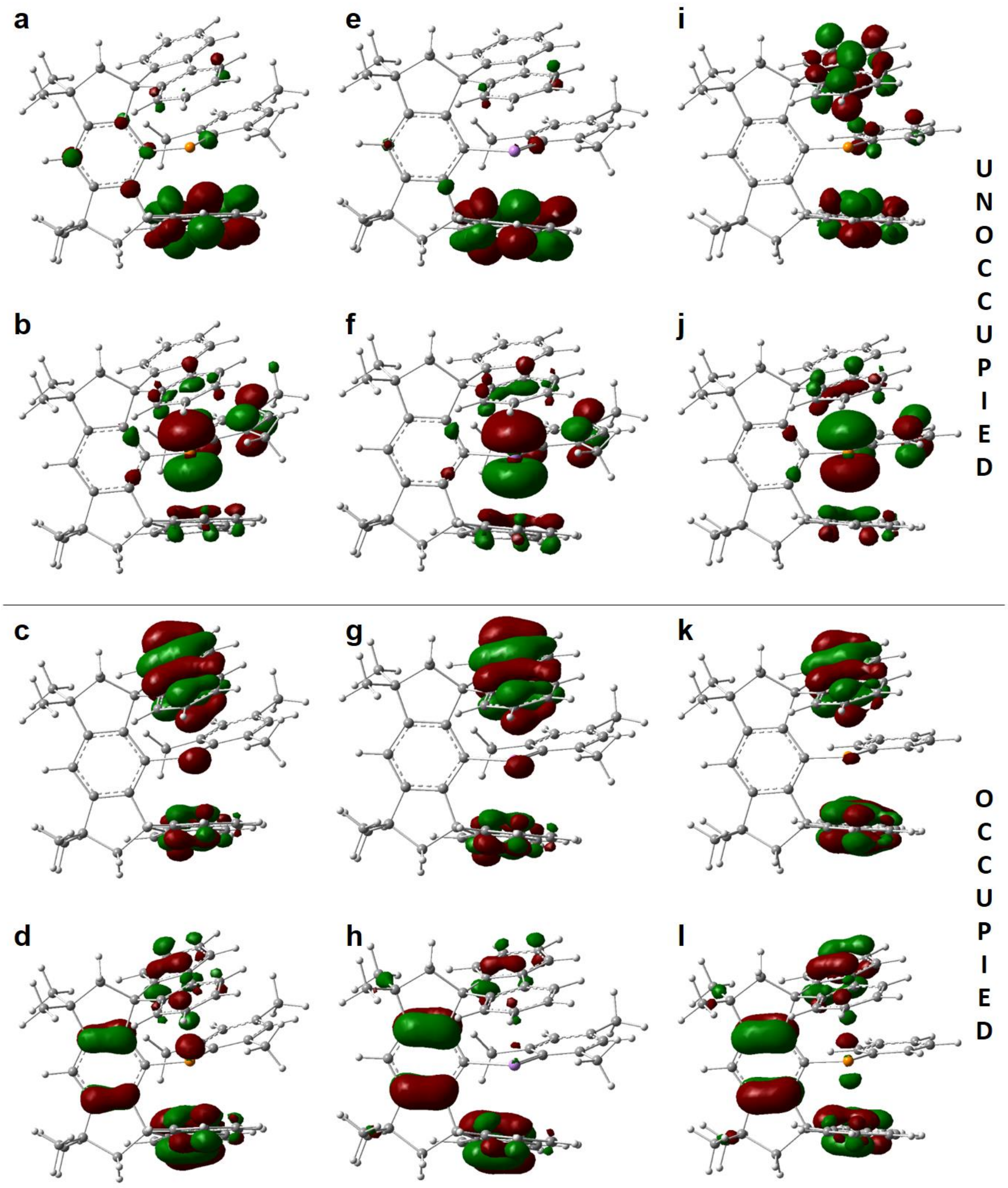

Figure S91.

MO *iso*-surface representations (0.04 a.u.) of LUMO+1 to HOMO-1 for compounds **6** (a-d), **7** (e-h), and **8** (i-l). The HOMOs show minor LP-contributions, the LUMO mainly reflects the anti-bonding π* orbital.

Table S13.

TD-DFT computed excited states of **6**, 7, and **8** (b3pw91/6-311+G(2df,p))

	**6**				**7**				**8**			
no.	state	E (eV)	E (nm)	f	state	E (eV)	E (nm)	f	state	E (eV)	E (nm)	f
1	T1	1.6581	747.75	0.0000	T1	1.9522	635.09	0.0000	T1	1.7754	698.33	0.0000
2	T2	1.9082	649.73	0.0000	T2	2.1762	569.72	0.0000	T2	1.9225	644.91	0.0000
3	T3	2.0429	606.90	0.0000	T3	2.2803	543.71	0.0000	S1	2.1169	585.70	0.1073
4	S1	2.0713	598.57	0.0902	S1	2.3141	535.78	0.0893	T3	2.1589	574.30	0.0000
5	T4	2.2239	557.51	0.0000	T4	2.3840	520.07	0.0000	S2	2.2466	551.87	0.0025
6	S2	2.2430	552.76	0.0165	S2	2.4622	503.54	0.0166	T4	2.2724	545.62	0.0000
7	T5	2.3238	533.54	0.0000	T5	2.4812	499.69	0.0000	S3	2.4072	515.05	0.0034
8	S3	2.3351	530.95	0.0106	T6	2.5308	489.91	0.0000	T5	2.4089	514.69	0.0000
9	T6	2.3517	527.21	0.0000	S3	2.5653	483.31	0.0071	S4	2.4770	500.54	0.0102
10	S4	2.3981	517.01	0.0045	S4	2.6085	475.30	0.0140	T6	2.6008	476.71	0.0000
11	T7	2.5893	478.84	0.0000	S5	2.7354	453.26	0.0044	T7	2.6710	464.19	0.0000
12	S5	2.6097	475.09	0.0101	T7	2.8176	440.04	0.0000	S5	2.7107	457.39	0.0109
13	S6	2.7213	455.60	0.0237	S6	2.9214	424.40	0.0364	T8	2.7707	447.48	0.0000
14	T8	2.7452	451.64	0.0000	T8	2.9216	424.37	0.0000	S6	2.8144	440.53	0.0126
15	S7	2.8214	439.43	0.0033	T9	3.0489	406.65	0.0000	T9	2.9301	423.14	0.0000
16	T9	2.8544	434.36	0.0000	S7	3.0665	404.32	0.0075	S7	2.9770	416.47	0.0282
17	T10	2.9636	418.36	0.0000	T10	3.0865	401.70	0.0000	T10	2.9770	416.47	0.0000
18	S8	3.0360	408.38	0.0138	S8	3.2025	387.15	0.0730	S8	3.1319	395.87	0.0038
19	S9	3.1215	397.19	0.0944	S9	3.2472	381.82	0.0421	S9	3.3545	369.61	0.0686
20	S10	3.4086	363.74	0.0336	S10	3.5597	348.30	0.0022	S10	3.5458	349.67	0.0136

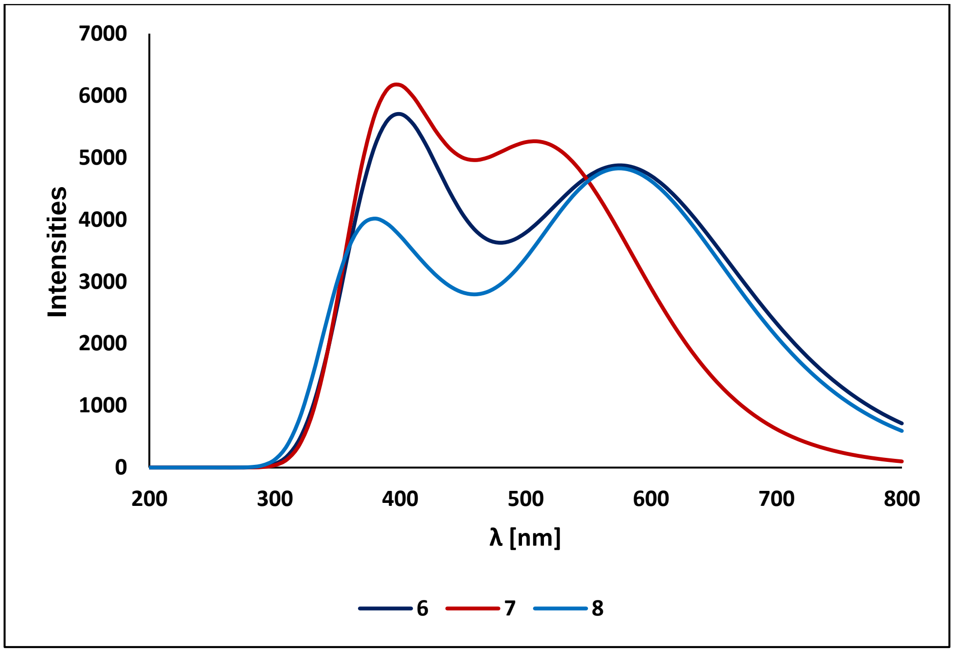

Figure S92.

Predicted UV/vis absorption spectra of **6**, **7**, and **8** (b3pw91/6-311+G(2df,p)). σ = 0.4 eV was used for peak broadening (Gaussian).

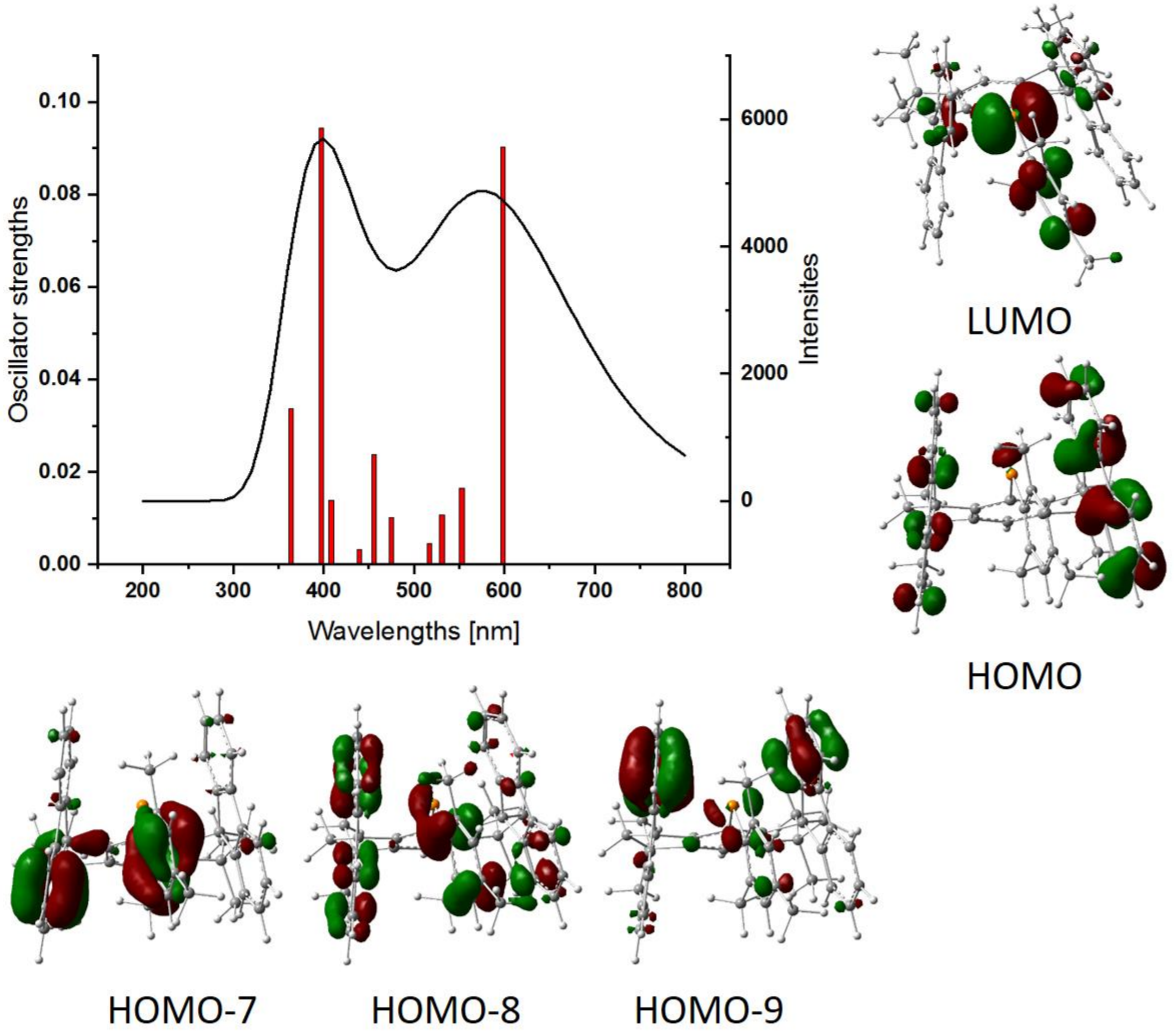

Figure S93.

Predicted UV spectrum (black line) and oscillator strengths (red bars) of **6** as well as MOs relevant for the two major transitions at about 400 nm (HOMO-9/HOMO-8/HOMO-7 → LUMO) and 600 nm (HOMO → LUMO); *iso*-surface values at 0.04 a.u. for every MO.

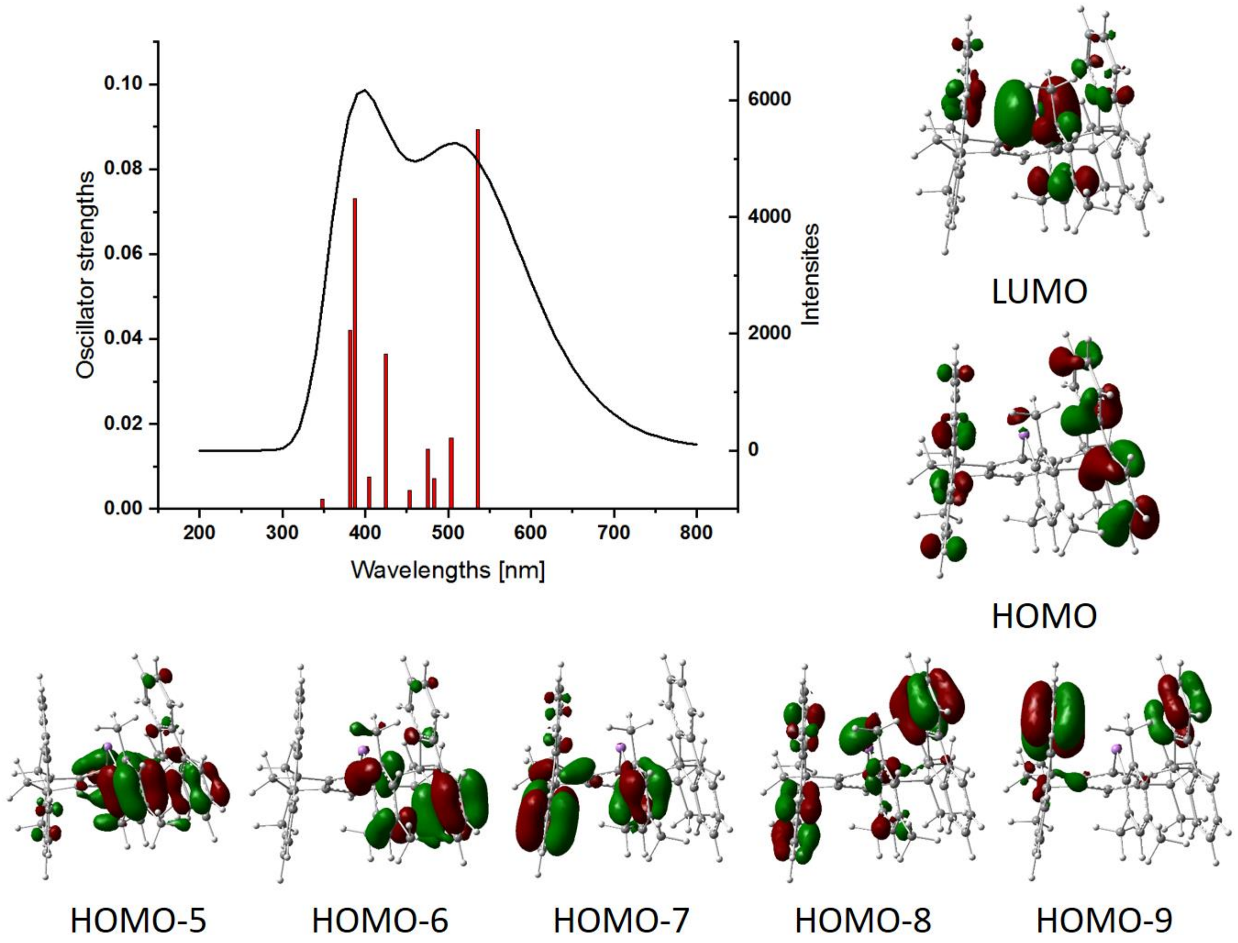

Figure S94.

Predicted UV spectrum (black line) and oscillator strengths (red bars) of **7** as well as MOs relevant for the three major transitions at about 385 nm (HOMO-9/HOMO-8/HOMO-7/HOMO-6/HOMO-5 → LUMO) and 535 nm (HOMO → LUMO); *iso*-surface values at 0.04 a.u. for every MO.

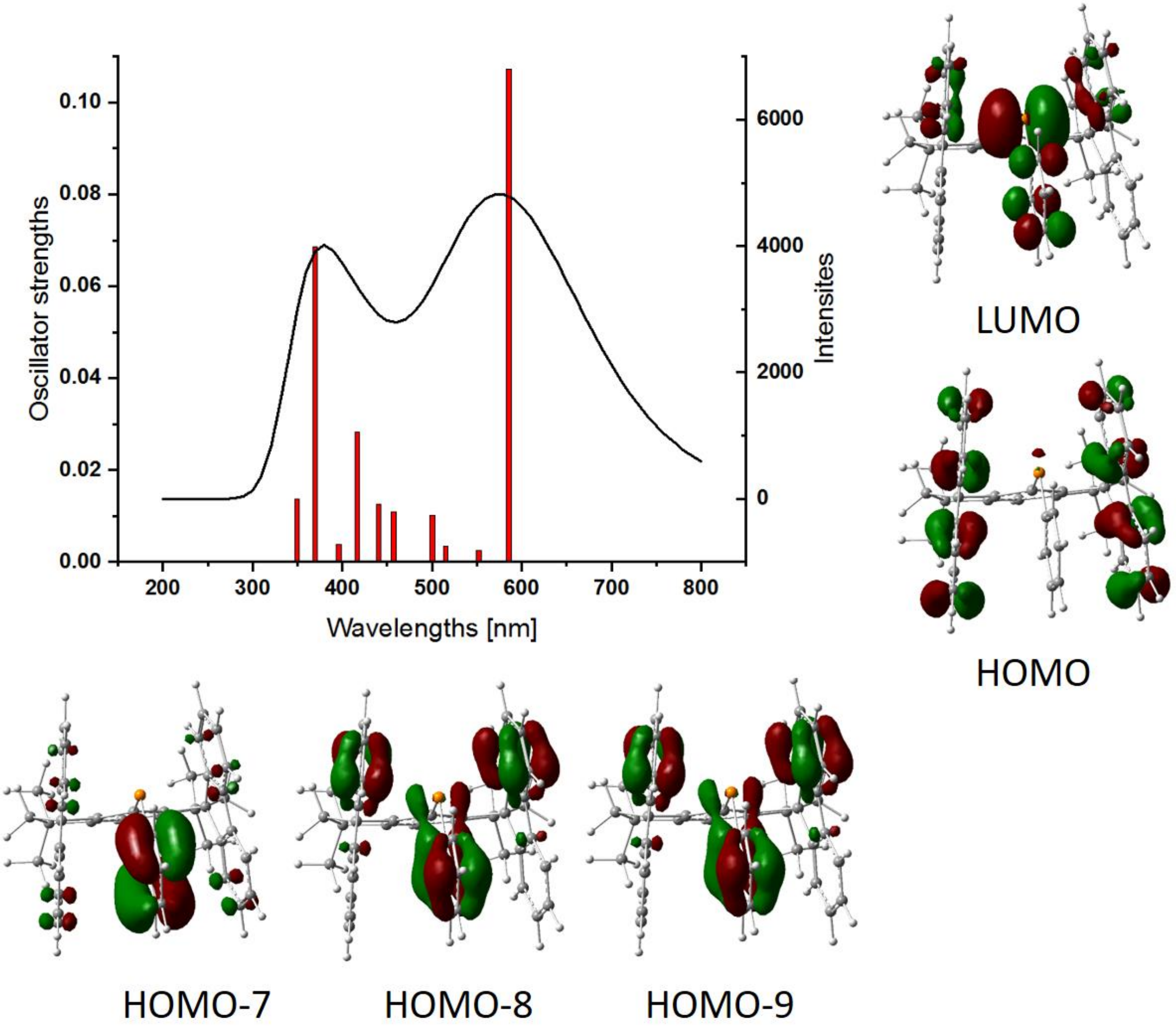

Figure S95.

Predicted UV spectrum (black line) and oscillator strengths (red bars) of **8** as well as MOs relevant for the two major transitions at about 370 nm (HOMO-9/HOMO-8/HOMO-7 → LUMO) and 590 nm (HOMO → LUMO); *iso*-surface values at 0.04 a.u. for every MO.

Additional references

[1] T. Matsuo, K. Suzuki, T. Fukawa, B. Li, M. Ito, Y. Shoji, T. Otani, L. Li, M. Kobayashi, M. Hachiya, Y. Tahara, D. Hashizume, T. Fukunaga, A. Fukazawa, Y. Li, H. Tsuji, K. Tamao, *Bull Chem. Soc. Jpn*., **2011**, *84*, 1178–1191.

[2] A. Hübner, T. Bernert, I. Sänger, E. Alig, M. Bolte, L. Fink, M. Wagner, H.-W. Lerner, *Dalton Trans.*, **2010**, *39*, 7528–7533.

[3] P. Romanato, S. Duttwyler, A. Linden, K. K. Baldridge, J. S. Siegel, *J. Am. Chem. Soc.*, **2010**, *132*, 7828−7829.

[5] G. R. Fulmer, A. J. M. Miller, N. H. Sherden, H. E. Gottlieb, A. Nudelman, B. M. Stoltz,, J. E. Bercaw, K. I. Goldberg, *Organometallics*, **2010**, *29*, 2176–2179.

[6] M. Ito, D. Hashizume, T. Fukunaga, T. Matsuo, K. Tamao, *J. Am. Chem. Soc.*, **2009**, *131*, 18024–18025.

[7] D. B. G. Williams, M. Lawton, *J. Org. Chem.*, **2010**, *75*, 8351–8354.

[8] U. Mayer, V. Gutmann, W. Gerger, *Monat. Chem.*, **1975**, *106*, 1235–1257.

[9] M. Beckett, G. Strickland, *Polym. Commun*., **1996**, *37*, 4629–4631.

[10] V. Gutmann, *Coord. Chem. Rev.*, **1976**, *18*, 225–255.

[11] G. M. Sheldrick, *Acta Cryst*., **2008**, *A64*, 112.

[12] L. Farrugia, *J. Appl. Cryst*., **1999**, *32*, 837.

[13] O. V. Dolomanov, L. J. Bourhis, R. J. Gildea, J. A. K. Howard, H. Puschmann, *J. Appl. Cryst.*, **2009**, *42*, 339–341.

[14] K. Brandenburg, Diamond, version 4.0.4, Crystal Impact GbR: Bonn, Germany, **2012**.

[15] J. P. Perdew, J. A. Chevary, S. H. Vosko, K. A. Jackson, M. R. Pederson, D. J. Singh, C. Fiolhais, *Phys. Rev*. *B*, **1992**, *46*, 6671–6687.

[16] A. D. Becke, *J. Chem. Phys*., **1993**, *98*, 5648–5652.

[17] M. J. Frisch, G. W. Trucks, H. B. Schlegel, G. E. Scuseria, M. A. Robb, J. R. Cheeseman, G. Scalmani, V. Barone, G. A. Petersson, H. Nakatsuji, X. Li, M. Caricato, A. V. Marenich, J. Bloino, B. G. Janesko, R. Gomperts, B. Mennucci, H. P. Hratchian, J. V. Ortiz, A. F. Izmaylov, J. L. Sonnenberg, D. Williams-Young, F. Ding, F. Lipparini, F. Egidi, J. Goings, B. Peng, A. Petrone, T. Henderson, D. Ranasinghe, V. G. Zakrzewski, J. Gao, N. Rega, G. Zheng, W. Liang, M. Hada, M. Ehara, K. Toyota, R. Fukuda, J. Hasegawa, M. Ishida, T. Nakajima, Y. Honda, O. Kitao, H. Nakai, T. Vreven, K. Throssell, J. A. Montgomery, Jr., J. E. Peralta, F. Ogliaro, M. J. Bearpark, J. J. Heyd, E. N. Brothers, K. N. Kudin, V. N. Staroverov, T. A. Keith, R. Kobayashi, J. Normand, K. Raghavachari, A. P. Rendell, J. C. Burant, S. S. Iyengar, J. Tomasi, M. Cossi, J. M. Millam, M. Klene, C. Adamo, R. Cammi, J. W. Ochterski, R. L. Martin, K. Morokuma, O. Farkas, J. B. Foresman, and D. J. Fox, Gaussian, Inc., Wallingford CT, **2016**.

[18] R. W. F. Bader, *Atoms in Molecules: A Quantum Theory* (Cambridge University Press: Oxford U.K., 1991).

[19] F. Biegler-König, J. Schönbohm, D. Bayles, *J. Comput. Chem*., **2001**, *22*, 545–559.

[20] M. Kohout, DGRID-4.6 Radebeul, **2015**.

[21] M. Kohout, *Int. J. Quantum Chem.*, **2004**, *97*, 651–658.

[22] M. Kohout, F. R. Wagner, Y. Grin, *Theor. Chem. Acc.*, **2008**, *119*, 413–420.

[23] E. R. Johnson, S. Keinan, P. Mori-Sanchez, J. Contreras-García, A. J. Cohen, W. Yang, *J. Am. Chem. Soc.*, **2010**, *132*, 6498–6506.

[24] J. Contreras-García, E. Johnson, S. Keinan, R. Chaudret, J.-P. Piquemal, D. Beratan, W. Yang, *J. Chem. Theor. Comp.*, **2011**, *7*, 625–632.

[25] J. P. Foster, F. Weinhold, *J. Am. Chem. Soc.*, **1980**, *102*, 7211–7218.

[26] A. E. Reed, L. A. Curtiss, F. Weinhold, *Chem. Rev.*, **1988**, *88*, 899–926.

[27] E. D. Glendening, J. K. Badenhoop, A. E. Reed, J. E. Carpenter, J. A. Bohmann, C. M. Morales, F. Weinhold, NBO 5.9, Theoretical Chemistry Institute, University of Wisconsin, Madison, WI, **2009**; http://www.chem.wisc.edu/~nbo5

[28] C. B. Hübschle, P. Luger, *J. Appl. Crystallogr*., **2006**, *39*, 901–904.
